# Acetylene in Organic Synthesis: Recent Progress and New Uses

**DOI:** 10.3390/molecules23102442

**Published:** 2018-09-24

**Authors:** Vladimir V. Voronin, Maria S. Ledovskaya, Alexander S. Bogachenkov, Konstantin S. Rodygin, Valentine P. Ananikov

**Affiliations:** 1 Institute of Chemistry, Saint Petersburg State University, Universitetsky prospect 26, Peterhof 198504, Russia; volgo-vv@mail.ru (V.V.V.); maria.s.ledovskaya@gmail.com (M.S.L.); alexterve@gmail.com (A.S.B.); konstantinrs@rambler.ru (K.S.R.); 2 N. D. Zelinsky Institute of Organic Chemistry Russian Academy of Sciences, Leninsky prospect 47, Moscow 119991, Russia

**Keywords:** acetylene, vinylation, cross-coupling, addition reactions, drugs, pharmaceutical substances, biologically active molecule, monomers, polymers

## Abstract

Recent progress in the leading synthetic applications of acetylene is discussed from the prospect of rapid development and novel opportunities. A diversity of reactions involving the acetylene molecule to carry out vinylation processes, cross-coupling reactions, synthesis of substituted alkynes, preparation of heterocycles and the construction of a number of functionalized molecules with different levels of molecular complexity were recently studied. Of particular importance is the utilization of acetylene in the synthesis of pharmaceutical substances and drugs. The increasing interest in acetylene and its involvement in organic transformations highlights a fascinating renaissance of this simplest alkyne molecule.

## 1. Introduction

Since the discovery of acetylene, new areas of acetylene chemistry have been continuously developed. The rich scope of chemical transformations available for a C≡C triple bond can be exemplified by coupling [1,2,3,4,5] and addition reactions [6,7]. The removal of trace acetylene from ethylene has led to another important process, selective hydrogenation of acetylene [8]. Recently, several opportunities of controlled accommodation of acetylene in microporous materials have appeared [9]. New functional materials allow separating, sensing and storing acetylene and other small molecules [10,11].

Acetylene, or ethyne, with the molecular formula C_2_H_2_, is the simplest alkyne. Due to its structural simplicity and high reactivity, acetylene represents a versatile building block for organic synthesis, and its chemistry has evolved rapidly [12]. In recent reviews [13,14] acetylene is compared with ethylene. Acetylene is commonly obtained from coal and natural gas and these sources will last longer than current oil sources. Acetylene chemistry is therefore of high importance in various aspects [15].

Acetylene can be produced by several well-established methods. One of them is based on the reaction of calcium carbide (obtained from coal and lime) with water [16]. The other methods include partial combustion of hydrocarbons, electrothermal cracking in an electric arc furnace, and thermal cracking with heat carriers [14,16].

Pure acetylene is a colorless gas with no odor. It is lightweight and flammable, with a high combustion temperature [17]. Due to this, acetylene is widely used in welding [16,18]. It is also suitable for brazing, cutting, hardening, texturing, and thermal spraying of many materials. Besides, the acetylene flame is extraordinary bright, which formerly contributed to the wide use of carbide lamps. The term “carbide lamp” reflects the interaction of calcium carbide with water, followed by burning of the released acetylene in another compartment of the device [19].

**Scheme 1:**
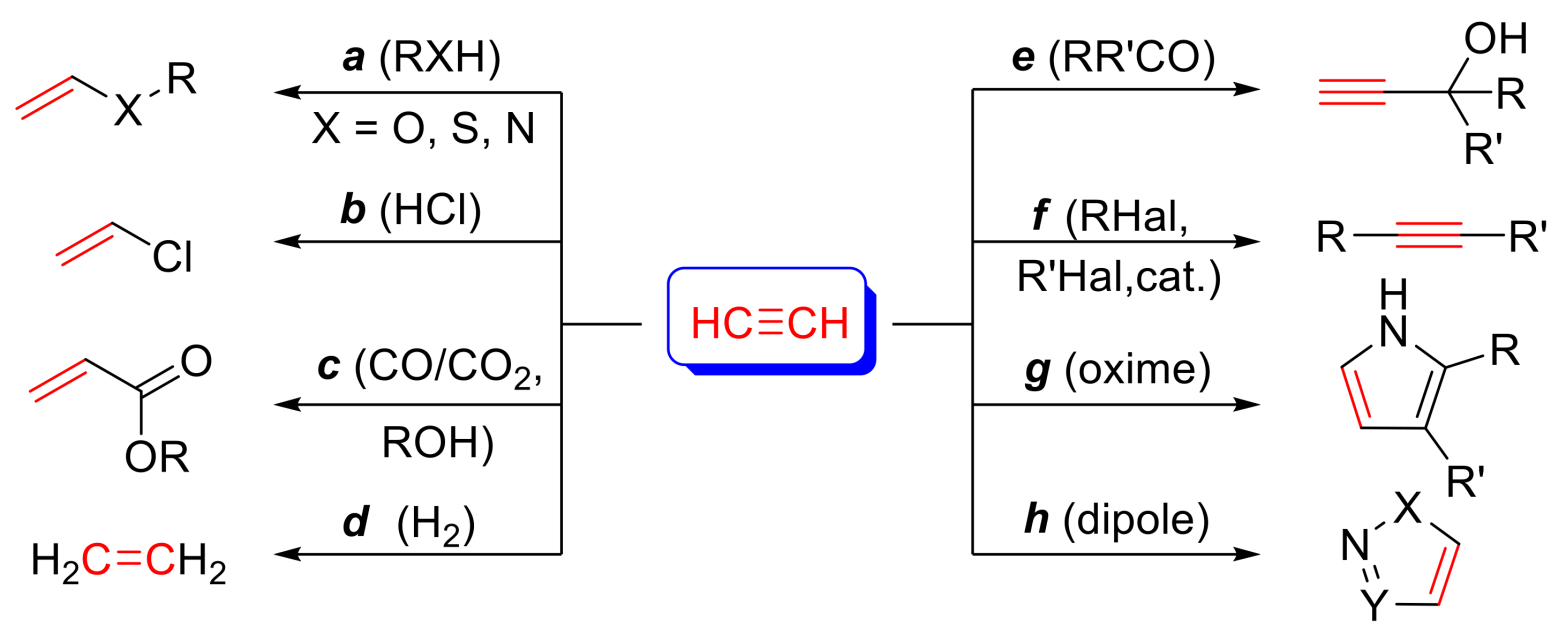
Representative examples in acetylene chemistry.

Chemical applications of acetylene are diverse, and a brief summarized scope is shown in Scheme 1. Some of them are of great practical relevance, e.g., the vinylation reaction (Scheme 1, path *a*) [20], a common route to vinyl-containing monomers for polymeric materials production. Polyvinyl derivatives are indispensable components of many varnishes, paints, adhesives, packing materials [15], in electroluminescent materials [21], coatings or fillers for pharmaceutical excipients and many more [22].

Hydrochlorination of acetylene (Scheme 1, path *b*), or addition of hydrochloric acid, is the simplest and widely used route to vinyl chloride [16], a commodity chemical used for production of the polyvinyl chloride resins [23]. Polyvinyl chloride is the third most commonly used sort of plastic, behind polyethylene and polypropylene, and the global demand for it continues to increase [24]. 

The other way of industrial acetylene utilization is carbonylation (Scheme 1, path *c*). By this way acrylic acid and derivatives can be obtained [25]. Produced on a global scale of more than three million tons a year, acrylic acid is widely used as a basic material for paints, plastics, superabsorbent polymers, and rubbers [26,27,28]. Partial reduction of acetylene (Scheme 1, path *d*) provides alternative means for ethylene synthesis [29,30,31,32,33]. This reaction has industrial prospects that will be discussed in detail.

Ethynylation with acetylene (Scheme 1, paths *e* and *f*) is a powerful approach to prepare substituted acetylenes. Ethynylation strategies include the base-catalyzed addition of acetylene to carbonyl compounds (Favorskii reaction, path *e*) [34,35] and various cross-coupling reactions (path *f*) [36,37,38]. 

Addition of ketoximes to acetylene (Scheme 1, path *g*) provides an access to a range of pyrroles, indoles, and related compounds [13,39] for applications in pharmaceutical chemistry, polymer synthesis, and dye manufacturing [21,22,40].

Cycloaddition reactions with acetylene (Scheme 1, path *h*) provide a shortcut to isoxazoles, pyrazoles, and triazoles [41,42,43,44]. This segment of acetylene chemistry is especially promising for obtaining novel heterocyclic molecules. 

This review discusses recent advances in acetylene chemistry by describing the most important processes at various levels of chemical complexity. Recent literature within the time period of 2013–2018 was considered to highlight emerging directions and applications in organic chemistry. Representative examples in each research direction are provided to cover most important trends in synthetic acetylene chemistry. It should be emphasized that the present review is focused on the reactions of acetylene (C_2_H_2_)—a fascinating molecule with significant practical importance and future potential. Reactions involving substituted alkynes have been described in details elsewhere and these discussions were not repeated here.

## 2. Acetylene in Organic Synthesis

Versatile and ubiquitously used synthetic transformations of acetylene involve functionalization of the C≡C bond; these processes are considered first (Section 2.1). Next, access to acrylic acid derivatives (Section 2.2) and preparation of substituted alkynes (Section 2.3) are discussed, followed by preparation of heterocyclic molecules (Section 2.4). Selected applications of acetylene in the synthesis of drugs and pharmaceutical substances is highlighted in a separate section (Section 2.5). Synthetic organic and organometallic reactions are also considered (Section 2.6 and Section 2.7), followed by industry-related reactions involving hydrogenation, hydrochlorination reactions and their mechanisms (Section 2.8 and Section 2.9). Finally, useful emerging concepts from the chemistry of alkynes are briefly mentioned (Section 2.10).

### 2.1. Functionalization of Triple Bond

Polymerization of vinyl-containing substrates has been a subject of close attention for many years. Polyvinyl compounds constitute a basis of many composites including paints, adhesives, varnishes, packaging [15], electroluminescent materials [21], pill coatings and many more [22]. Despite the widest range of applications, their chemistry is still in progress. Vinyl ethers, thioethers, sulfur- and nitrogen analogs become increasingly involved in synthesis of various cyclic compounds [45,46,47,48,49,50], natural substances, and medicinal chemicals [51,52], hence the demand for their development, modification, and systematization. A number of studies have been devoted to metathesis [53,54] and cross-coupling reactions of vinyl derivatives [55,56]. This section presents a review of the latest achievements in this field. 

#### 2.1.1. Synthesis of Vinyl Ethers

Addition of alcohols to acetylenes was introduced by Favorskii [57,58]. While studied isomerization of alkynes in the presence of alcohols and alkalis, he found that the alcohols react with acetylenes to give ethers. Later on, this reaction was turned into an industrial method for vinyl ether synthesis based on the addition of alcohols to acetylene, known as vinylation [14,20]. Under industrial conditions, it is carried out in autoclaves at elevated pressures and high temperatures in the presence of alkaline catalysts [13,14]. It can be performed on a smaller scale in special laboratory reactors or standard flasks and tubes. In the laboratory practice of the last decade, gaseous acetylene is frequently and effectively replaced with calcium carbide as an acetylene source. Calcium carbide readily reacts with water and produces gaseous acetylene [59]. Here we will consider the recent examples of acetylene and carbide usage in vinyl ether synthesis.

**Scheme 2:**
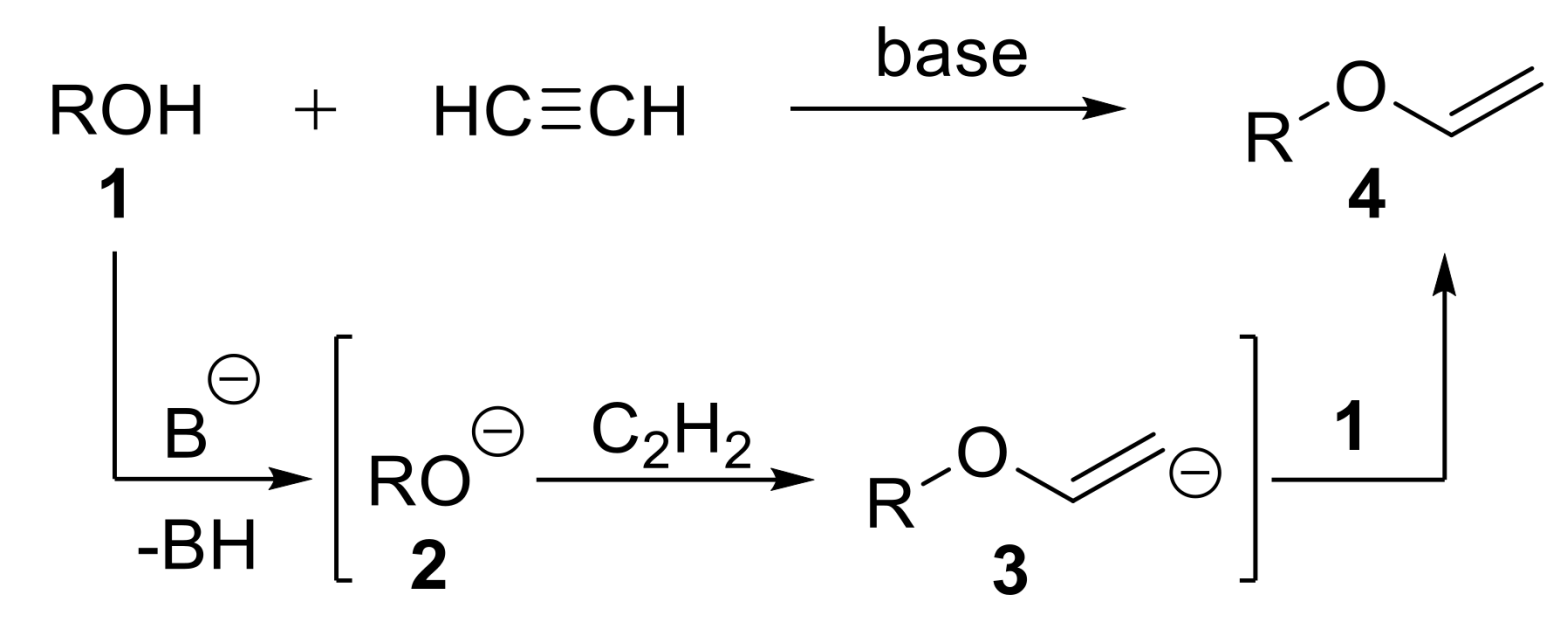
Synthesis of vinyl ethers involving acetylene.

The reaction of alcohols with acetylene can be classified as nucleophilic addition to carbon-carbon triple bond (Scheme 2). This process requires the presence of a base as a catalyst. The base interacts with an alcohol molecule **1** giving alcoholate anion **2**. The alkoxide ion reacts with acetylene forming the corresponding carbanion **3**. The latter acts with the molecule of alcohol or water, thus giving vinyl ether **4** [20,60]. 

**Scheme 3:**
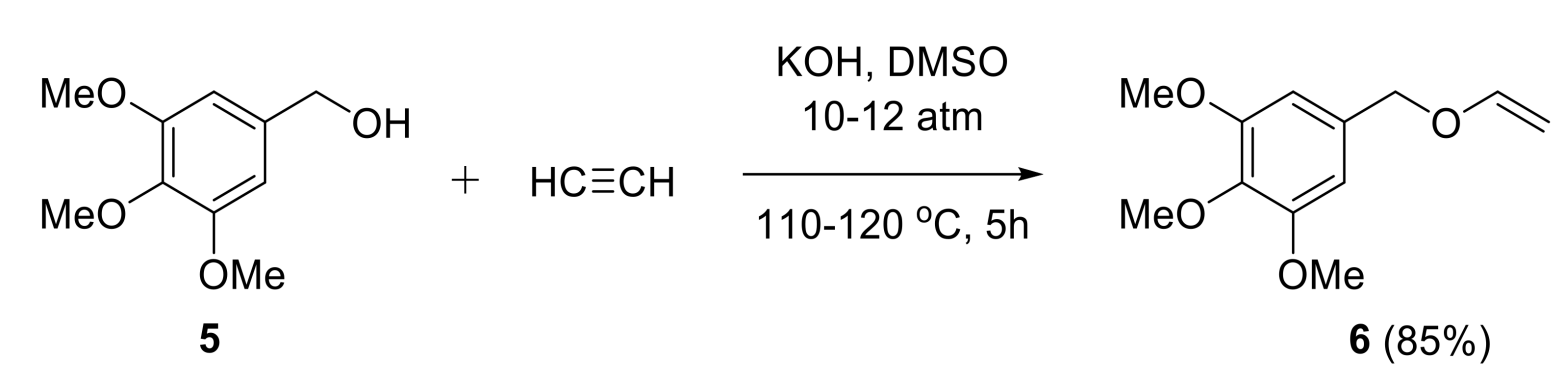
3,4,5-Trimethoxybenzyl vinyl ether synthesis.

An effective vinylation methodology for 3,4,5-trimethoxybenzyl alcohol (**5**) was proposed [61]. The resulting 3,4,5-trimethoxybenzyl vinyl ether (**6**) is a promising starting material for drug synthesis. The reaction was carried out under the standard vinylation conditions [20]: superbasic KOH-DMSO system as a reaction media, high temperature and elevated acetylene pressure. The desired vinyl derivative was isolated in 85% yield (Scheme 3). 

**Scheme 4:**
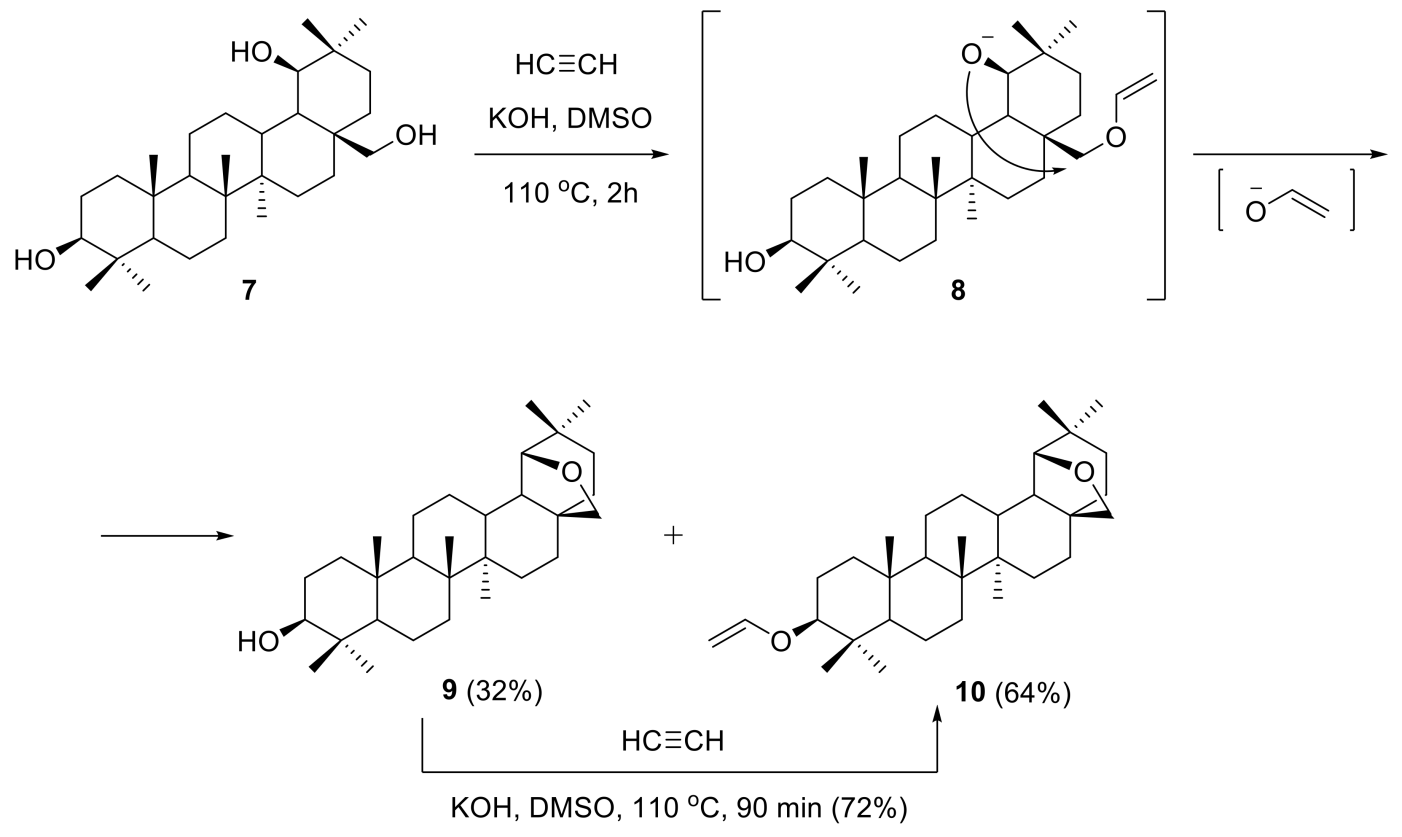
Triterpenoid vinylation.

Vinylation of triterpenoid **7** under atmospheric acetylene pressure resulted in vinyl derivative **10** in good yield (64%) [62]. Formation of the oxygen bridge was thought to occur through intramolecular formation of vinyl ether **8** with its subsequent devinylation-cyclization and vinylation of the last OH-group (Scheme 4). The side product **9** with a free hydroxyl group can be easily converted to the desired ether **10**.

**Scheme 5:**
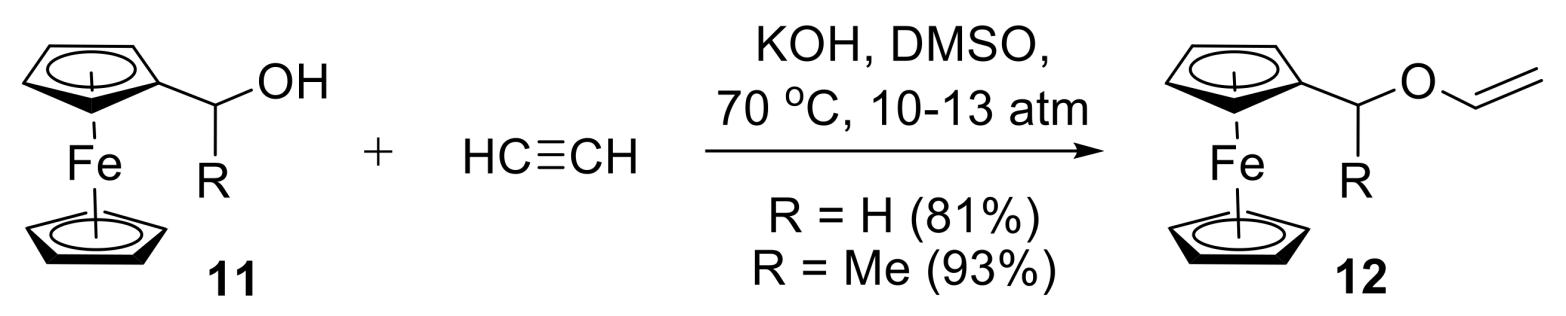
Reaction of ferrocene-derived alcohols with acetylene.

Another recently published example of superbase-catalyzed (KOH-DMSO system-mediated) vinylation involves hydroxymethyl- and α-hydroxyethylferrocenes **11** [63]. Vinyl ethers **12** were obtained in excellent yields (Scheme 5) and represented a promising route for development of new polymeric materials with specific physical and chemical properties.

**Scheme 6:**
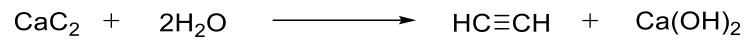
Reaction of calcium carbide with water.

Despite the outstanding chemical versatility of acetylene and the widest range of possibilities for its application, it is a highly flammable gaseous substance [64]. Safety precautions should be taken to avoid its leakage and explosion. Besides, gaseous materials usage requires additional measuring equipment and high-pressure furnishing. The development of alternatives for gaseous acetylene is an important problem of modern chemistry. A reasonable solution of these complications is provided by utilization of calcium carbide as an in situ source of acetylene, which is immediately released upon reaction of CaC_2_ with water (Scheme 6) [59]. A small admixtures of water does not interfere with the vinylation, and sometimes is even favorable [60,65]. 

**Scheme 7:**
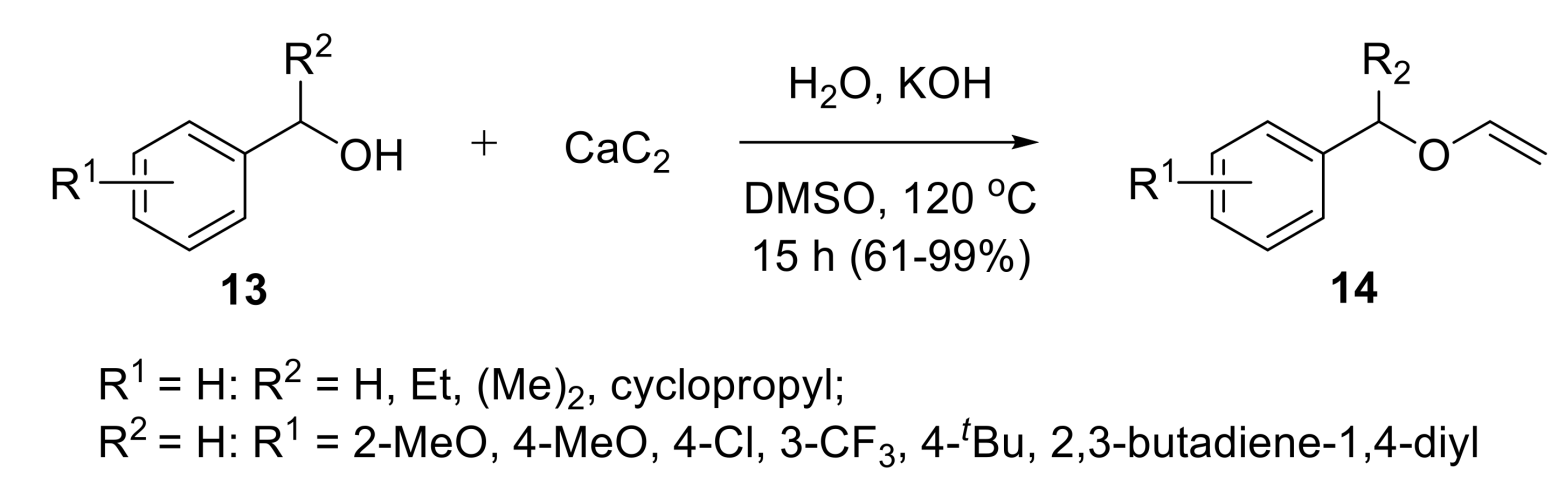
Vinylation of benzyl alcohols with calcium carbide.

Successful vinylation of benzyl alcohols **13** with calcium carbide was performed without any complicate equipment [66]. The reactions were conducted in standard reaction tubes or in a flask for gram-scale synthesis. The desired vinyl ethers **14** were obtained in good to excellent yields (Scheme 7). Using CaC_2_ instead of gaseous acetylene provides certain economy, as only 2.5 to 3.3-fold excess of it is required to complete the reaction. Similar reactions with gaseous acetylene (see Scheme 3, Scheme 4 and Scheme 5) were carried out under high acetylene pressures (10-13 atm) or in acetylene flow [61,62,63].

**Scheme 8:**
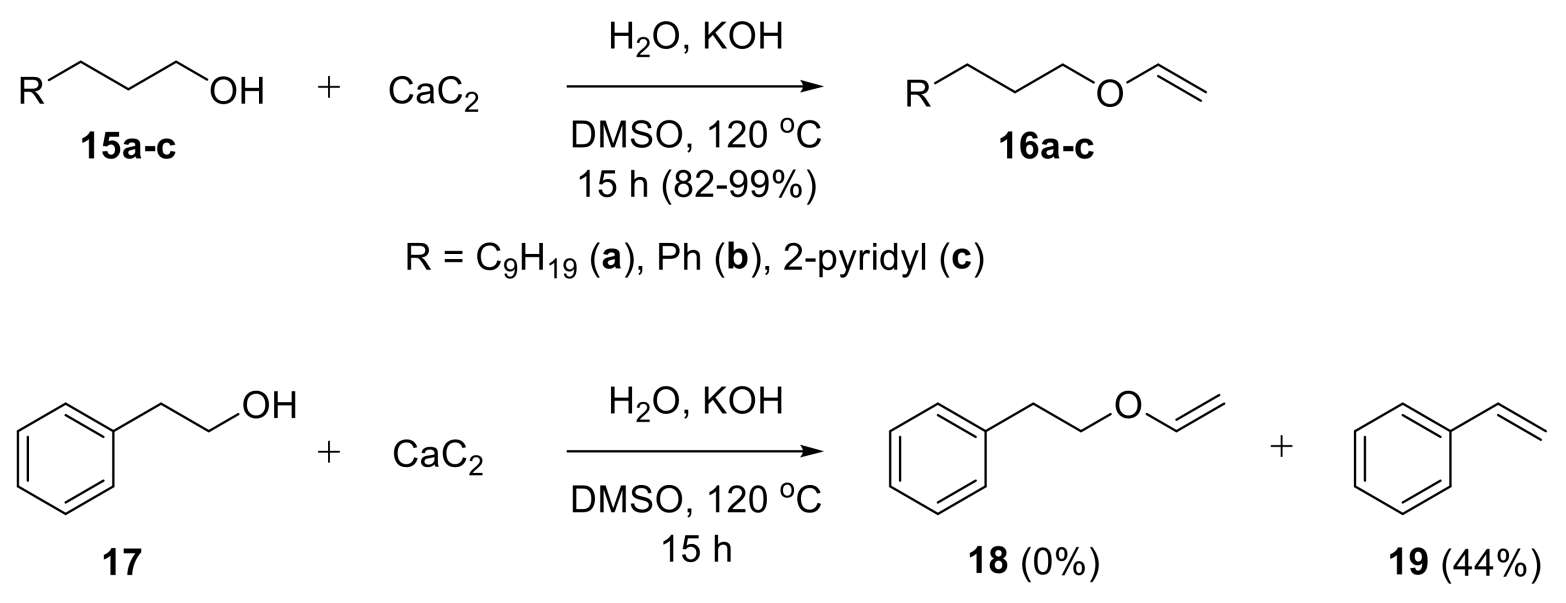
Vinylation of alcohols with calcium carbide.

Apart from benzyl alcohols, the successful vinylation of dodecyl alcohol (**15a**), 3-phenylpropanol (**15b**) and 3-(pyridine-2-yl)propanol (**15c**) was also performed under the same conditions (Scheme 8) [66]. Vinyl ethers **16a-c** were obtained in very good yields. For phenethyl alcohol (**17**), the unexpected formation of styrene **19** instead of vinyl ether **18** was observed (Scheme 8).

**Scheme 9:**
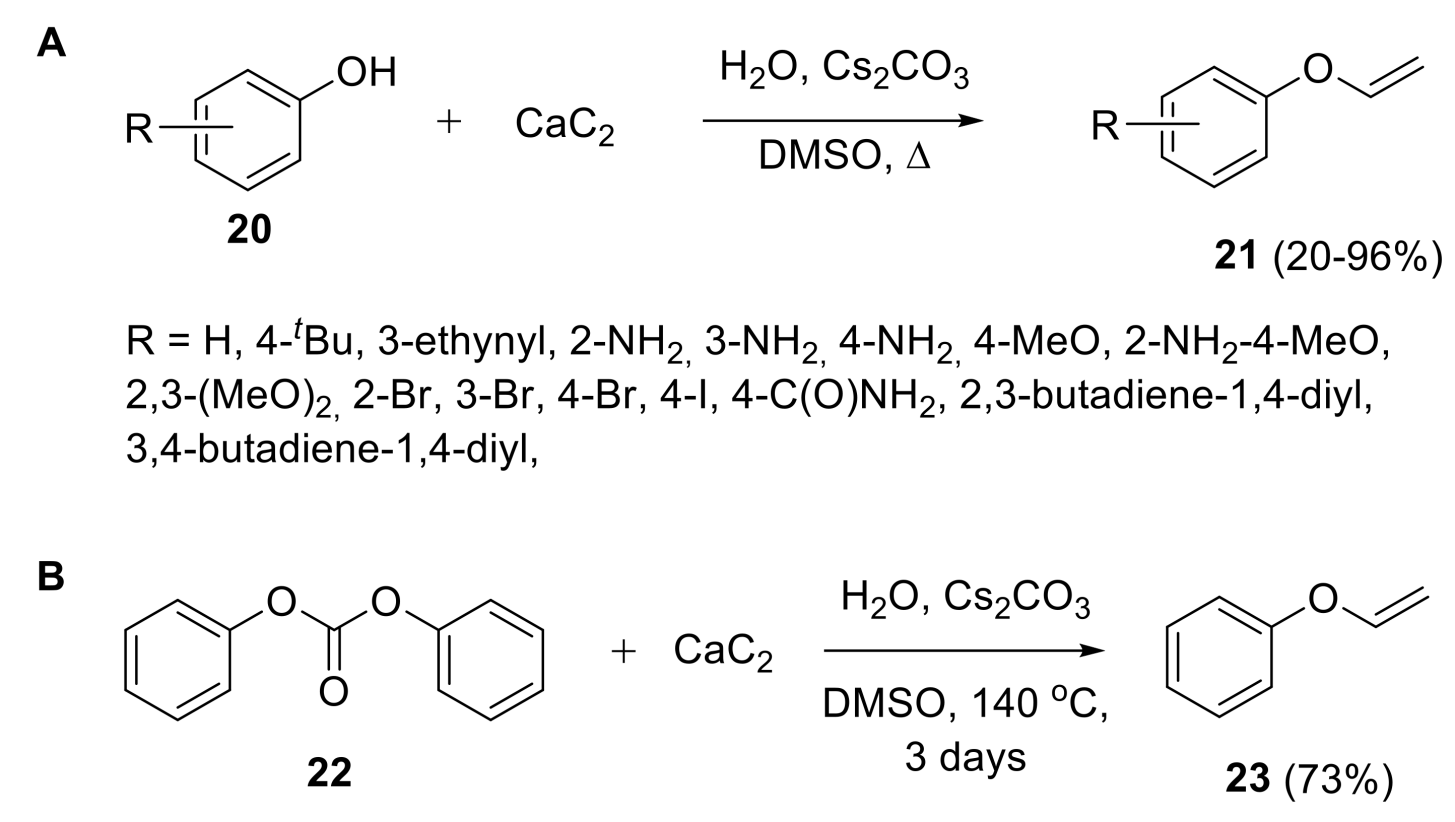
The synthesis of aryl vinyl ethers with calcium carbide.

The reaction of phenols **20** with calcium carbide as an acetylene source was carried out at similar conditions [67,68]. Apparently, Cs_2_CO_3_ is also capable of providing a superbasic system with DMSO. The method allows obtaining vinyl derivatives **21** in up to 96% yields (Scheme 9A). Using the developed technique, diphenyl carbonate **22** was also transformed to phenyl vinyl ether (**23**) in 73% yield (see Scheme 9B) [67]. 

**Scheme 10:**
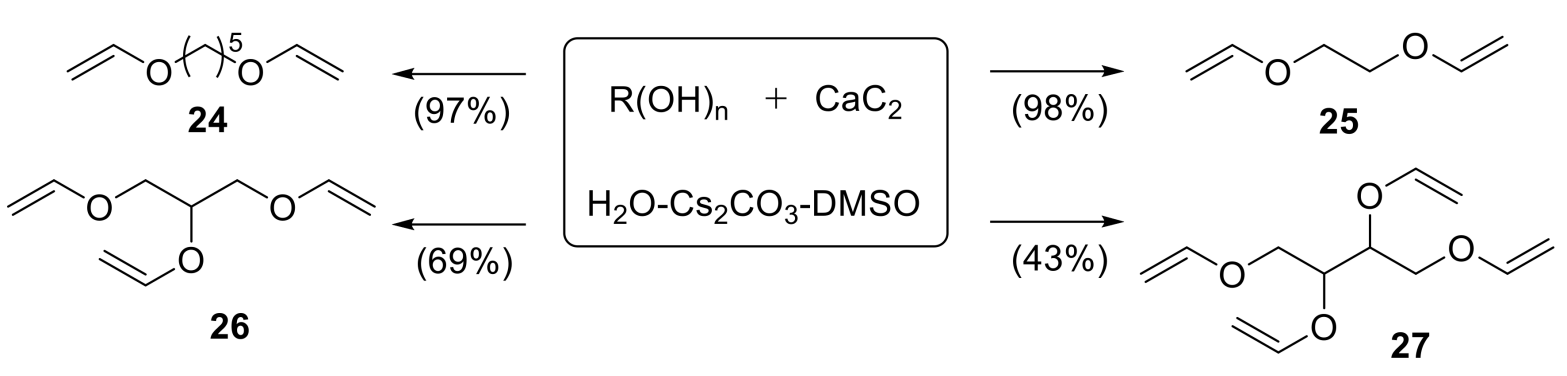
Polyvinyl ethers synthesis with in situ generated acetylene.

The vinylation of 1,5-pentanediol, ethylene glycol, glycerol and erythritol with carbide was also performed in a superbasic Cs_2_CO_3_-DMSO system [67]. The corresponding vinyl derivatives **24**–**27** were obtained in moderate to excellent yields (Scheme 10).

**Scheme 11:**
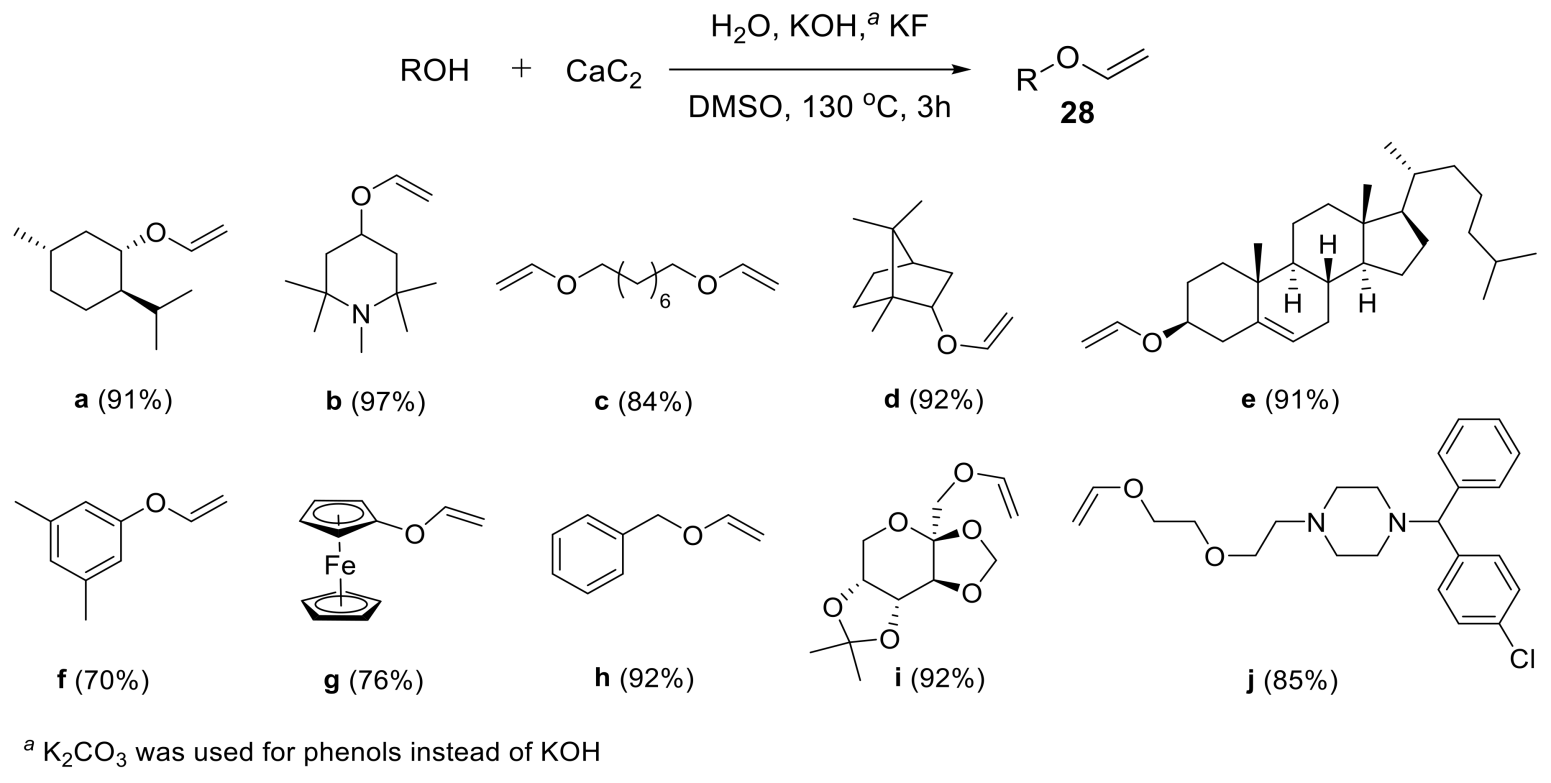
Representative scope of alcohols vinylation with CaC_2_ in KOH-DMSO-KF system.

Another synthetic procedure for vinylation of alcohols and phenols was recently developed [69,70]. It employs KOH-DMSO-KF and K_2_CO_3_-DMSO-KF ternary mixtures instead of the renowned KOH-DMSO and Cs_2_CO_3_-DMSO systems. Potassium fluoride significantly accelerates the reaction [70]. The KOH-DMSO-KF mixture works well for aliphatic alcohols, particularly steroids and carbohydrates, giving vinyl ethers **28** in good to excellent yields (Scheme 11) [69,70]. It also favors the reaction with ferrocenyl alcohol and allows obtaining its vinylated derivative **28g** in 76% yield. The alternative K_2_CO_3_-DMSO-KF system is perfect for vinylation of phenols.

In general, both acetylene and calcium carbide as an acetylene source can be used in vinyl ether synthesis. The reaction of alcohols with acetylene or CaC_2_ is applicable to a wide variety of substrates. It can effectively proceed with aliphatic substrates, phenols, naphthols, ferrocene-derived alcohols, nitrogen-containing substrates, carbohydrates, etc. Using calcium carbide in a laboratory is preferable for its convenience and safety, as CaC_2_ is a solid material that can be easily quantified and handled without special equipment. Direct addition of carbide into the mixture allows carrying out the reaction under milder conditions. When using gaseous acetylene, the expenditure is considerably higher, even under low pressures, because the constant gas flow must be maintained until the end of the reaction.

#### 2.1.2. Synthesis of S-, Se- and Te-Vinyl Derivatives

Organochalcogen compounds occupy an important niche in contemporary organic synthesis due to their availability, rich chemistry [71,72,73] and the biological activities [74]. Among the organochalcogen compounds, vinyl chalcogenides have attracted a considerable attention in recent years [75,76,77]. Vinyl sulfides are of particular interest as intermediates for various organic transformations. Sulfur-containing polymers derived from vinyl monomers have attracted a great deal of attention, due to their potential applications in numerous areas in polymer research. For example, they have a wide variety of applications in plastic lenses, optical disc substrates, optical adhesives or encapsulants, antireflective coatings, photoresists [75,76,77,78,79]. 

Addition of thiols and selenols to acetylene is the simplest route to vinyl sulfides and vinyl selenides. It is chemically similar with the nucleophilic addition of alcohols to acetylene described previously and it is also require basic catalysis [20], and is accordingly called thiovinylation.

**Scheme 12:**
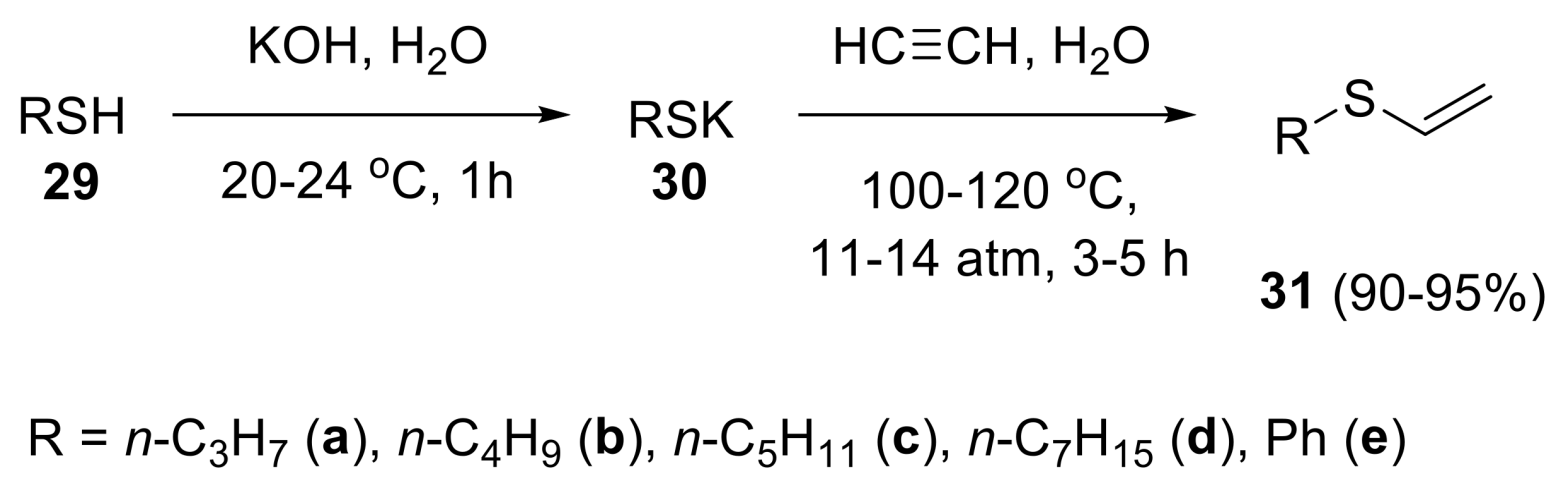
Addition of thiols to acetylene.

The common approach to this reaction based on the use of gaseous acetylene was proposed recently. The simplest alkyl vinyl sulfides **31a**–**d** and phenyl vinyl sulfide **31e** were synthesized in excellent yields [80]. On the first step, generation of potassium thiolate **30** from thiol **29** occurred (Scheme 12). Potassium thiolate further reacted with acetylene and water giving vinyl ether **31**. This approach can be considered as green, because the reactions were performed in aqueous media. It is probably applicable to a wide range of thiols. Its serious disadvantage is the high acetylene pressure requirement.

**Scheme 13:**
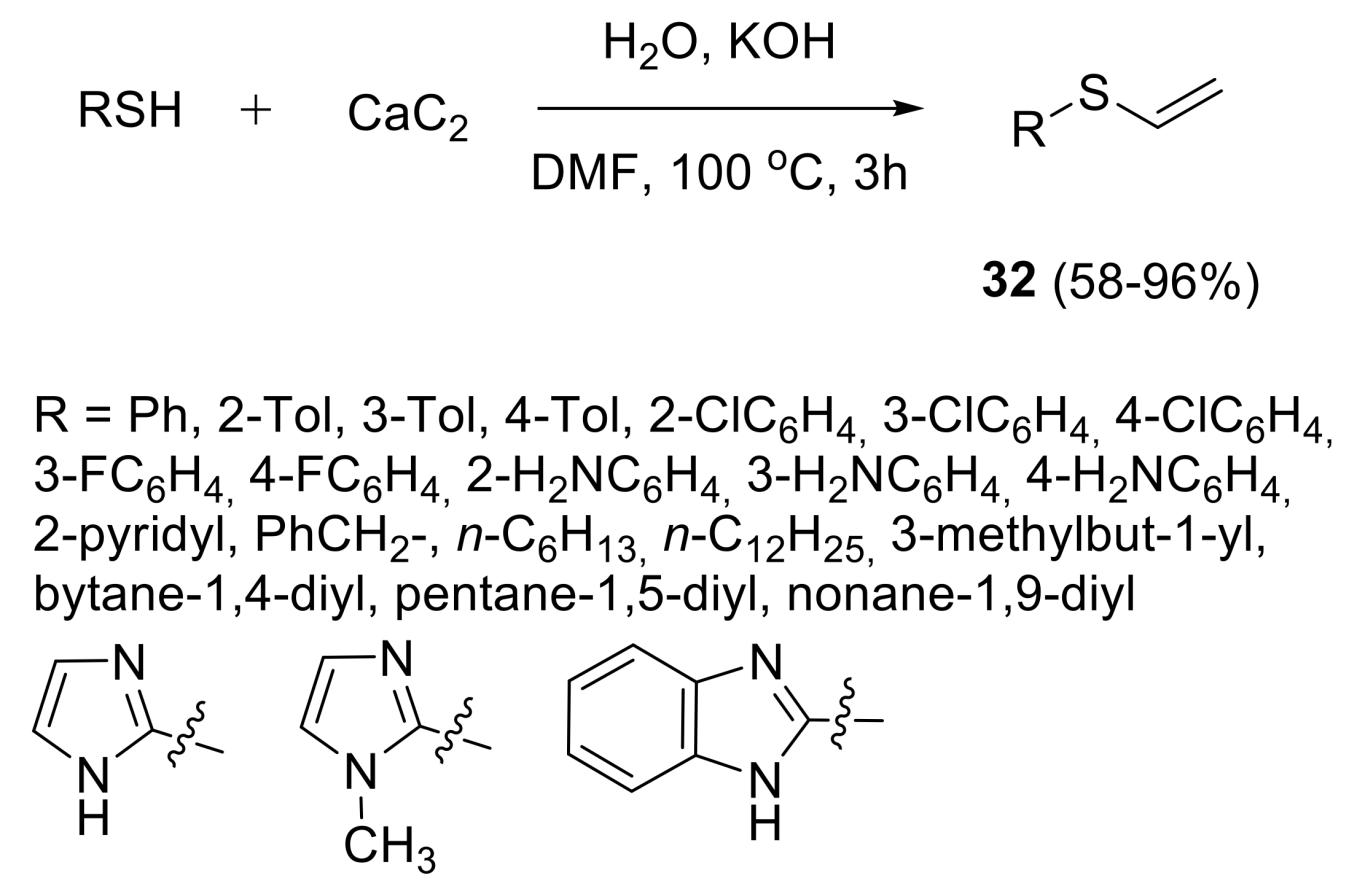
Reactions of carbide-induced acetylene with thiols.

The same is possible with calcium carbide as an acetylene source [81,82]. The wide substrate scope of CaC_2_-mediated thiovinylation includes thiophenols [81], alkyl thiols [81], dithiols [82], and imidazole and benzimidazole thiols (Scheme 13) [82]. For most of them, vinyl sulfides **32** were obtained in excellent yields (88–96%), with exceptions of the pyridine-2-thiol and 1-methylimidazole-2-thiol addition to acetylene that resulted in 72% and 58% yields, respectively.

**Scheme 14:**
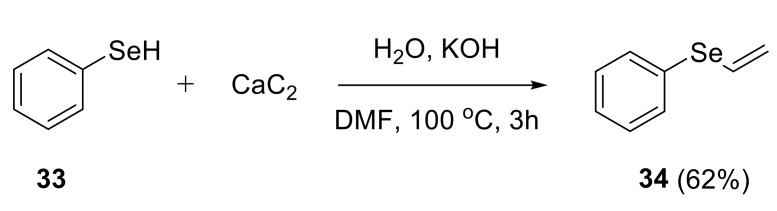
Phenyl vinyl selenide synthesis with calcium carbide.

Phenyl selenol (**33**) was also modified by calcium carbide. Vinyl derivative **34** was obtained in 62% yield (Scheme 14) [81]. 

**Scheme 15:**
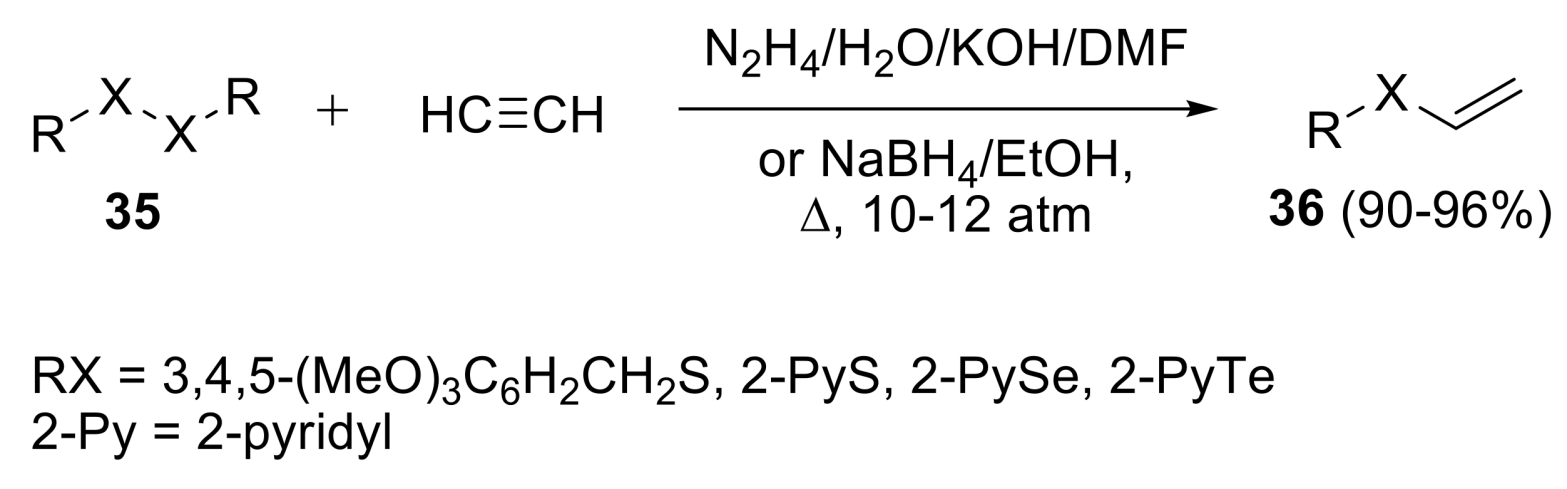
Vinyl chalcogenide synthesis.

Another reaction, which is also acetylene-dependent, allows obtaining vinyl chalcogenides from disulfides, diselenides, and ditellurides; it leads to vinyl sulfides, vinyl selenides, and vinyl tellurides, respectively. It has several reported implementations [61,83,84]. The first resulted from interaction of a dichalcogenide **35** with gaseous acetylene in the presence of a reducing agent, e.g., hydrazine-KOH mixture or sodium borohydride. To complete the reaction, the presence of the reducing agent is required (Scheme 15) [61,84]. Using this methodology, the corresponding vinyl chalcogenides **36** were obtained in excellent yields.

**Scheme 16:**
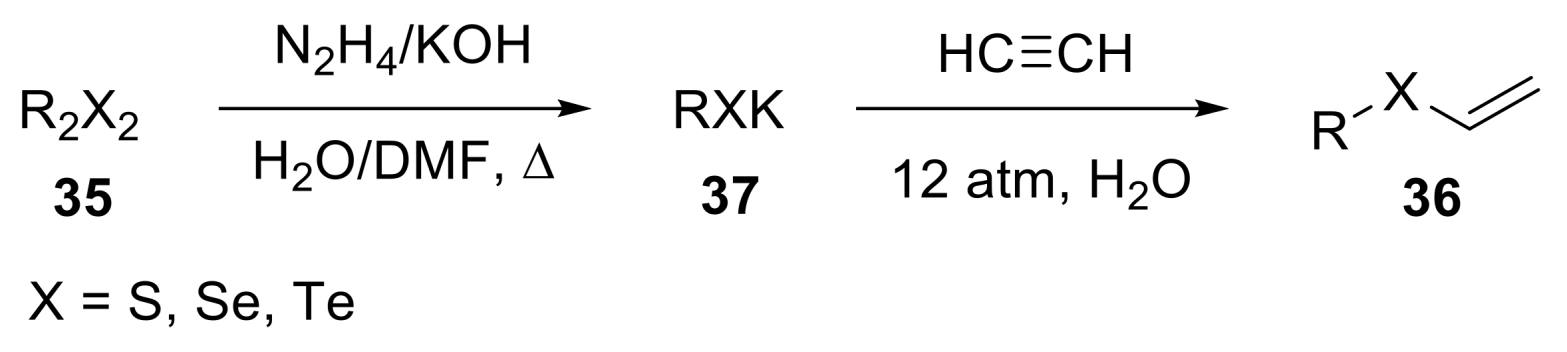
The mechanism of vinyl chalcogenides formation.

The reaction proceeds in two steps. At first, dichalcogenide **35** interacts with the reducing-basic mixture giving potassium chalcogenide **37** (Scheme 16), which further reacts with acetylene and water to produce vinyl derivative **36** [84]. 

Another variation on the theme was recently proposed [61,83,84]. In this setting, alkyl or aryl halide was directly exposed to sulfur, selenium or tellurium one-flask with a reducing agent and acetylene. Heating of this mixture under high acetylene pressure promoted formation of vinyl chalcogenides in moderate yields (52–68%) [83,84].

**Scheme 17:**
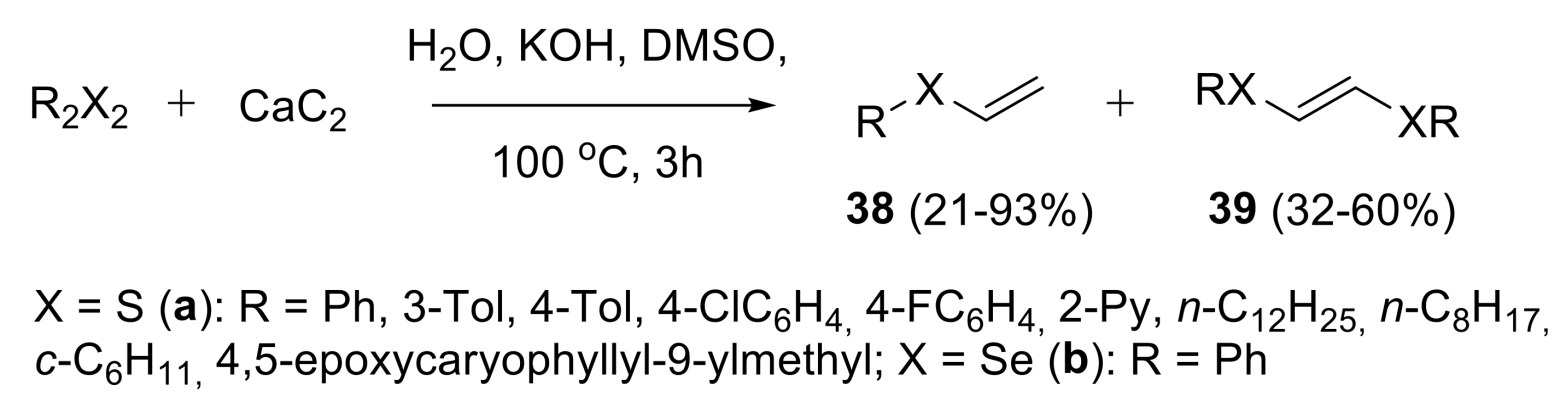
Reactions of carbide-induced acetylene with dichalcogenides.

The third protocol for synthesis of vinyl chalcogenides from dichalcogenides and acetylene is based on the in situ generation of the acetylene itself. As a source of acetylene calcium carbide can be used [85]. Vinyl sulfides **38a** and phenyl vinyl selenide (**38b**) were obtained in up to 93% yields (Scheme 17), although in a number of cases the yields are diminished by competitive formation of *bis*-chalcogenide derivatives **39**.

Thus, reactions of thiols and selenols (or dichalcogenides) with acetylene (or calcium carbide as its source) provide convenient access to the wide range of vinyl chalcogenides. Availability of both acetylene and calcium carbide associates with good prospects of development in this direction.

#### 2.1.3. Synthesis of N-Vinyl Derivatives

N-Vinylated compounds are versatile building blocks for polymerization. The nitrogen-containing polymers showed exceptional electroluminescent properties and photoconductivity and widely used in the pill coating or excipient formulations [21,22,86,87,88,89]. The synthesis of N-vinyl derivatives is a demanded goal of modern chemistry.

**Scheme 18:**
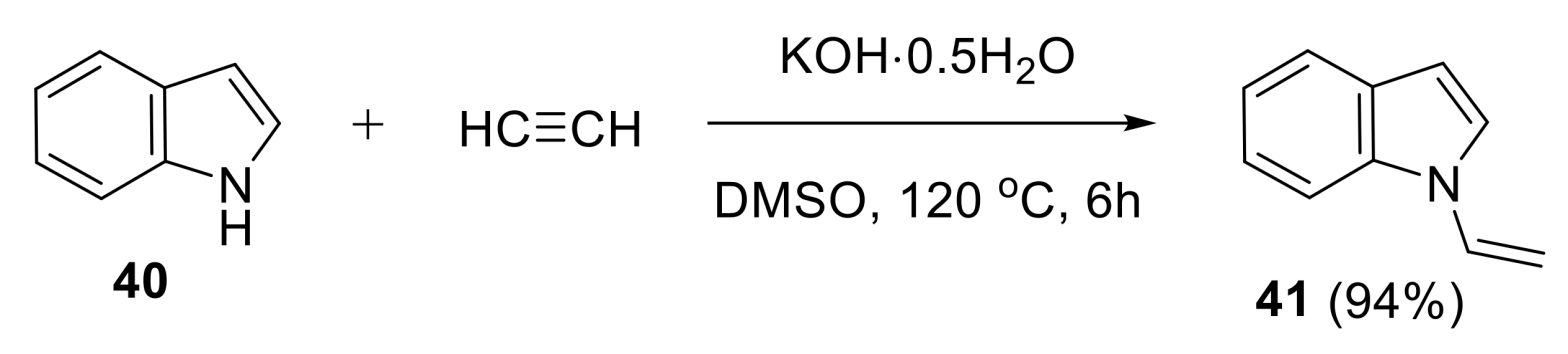
*N*-Vinyl indole synthesis.

A simplest synthetic route to N-vinyl derivatives is provided by addition of nitrogen compounds to acetylene. For example, reaction of indole (**40**) with acetylene in the presence of potassium hydroxide gives *N*-vinylindole (**41**) in 94% yield (Scheme 18) [90]. This reaction illustrates common route to vinylated nitrogen derivatives due to the high availability of acetylene and the wide scope of potentially suitable nitrogen substrates [20]. 

**Scheme 19:**
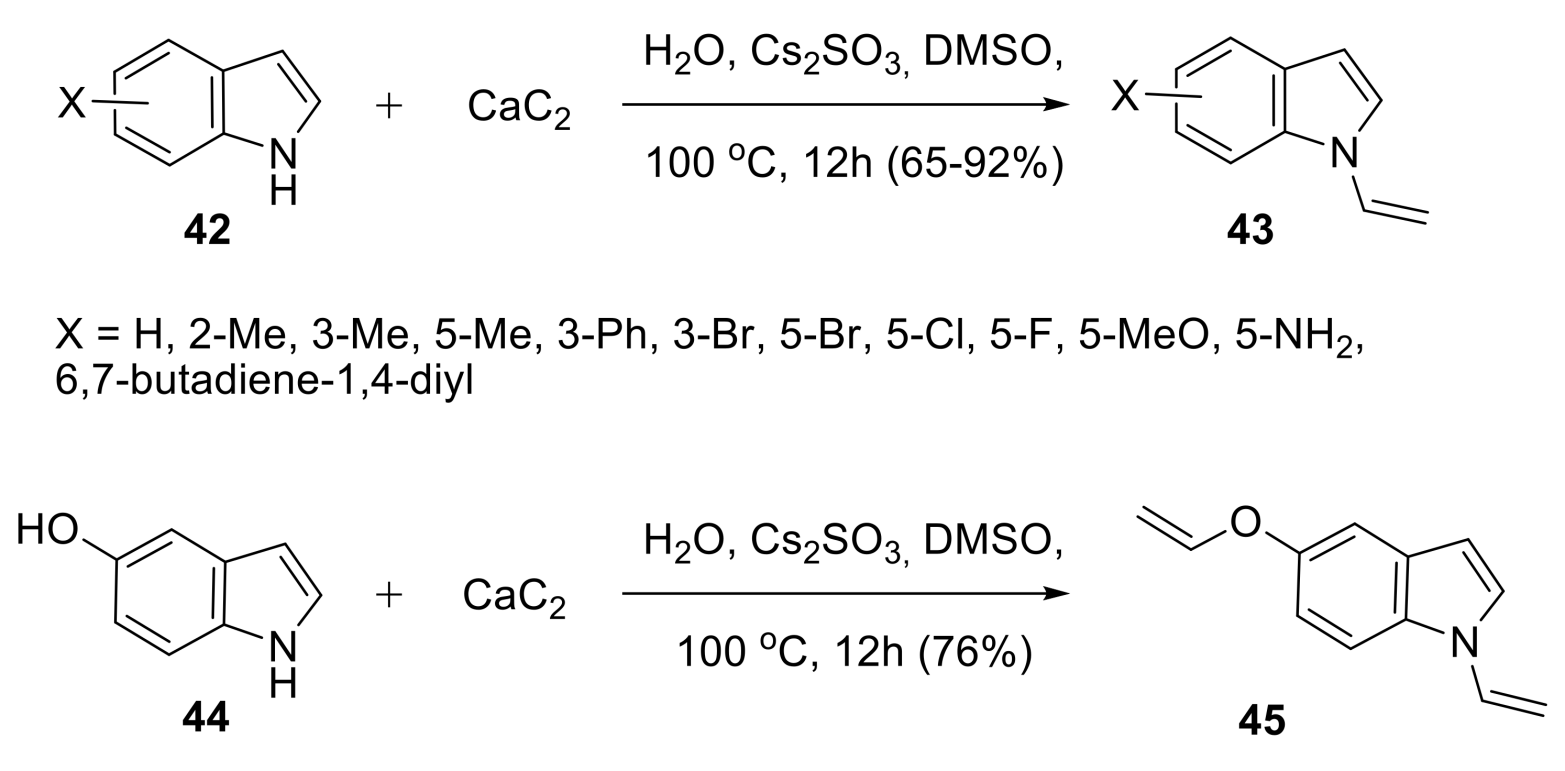
Vinylation of indoles with carbide-induced acetylene.

Replacement of gaseous acetylene with solid calcium carbide, proposed in several recent publications [68,91], seems reasonable. Calcium carbide facilitated obtaining of N-vinylindoles **43** from the corresponding substituted indoles **42** in good to excellent yields (Scheme 19) [68]. The reaction was performed in a Cs_2_CO_3_-DMSO mixture, similarly with the above-mentioned protocol for alcohol vinylation. In one case, reaction of 1*H*-indol-5-ol (**44**) with CaC_2_ reproducibly resulted in formation of a divinyl derivative **45**.

**Scheme 20:**
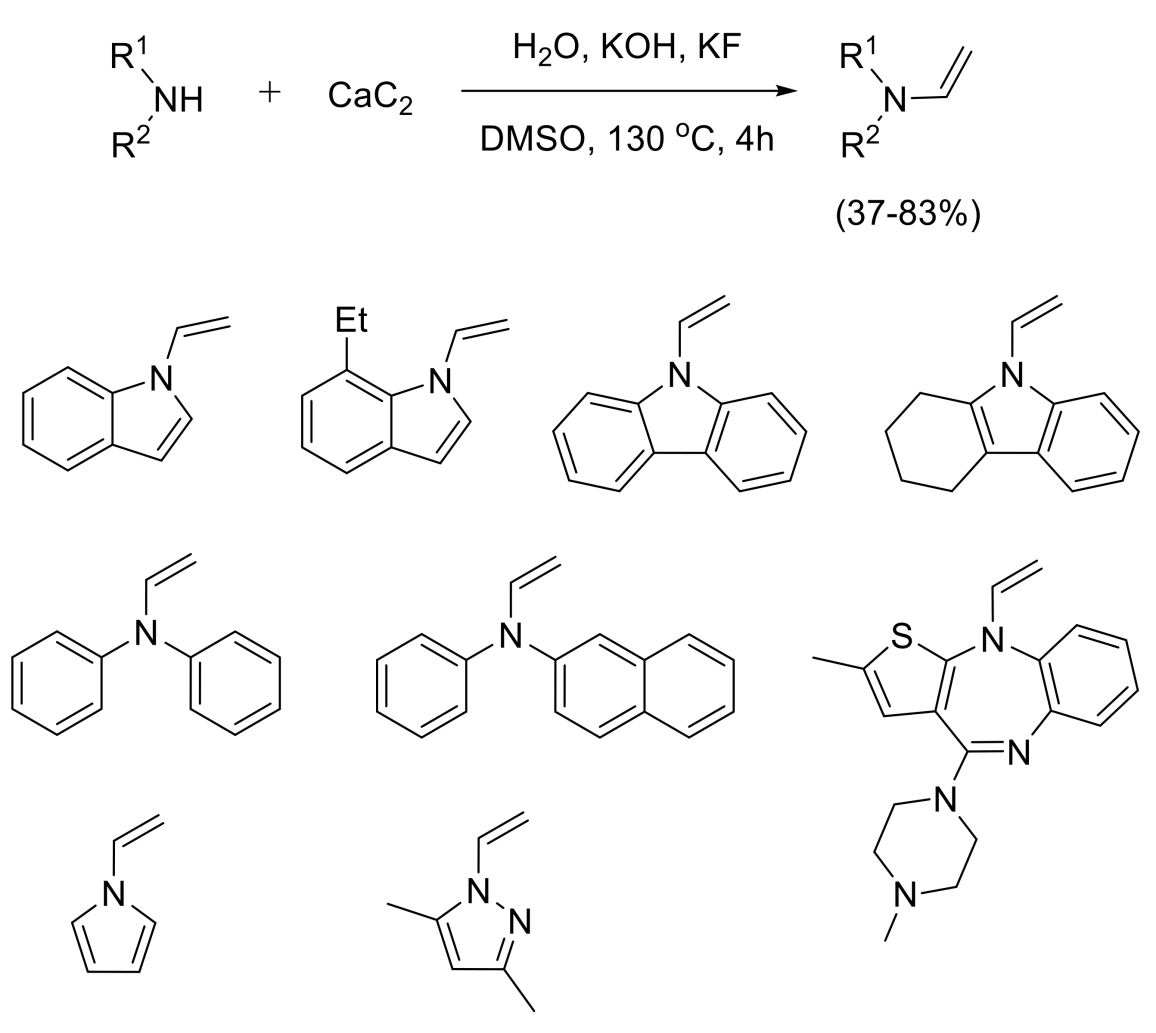
Representative scope of amine core vinylation with CaC_2_.

A new protocol was developed for the carbide-mediated nitrogen core vinylation in superbasic KOH-KF-DMSO system (Scheme 20) [91]. Its application to various indoles, carbazoles, diaryl amines, pyrroles, pyrazoles, and other nitrogen substrates results in moderate to good yields of the target products, additionally increased by the presence of fluoride.

Thus, both acetylene and calcium carbide effectively promote the synthesis of vinyl indoles. The reaction has a wide substrate scope, as it also works for diarylamines, indoles, carbazoles, and other nitrogen heterocycles.

#### 2.1.4. Synthesis of C-Vinyl Derivatives

Vinylation of carbon atoms with acetylene affords a number of important polymers, e.g., polystyrenes for manufacturing of plastic tableware, packaging, building blocks and accessories (insulation, tapes, plugs, cases), and other useful items [92]. 

**Scheme 21:**
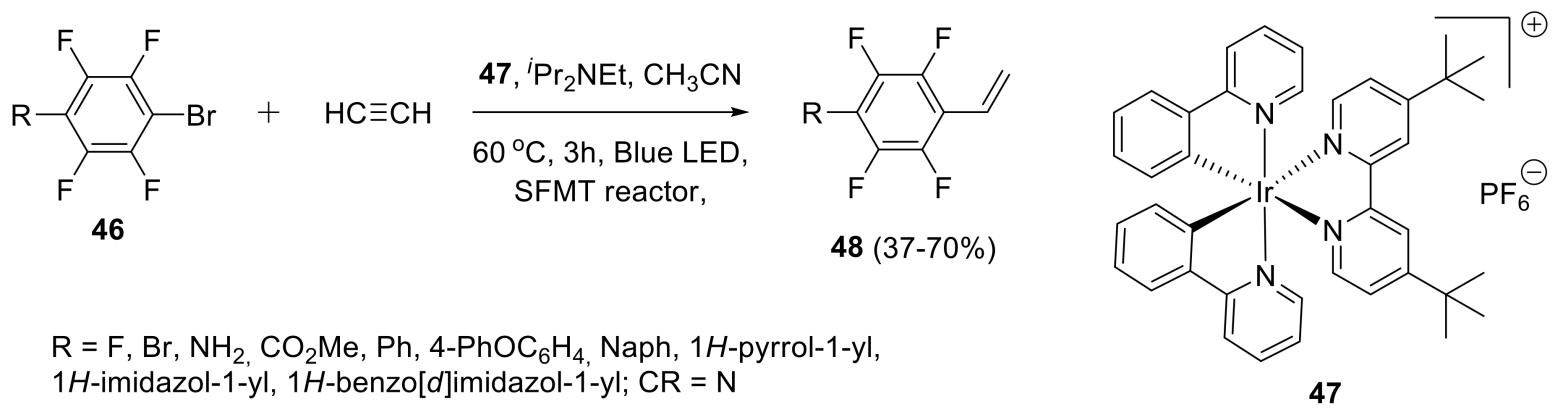
Styrenes synthesis with acetylene.

Acetylene gas was successfully used in the synthesis of fluorinated styrene compounds. The transition metal-catalyzed reaction was accelerated by a stop-flow micro-tubing reactor developed by the authors [93]. Within this device, fluorinated aryl bromides **46** reacted with acetylene in the presence of iridium photocatalyst **47** and diisipropyl ethylamine giving the corresponding vinyl derivatives **48** in 37–70% yields (Scheme 21). Application of this approach is compromised by formation of side products.

**Scheme 22:**
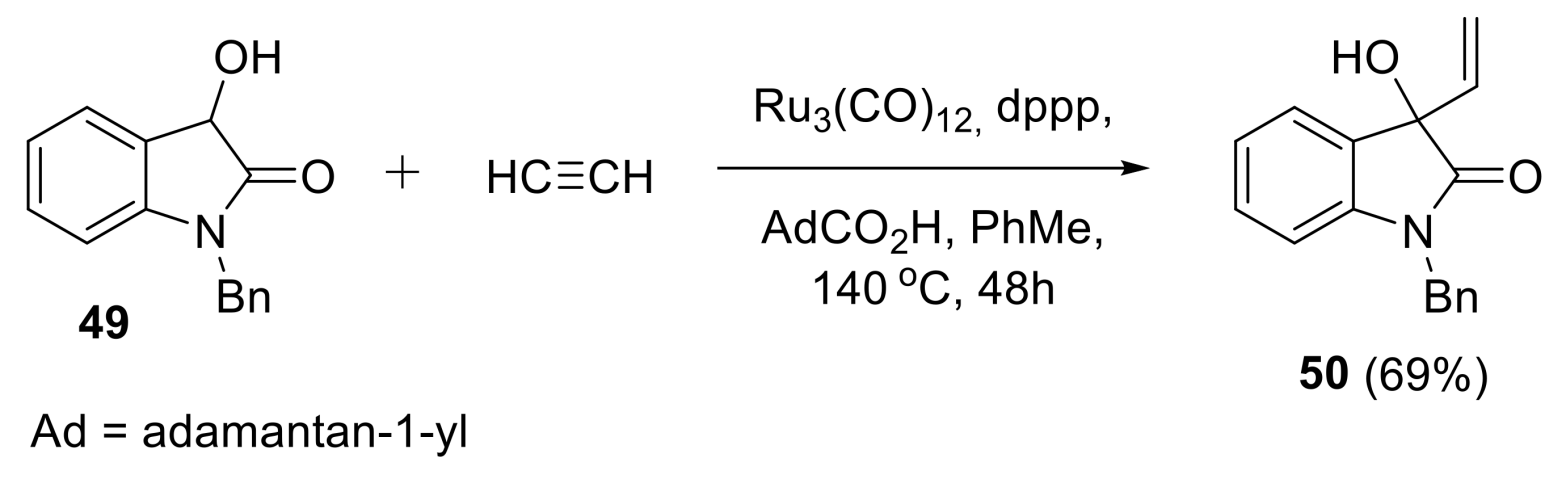
Modification of *N*-benzyl-3-hydroxy-2-oxindole with acetylene.

A recently published example of a modification of *N*-benzyl-3-hydroxy-2-oxindole (**49**) with acetylene involves ruthenium carbonyl catalysis [94]. The reaction proceeds in the presence of 1,3-*bis*(diphenylphosphino)propane and adamantanecarboxylic acid and allows obtaining vinyl derivative **50** in 69% yield (Scheme 22). This product is a potential spirooxindole precursor with specific arrangement of functional groups in its structure [94]. 

The examples of C-vinylation are of certain theoretical importance and immense practical relevance. Further progress in discovering their potential is expected in the near future.

### 2.2. Synthesis of Acrylic Acid Derivatives

**Scheme 23:**
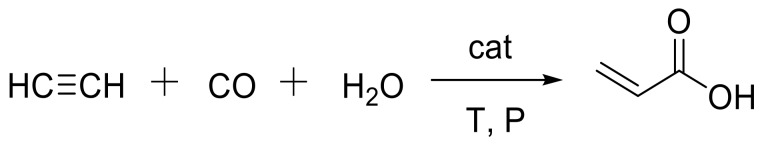
Catalytic carbonylation of acetylene.

Acrylic acid (AA) and its derivatives are important components of plastics, rubbers, superabsorbent polymers, and paints. In industry, AA is prepared by partial oxidation of propene [95], which is refined from petroleum. Carbonylation of acetylene, introduced by Reppe [96], represents an alternative route to AA, which is better in terms of atom economy and independence from oil sources (Scheme 23). 

Reppe used the nickel carbonyl as a catalyst to yield AA under 313.15 K and 0.1 MPa. Ni-Catalysts, especially nickel halides, are commonly used as homogeneous catalysts for AA production [14,97]. Ni_2_O_3_ is active as a heterogeneous catalyst in the presence of copper salts [98]. It was experimentally compared with NiO and other common homogeneous catalysts, including NiBr_2_, NiCl_2_, Ni(NO_3_)_2_, and Ni(OAc)_2_. Under fixed conditions (4 MPa, 235 °C, and CuBr_2_ as a promoter), Ni_2_O_3_ showed the best average activity with 764.6 (mol AA) (mol Ni)^−1^ h^−1^. It could be reused at least five times without loss of activity and regenerated through calcination within 400 °C. The other catalysts’ activities were related as Ni(OAc)_2_ > NiCl_2_ ≈ NiBr_2_ > NiO > Ni(NO_3_)_2_ > Ni(OAc)_2_/PPh_3_/ methanesulfonic acid. 

Molecular sieves (zeolites) are one of the most common and versatile heterogeneous catalysts [99]. Zeolite-based Ni catalysts were tested in acetylene carbonylation [97,100]. Comparison of the catalysts prepared by using different supports and nickel introduction procedures (incipient wetness impregnation and aqueous ion exchange) revealed the best catalytic activity of Ni-exchanged Y zeolites in the presence of Cu salt as a promoter (62 g AA/(gcat.∙h)). Multiple reuse of NiY in a batch reactor confirmed its stable activity at 235 °C and 3.6 MPa of initial total pressure. 

An alternative acetylene carboxylation approach is based on using formic acid (FA) instead of toxic CO. FA provides an efficient in situ source of CO for hydrocarboxylation and hydroformylation of olefins [101]. 

**Scheme 24:**
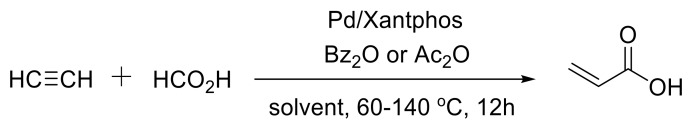
Hydrocarboxylation of acetylene with FA.

Hydrocarboxylation of acetylene with FA in the presence of palladium catalysts and a catalytic amount of benzoic or acetic anhydride is shown in Scheme 24 [28]. 

**Figure 1:**
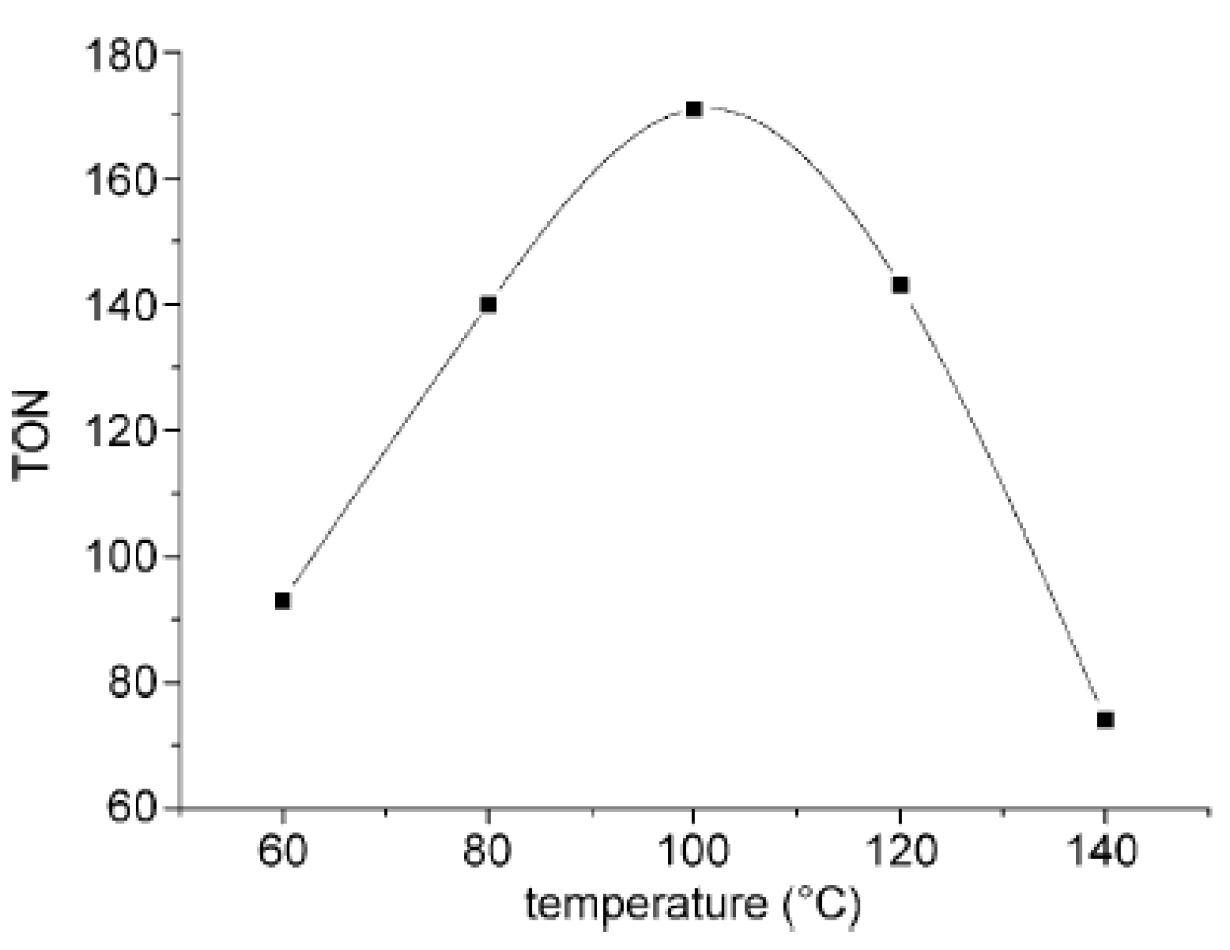
Effect of reaction temperature on acetylene hydrocarboxylation. Reaction conditions: [Pd_2_(dba)_3_]·CHCl_3_ (2.5 mmol), Xantphos (50 mmol), benzoic anhydride (0.1 mmol), HCO_2_H (1.5 mmol), acetylene (initial pressure 10 atm, ca.12 mmol), in THF (3 mL) for 12 h (reproduced with permission from [28]. Copyright 2015, WILEY-VCH Verlag GmbH & Co., KGaA).

**Figure 2:**
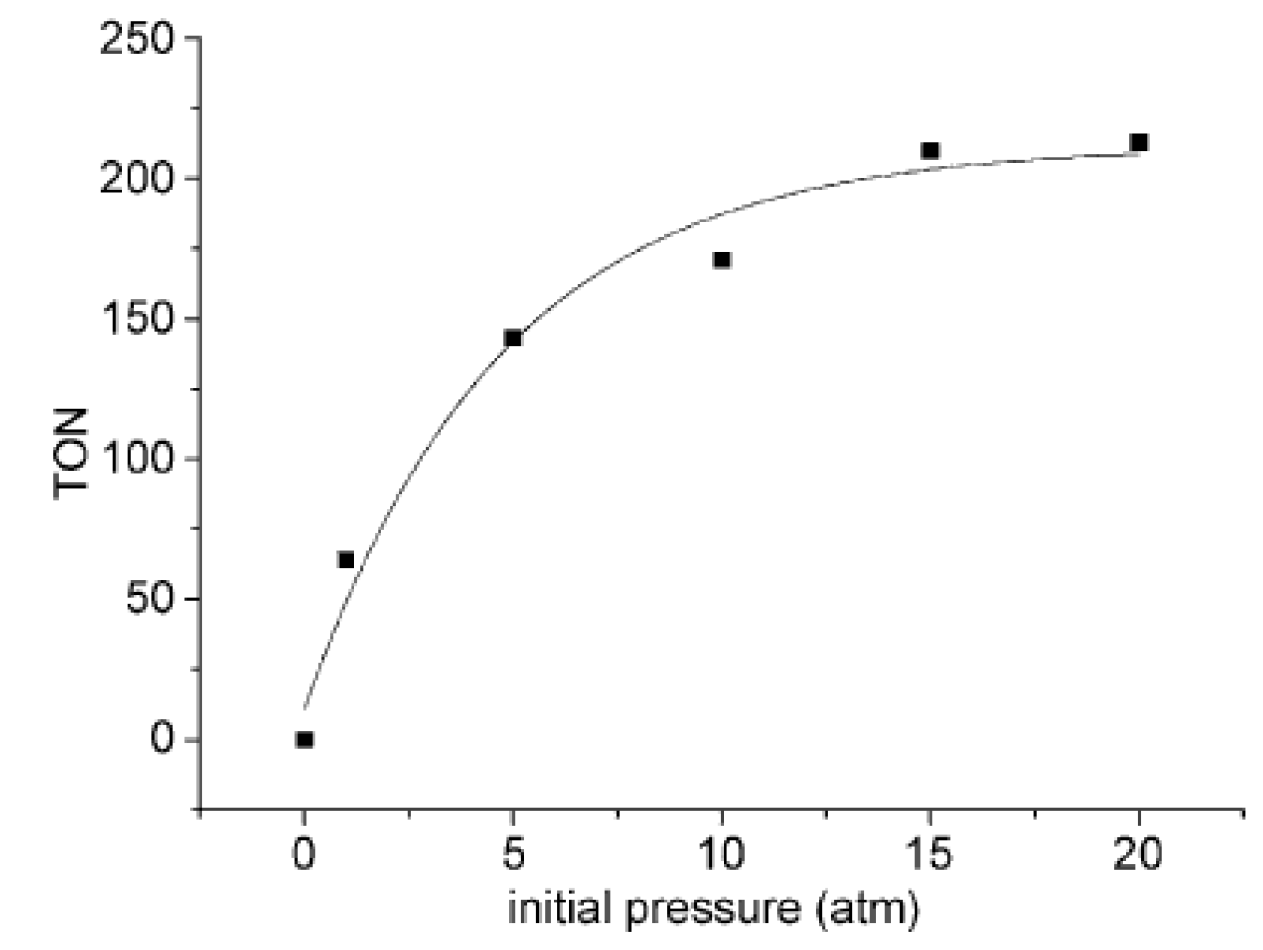
Effect of initial pressure on acetylene hydrocarboxylation. Reaction conditions: [Pd_2_(dba)_3_]·CHCl_3_ (2.5 mmol), Xantphos (50 mmol), benzoic anhydride (0.1 mmol), HCO_2_H (1.5 mmol), in THF (3 mL) at 100 °C for 12 h (reproduced with permission from [28]. Copyright 2015, WILEY-VCH Verlag GmbH & Co. KGaA).

**Figure 3:**
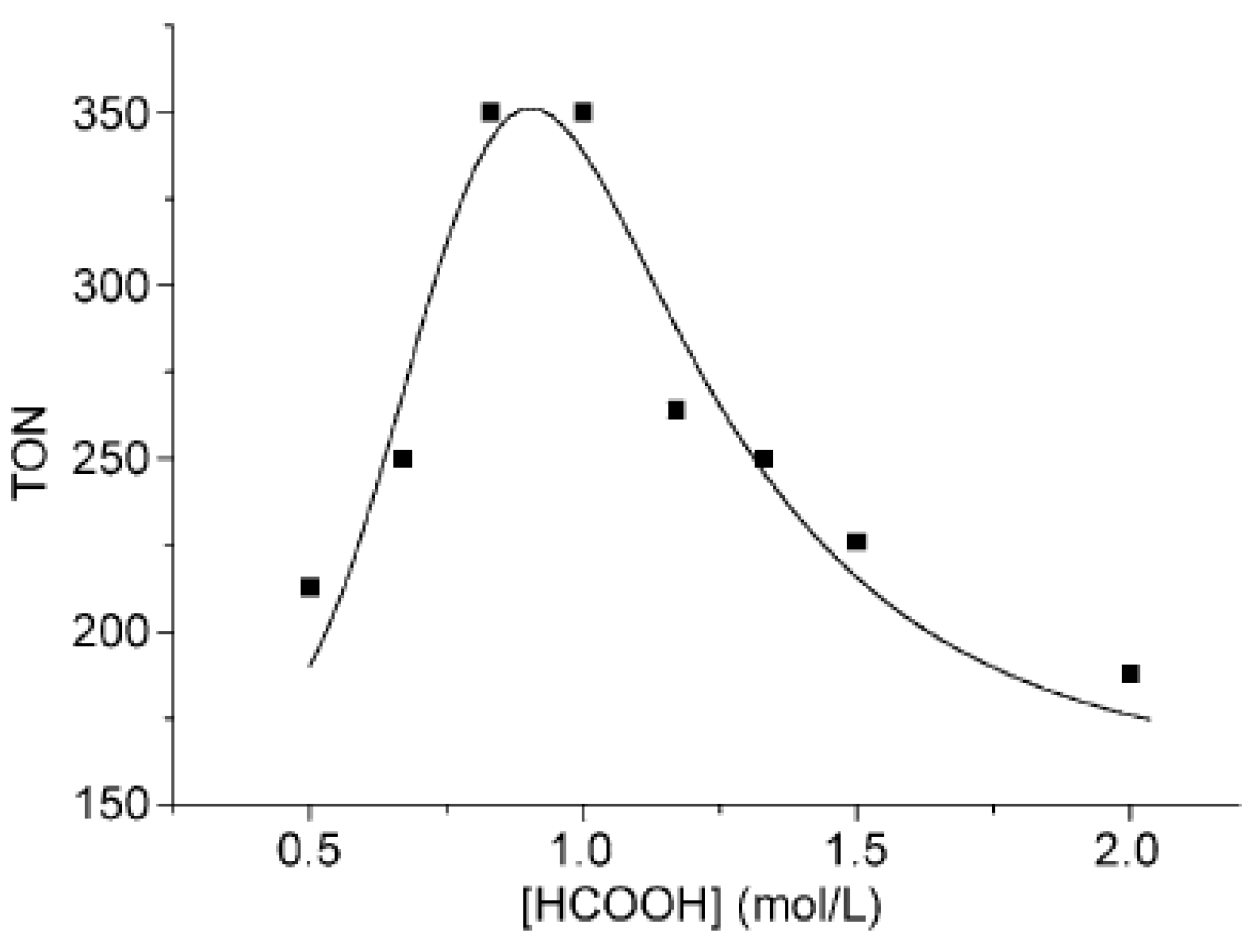
Effect of FA formic acid concentration on acetylene hydrocarboxylation. Reaction conditions: [Pd_2_(dba)_3_]·CHCl_3_ (2.5 mmol), Xantphos (50 mmol), benzoic anhydride (0.1 mmol), acetylene (initial pressure 15 atm, ca. 18 mmol), in THF (3 mL) at 100 °C for 12 h (reproduced with permission from [28]. Copyright 2015 WILEY-VCH Verlag GmbH & Co. KGaA).

**Figure 4:**
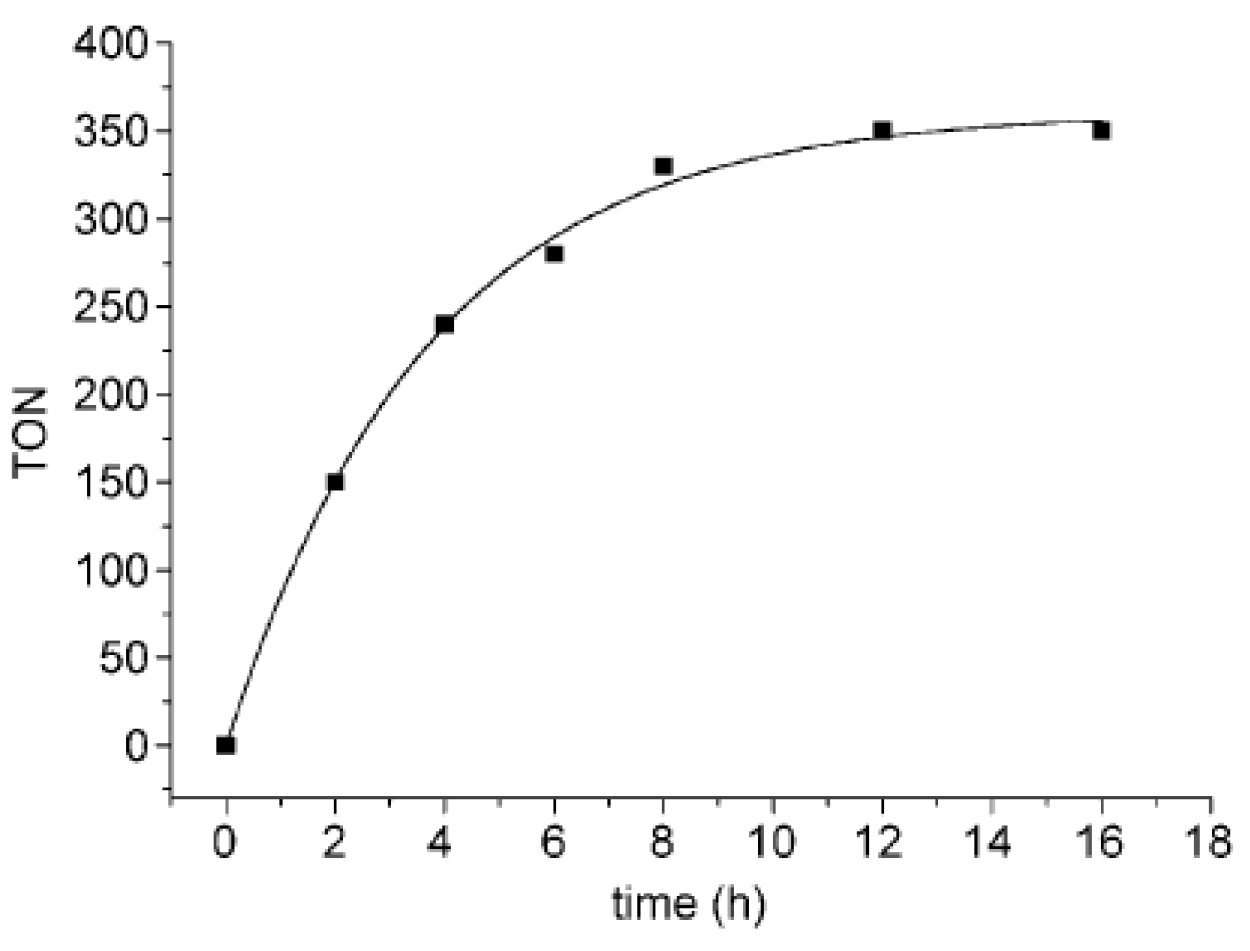
Effect of reaction time on acetylene hydrocarboxylation. Reaction conditions: [Pd_2_(dba)_3_]·CHCl_3_ (2.5 mmol), Xantphos (50 mmol), benzoic anhydride (0.1 mmol), HCO_2_H (3 mmol), acetylene (initial pressure 15 atm, ca.18 mmol), in THF (3 mL) at 100 °C (reproduced with permission from [28]. Copyright 2015 WILEY-VCH Verlag GmbH & Co. KGaA).

Procedure of hydrocarboxylation with FA promotes conversion of acetylene into AA with TONs of up to 350 s^−1^ under mild conditions in the presence of [Pd_2_(dba)_3_]·CHCl_3_. Effects of reaction temperature, acetylene pressure, FA concentration, and reaction time on the outcomes are presented in Figure 1, Figure 2, Figure 3 and Figure 4. 

The same combined catalytic system is suitable for effective hydrocarboxylation of other terminal alkynes. Its application to internal alkynes provides 2,3-substituted derivatives of AA in up to 97% yields.

**Scheme 25:**
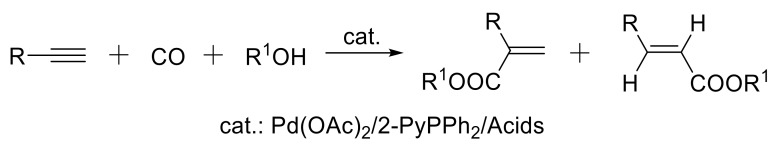
Palladium-catalyzed hydrocarbonylation of terminal alkynes.

Homogeneous catalytic systems composed of 2-pyridyl-diphenylphosphine (2-PyPPh_2_), palladium acetate (Pd(OAc)_2_), and sulfonic acid are highly active and selective in alkyne carbonylation (Scheme 25) [102]. Synthesis of a 2-PyPPh_2_ ligand-based porous organic polymer (POL-2V-P,N) laid the foundation for Pd/POL-2V-P,N catalyst. It is prepared by immobilizing Pd(OAc)_2_ on the POL-2V-P,N support [103]. The heterogenized Pd/POL-2V-P,N catalyst shows higher activity in acetylene methoxycarbonylation than the corresponding Pd-P,N complex under the same conditions.

**Scheme 26:**
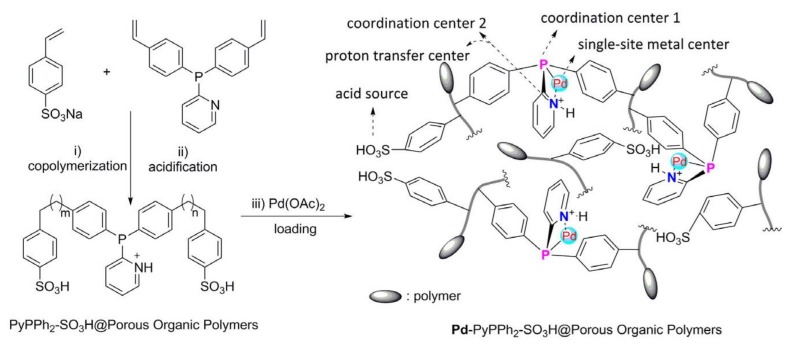
Synthesis of Pd–PyPPh_2_–SO_3_H@POPs catalyst. *Reagents and conditions*: (i) H_2_O, THF, AIBN, 120 °C; (ii) 1M H_2_SO_4_; (iii) Pd(OAc)_2_, THF.

A multifunctional copolymer (PyPPh_2_–SO_3_H@porous organic polymers, POPs) was prepared by combining acidic groups and heterogeneous P, N ligands through the copolymerization of vinyl-functionalized 2-pyridyldiphenylphosphine (2-PyPPh_2_) and *p*-styrene sulfonic acid (Scheme 26) [104]. 

In comparison with traditional homogeneous Pd(OAc)_2_/_2_-PyPPh_2_/ *P*-toluenesulfonic acid catalysts, the copolymer supported palladium catalyst (Pd–PyPPh_2_–SO_3_H@POPs) exhibits higher activity in alkoxycarbonylation of terminal alkynes under the same conditions. The advantage is attributed to synergistic action of the single-site Pd centers, 2-PyPPh_2_ ligands, and SO_3_H groups, as well as the swelling properties and high enrichment of the reactant concentrations by the porous catalyst.

### 2.3. Preparation of Substituted Alkynes 

#### 2.3.1. Cross-Coupling Reactions

**Scheme 27:**
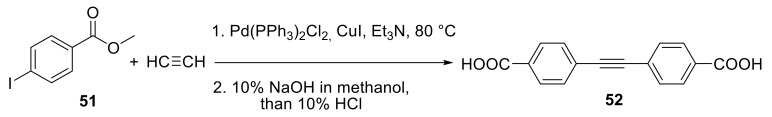
Palladium-catalyzed Sonogashira coupling of acetylene with methyl 4-iodobenzoate.

Sonogashira reaction was discovered in 1975 [105] and represents a fascinating route to various alkynes. Sonogashira cross coupling of acetylene with iodo- and bromoarenes can be successfully applied to obtain symmetric diaryl ethynes [42,106,107,108]. For example, palladium-catalyzed Sonogashira coupling of acetylene with methyl 4-iodobenzoate (**51**) gives 4,4′-(1,2-ethynediyl)-bisbenzoic acid (**52**), a comonomer for the synthesis of liquid crystalline polymer (Scheme 27) [107]. 

**Scheme 28:**
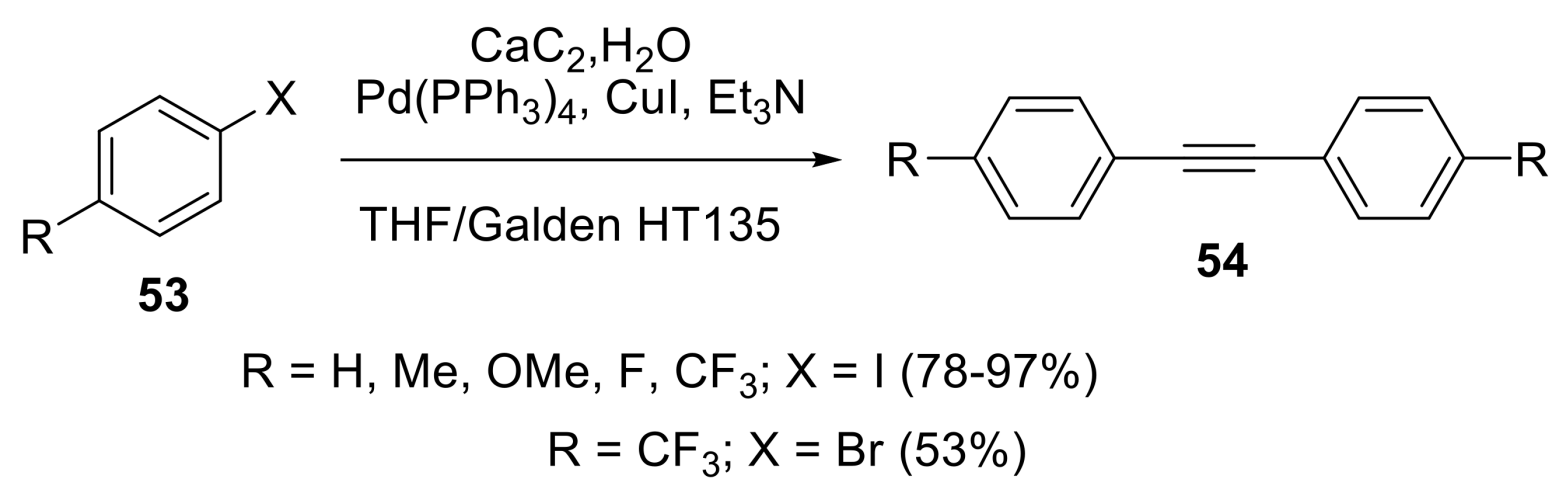
Cross-coupling with CaC_2_-derived acetylene.

In recent studies [37,42,106], calcium carbide was directly used as an acetylene source in cross-coupling reactions to synthesize symmetric diaryl ethynes. Sonogashira coupling of generated from CaC_2_ and water acetylene with aryl halides **53** gave rise to substituted diphenylacetylenes **54** (Scheme 28) [42]. The reaction was carried out in a multiphase one-pot system with fluorinated solvent Galden HT135. The solvent formed a liquid membrane, which separates CaC_2_ from the water phase and facilitates reagents transport in a controllable manner.

**Scheme 29:**
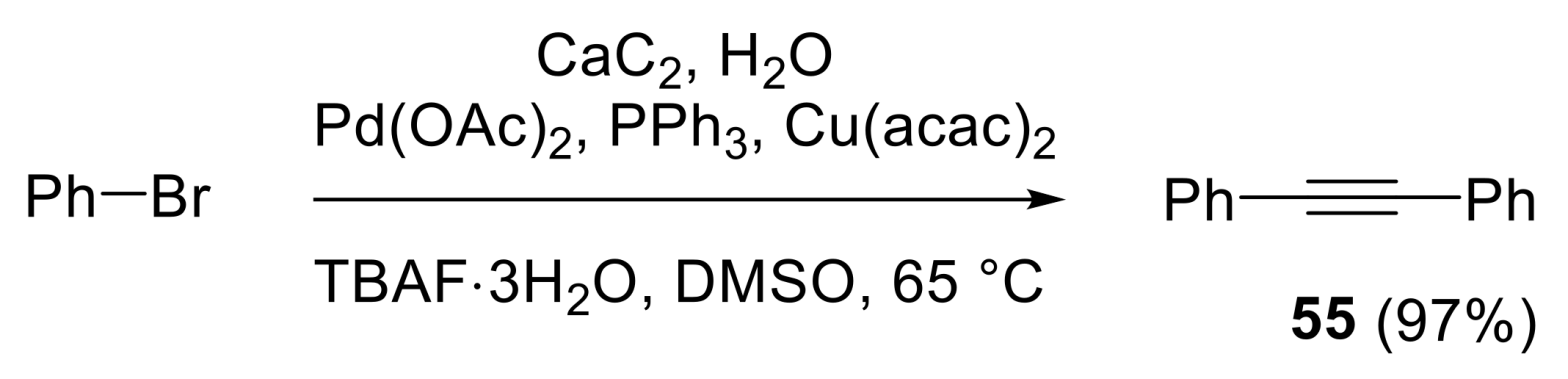
Catalytic synthesis of diphenylacetylene.

Sufficiently good yields were obtained with aryl iodides; the yields for aryl bromides were significantly lower. Using di(4-trifluoromethylphenyl)acetylene resulted in moderate yields of the product (Scheme 28), and 1-bromo-4-methylbenzene showed no reactivity under these conditions [42]. Tetra-*n*-butyl ammonium fluoride (TBAF) effectively promoted Sonogashira cross coupling reactions between the in situ released acetylene and aryl bromides [106]. For example, yield of diphenylacetylene **55** was increased up to 97% by using TBAF as an additive (Scheme 29) [106]. 

**Scheme 30:**
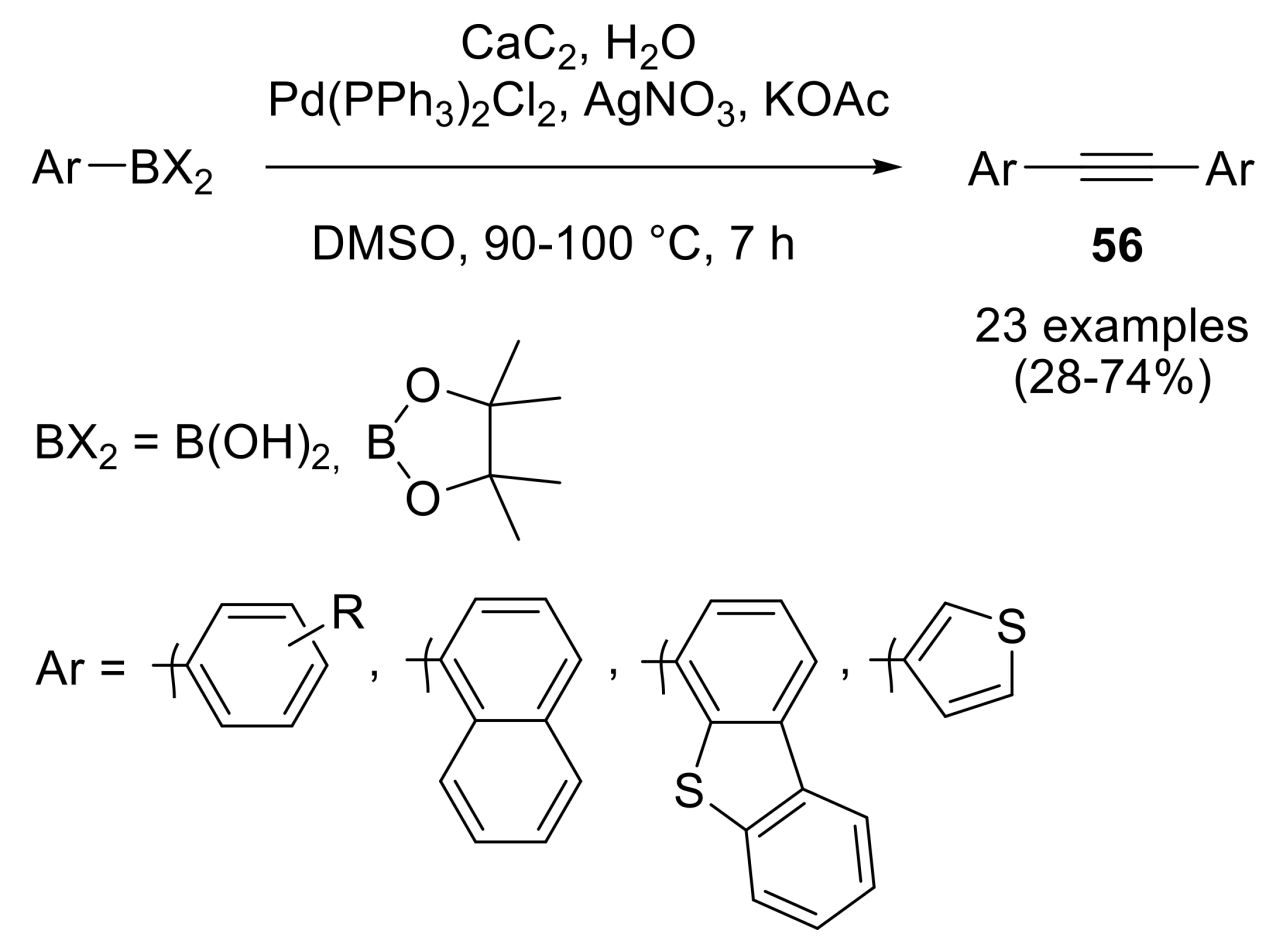
Catalytic synthesis of diarylethynes.

Palladium-catalyzed cross coupling of arylboronic acids or arylboronic pinacol esters with the carbide-derived acetylene led to corresponding diaryl ethynes **56** (Scheme 30) [37]. The synthesis was initiated by simple mixing of the components and afforded the desired products in satisfactory yields.

**Scheme 31:**
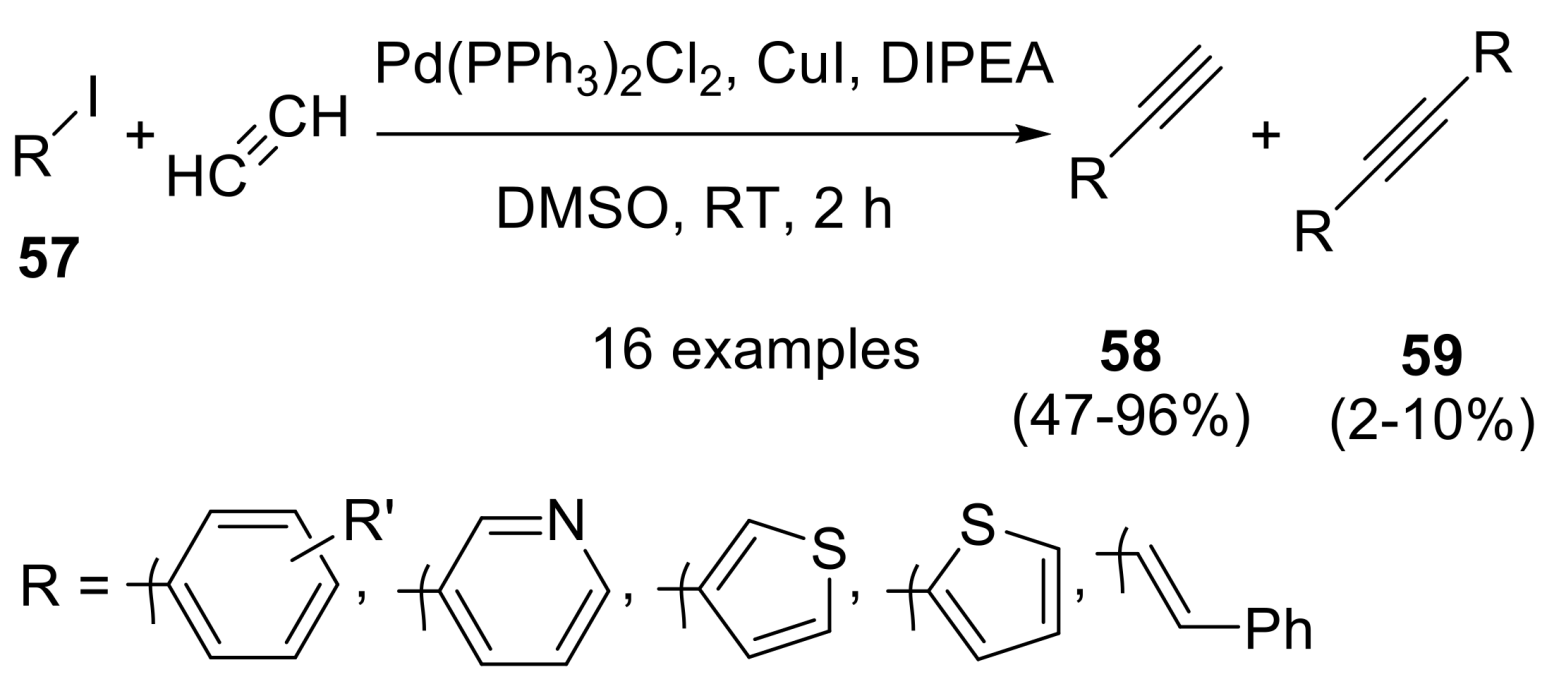
Sonogashira coupling with aryl iodides in a flow reactor.

Catalytic cross coupling with acetylene allows facile production of terminal alkynes, versatile building blocks for drug development and material synthesis. However, constructing terminal alkynes directly from acetylene is problematic due to the low selectivity between terminal and symmetric disubstituted alkynes. This obstacle can be successfully resolved by using flow reactors [93,109]. Significant improvements of the reactivity and selectivity result from the distinguished gas/liquid interfacial contact. Sonogashira coupling between acetylene and aryl iodides **57** in a stop-flow microtubing reactor convincingly affords terminal arylacetylenes **58** as major products (Scheme 31) [93]. 

**Scheme 32:**
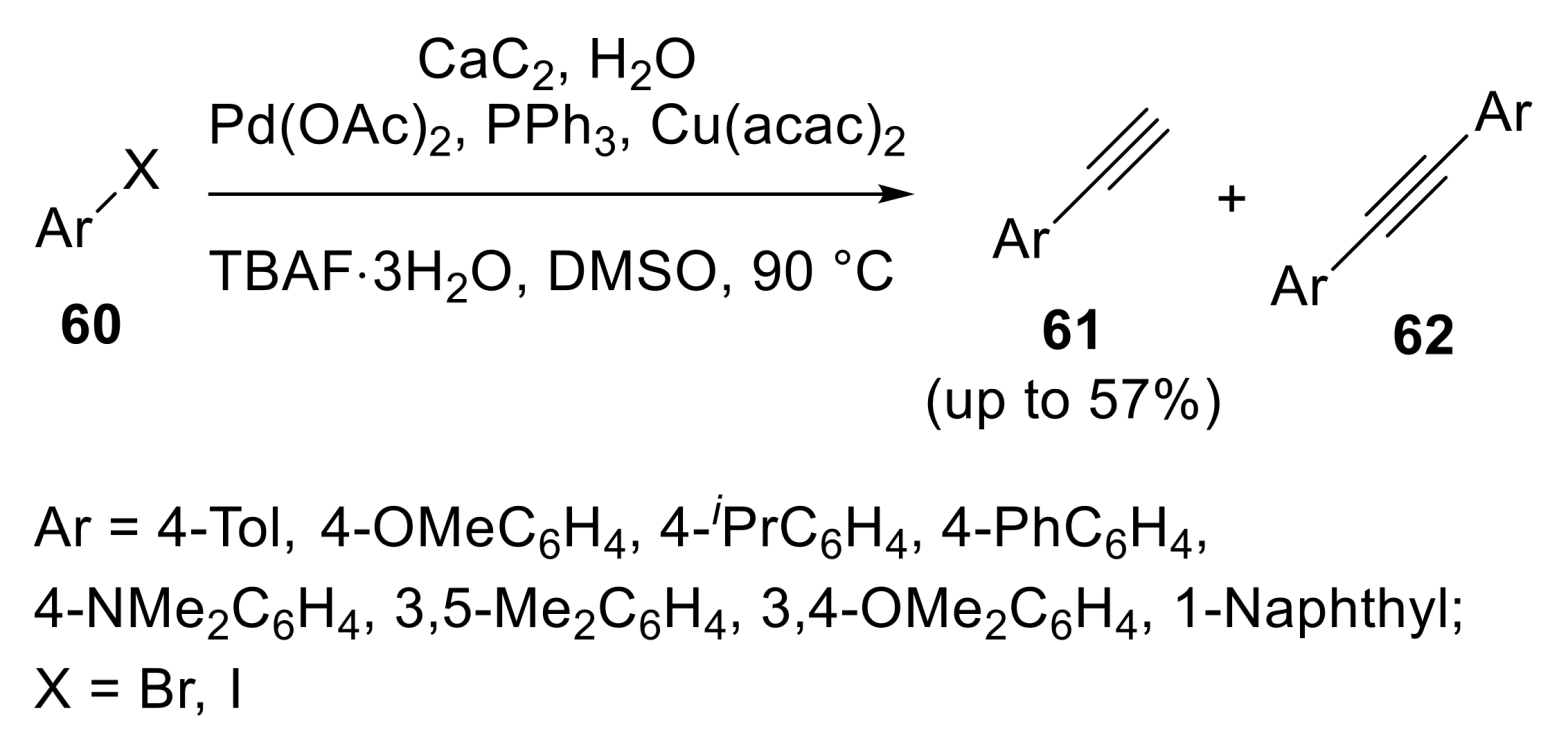
TBAF-promoted Sonogashira coupling of arylhalides with CaC_2_-derived acetylene.

Mono-vs. disubstituted product selectivities of the TBAF-promoted Sonogashira coupling of arylhalides **60** with carbide-derived acetylene under various conditions have been examined in a pilot study [106]. The best monosubstitution selectivity was achieved when extra amounts of TBAF and water were gradually added to the reaction mixture during the reaction. The ratio of calcium carbide and water is also important; the best results were obtained at H_2_O:CaC_2_ molar ratio of 1.3:1. Reactions with 4-methyl-, 4-phenyl-, 4-(dimethylamino)phenyl bromides and 3,5-dimethylphenyliodide gave the corresponding monosubstituted products **61** in 28–57% yields. Using other aryl halides afforded mixtures of mono- and di-coupled products (with **61**:**62** ratio of 1:1 to 4:1) in 57–90% yields (Scheme 32).

**Scheme 33:**
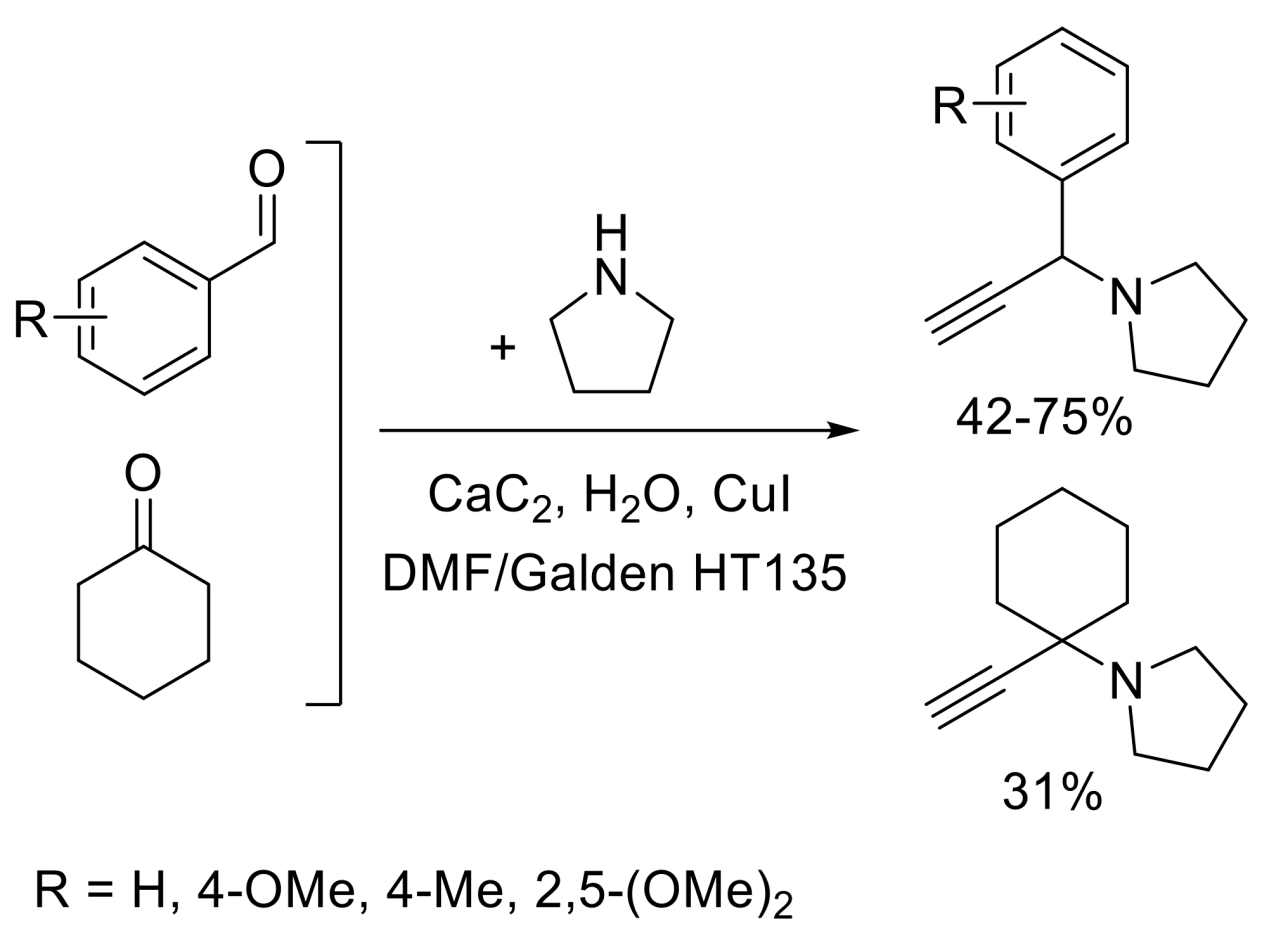
Three-component aldehyde-alkyne-amine coupling.

A three-component aldehyde-alkyne-amine coupling reaction was also possible with calcium carbide as an acetylene source [42]. The reaction has been originally performed with cyclohexanone (instead of aldehydes) in the multiphase one-pot system with fluorous liquid membrane Galden HT135. The system allows obtaining up to 75% yields of the desired products (Scheme 33).

**Scheme 34:**
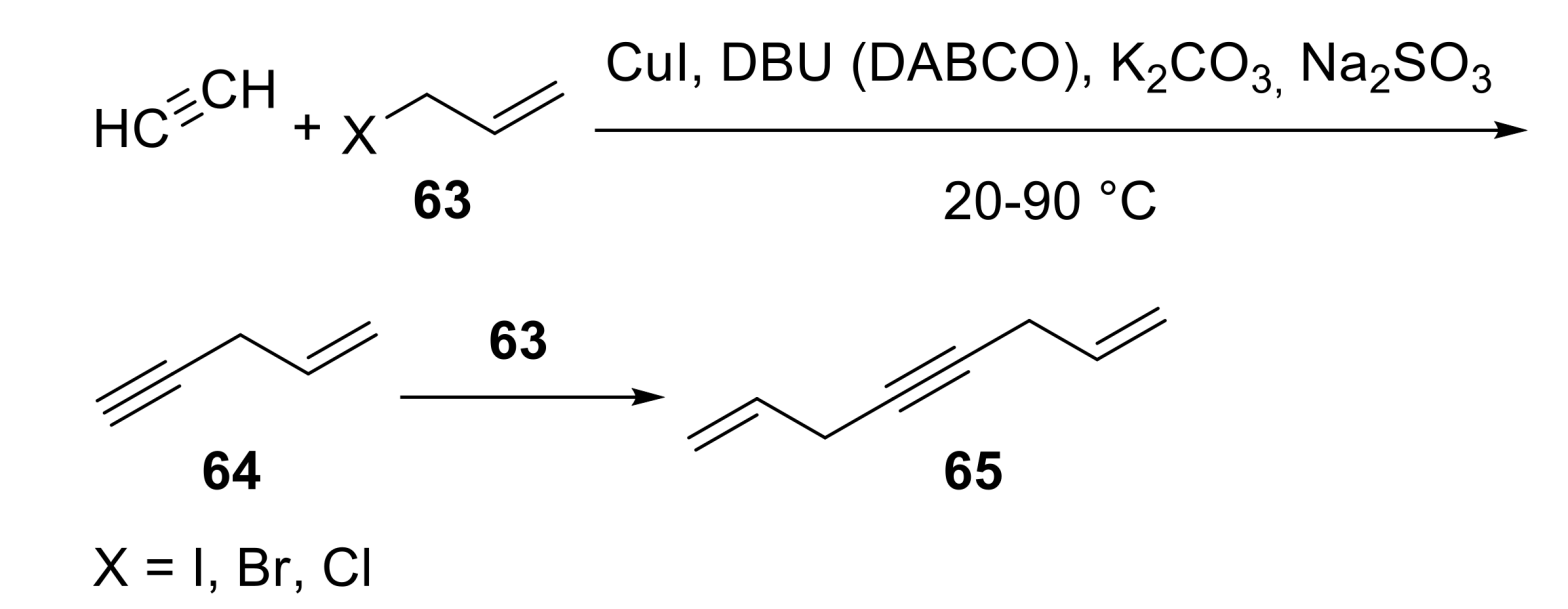
Copper-catalyzed Sonogashira-type allylation.

A copper-catalyzed Sonogashira-type reaction provides the means for allylation of acetylene with allyl halides [110]. Mono-coupled product **64** can be obtained in up to 80% yield by cross-coupling of acetylene with allyl halides **63** under atmospheric pressure in the presence of copper(I) iodide, a base, and a reducing agent. Excess amounts of allyl halide over acetylene lead to predominant formation of disubstituted product **65** in up to 72% yield (Scheme 34).

**Scheme 35:**
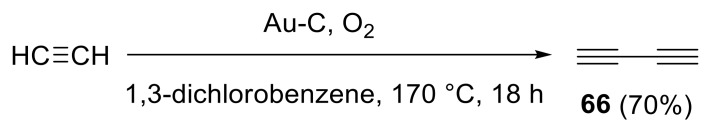
Gold-catalyzed oxidative homocoupling.

A recently reported catalytic system promoted oxidative homocoupling of acetylene under oxygen atmosphere in the presence of carbon-supported gold nanoparticles [111]. The reaction resulted in 70% yield of corresponding dimer **66** (Scheme 35) [111].

#### 2.3.2. Favorskii Reaction

**Scheme 36:**
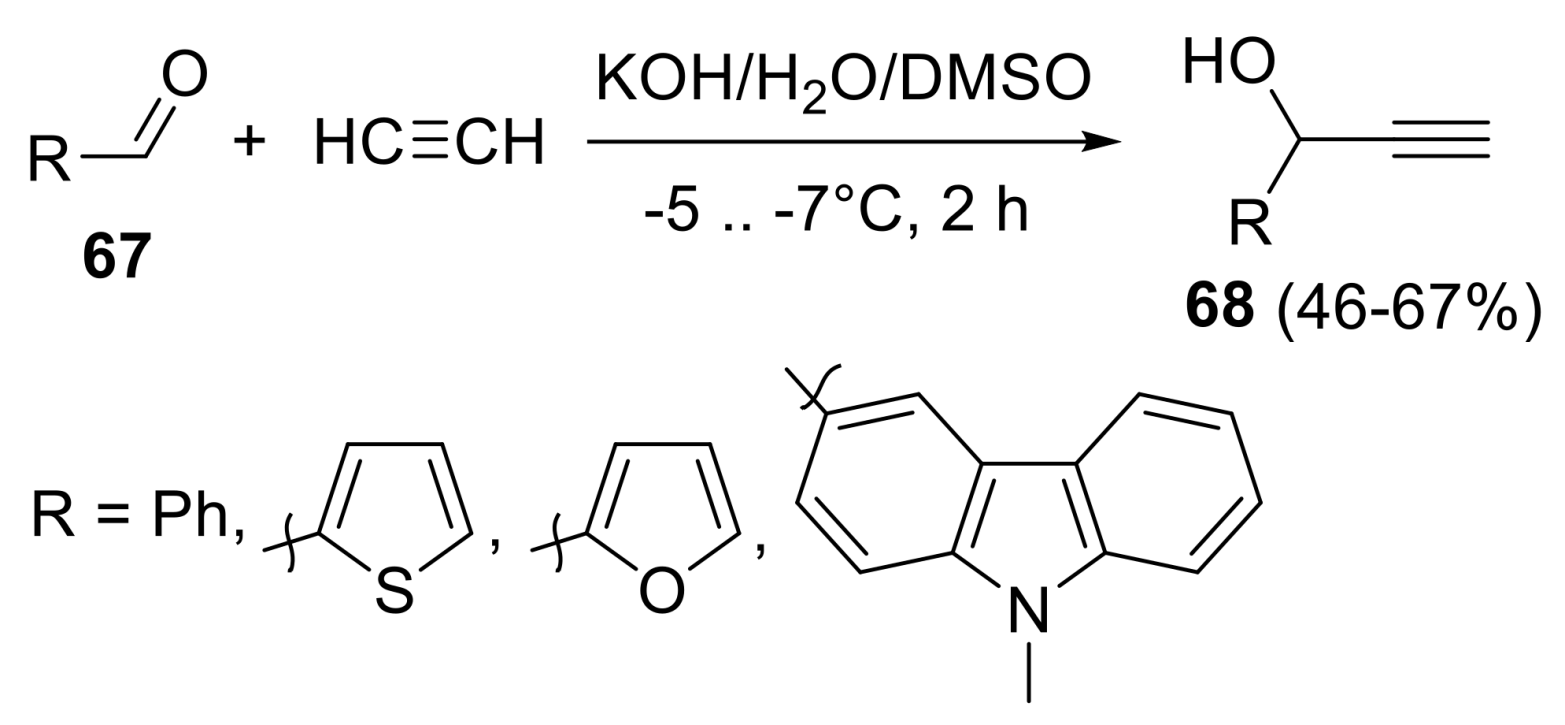
Synthesis of secondary propargyl alcohols from aldehydes.

Introduced in the early 20th century by Favorskii [112] the reaction for obtaining propargyl alcohols by alkynylation of carbonyl compounds is still a heavy hitter [34,113,114,115,116,117,118,119,120]. Convenient and mild synthetic procedures for alkynylation of various aldehydes and ketones have been recently reported by Trofimov et al. [34,113,114]. The catalytic system KOH-H_2_O-DMSO [34,113] was appropriate for the synthesis of secondary propargyl alcohols **68** from aromatic and heteroaromatic aldehydes **67** and acetylene (Scheme 36). The reaction was performed under atmospheric pressure, at constant bubbling of acetylene through a reaction mixture. The target products were obtained in satisfactory yields.

**Scheme 37:**
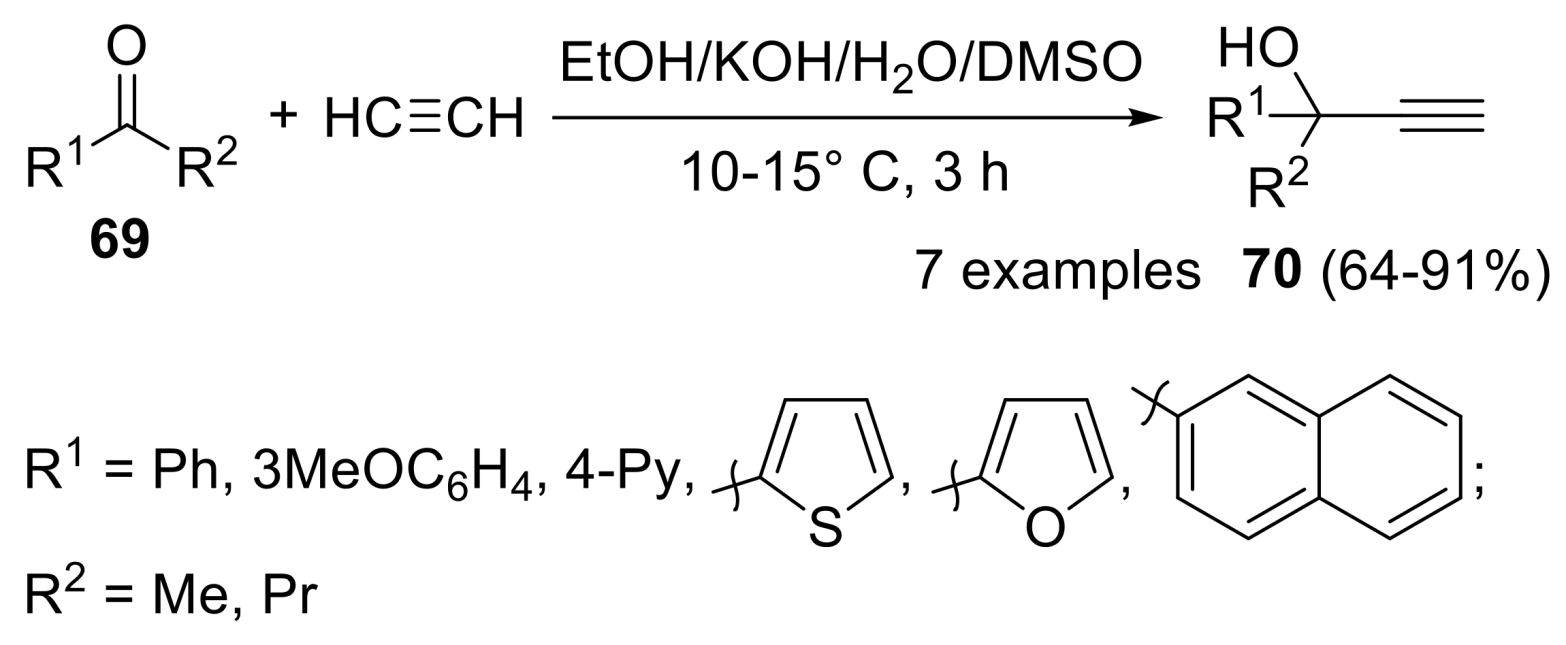
Reaction of alkyl aryl ketones with acetylene.

The procedure was modified by addition of ethanol to the KOH-H_2_O-DMSO system and used for the Favorskii reaction of alkyl aryl ketones **69** with acetylene (Scheme 37) [114]. The desired tertiary propargyl alcohols **70** were obtained in good yields.

**Scheme 38:**
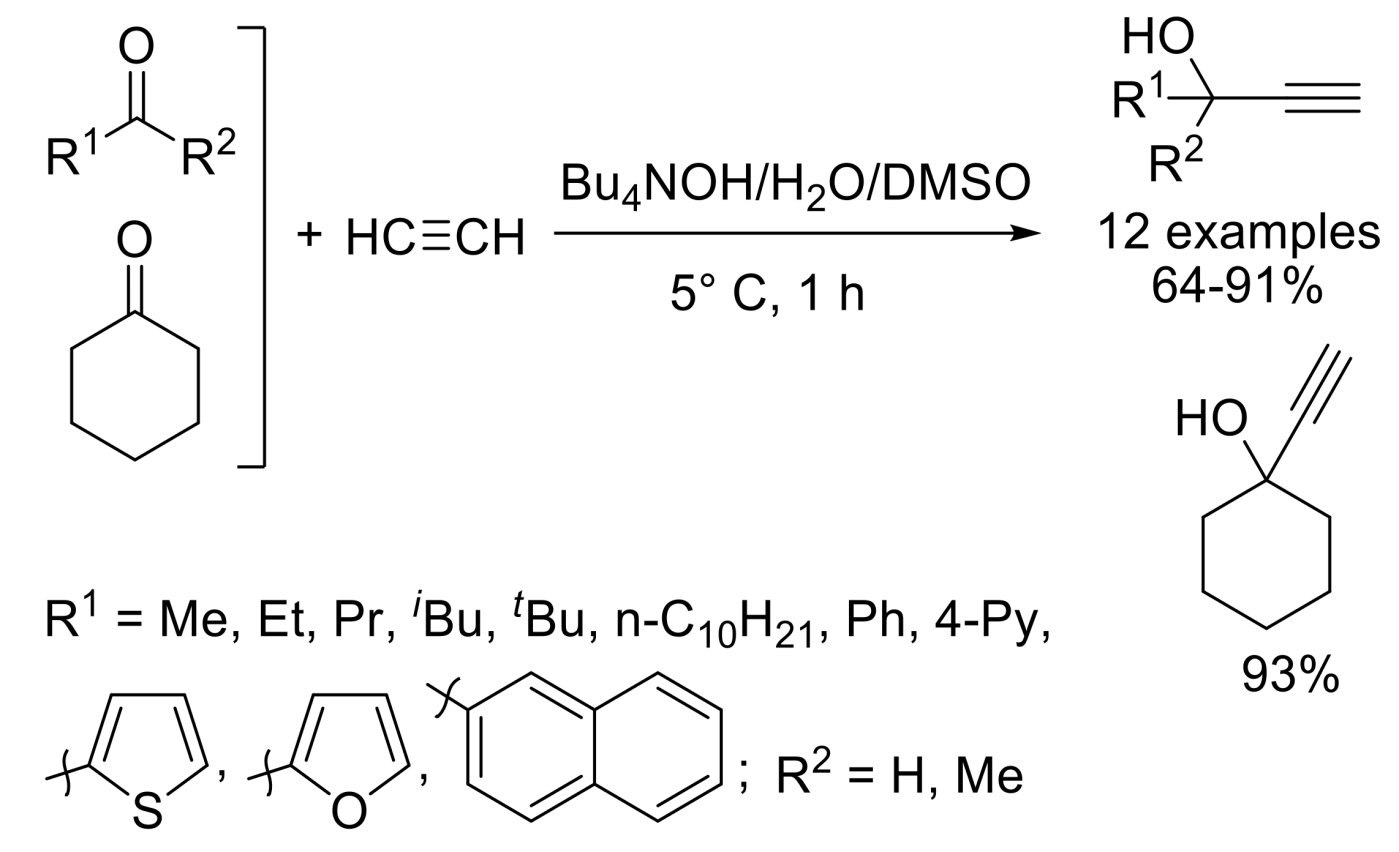
Bu_4_NOH-mediated alkynylation of carbonyl compounds.

The Bu_4_NOH-H_2_O-DMSO [115] catalytic system was suitable for alkynylation of both aromatic and aliphatic aldehydes and ketones. The reaction was carried out in a flow reactor under atmospheric pressure of acetylene. This protocol was efficient for different types of substrates: propargylic alcohols were prepared in high yields from aromatic, heteroaromatic, cyclic and acyclic aliphatic carbonyl compounds (Scheme 38).

**Scheme 39:**
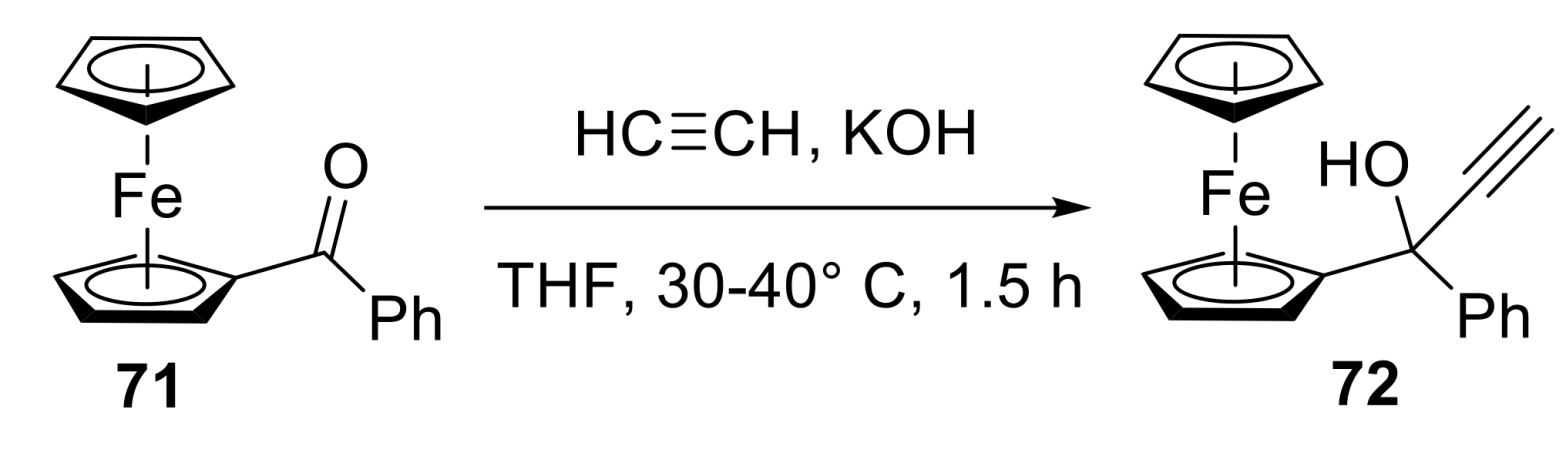
Synthesis of ferrocene-substituted propargyl alcohols.

The synthesis of ferrocene-derived propargylic alcohol demonstrates the versatility of Favorskii reaction. 1-Phenyl-1-ferrocenyl-2-propin-1-ol (**72**) was formed through passing of acetylene into a KOH suspension in THF with benzoylferrocene **71** (Scheme 39) [116]. 

**Scheme 40:**
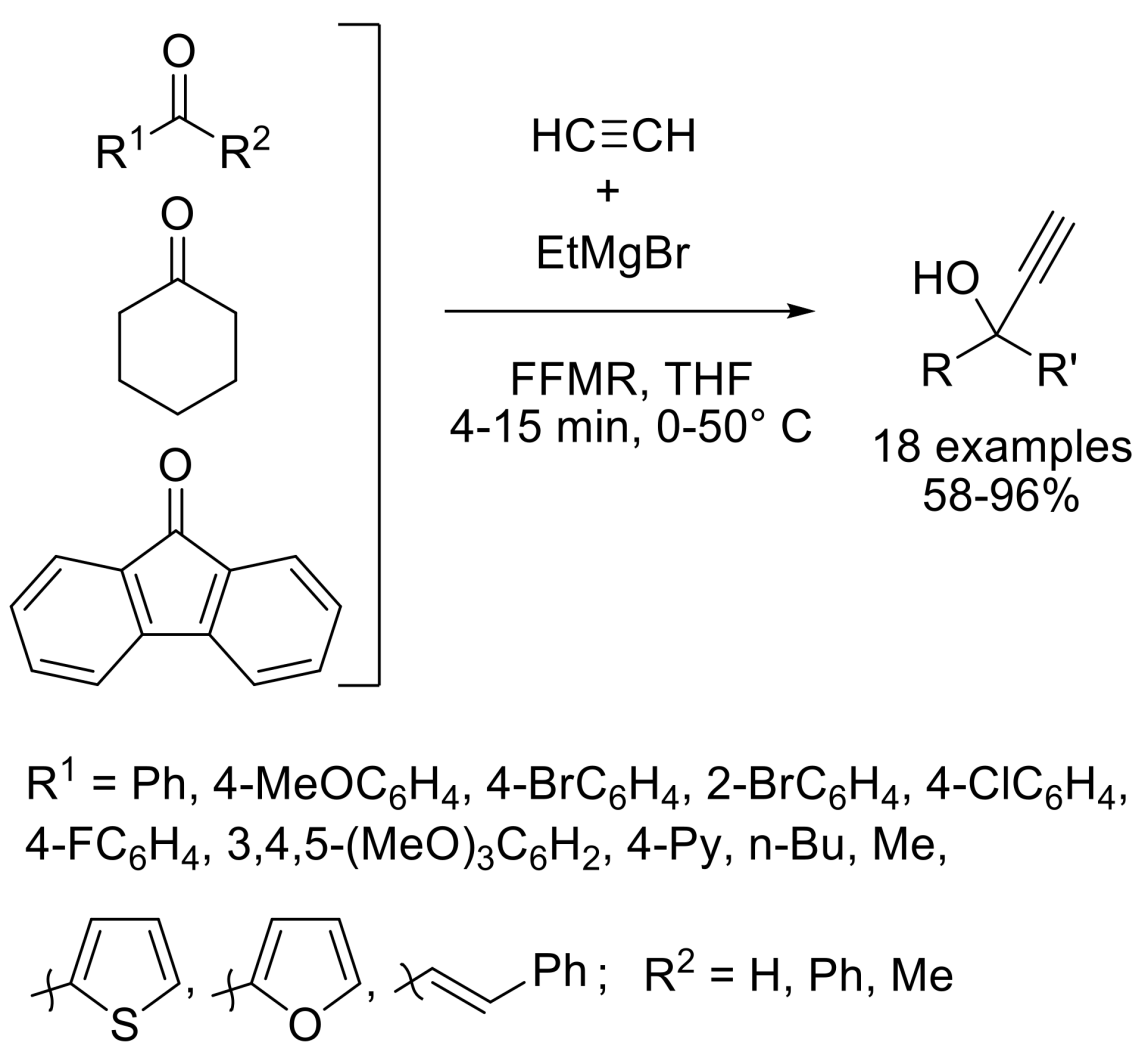
Favorskii alkynylation with in situ generated ethynylmagnesium bromide.

Alkynylation of carbonyl compounds with metal acetylides represents an alternative to the direct base-catalyzed alkynylation with gaseous acetylene [117,118]. Reaction of aldehydes and ketones with in situ generated ethynylmagnesium bromide is carried out in a commercially available falling film microreactor (FFMR) [117]. Consistent gas-liquid contact in the continuous microflow setting is a prerequisite for effective generation of ethynyl-Grignard reagent from EtMgBr and acetylene. The protocol affords a number of propargyl alcohols in high yields and short reaction times, under atmospheric pressure and low reaction temperature (Scheme 40).

**Scheme 41:**
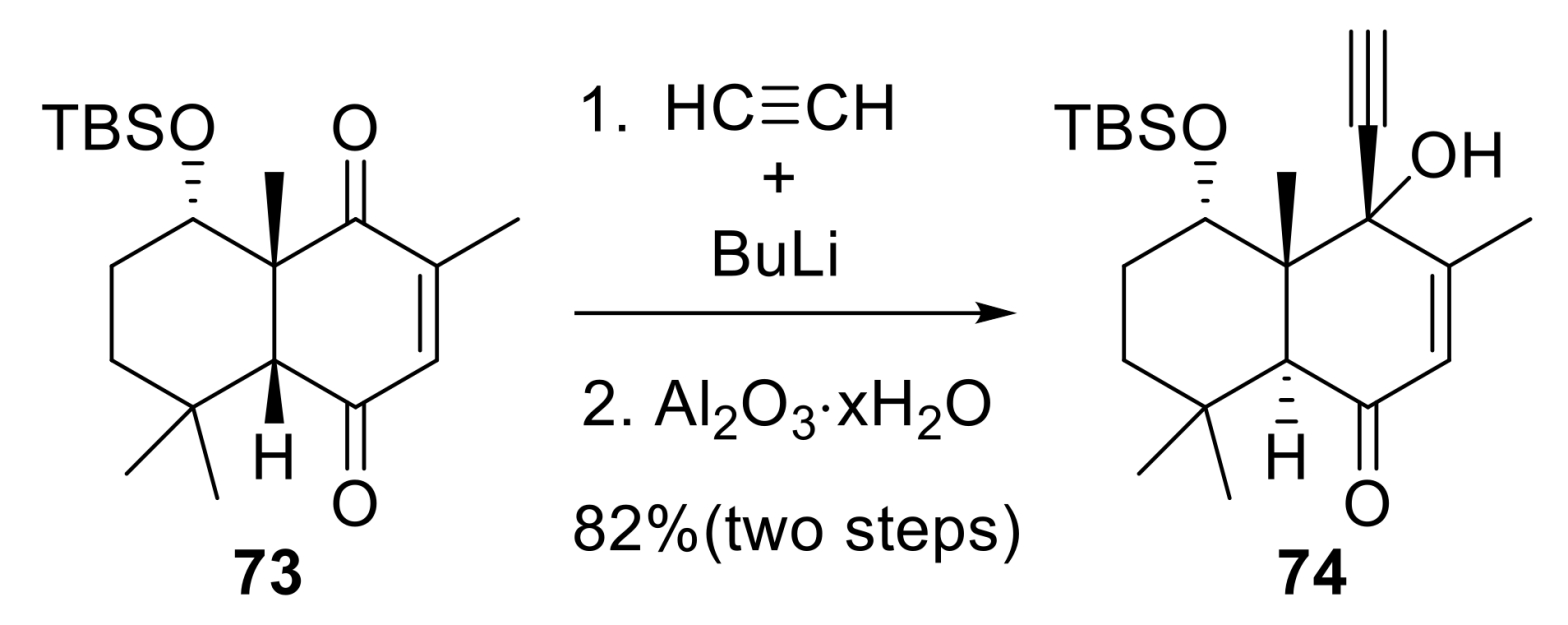
Favorskii alkynylation with in situ generated lithium acetylide.

Lithium acetylide was used for the preparation of decalin-derived acetylenic alcohol **74**, an intermediate for the multistep synthesis of racemic forskolin [118]. Selective addition of monolithium acetylide to one of the two carbonyl groups in *cis*-decalin **73** and subsequent epimerization in the presence of aluminum oxide afforded *trans*-decalin **74** in overall yield 82% (Scheme 41).

**Scheme 42:**
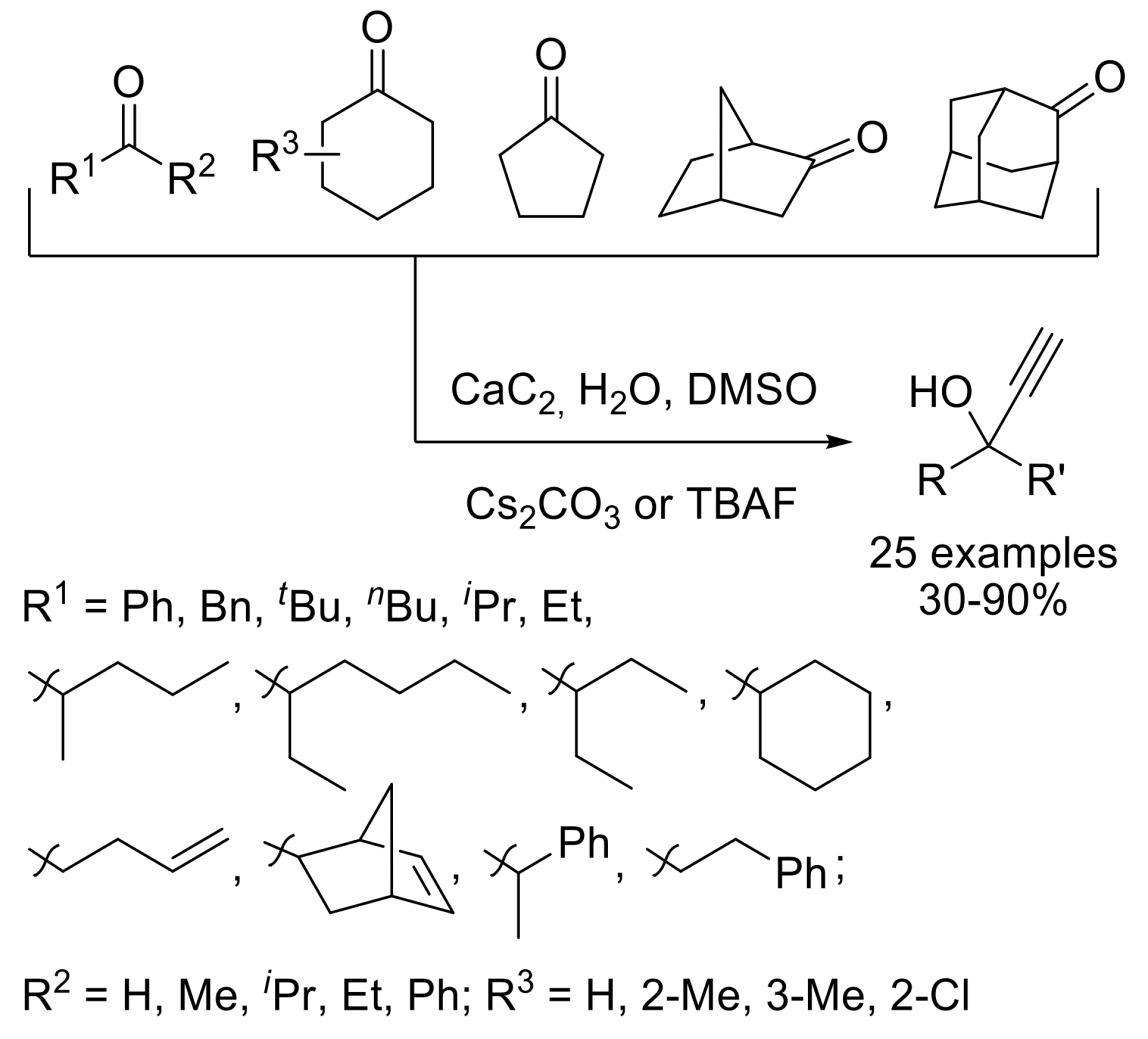
Favorskii reaction with in situ generated acetylene.

Replacement of gaseous acetylene with solid calcium carbide is frequently proposed in recent studies of Favorskii reaction [119,120]. Reactions can be performed for a wide substrate scope including aromatic and aliphatic aldehydes and ketones (Scheme 42). 

TBAF [119] or Cs_2_CO_3_ [120] were used as catalysts, and using of TBAF provided higher yields. Activation of calcium carbide by fluoride is thought to generate an acetylide “ate”-complex that readily adds to carbonyl groups [119]. Both Sonogashira cross coupling and Favorskii alkynylation with acetylene are currently in demand. Mild and environmentally benign protocols for these reactions, including those with the in situ generated acetylene, continuously evolve.

### 2.4. Synthesis of Heterocycles

#### 2.4.1. Cycloaddition Reactions

Priority of pot-, atom-, and step-economy is one of the prevailing concepts in modern applied chemistry [121]. Cycloaddition reactions of acetylene fully conform to these rules, which makes these reactions especially relevant. Cycloaddition usually implies formation of several C-C and C-heteroatom bonds in one synthetic operation. Besides, these reactions afford many useful heterocyclic compounds. In particular, [3 + 2]-cycloaddition reactions of acetylene with active dipoles are increasingly used in synthesis of five-membered heterocycles [41,42,43,44,122,123,124,125,126,127,128]. 

**Scheme 43:**
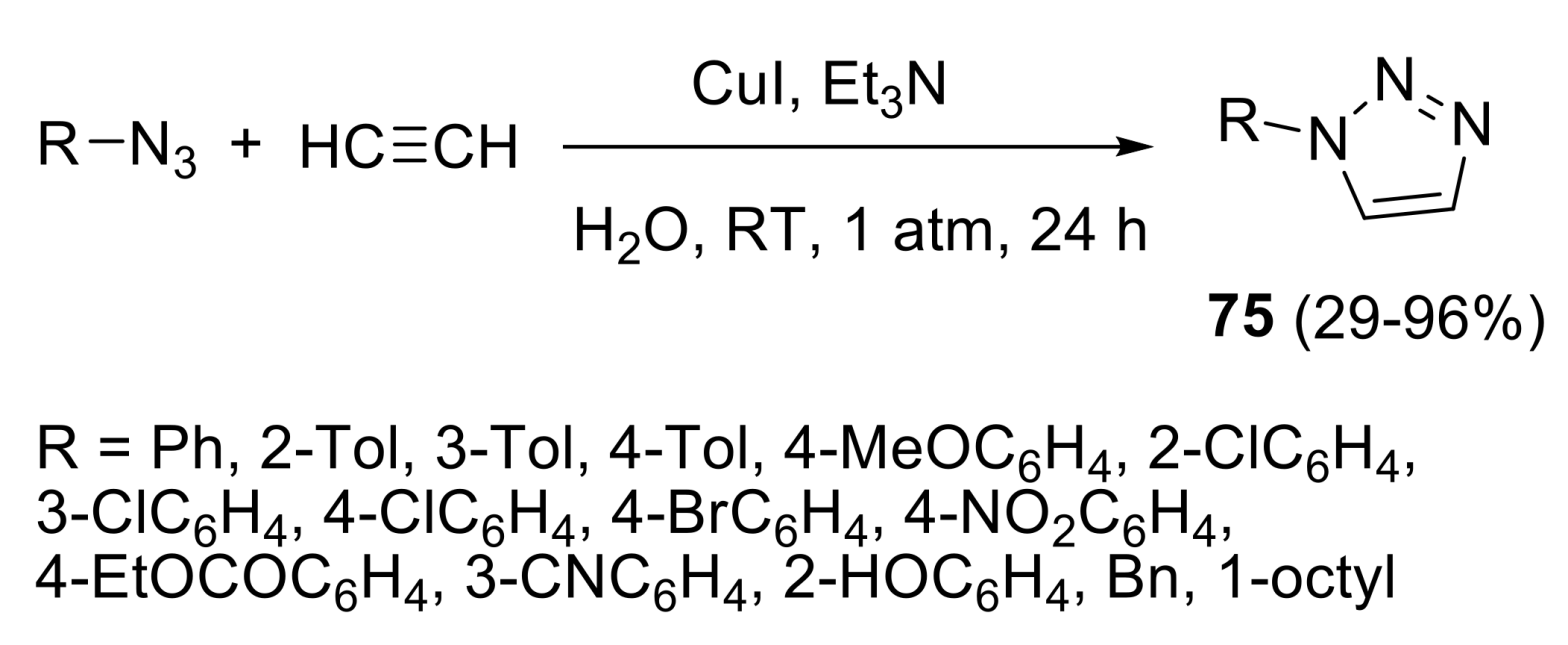
[3 + 2]-Cycloaddition of azides to acetylene, method 1.

1,2,3-Triazoles are produced in the copper-catalyzed click-reactions of organic azides with acetylene [44,122,123,124]. These reactions can be performed in aqueous media under atmospheric pressure [44,122]. A handy procedure for obtaining 1-substituted 1,2,3-triazoles involves CuI-Et_3_N as a catalyst and water as a solvent [44]. Its application to a variety of substrates facilitates transformation of substituted phenyl, benzyl, and octyl azides into corresponding substituted triazoles **75** in up to 96% yield (Scheme 43).

**Scheme 44:**
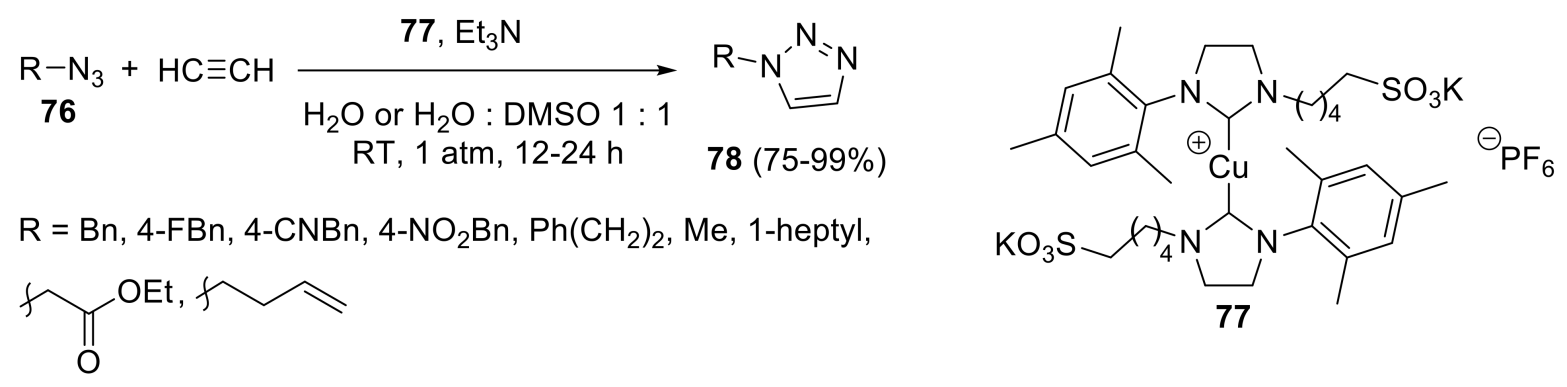
[3 + 2]-Cycloaddition of azides to acetylene, method 2.

A water soluble catalyst **77** based on *N*-heterocyclic carbenes-Cu complex for [3 + 2]-cycloaddition of azides **76** and acetylene was proposed [122]. Various alkyl and benzyl triazoles **78** were obtained in high yields using **77** as catalyst. The reactions were carried out in water or 1:1 DMSO/water mixture (Scheme 44).

**Scheme 45:**
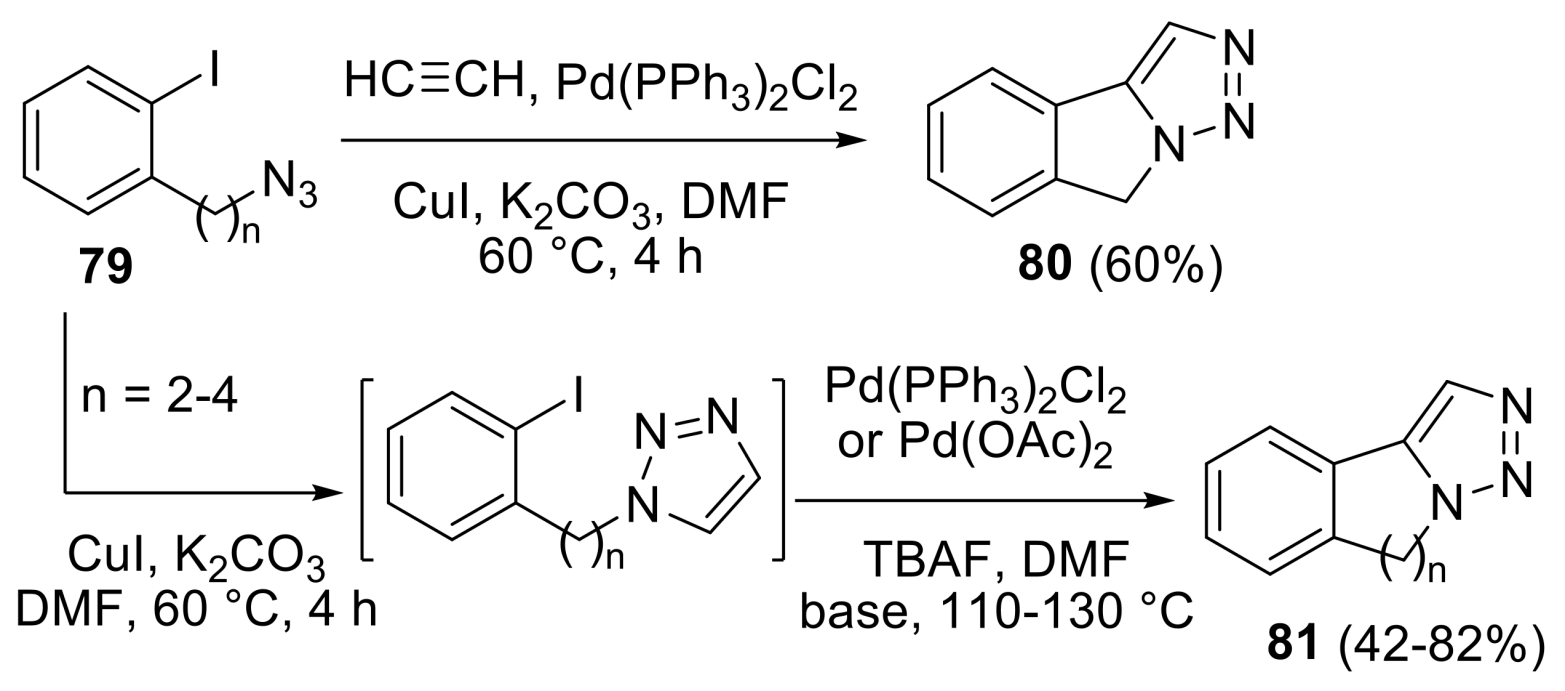
Click-reaction of *o*-iodo-azides with C_2_H_2_.

Triazole-fused benzoheterocycles were prepared via Cu-catalyzed click-reaction of *o*-iodo-azides **79** with acetylene and subsequent Pd-catalyzed intramolecular coupling [123]. The triazole-fused isoindole derivative **80** can be obtained in one-pot manner. Obtaining of six-, seven- and eight-membered heterocycles **81** requires a two-step procedure (Scheme 45).

**Scheme 46:**
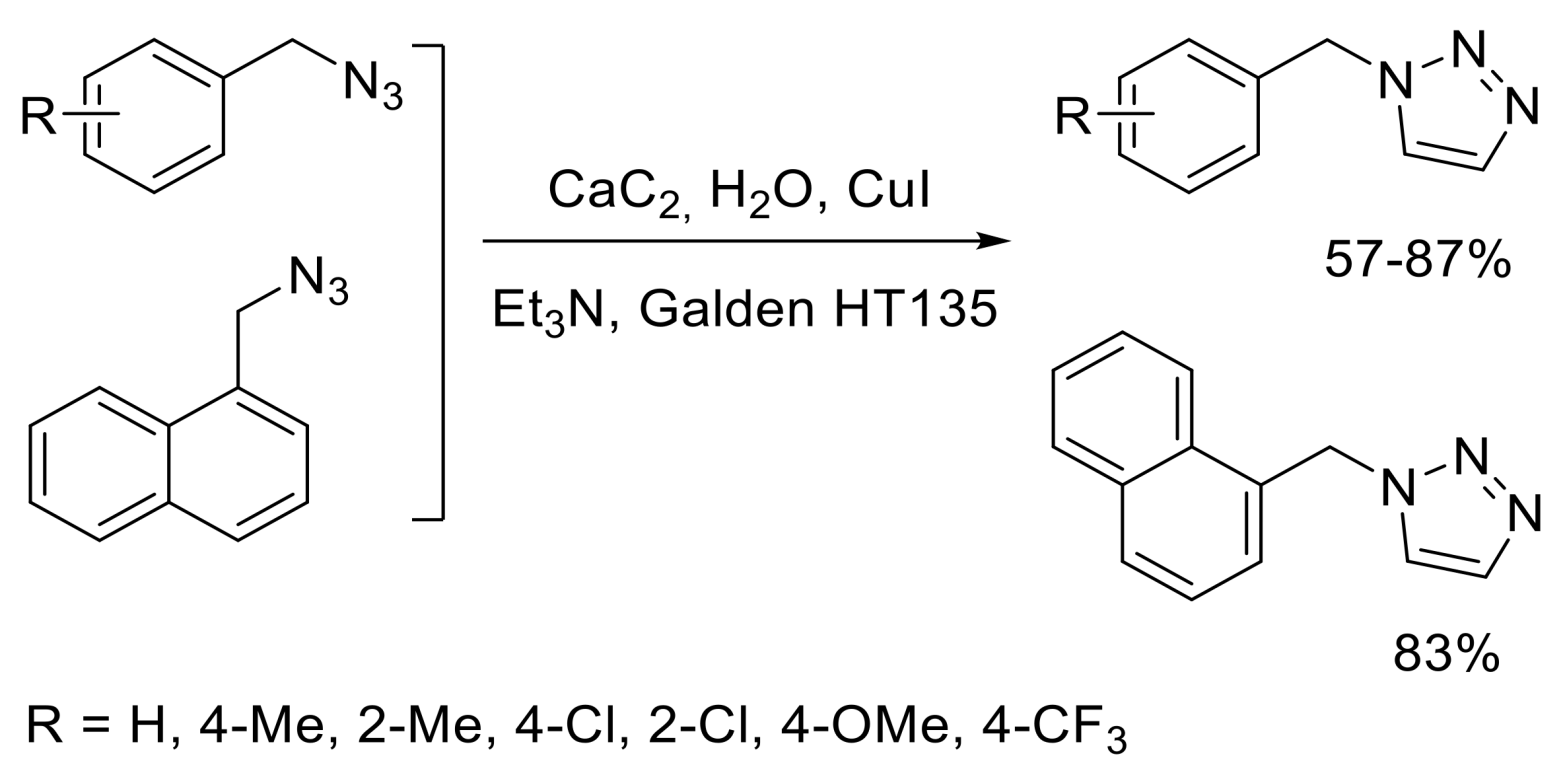
Cycloaddition with CaC_2_.

Identical Cu-catalyzed cycloadditions can be performed with calcium carbide instead of gaseous acetylene [42]. Triethylamine serves as a solvent and base simultaneously, while Galden HT135 effectively separates CaC_2_ from the organic phase. Benzyl azides with variously substituted phenyl rings undergo cyclization with the released acetylene to afford good yields of the corresponding triazoles (Scheme 46).

Similar 1,3-dipolar cycloadditions of the calcium carbide-derived acetylene to diazo compounds [126], nitrile oxides [41] and nitrile imines [43] were reported.

**Scheme 47:**
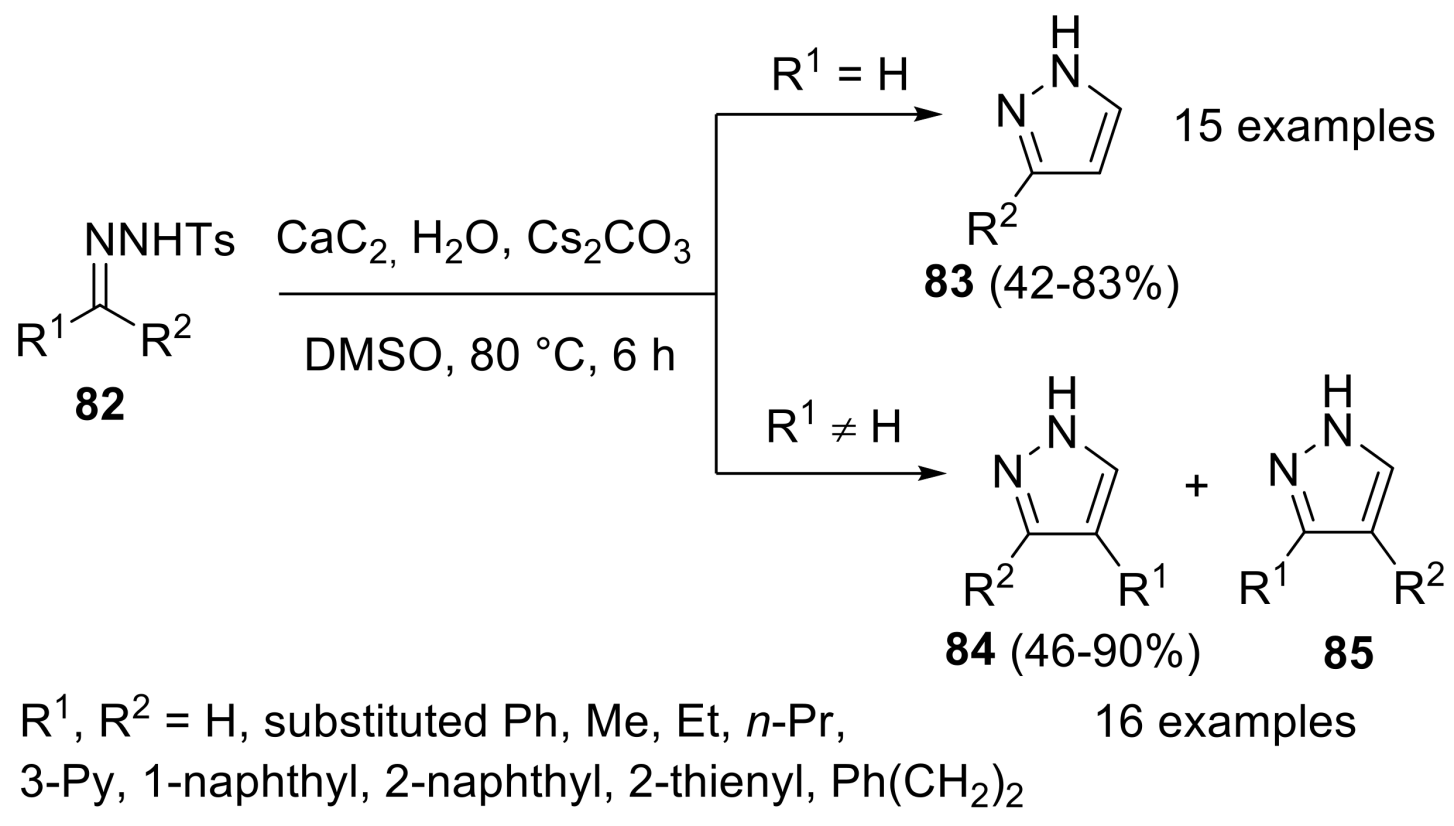
Synthesis of NH-pyrazoles from N-tosylhydrazones.

Calcium carbide was used as an acetylene source to react with a wide range of *N*-tosylhydrazones **82** derived from aldehydes or ketones, affording various substituted pyrazoles in good yields with high regioselectivities [126]. When *N*-tosylhydrazones were derived from aldehydes, the starting materials were smoothly transformed into the corresponding products **83**. Ketone-derived *N*-tosylhydrazones afforded the pyrazoles **84** and **85** with regioselectivities of more than 5:1 (Scheme 47).

**Scheme 48:**
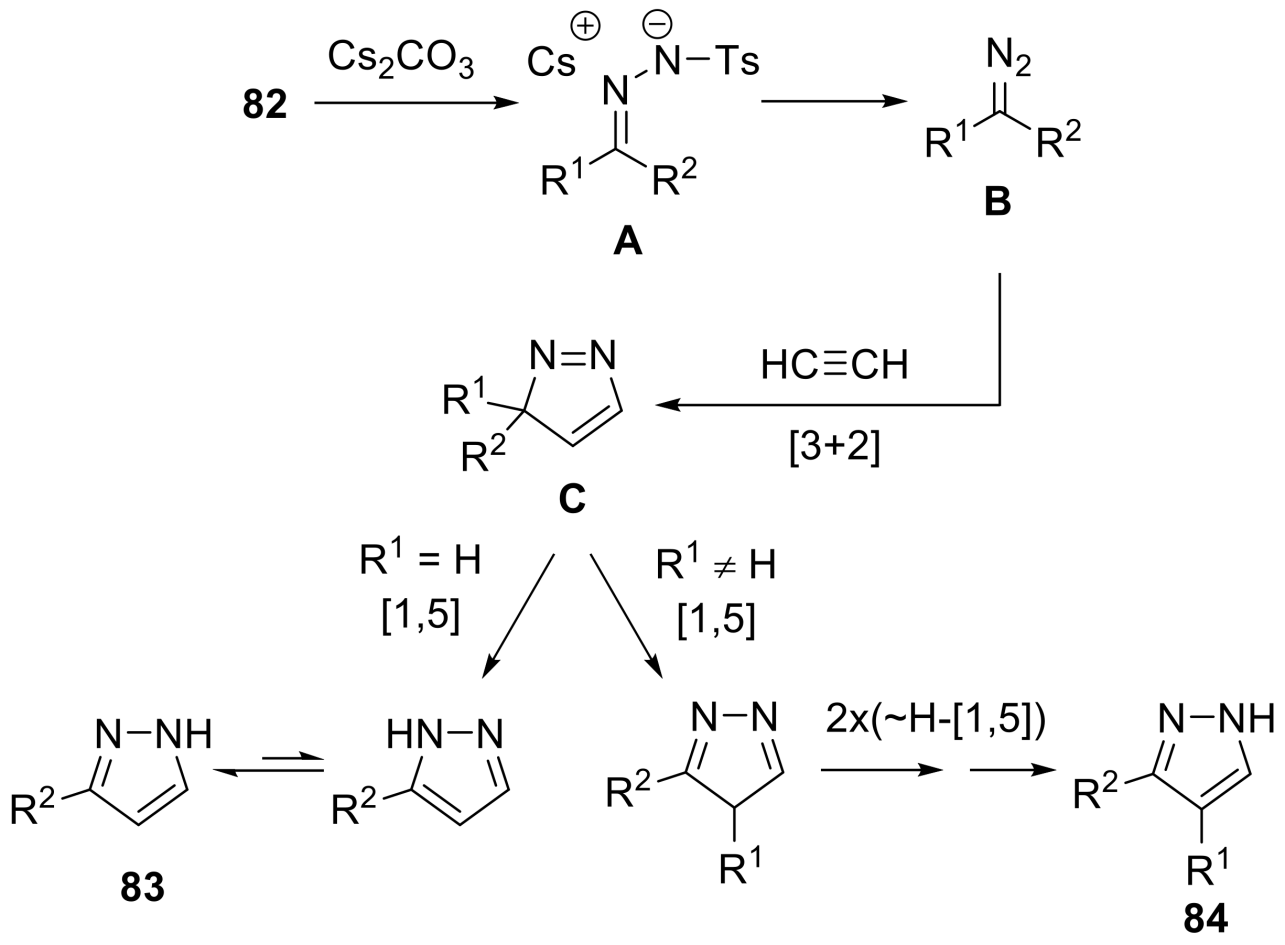
*NH*-Pyrazole formation mechanism.

The mechanism of these transformations plausibly involves [3 + 2]-cycloadditions of in situ generated diazo compounds followed by [1,5]-sigmatropic rearrangements (Scheme 48) [126]. Initial deprotonation of N-tosylhydrazone **82** in the presence of Cs_2_CO_3_ gives cesium salt **A**, which undergoes rapid transformation into reactive diazo compound **B**. Its [3 + 2]-cycloaddition with acetylene affords a 3*H*-pyrazole compound **C**. When R^1^ is H, **83** is formed via [1,5-H]-shift and tautomerization. When R^1^ is aryl and R^2^ is alkyl, migration of R^1^ or R^2^ to C4 occurs. Subsequent multiple [1,5]-sigmatropic rearrangements result in formation of **84** or **85**. Since the migration ability of electron-rich aryl group is higher than that of alkyl group [126], migration of R^1^ to C4 is favored, and pyrazoles **84** predominate as major products. This proposed mechanism was supported by deuterium-labeling experiments [126]. 

**Scheme 49:**
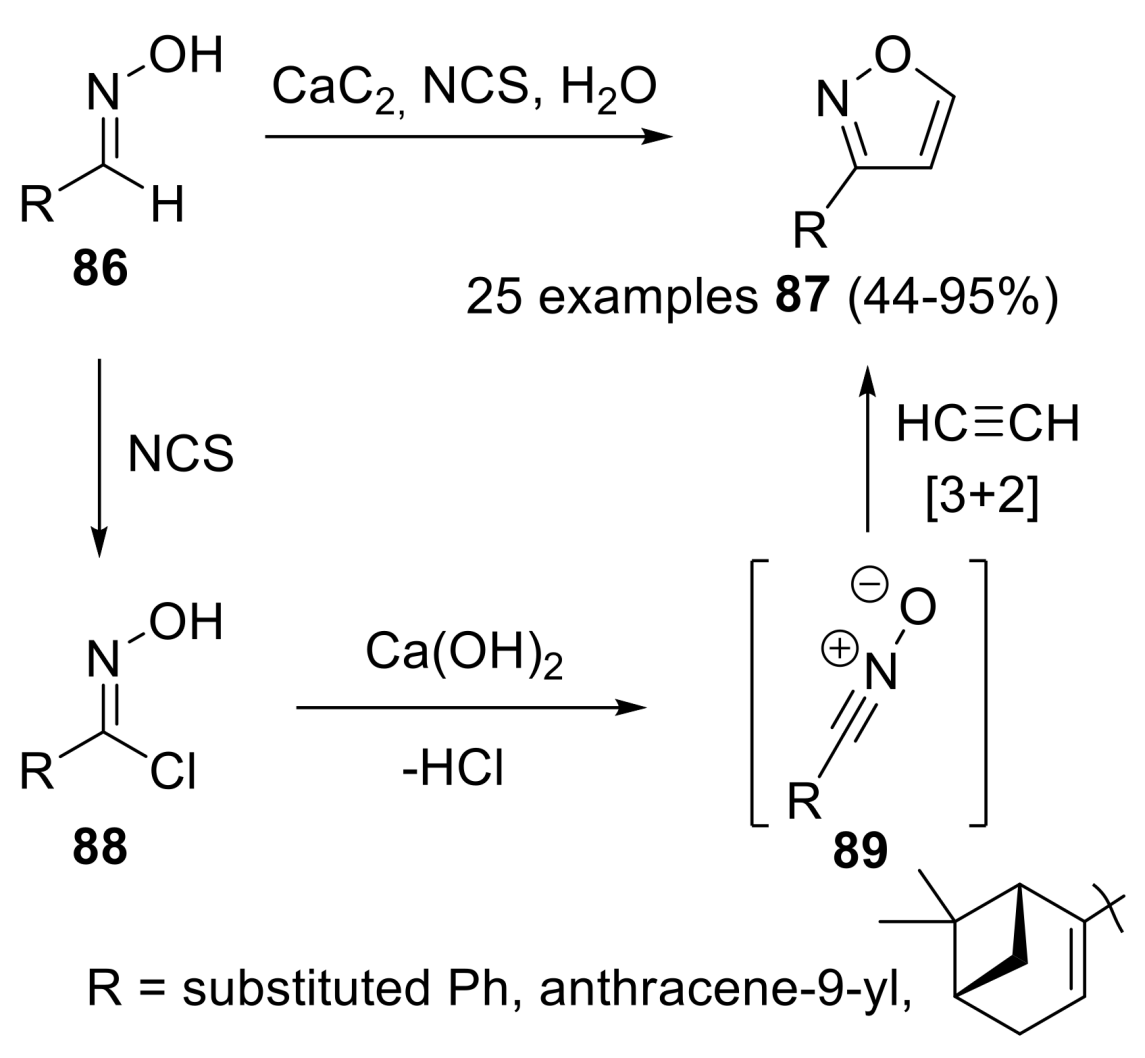
CaC_2_-mediated synthesis of isoxasoles from aldoximes.

A convenient synthetic methodology for the one-pot preparation of isoxazoles **87** directly from the reaction of calcium carbide with aldoximes **86** was developed [41]. This method is based on a 1,3-dipolar cycloaddition of nitrile oxides to acetylene. Nitrile oxides precursors, chloroaldoximes **88** were formed in situ by aldoxime chlorination with *N*-chlorosuccinimide (NCS). Reaction of CaC_2_ with the water provides the sources of both acetylene and Ca(OH)_2_ base to enable generation of nitrile oxides **89**. Various 3-substituted isoxazoles **87** were synthesized from the corresponding aldoximes **86** in up to 95% yields (Scheme 49). 

**Scheme 50:**
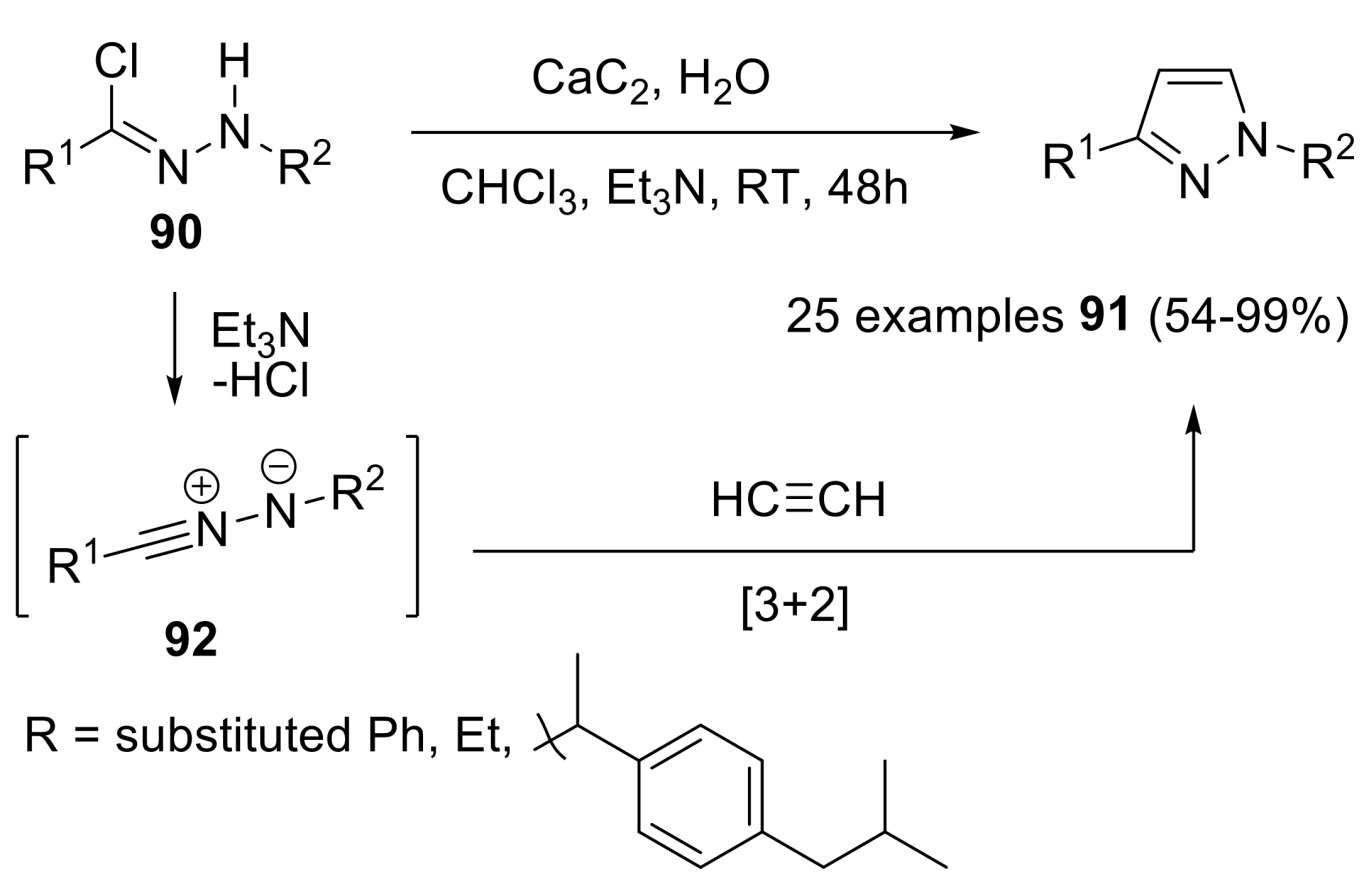
Obtaining 1,3-disubstituted pyrazoles by [3 + 2]-cycloaddition.

An efficient protocol for the preparation of 1,3-disubstituted pyrazoles **91** from [3 + 2]-cycloaddition reactions of in situ generated nitrile imines **92** with CaC_2_-derived acetylene was reported [43]. The reactions were performed in a two-chambered reactor in order to separate the water-sensitive nitrile imines from the acetylene-generating mixture. One part of the reactor was loaded with hydrazonoyl chloride precursors **90** of active nitrile imine species and Et_3_N as a base. The other part was used to generate acetylene from CaC_2_ and water. Escaping from the carbide chamber, acetylene readily dissolves in the reaction mixture with nitrile imines freshly derived from hydrazonoyl chlorides and Et_3_N as a base. A variety of 1,3-disubstituted pyrazoles **91** were produced in up to quantitative yields by this approach (Scheme 50).

Utilization of carbide and water as an acetylene source in cycloaddition reactions is not just safe and convenient, but allows to obtain deuterium-labeled heterocycles by using deuterated water, as exemplified by syntheses of 4,5-dideuteroisoxazoles and 4,5-dideuteropyrazoles [41,43]. 

**Scheme 51:**
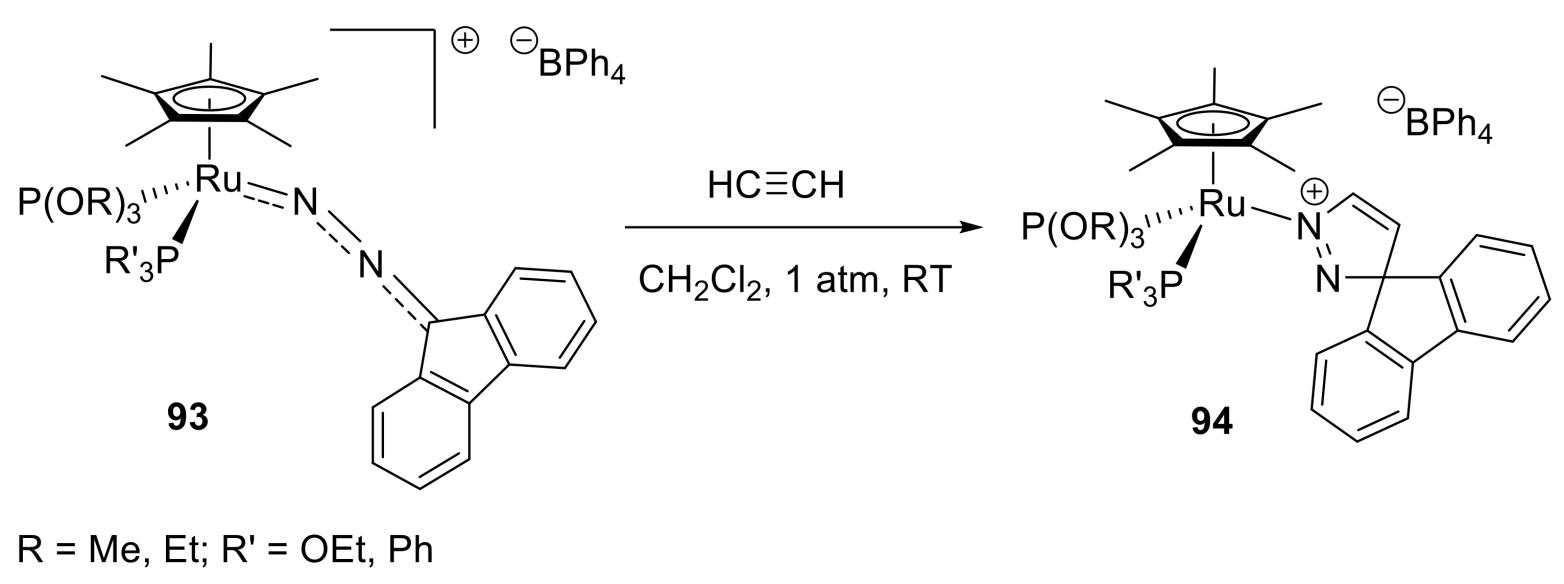
Synthesis of 3*H-*pyrazole derivatives of diazoalkane dipole complexes.

Unusual dipoles, diazoalkane complexes **93** reacted with acetylene under mild conditions [127]. The reactions proceeded through the [3 + 2]-cycloaddition of the acetylene to the coordinated diazoalkane, yielded 3*H*-pyrazole derivatives **94** (Scheme 51).

**Scheme 52:**
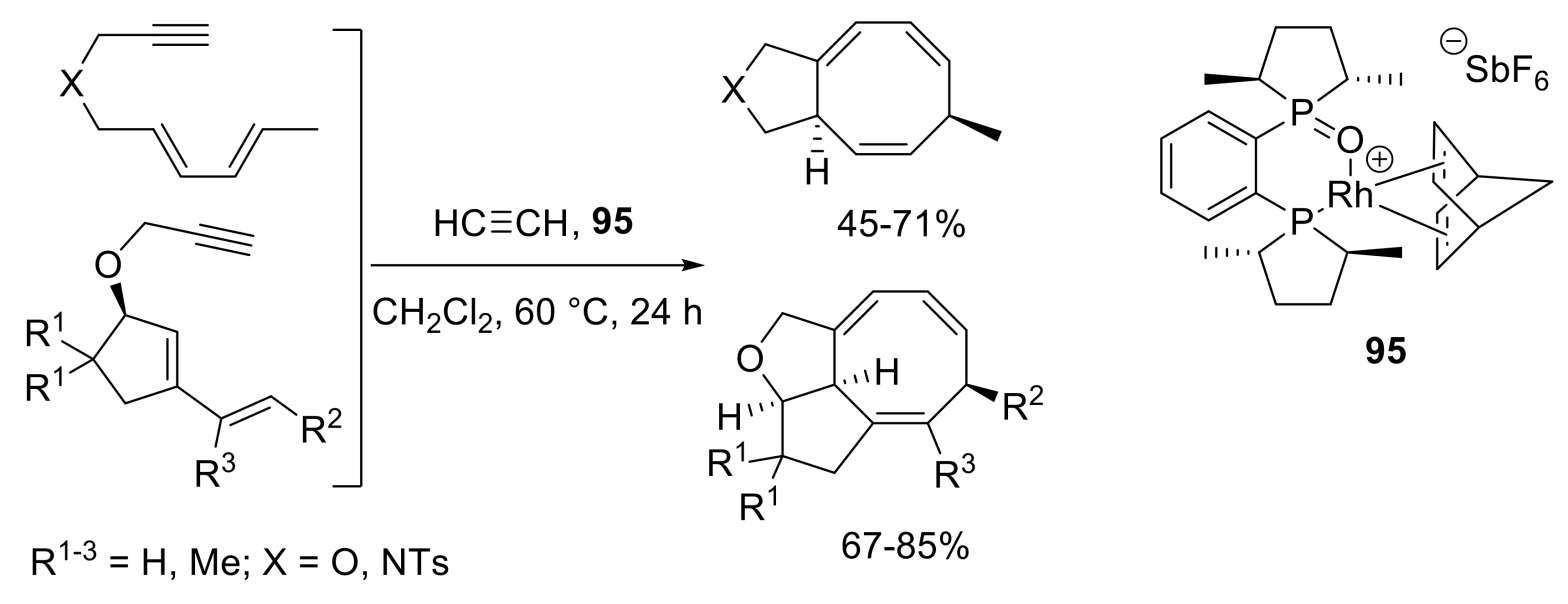
Three-component cycloaddition with acetylene.

Three-component cycloaddition reactions of acetylene were also described [129,130,131,132]. Rhodium-catalyzed [4 + 2 + 2]-cycloaddition of dienynes and acetylene gave rise to oxygen- and nitrogen-containing polycyclic cyclooctatrienes in moderate to good yields (Scheme 52) [129]. The desired products were obtained in greater than 20:1 diasteromeric excess.

**Scheme 53:**
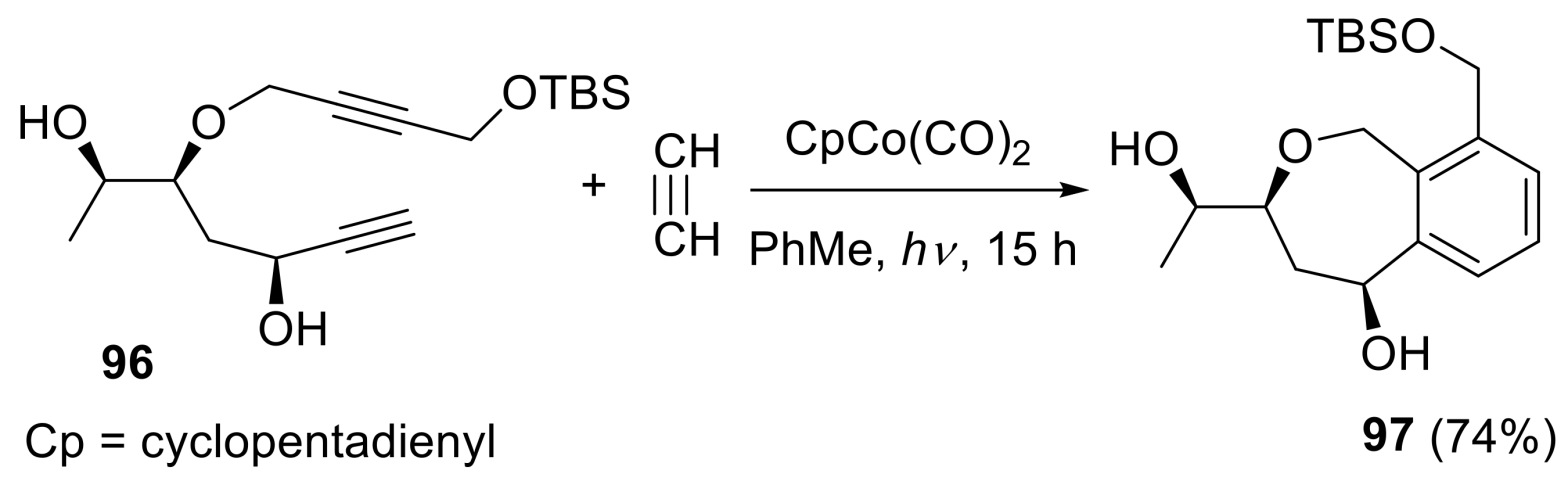
Cobalt-catalyzed [2 + 2 + 2]-cycloaddition.

Cobalt-mediated [2 + 2 + 2]-cycloaddition reaction of acetylene and diyne **96** afforded bicyclic product **97**, which was employed as an intermediate in the total synthesis of the putative structure of xylarinol B (Scheme 53) [130]. 

Synthesis of boron-containing heterocycles [131,132] through [2 + 2 + 2]-cycloaddition reactions involving acetylene was also reported. In addition, [2 + 2]-cycloaddition with acetylene were used for the preparation of Ge- [133], Si- and Ti-containing [134] inorganic heterocycles.

#### 2.4.2. Trofimov Reaction

**Scheme 54:**
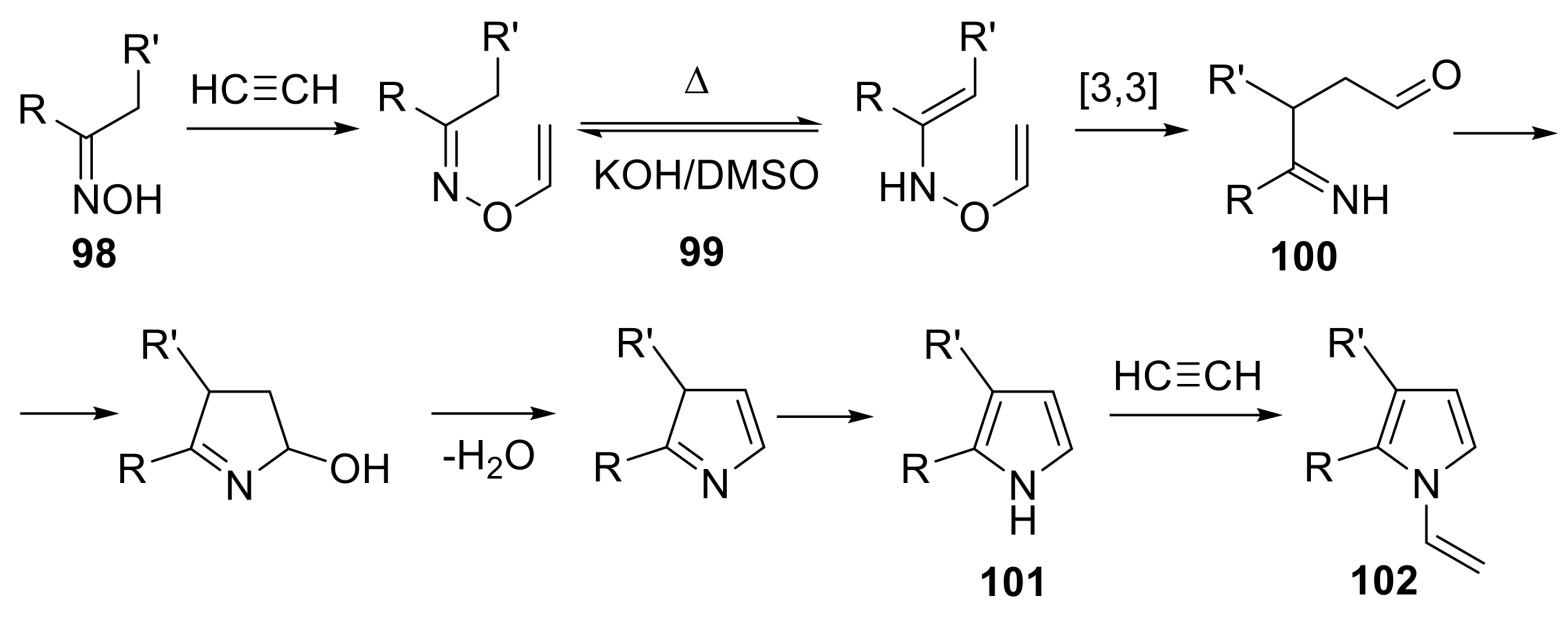
Trofimov reaction.

Reaction of ketoximes with acetylene under superbasic conditions, known as Trofimov reaction, is widely used for synthesis of pyrroles and 1-vinylpyrroles [13,39]. The reaction mechanism [39] involves initial O-vinylation of starting oxime **98** and subsequent [3,3]-sigmatropic shift in the intermediate O-vinyl oxime **99** to form aldehyde-imine compound **100**. The aldehyde-imine intermediate undergoes a cyclization-dehydration-tautomerization sequence to give pyrrole **101**. Further reaction with the second acetylene molecule leads to the formation of N-vinyl pyrrole **102** (Scheme 54).

**Scheme 55:**
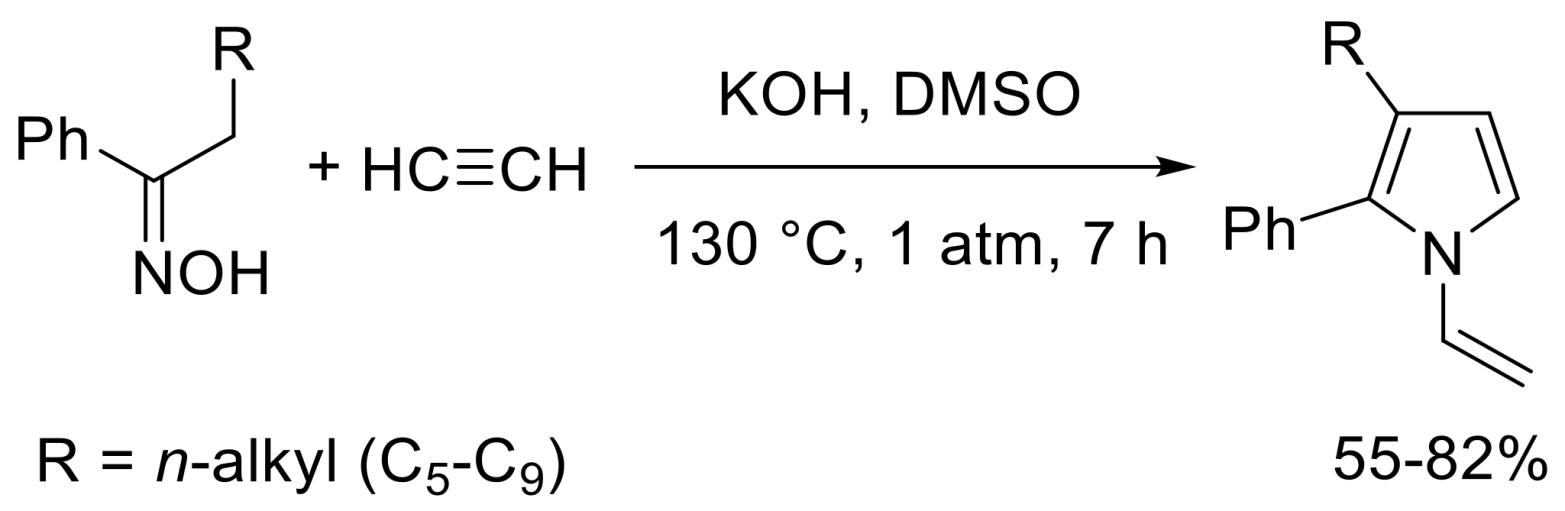
A modified procedure for obtaining 3-alkyl-2-phenyl-1-vinylpyrroles.

Heterocyclization of ketoximes with acetylene into pyrroles and N-vinylpyrroles is normally performed in an autoclave under an initial acetylene pressure of 8-16 atm (most commonly 10–12 atm) [135] with KOH-DMSO as a superbase [135]. A recently developed modification of the procedure involves a flow reactor [136]; it allows to perform the synthesis of 3-alkyl-2-phenyl-1-vinylpyrroles under atmospheric acetylene pressure (Scheme 55) [137]. 

**Scheme 56:**
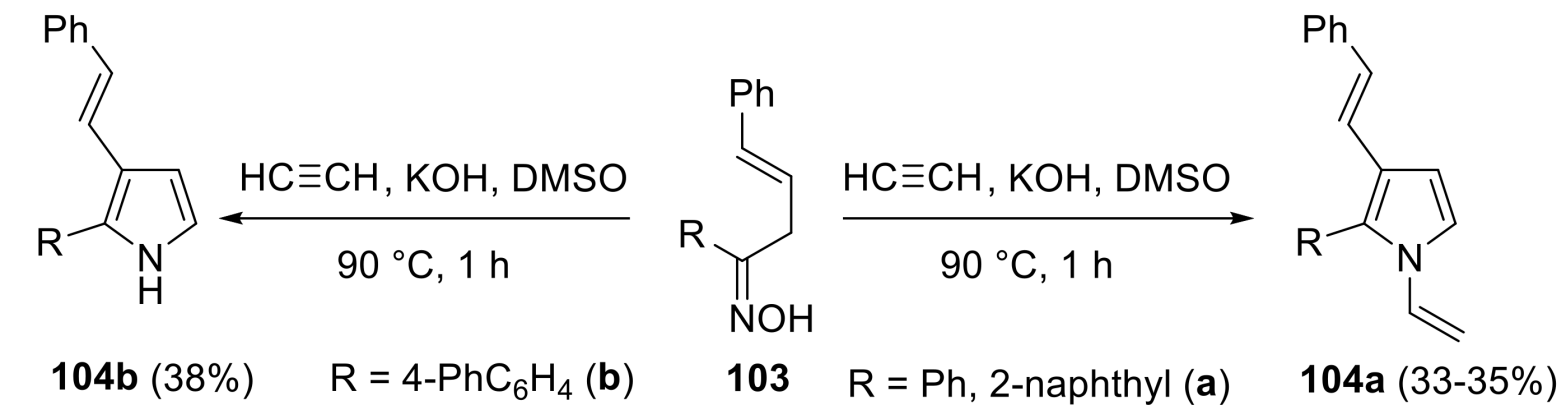
Synthesis of 3-(*E*)-styrylpyrroles from (*E*)-styrylmethyl ketoximes.

The substrate scope of Trofimov reaction is constantly expanding [138,139,140]. The synthesis of 3-(*E*)-styrylpyrroles **104** from (*E*)-styrylmethyl ketoximes **103** and acetylene was recently reporded [138]. Phenyl- and naphthyl-derivatives **104a** gave rise to the corresponding 1-vinylpyrroles **104a**. 1*H*-Pyrrole **104b** was obtained from biphenyl-derived oxime **103b** (Scheme 56).

**Scheme 57:**
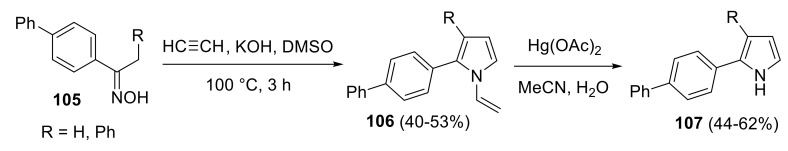
Trofimov heterocyclization of bifenyl-derived oximes.

Other biphenyl-derived oximes **105** reacted with acetylene under superbasic conditions to afford predominantly 1-vinylpyrroles **106** [139]. 1-Unsubstituted pyrroles **107** can be obtained by devinylation of 1-vinylpyrroles **106** in the presence of mercuric acetate (Scheme 57) [139]. 

**Scheme 58:**
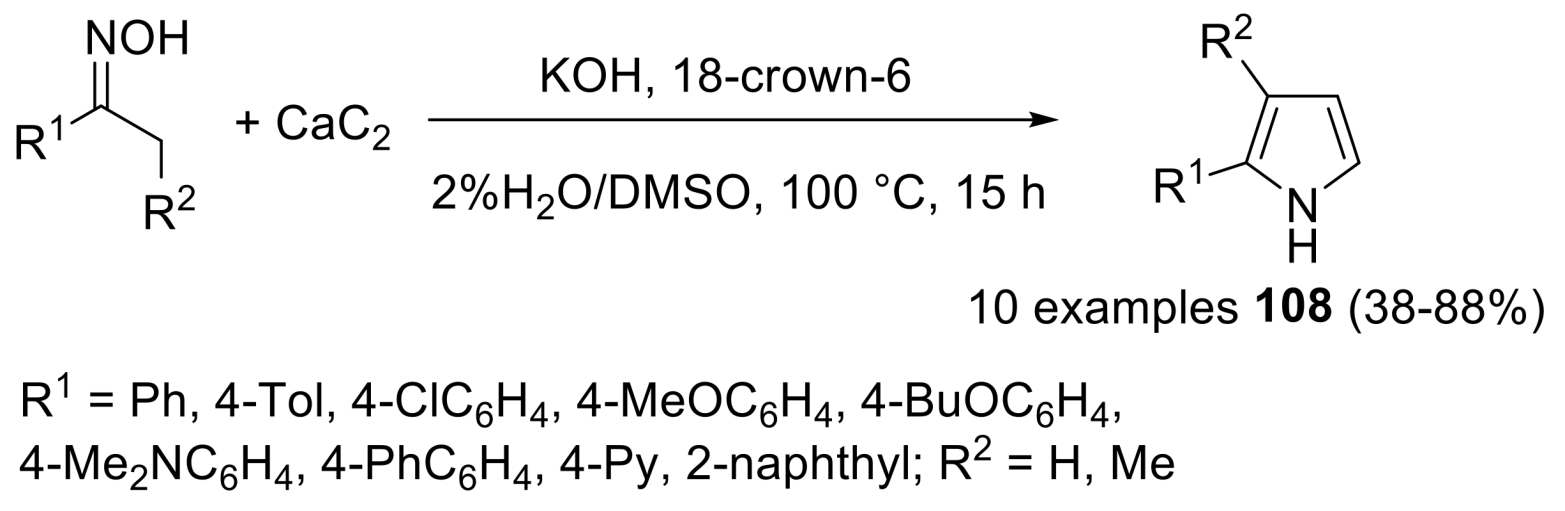
Synthesis of 2-arylpyrroles with calcium carbide and water.

Various 2-arylpyrroles **108** were synthesized through the Trofimov reaction directly from calcium carbide as an acetylene source (Scheme 58) [40].

**Scheme 59:**
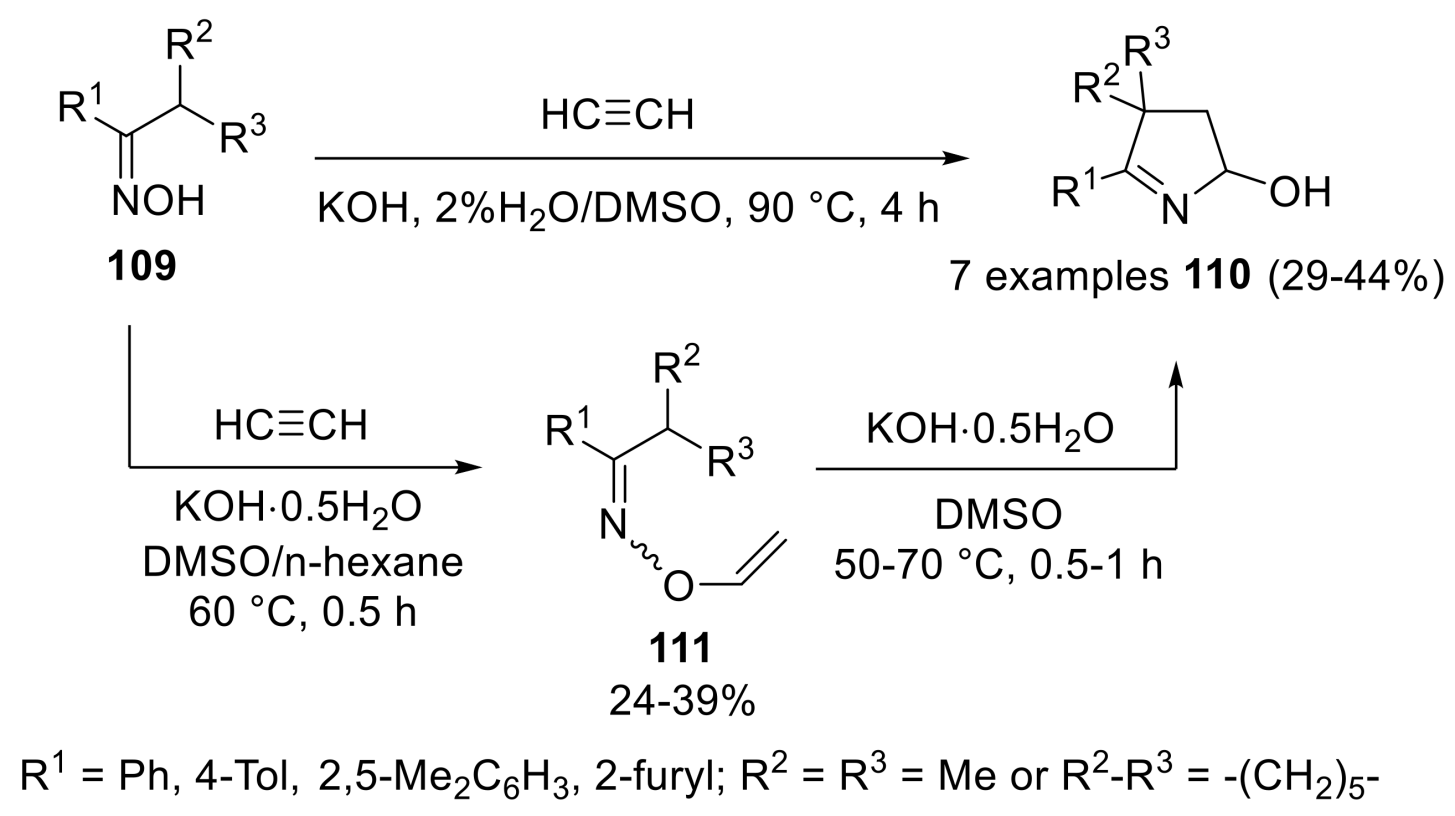
Reaction of acetylene with ketoximes.

The reactions were performed using DMSO with 2% of water as a solvent. 18-Crown-6 was used as a catalyst to improve the pyrroles yields. It proceeds selectively and results in formation of 1*H*-pyrroles with only trace amounts of 1-vinylpyrroles When using ketoximes with only one neighboring C-H bond, the formation of 1H-pyrroles is inhibited. In such cases the intermediate 5-hydroxypyrrolines [141,142] and 3*H*-pyrroles [143,144] can be isolated as main products. Reactions of acetylene with various *sec*-alkyl aryl ketoximes **109** afforded the corresponding 5-hydroxypyrrolines **110** in moderate yields [141,142]. The reaction can be performed in a one-pot manner [141] or in a two-step sequence [142] isolating O-vinylketoxime intermediates **111** in low yields (Scheme 59).

#### 2.4.3. Other Reactions for Accessing Heterocyclic Molecules

**Scheme 60:**
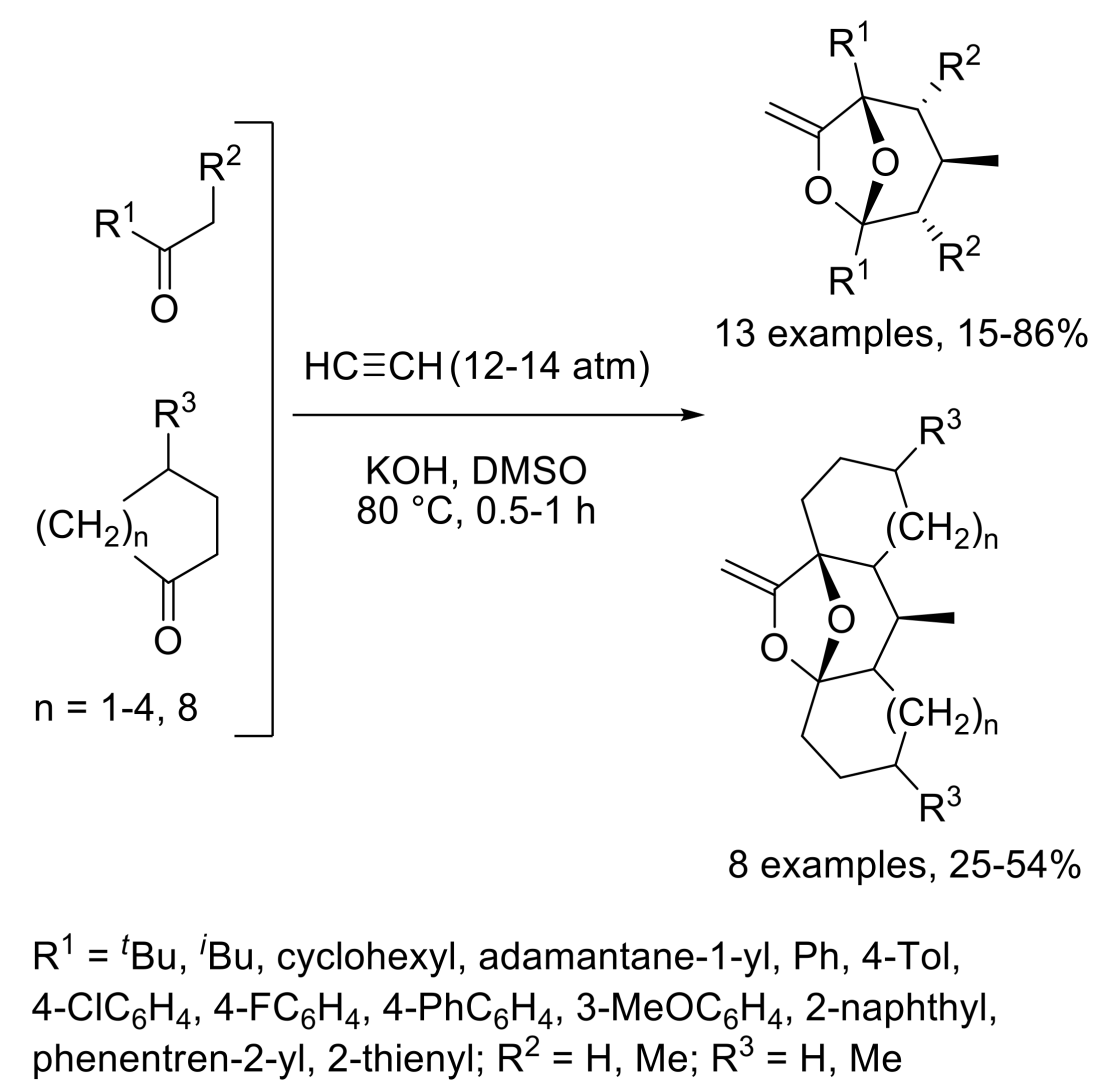
Reaction of acetylene and ketones.

As an alternative to Favorskii reaction, ketones and acetylene can undergo a one-pot cascade cyclization to give 7-methylene-6,8-dioxabicyclo[3.2.1]octanes [145,146,147,148,149,150]. Under optimized conditions [145,146] a variety of bicyclic products were obtained from dialkyl [145] and aryl alkyl [146,147] ketones (Scheme 60).

**Scheme 61:**
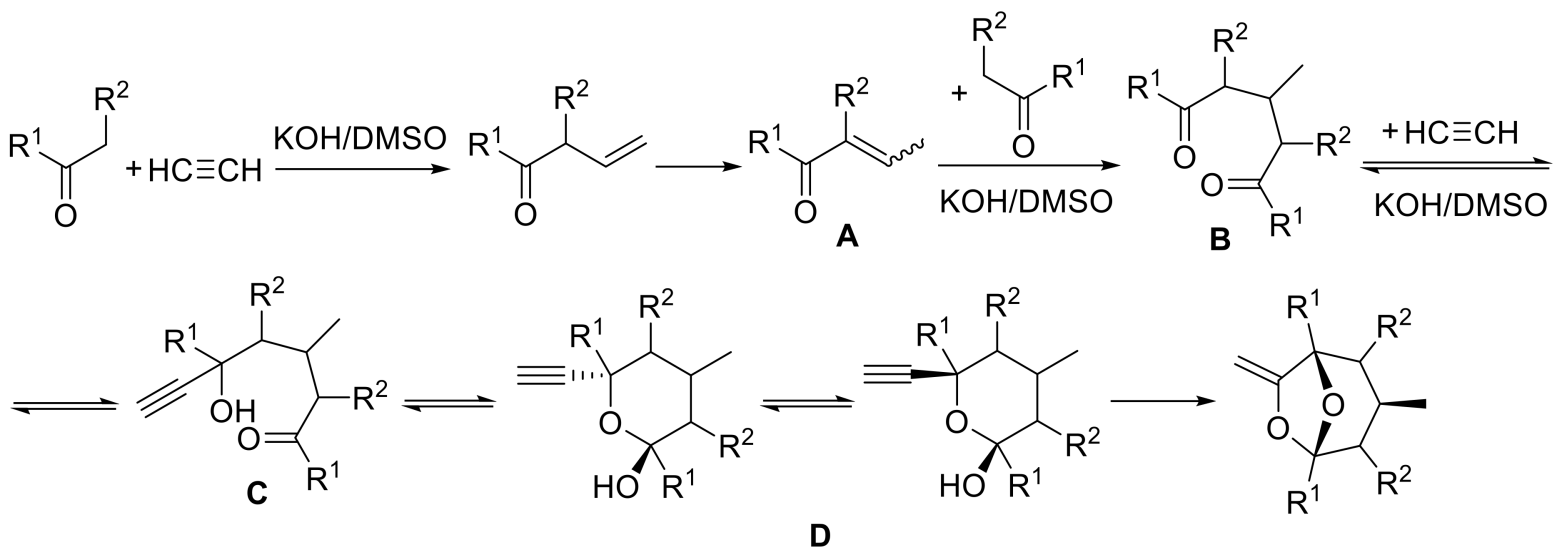
Plausible mechanism of acetylene and ketones reaction.

A proposed mechanism for this reaction involves the following cascade transformations [145]. A superbase-promoted addition of the initial ketone to acetylene yields an intermediate 2-propenyl ketone, which undergoes prototropic isomerization into 1-propenyl ketone **A**. The next step involves Michael addition of a second initial ketone molecule to **A** thus affording 1,5-diketone **B**. Further on, an acetylene unit is added to a carbonyl group of **B** to form acetylenic ketoalcohol **C**. Subsequent cascade cyclizations include reversible formation of hemiacetal **D**, which undergoes nucleophilic addition to the triple bond and becomes the exocyclic enol ether moiety in the final product (Scheme 61).

**Scheme 62:**
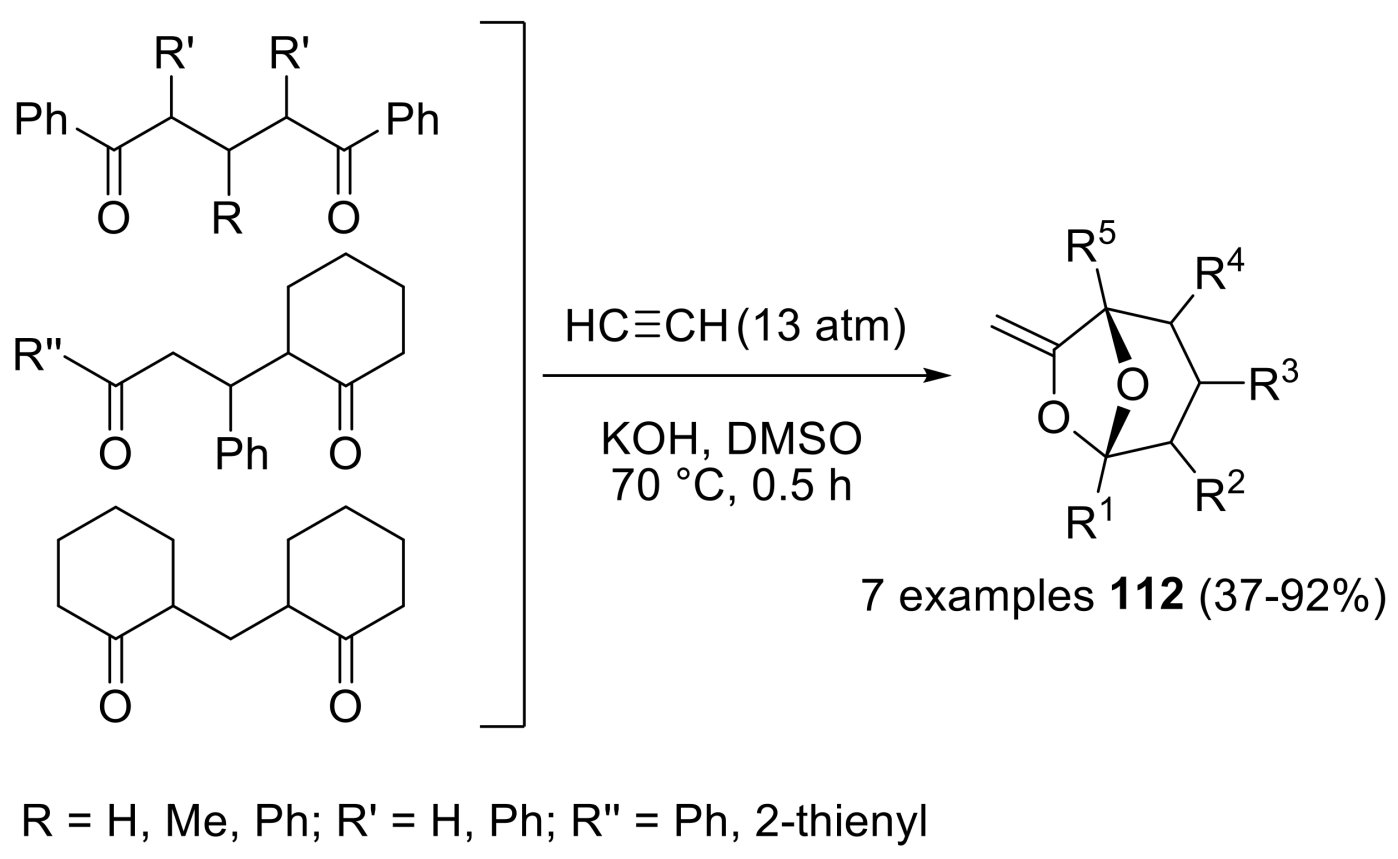
Cascade cyclization of ketones with acetylene under superbase conditions.

The mechanism implies that 1,5-diketones readily cyclize with acetylene under superbasic conditions to give 7-methylene-6,8-dioxabicyclo[3.2.1]octanes. Indeed, the alkyl and aryl substituted 1,5-diketones are susceptible to cascade cyclization with acetylene [148]. The reactions were performed under conditions similar to those for the monoketones and afforded both symmetrically and unsymmetrically substituted 7-methylene-6,8-dioxabicyclo[3.2.1]octanes **112** (Scheme 62).

**Scheme 63:**
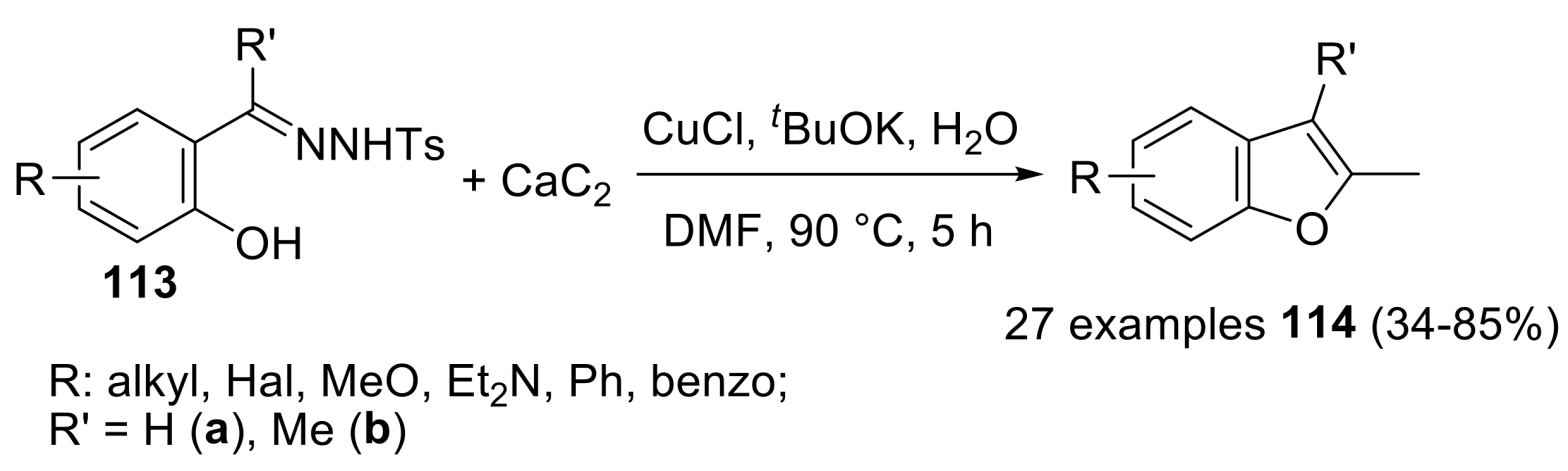
Calcium carbide-based synthesis of benzofuran derivatives.

A recently reported synthesis of methyl-substituted benzofuran rings resulted from combination of calcium carbide-derived acetylene with salicylaldehyde *p*-tosylhydrazones **113a** and 2-hydroxyacetophenone *p*-tosylhydrazones **113b** [151]. The reactions were performed with a copper chloride catalyst. Various 2-methylbenzofurans **114a** and 2,3-dimethylbenzofurans **114b** were obtained in satisfactory yields (Scheme 63).

**Scheme 64:**
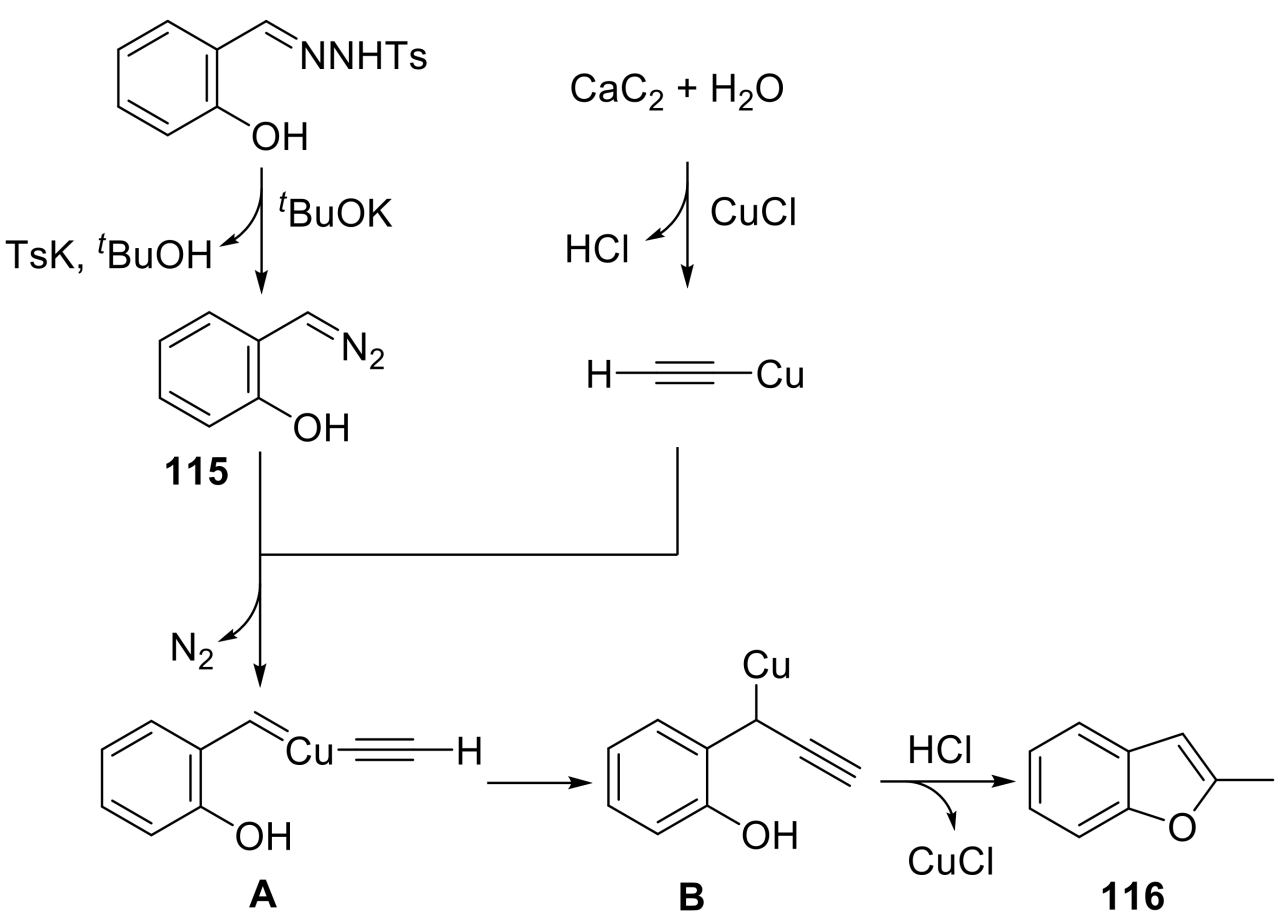
Mechanism of benzofuran formation.

The following mechanism was proposed for this reaction (Scheme 64) [151]. Initial reaction of calcium carbide with water in the presence of CuCl gives ethynylcopper as an intermediate and coincides with the in situ formation of 2-(diazomethyl)phenol **115**. The ethynylcopper intermediate reacts with 2-(diazomethyl)phenol to give a copper-carbene species **A**, which transforms into **B** by reductive elimination. Subsequent acidolysis of **B** leads to intramolecular cyclization to afford the final product **116**.

A palladium-catalyzed cascade protocol for the synthesis of 4-methyl-1-(1*H*-pyrrolo[2,3-*b*]-quinoxalin-2-yl)cyclohexanols **119a** and 2-phenyl-1-(1*H*-pyrrolo[2,3-*b*]quinoxalin-2-yl)propan-1-ols **119b** from *N*-alkyl(aryl)-3-chloroquinoxalin-2-amines **117**, calcium carbide and cyclohexanones **118a** or 2-phenylpropanal **118b** has been developed [152].

**Scheme 65:**
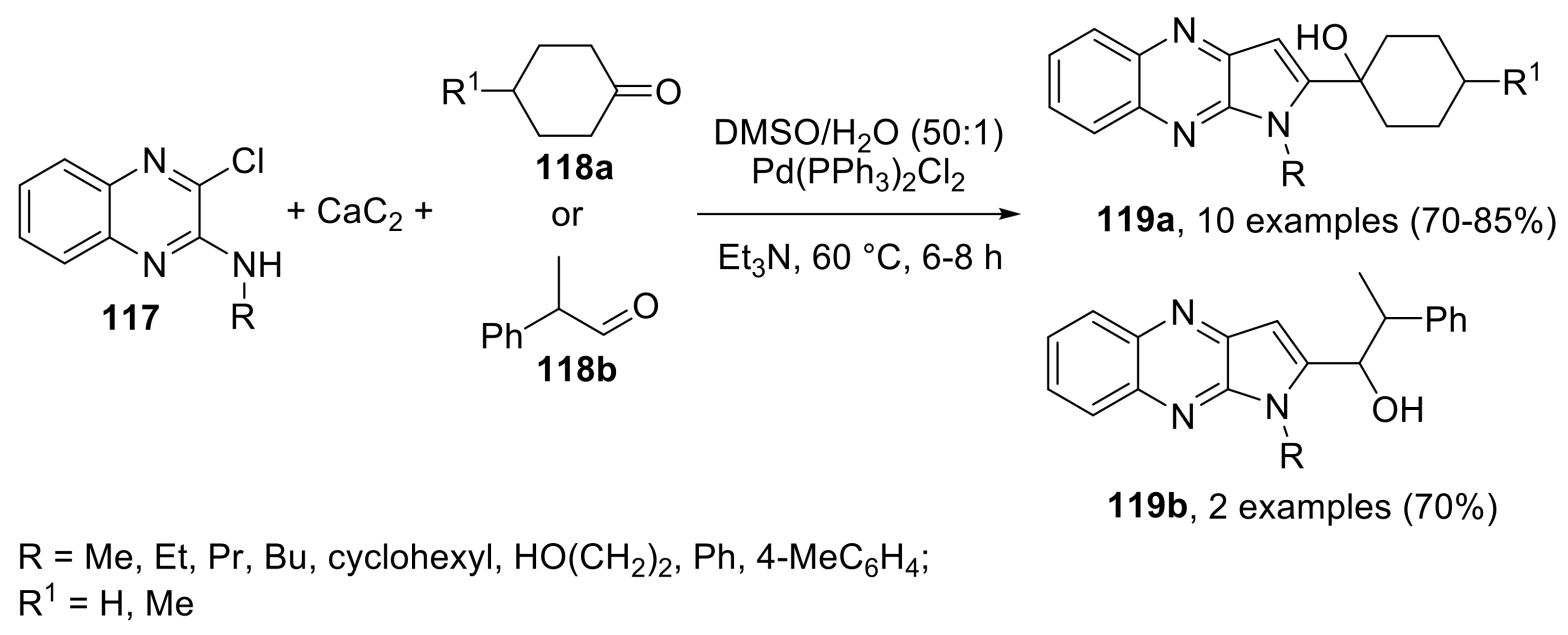
Pd-catalyzed amine/aldehyde/acetylene reaction.

**Scheme 66:**
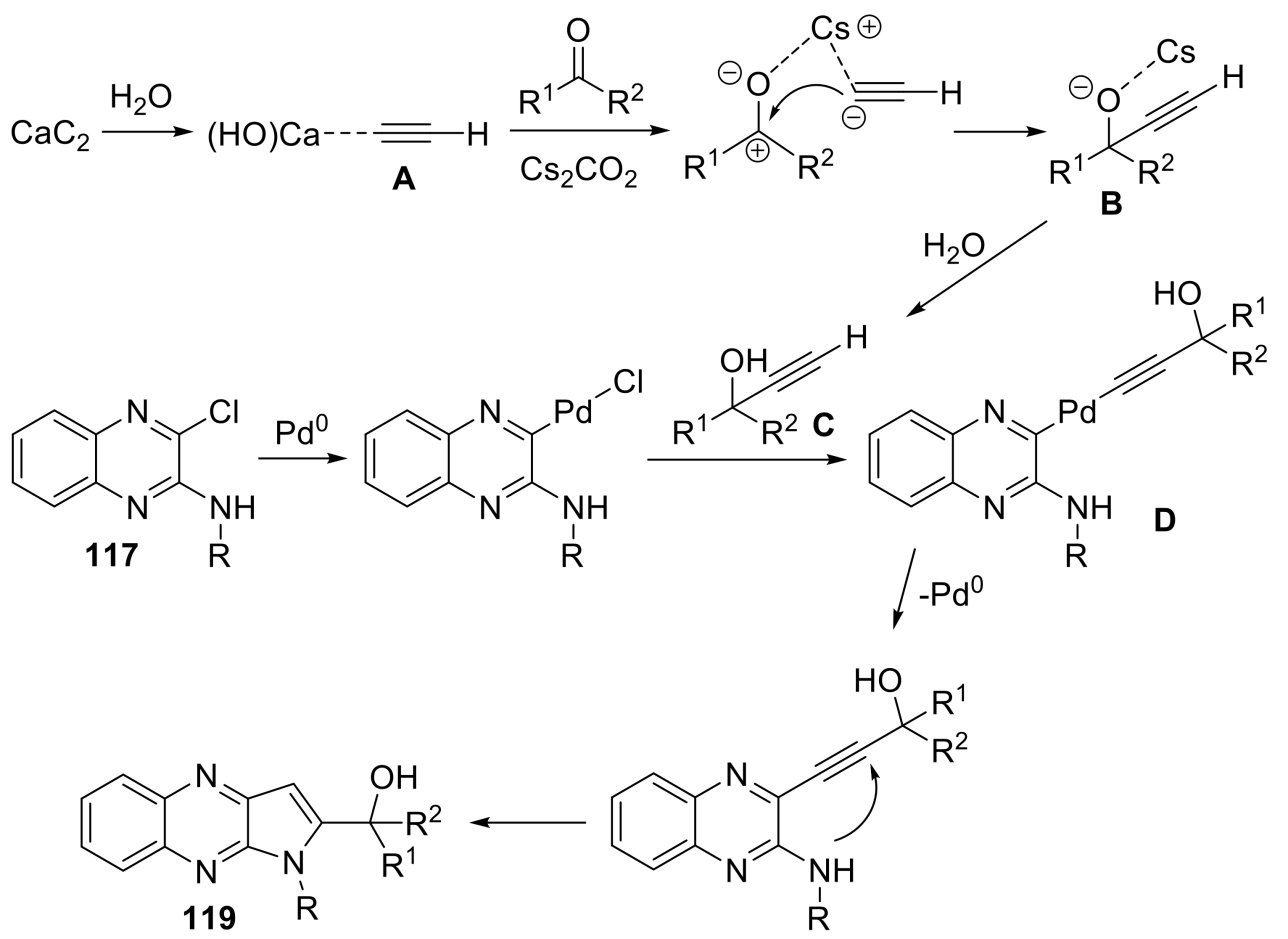
Proposed mechanism of three component coupling.

The reactions were performed in the presence of catalytic amounts of Pd(PPh_3_)_2_Cl_2_ in a DMSO/H_2_O solvent mixture. This copper-free one-pot Sonogashira-type synthesis provided high yields of 2,3-disubstituted pyrrolo[2,3-*b*]quinoxalines (Scheme 65)A proposed reaction mechanism [152] includes the following steps. Initially, calcium acetylide **A** is generated from CaC_2_ and water. Aldehyde or ketone activation by cesium cation promotes a nucleophilic attack of acetylide **A** on the carbonyl group to afford propargyl alcohol **C** via intermediate **B**. A subsequent palladium-catalyzed cross-coupling reaction involves heteroaryl chloride **117** and propargyl alcohol **C** to afford intermediate **D**. At the final step, ring closure by intramolecular hydroamination leads to the formation of 2-substituted pyrrolo[2,3-*b*]quinoxalines **119** (Scheme 66).

In summary, acetylene is available and flexible starting material for heterocycles synthesis. The classical protocols for 1,3-dipolar cycloaddition and Trofimov reaction are constantly modified, and new methodologies based on cascade reactions of acetylene continuously develop, as they allow obtaining fine and versatile heterocyclic compounds from simple and inexpensive chemicals.

### 2.5. Applications in Medicinal Chemistry and Drug Discovery

Acetylene is involved in drug synthesis by two chemical approaches: reactive species vinylation and Favorskii reaction.

**Scheme 67:**
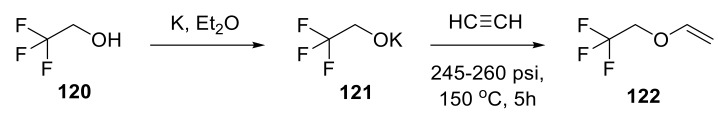
Synthesis of fluoroxene by trifluoroethanol vinylation.

Vinylation of trifluoroethanol **120** with acetylene gave an inhalation anesthetic fluoroxene **122** (Scheme 67) [153]. The transformation was performed in two steps: first, the starting alcohol was treated with potassium, and then the resulting alkoxide **121** was heated under the elevated pressure in a metal pressure reactor giving the vinyl ether **122**.

**Scheme 68:**
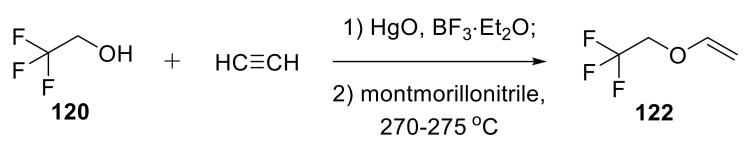
Two-step fluoroxene synthesis.

Fluroxene (**122**) was also prepared from acetylene by an alternative two-step procedure (Scheme 68) [154]. First, trifluoroethanol (**120**) reacted with acetylene in the presence of mercury oxide and boron trifluoride diethyl etherate giving di(2,2,2-trifluoroethyl) acetaldehyde acetal. The latter was heated with montmorillonitrile producing **122**.

**Scheme 69:**
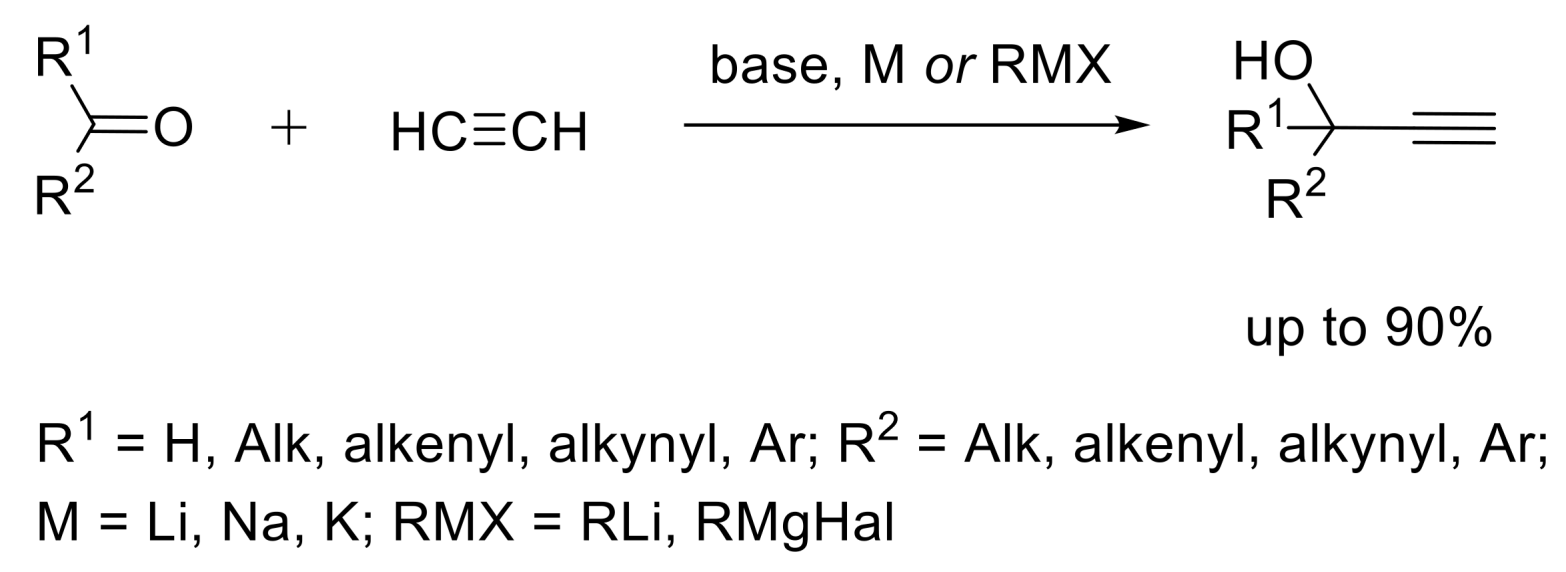
The Favorskii reaction general scheme.

The Favorskii reaction is usually performed either by using a Grignard method, or in the presence of a base (Scheme 69) [155]. The first procedure involves a preliminary reaction of acetylene with Grignard reagent or lithium, sodium, potassium in liquid ammonia or lithium-organic compounds [155,156,157]. The resulting acetylide reacts with the ketone giving a tertiary acetylenic alcohol. The second procedure is equally widespread [155,158]. Both procedures afford acetylenic alcohols from aldehydes and ketones with arbitrarily chosen substituents in moderate to excellent yields [155,156,157,158].

**Scheme 70:**
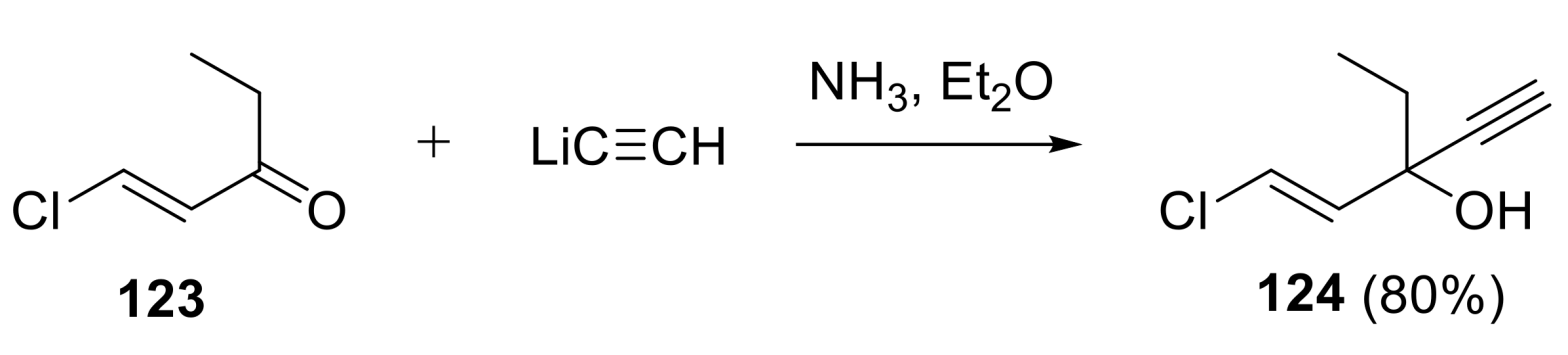
Ethchlorvynol synthesis.

A sedative drug ethchlorvynol **124** was obtained from ethyl *β*-chlorovinyl ketone **123** and lithium acetylenide in 80% yield (Scheme 70). Lithium acetylide was generated from acetylene and lithium metal in liquid ammonia. The procedure allows obtaining a range of ethynyl *β*-halogenvinyl carbinols [157]. 

**Scheme 71:**
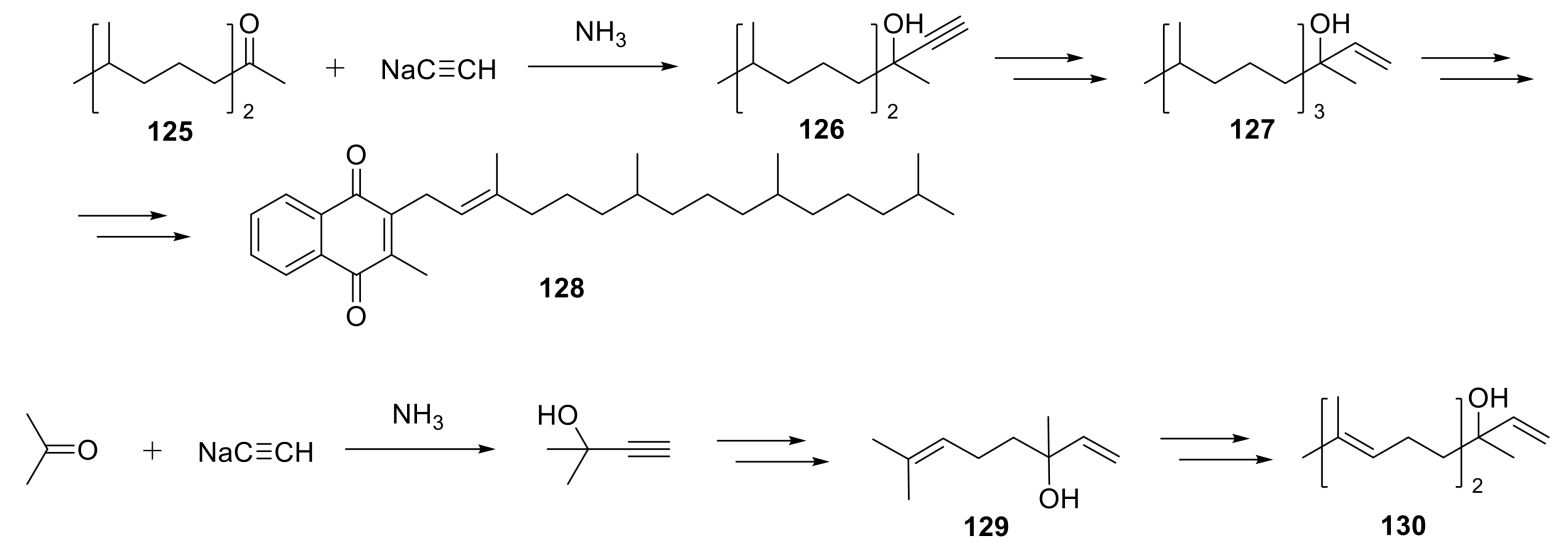
Terpene synthesis with acetylene.

Combining the Favorskii reaction with other synthetic methods, some terpenes were synthesized [156]. Starting from ketone **125** and sodium acetylide, generated by bubbling of acetylene throught sodium-ammonia mixture, the precursor **126** of isophytol **127** was obtained (Scheme 71). Isophytol **127** can be converted to *rac*-vitamin K_1_ **128** in some steps. Linalool **129** and nerolidol **130** can be obtained by similar procedures, starting from acetone and subsequently involving Favorskii reaction with partial hydrogenation and condensations [156].

The wide class of steroids ethynyl derivatives was synthesized by Favorskii reaction. These substances are used for the improvement of elderly human health and well-being quality (hormonal therapy), the treatment of rheumatic diseases, to improve sports performance and in cancer therapy [159,160]. 

**Scheme 72:**
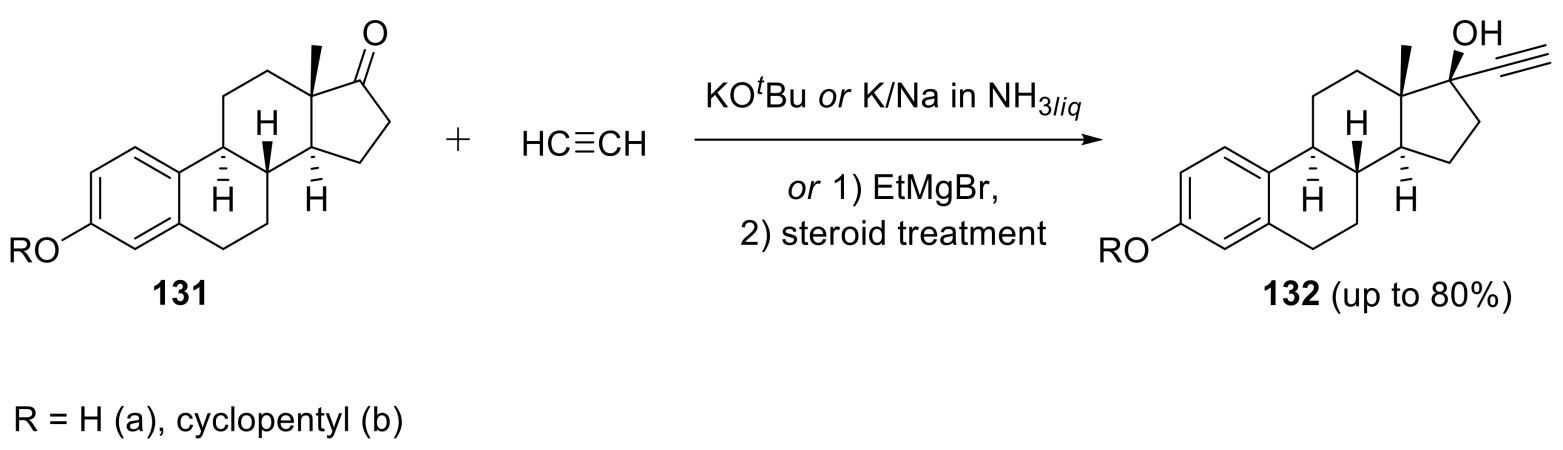
Synthesis of 17-ethynylestradiol and quinestrol.

17-Ethynylestradiol [161,162] (**132a**) and its prodrug quinestrol [163] (**132b**), which are used in female hormonal therapy, were obtained from estrone (**131a**) and estrone 3-cyclopentyl ether (**131b**), correspondingly (Scheme 72). Both ethynyl derivatives can be produced from acetylene in the presence of strong bases as catalysts or from ethynyl magnesium bromide or ethynyl lithium as a starting material [161,162,163]. Quinestrol can be produced by etherification of 17-ethynylestradiol [159].

**Scheme 73:**
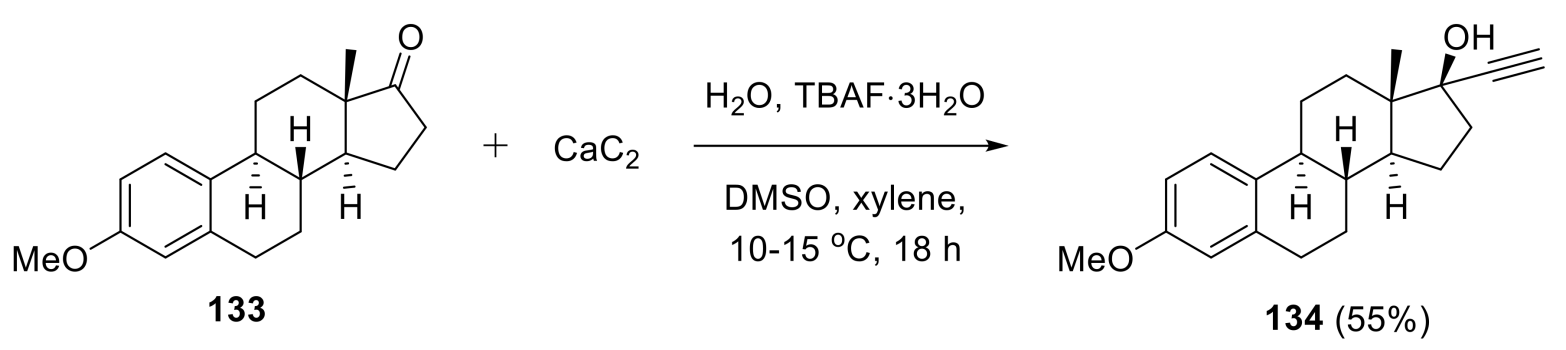
Synthesis of mestranol by Favorskii reaction.

Another prodrug of 17-ethynylestradiol, mestranol (**134**), was synthesized recently from the solid acetylene source, calcium carbide, and estrone 3-methyl ether (**133**) (Scheme 73) [119]. The presence of fluoride reportedly enhances the reaction. Such procedures are safer than the use of gaseous acetylene in the presence of metals and strong bases.

**Scheme 74:**
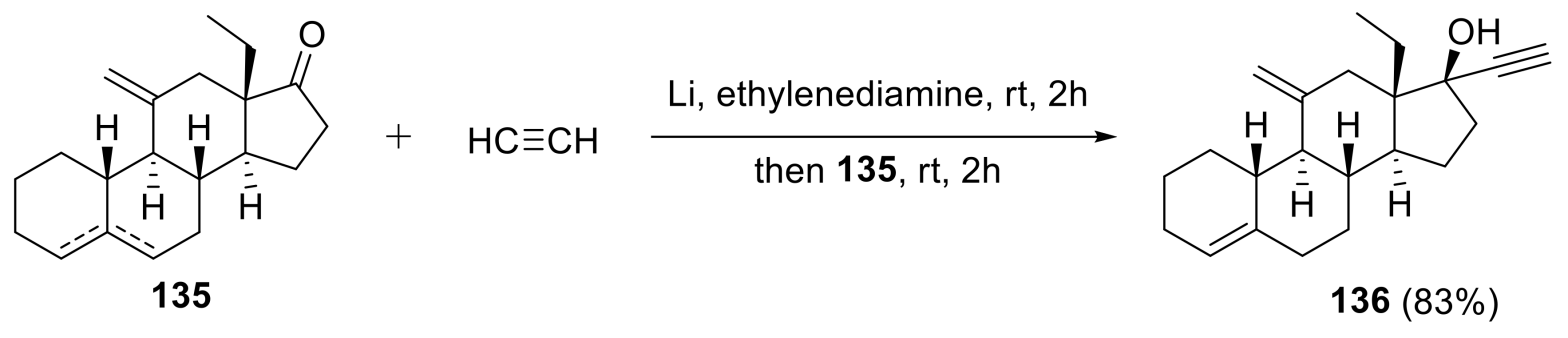
Synthesis of desogestrel.

A similar synthetic procedure allows obtaining desogestrel, a highly potent oral contraceptive medication (**136**, Scheme 74). Subsequential reaction of acetylene with lithium in ethylenediamine and the isomeric starting material **135** gave **136** in 83% yield [164].

**Scheme 75:**
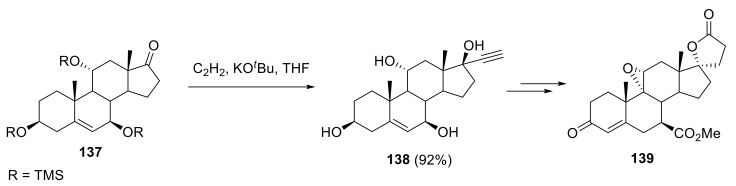
Eplerenone precursor synthesis by Favorskii reaction.

Eplerenone, which is used in the treatment of congestive heart failure and hypertension, can be synthesized by Favorskii reaction. The key step in its synthesis is provided by a reaction of acetylene with TMS-protected precursor **137** (Scheme 75). This step gave an acetylene-derived alcohol **138**, which was successively converted into eplerenone **139** [165].

Favorskii reactions with acetylene, acetylenides, or calcium carbide provide a neat and effective means for the synthesis of ethynyl-substituted steroids [159,160,166]. The approach allows obtaining a wide range of active pharmaceutical ingredients for hormone medications, anti-rheumatic and other anti-inflammatory drugs, medications for prevention and treatment of heart disorders, and many more [167].

### 2.6. Organometallic Complexes of Acetylene

Transition metal alkynyl complexes are potent intermediates in supramolecular design and organometallic synthesis [168,169,170,171]. Their tunable nature makes them excellent candidate molecules in non-linear optics [172,173] and polymer chemistry [174,175], as antitumor drugs [176,177] and luminescent materials [178,179]. In most cases, acetylene is utilized as a bridge and possesses a high potential due to possibility of Sonogashira-like reactions.

**Scheme 76:**
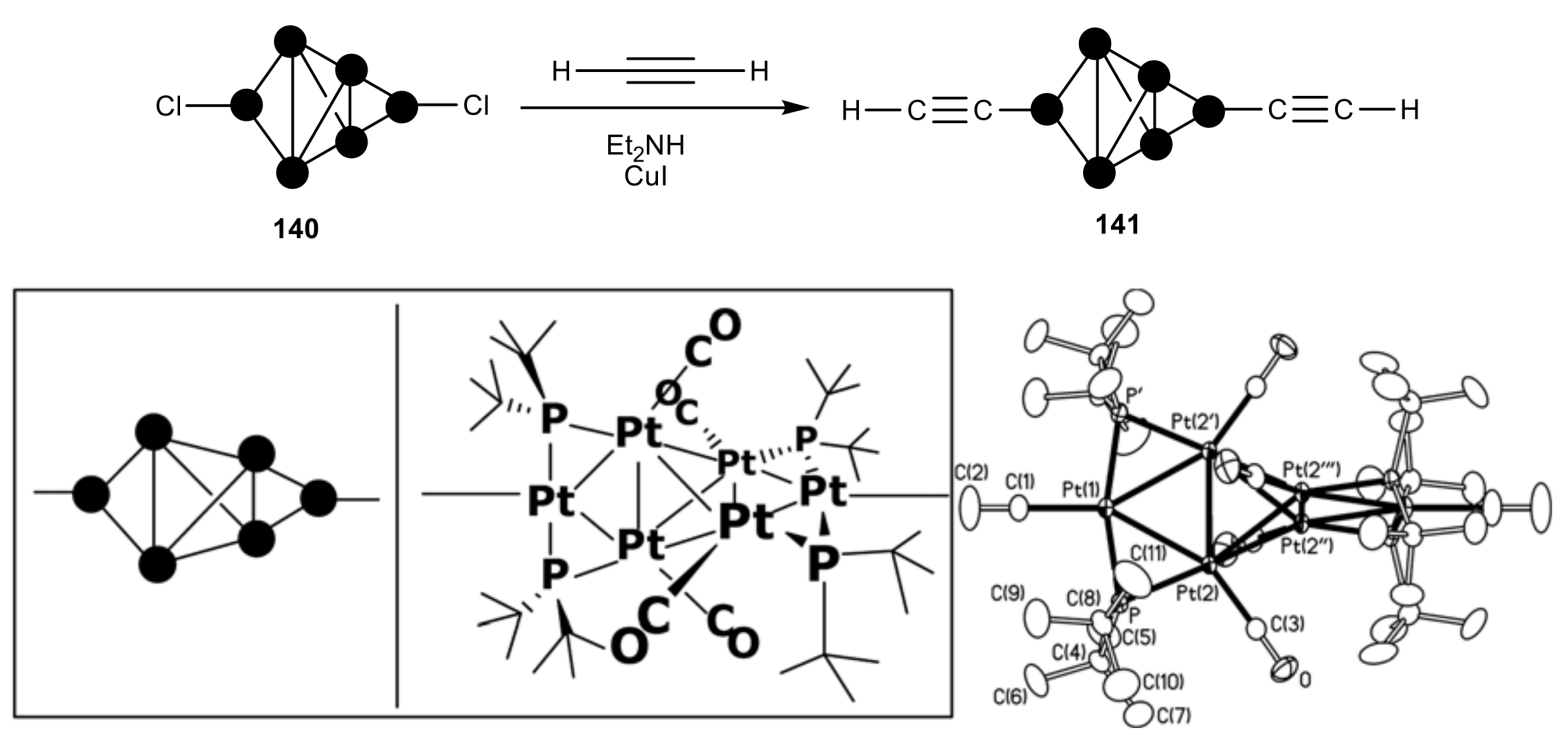
Synthesis and 3D representations of symmetrical bis-alkynyl derivative **141**. Hydrogen atoms are omitted for clarity. Thermal ellipsoids are set at 30% probability. ’ = 1 − x, 1 − y, z; ’’ = y, 1 − x, −z; ’’’ = 1 − y, x, −z. (Reproduced with permission from ref. [180]. Copyright 2016 the Royal Society of Chemistry.).

A symmetrical bis(alkynyl) derivative **141** is obtained essentially in compliance with the established Sonogashira mechanism (Scheme 76) [180]. The reaction is carried out under atmospheric acetylene pressure; it is initiated by mixing acetylene with metal cluster **140** in 2:1 molar ratio.

**Scheme 77:**
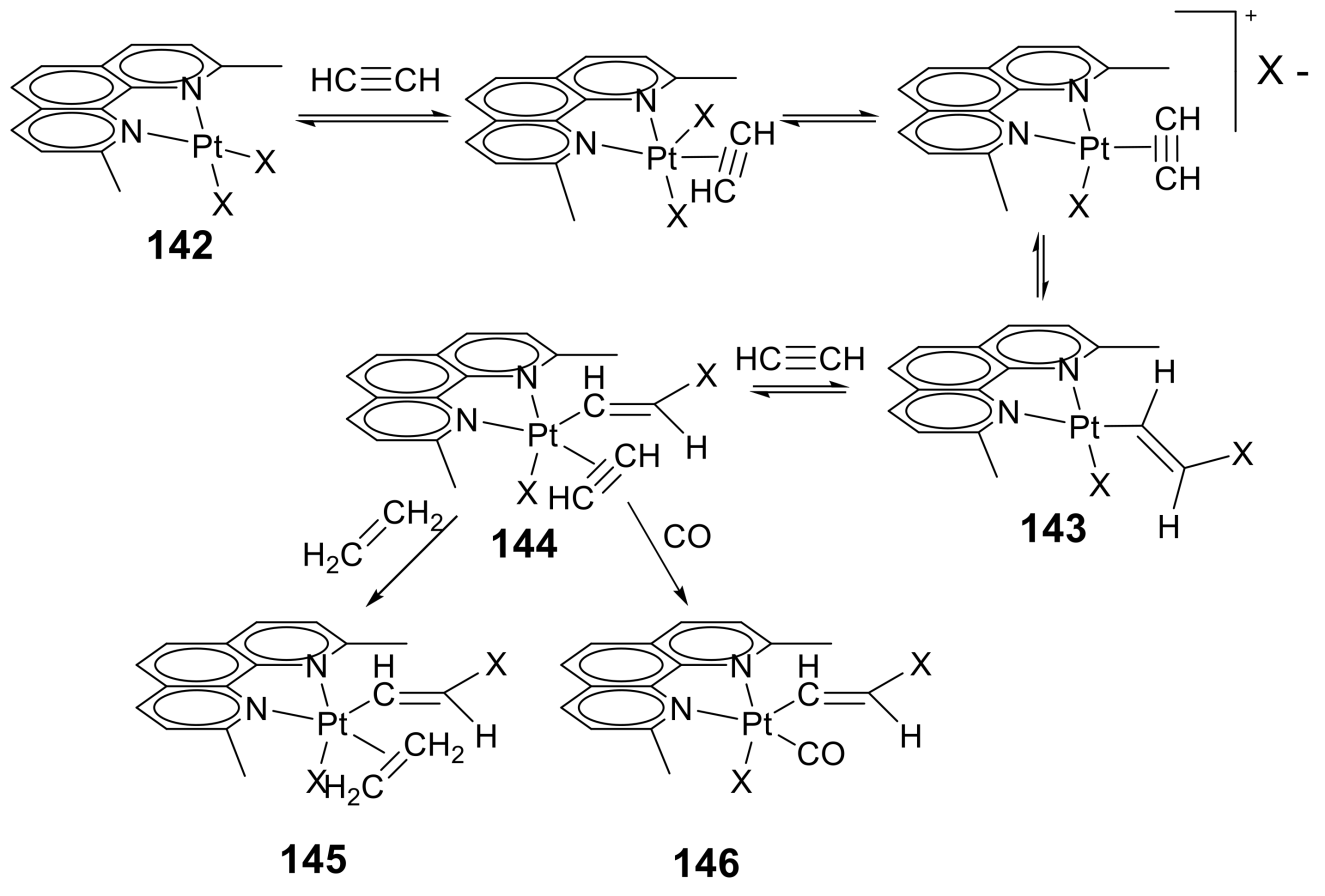
A mechanism proposed for the formation of acetylene-alkenyl complexes.

Insertion of acetylene into Pt-X bonds of square planar [PtX_2_(*N*^*N*)] complexes (X = Cl, Br, I), e.g., [PtX_2_(Me_2_phen)] complexes **142**, has been demonstrated [181]. Formation of Pt(II) five-coordinate comlexes is favored when X = bromide or iodide; with chloride, the complexes show negligible reactivity. The obtained five-coordinate complexes [PtX_2_(*η*^2^-CH≡CH)Me_2_phen)] **144** quickly relapse into four-coordinate alkenyl complexes [PtX_2_(*η*^1^-*E*-CH=CHX)Me_2_phen)] **143**. Excess of acetylene results in equilibrium between four- and five- coordinate complexes. Facile substitution of the *π*-bonded acetylene with free olefins or CO results, respectively, in alkene-alkenyl **145** or carbonyl-alkenyl **146** complexes (Scheme 77). In some cases, it is possible to isolate Pt-containing intermediates with acetylene to study kinetics and other properties [182]. 

**Scheme 78:**
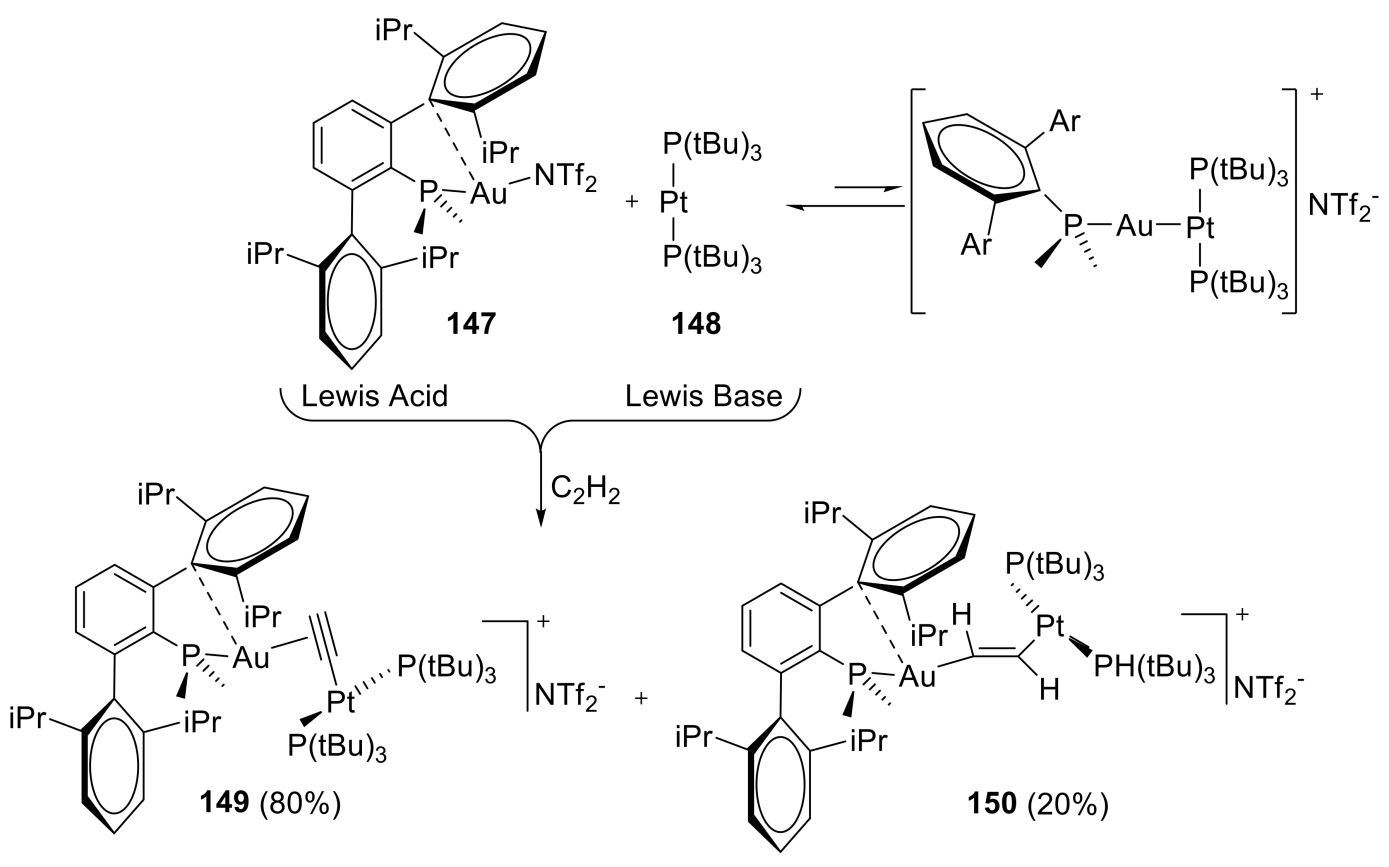
Reaction of Au and Pt complexes with acetylene.

An unusual heterobimetallic Au(I)/Pt(II) complex with acetylide (-C≡CH) bridge was formed in reaction between Au(I) and Pt(0) complexes **147** and **148** [183]. These complexes with bulky ligands terphenyl phosphine and tri-*tert*-butylphosphine, respectively, act as a Lewis acid and a Lewis base. The metal pair acquires a remarkable capacity of activating acetylene molecule. Upon exposure of benzene solutions of complexes **147** and **148** to acetylene their color instantly changes from bright yellow to intense orange. According to NMR data, the Au and Pt complexes were converted into two structurally distinct products (Scheme 78). In complex **149**, the triple bond bridge is *π*-bonded to a cationic Au(I) center and *σ*-bonded to a Pt(II) hydride fragment. In complex **150**, which lacks the triple bond, the metals are linked by a *μ*-ethene-1,2-diyl moiety. 

**Scheme 79:**
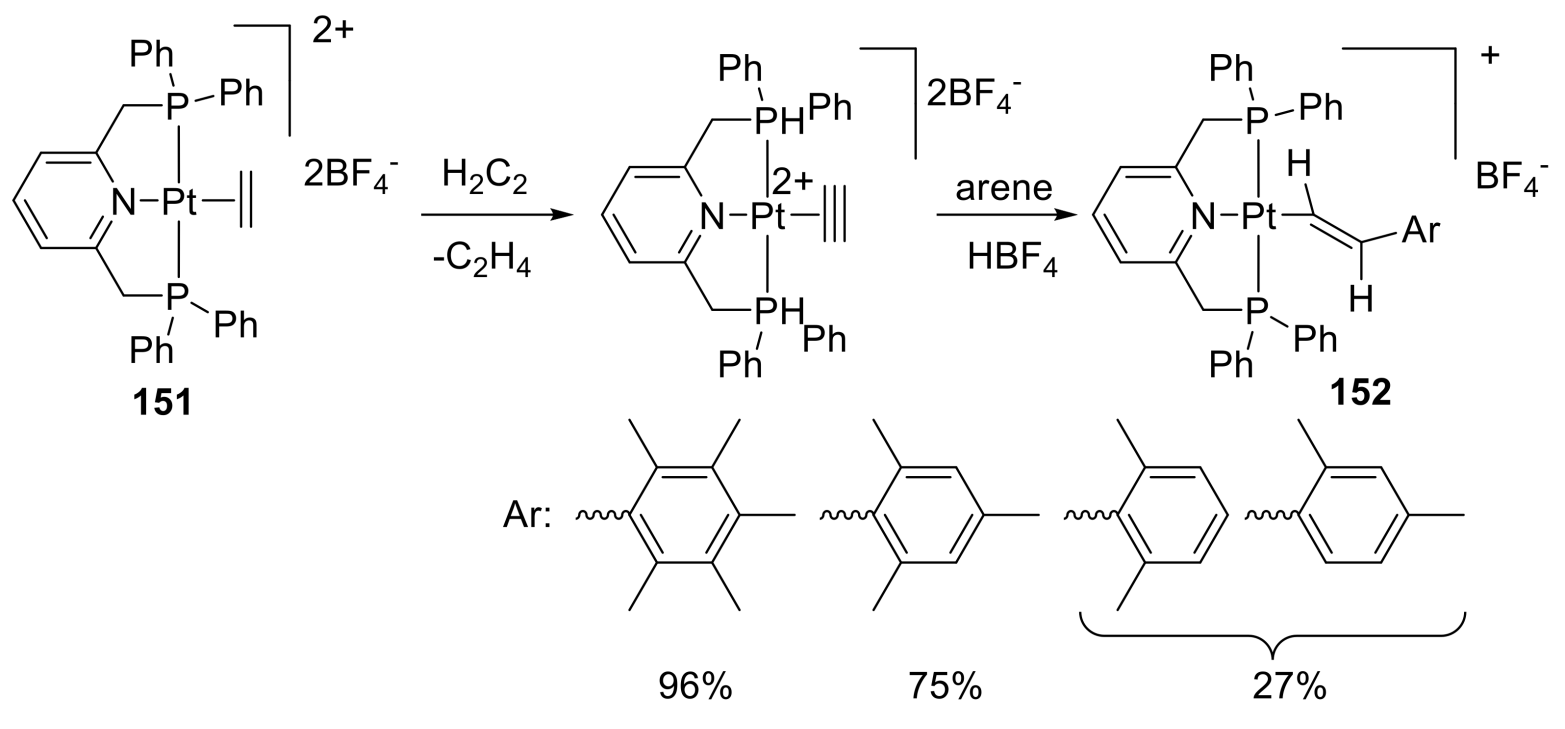
Pt-catalyzed selective hydroarylation.

Hydroarylation of alkynes provides a valuable route to various alkenes, although chemo-, regio and stereoselectivities of this reaction may be compromised by collateral formation of diaryl alkanes, dialkenyl arenes, and aryl dienes. An in situ generated dicationic complex [Pt(2,6-bis(dephinylphosphinomethyl)pyridine)C_2_H_2_](BF_4_)_2_ **151** reacts with arenes [184]. Only electron-rich arenes are incorporated into arylalkenyl products **152** (Scheme 79). Another interesting study of unsaturated Pt(II) complex showed tautomerization of acetylene to vinylidene [185]. In this case, C-C bond formation occurs via isomeric methyl vinylidene structure instead of the classical migratory insertion reactivity. Vinylidene formation is also characteristic of the iridium pyridylidenes 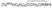 (R = H, Me, Ph) reaction with acetylene [186] The reaction afforded four-membered iridacycles with IrC(=CH_2_)N moiety. 

**Scheme 80:**
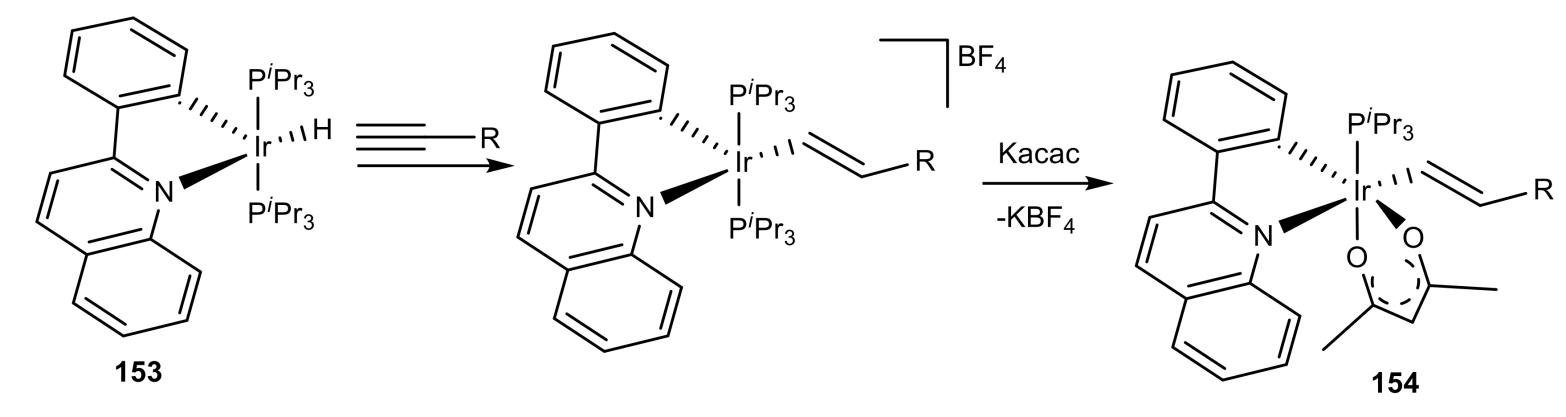
Preparation of Ir-based vinyl and styryl compounds.

Insertion into Ir-H bond is also possible. Acetylene and terminal alkynes react with Ir complex **153** to produce five-coordinate styryl derivatives **154** (Scheme 80). The short-lived yellow-orange six-coordinate vinyl complexes are emissive upon photoexcitation [187]. 

**Scheme 81:**
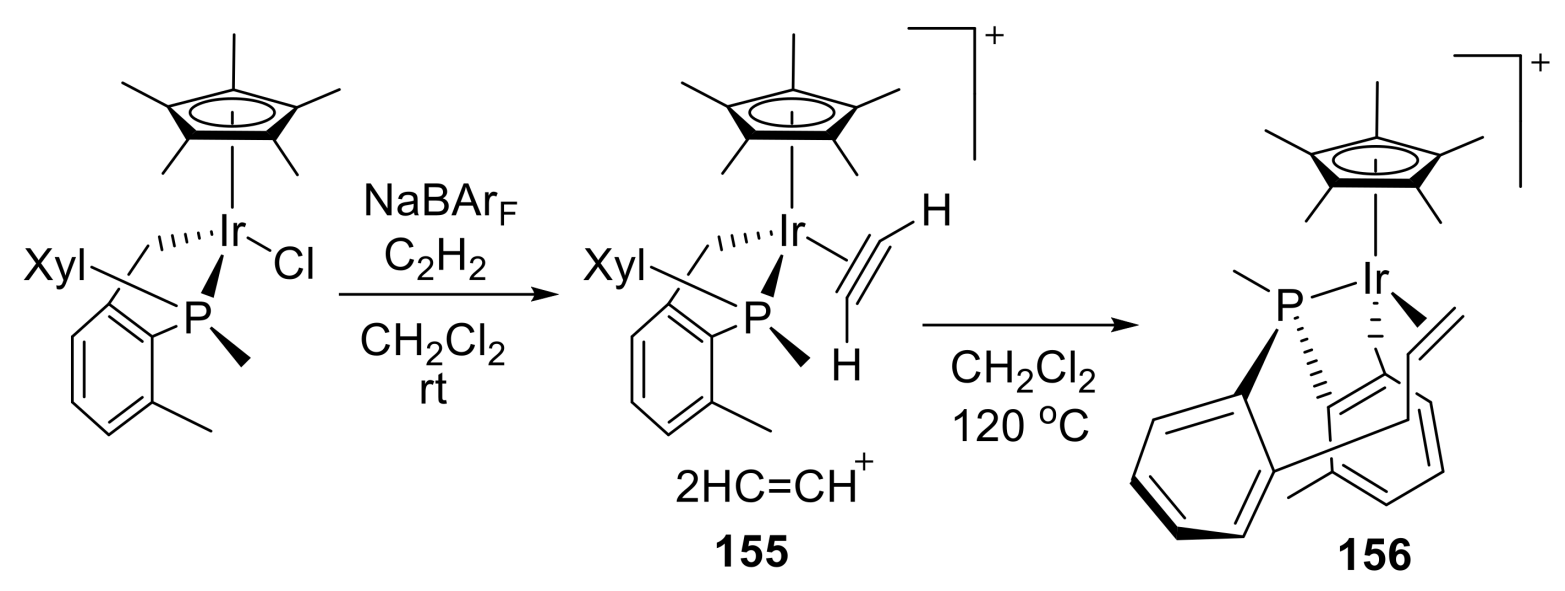
Synthesis of [(*η*^5^-C_5_Me_5_)Ir(C^P)]^+^ acetylene adduct and its thermal transformation.

An electrophilic cationic complex [(*η*^5^-C_5_Me_5_)Ir(C^P)]^+^ reacted with acetylene at room temperature to form the [(*η*^5^-C_5_Me_5_)Ir(C^P)(*η*^2^-acetylene)]^+^ *π* adducts **155** [188]. The C-C bond formation led to an iridium-bound alkene moiety as a thermodynamic product, whereas similar complexes with bulkier alkynes underwent further transformation into allylic structures. NMR experiments with D-labeled compounds implicated an undetected vinylidene structure, Ir=C=C(H)Ph, in the key C-C bond formation. Heating of complex **155** solution in a pressure vessel afforded an extraordinary thermostable complex **156** (Scheme 81).

**Scheme 82:**
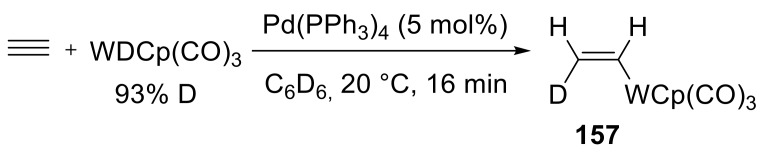
Acetylene insertion into deuterated tungsten complex.

Selective insertion of alkynes into Mo-H and W-H bonds was catalyzed by Pd(0) complex and resulted in alkenylmolybdenum and alkenyltungsten complexes [189]. Interestingly, no incorporation of deuterium into *trans* and geminal positions of vinyltungsten complex **157** was observed in the reaction of acetylene with tungsten complex WDCp(CO)_3_ (Scheme 82). Tungsten complexes with diphosphines [Tp*W(CO)(I){*η*^2^-C_2_(PPh_2_)_2_}] (Tp* = hydrido-tris(3,4,5-trimethyl-pyrazolyl)borate) showed highly reversible redox potentials [190]. A variety of substituted bisphosphine acetylene bridged dinuclear complexes with a side-on bound Tp*W(CO)(I) moiety and a chelate-like coordinated PtCl_2_ unit was reported [191].

Thus, promotion of acetylene insertion by metal atoms results in modified metal complexes, which can be isolated. Various metal complexes are used as catalysts or building blocks and also, notably, in luminescence applications. Here we descried a few representative examples of reactions with organometallic complexes, where a number of other possibilities are available as well as other terminal alkynes react with metal complexes in a similar manner [192].

### 2.7. Miscellaneous Synthetic Transformations

**Scheme 83:**
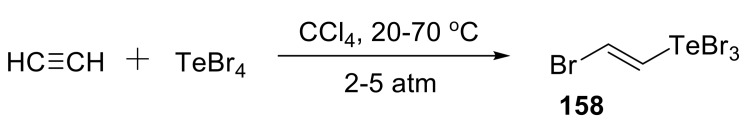
Synthesis of (*E*)-2-bromovinyltellurium tribromide.

Other applications of acetylene involve addition, cycloaddition, and polymerization reactions. A reaction of tellurium tetrabromide with acetylene in tetrachloromethane provides stereoselective synthesis of (*E*)-2-bromovinyltellurium tribromide **158** (Scheme 83) [193].

Compounds with 2-bromovinyltellanyl group are used in stereoselective syntheses of alkenes via consecutive replacement of bromine and tellurium atoms by organic groups in cross-coupling reactions [194].

**Scheme 84:**
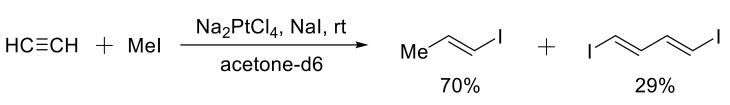
Pt(IV)-catalyzed addition of iodomethane to acetylene.

The reaction of iodomethane and acetylene in the presence of Na_2_PtCl_4_ and NaI yielded a mixture of iodoethylenes (Scheme 84) [195]. A mechanism proposed by the authors involved intermediate formation of a Pt(IV) methyl vinyl derivative by iodoplatination of acetylene with a reversibly formed Pt(IV) methyl complex.

**Scheme 85:**
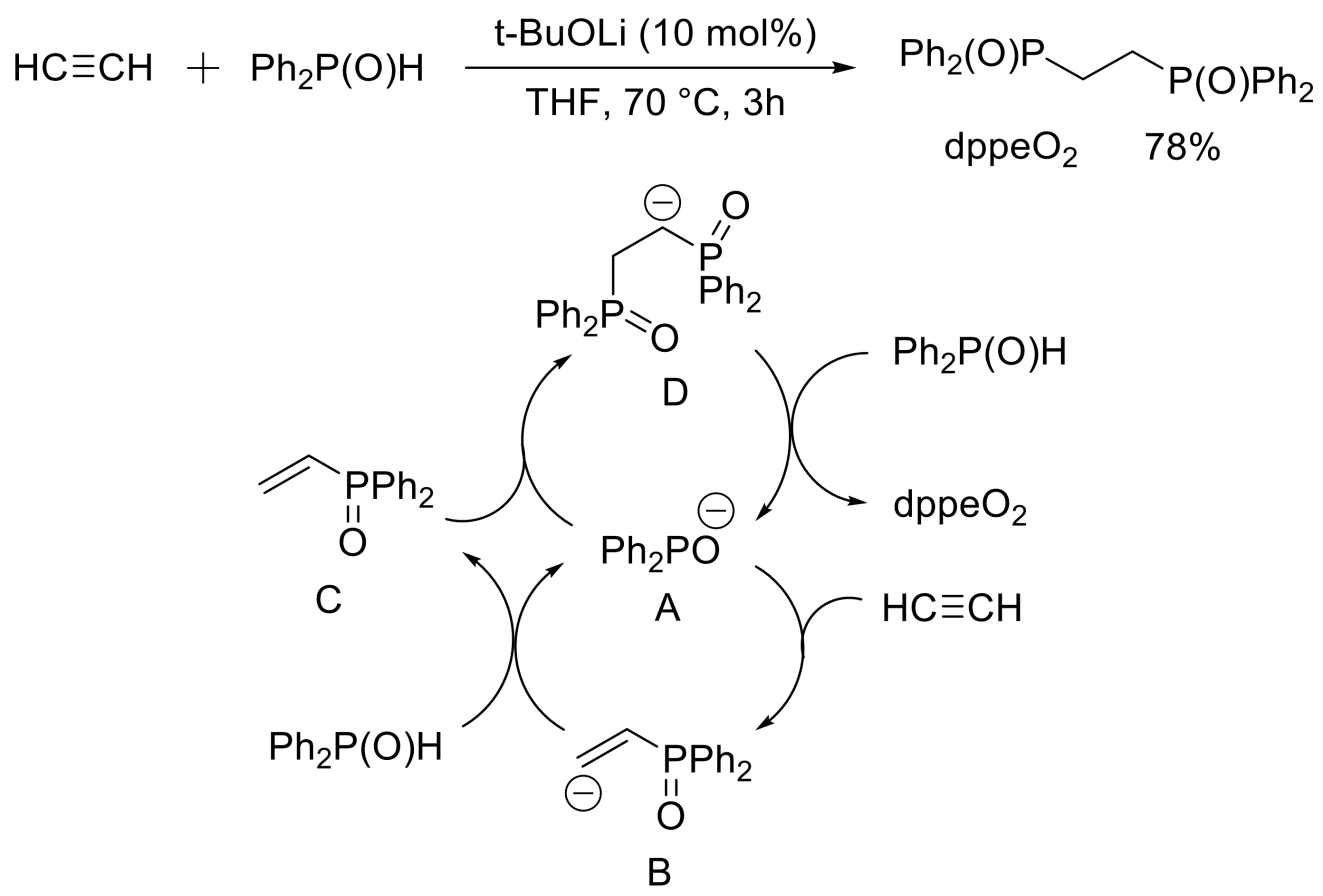
A mechanism proposed for the formation of dppe oxide.

Another example of catalytic addition to acetylene, preparation of 1,2-bis(diphenylphosphino)ethane dioxide (dppeO_2_), proceeded in the presence of *t*-BuOLi (Scheme 85) [196]. A possible mechanism for this reaction includes deprotonation of Ph_2_P(O)H by *t*-BuOLi. The resulting formation of anion **A** directs the catalytic cycle toward dppeO_2_.

**Scheme 86:**
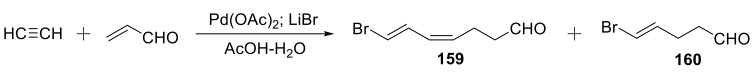
Pt(II)-catalyzed addition of acrolein to acetylene in the presence of LiBr.

**Scheme 87:**
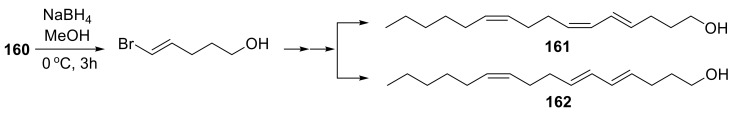
Synthesis of (4*E*,6*Z*,10*Z*)-hexadeca-4,6,10-trien-1-ol **161** and (4*E*,6*E*,10*Z*)-hexadeca-4,6,10-trien-1-ol **162**.

A tandem addition reaction of acrolein with acetylene to form (*E*)-5-bromopent-4-enal **160** involved Pd(OAc)_2_ as a catalyst (Scheme 86) [197]. The best yields (73%) are obtained under optimized conditions of 1.5/1 AcOH/H_2_O molar ratio, C_2_H_2_ pressure of 1.2 atm for 72 h at 25 °C, with 1 mmol% Pd(OAc)_2_, 1 mol% LiBr, and 1.2 mol% acrolein.

(*E*)-5-Bromopent-4-enal **160** provides starting material for synthesis of (4*E*,6*Z*,10*Z*)-hexadeca-4,6,10-trien-1-ol **161** and (4*E*,6*E*,10*Z*)-hexadeca-4,6,10-trien-1-ol **162** [197], the active pheromone components of cocoa pod borer moth (*Conopomorpha cramerella*), the most injurious pest that damages cocoa plantations in Southeast Asia (Scheme 87) [198].

**Scheme 88:**
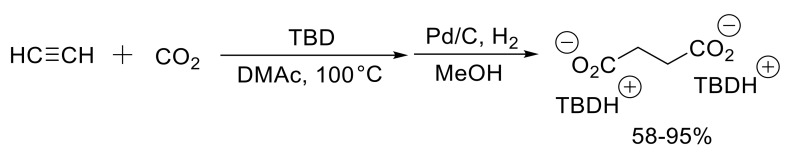
Acetylene carboxylation in the presence of TBD.

Direct carboxylation of acetylene with CO_2_ in the presence of 1,5,7-triazabicyclo[4.4.0]dec-1-ene (TBD) led to acetylene dicarboxylic acid derivatives (Scheme 88) [199].

**Scheme 89:**
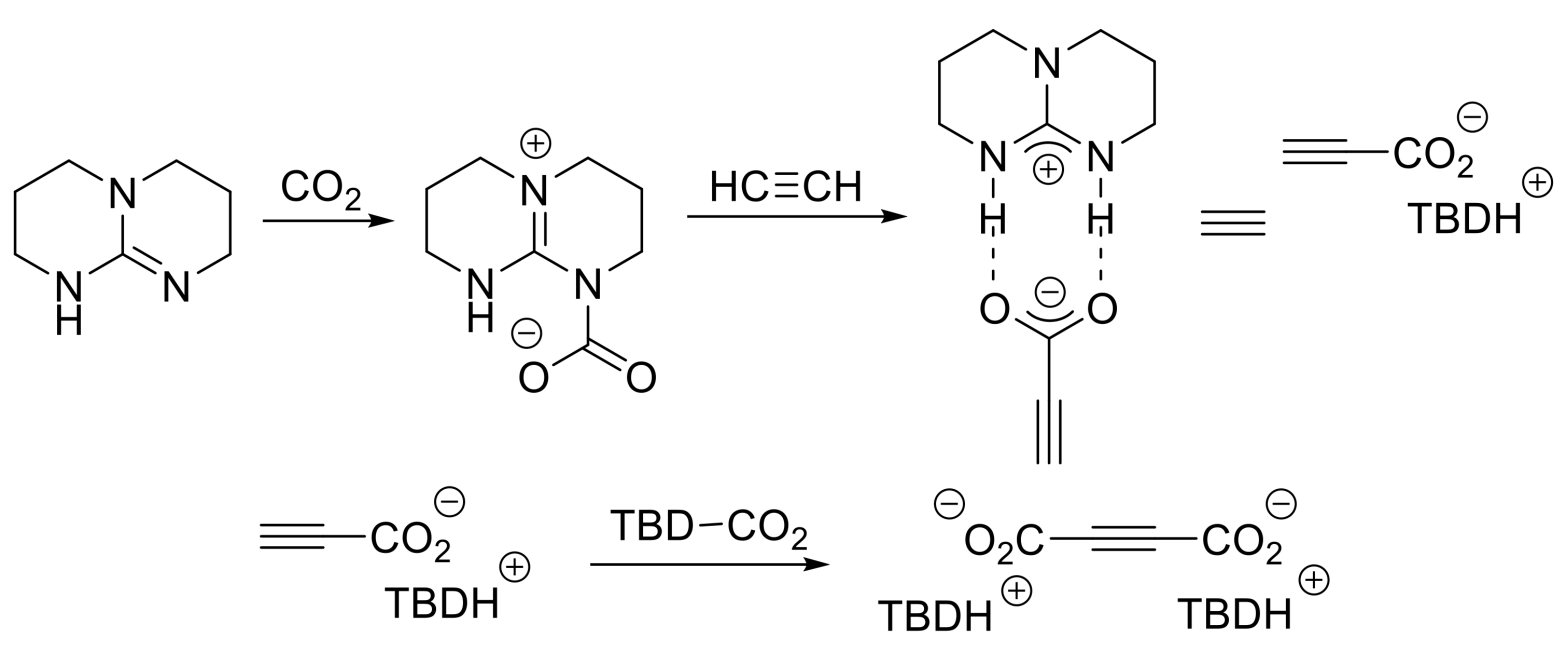
A mechanism proposed for the TBD-promoted formation of acetylene dicarboxylic acid derivatives.

A possible mechanism for this reaction is shown in Scheme 89. The carboxylation is initiated by formation of a TBD-CO_2_ adduct, which undergoes nucleophilic addition of acetylene to afford a propiolate-TBD salt.

**Scheme 90:**
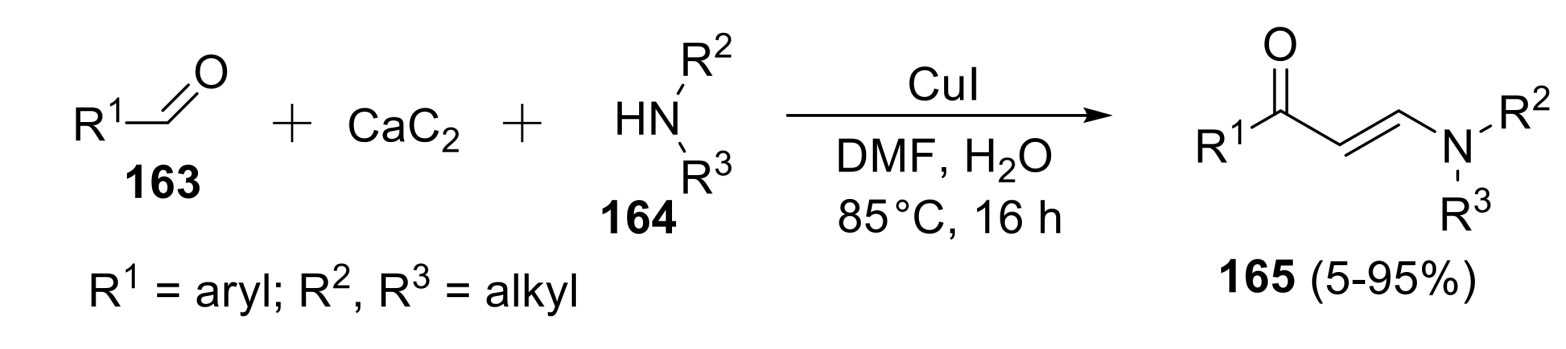
Three component synthesis of enaminones.

Enaminones **165** can be obtained by a three component reaction of calcium carbide, aryl aldehydes **163**, and amines **164** (Scheme 90) [200].

**Scheme 91:**
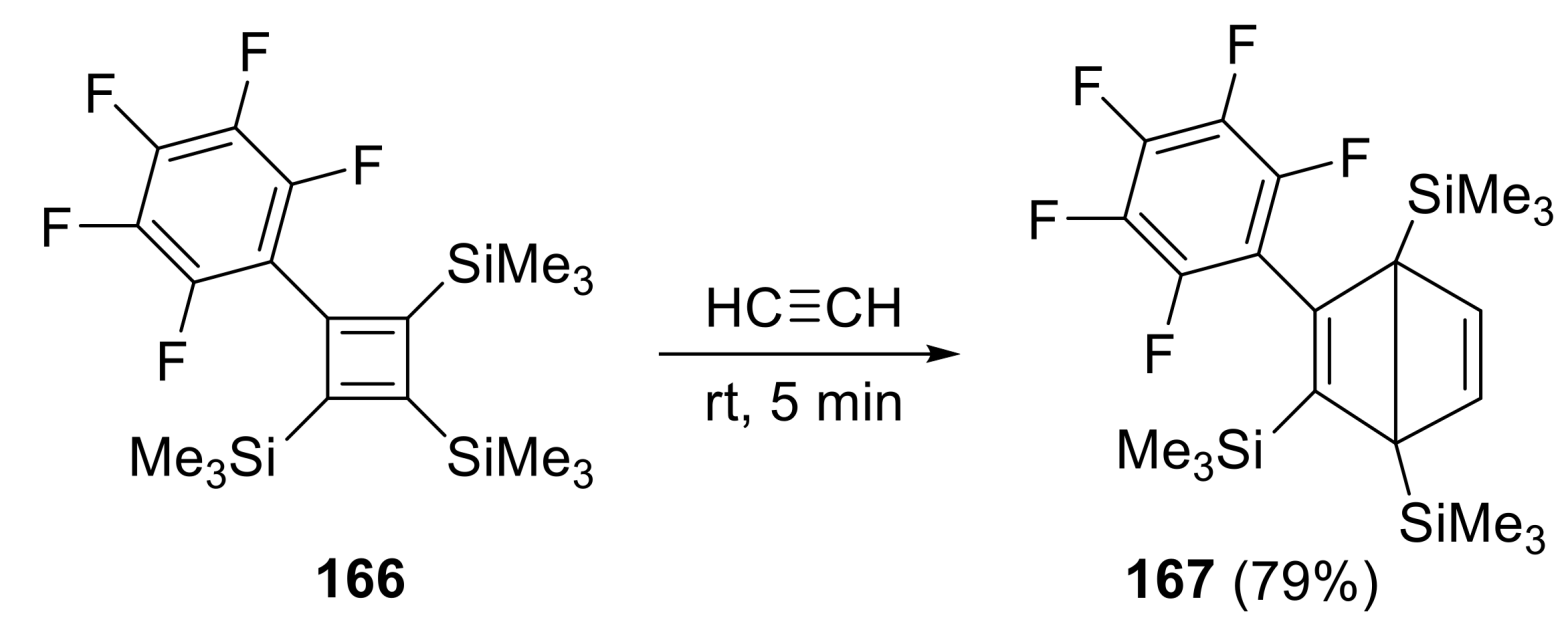
[2 + 2]-Cycloaddition of acetylene to pentafluorophenyltris(trimethylsilyl)cyclobutadiene.

Another interesting example of acetylene addition is provided by a non-classical Diels-Alder reaction with substituted cyclobutadiene **166** [201]. The dried acetylene is bubbled into a toluene solution of pentafluorophenyltris(trimethylsilyl)cyclobutadiene to afford the Dewar benzene-like structure **167** (Scheme 91).

**Scheme 92:**
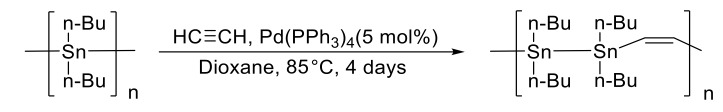
Synthesis of polystannanes with –CH=CH– link.

A catalytic insertion of acetylene into polystannane (Scheme 92) [202] provides an interesting link with the chemistry of polystannanes, the unique conductive main group polymers with a backbone composed entirely of tin [203].

### 2.8. Selective Semi-Hydrogenation of Acetylene

**Scheme 93:**
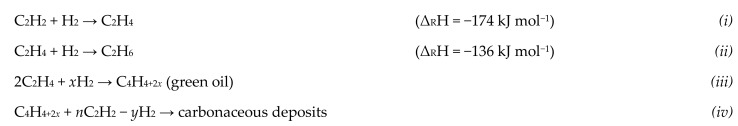
Reaction possibilities of acetylene hydrogenation.

Naphtha cracking generates streams of ethylene, which may be directly used in polymerization or partial oxidation to ethylene oxide. Acetylene impurities constitute 0.5–2 vol% of the primary ethylene streams. Their amount should be diminished to 5 vol% prior to polymerization. Acetylene, which is poisonous to the downstream polymerization catalysts, is removed from the gas mixture by hydrogenation. This step involves hydrogenation of acetylene to ethylene (*i*), hydrogenation of ethylene to ethane (*ii*), acetylene polymerization into green oil (*iii*), and carbonaceous deposition (*iv*, Scheme 93). The aim of the purification step is to achieve (*i*) avoiding (*ii*), (*iii*), and (*iv*).

Selective hydrogenation of acetylene requires *π*-bonding of its molecules to the surface of a catalyst. The process is hindered when the adsorption centers are placed too densely; its performance can be enhanced by the active-site-isolation approach. As for the selectivity, all improvements fall into three groups. The first type of improvements results from modification of electronic properties of the catalyst is caused by alloying and/or support influence. The ligand effect [204] is caused by interaction of two metals and results in shift of their d-band center, which affects the adsorption strength and, the activation energy. Concomitant changes in structure and composition of the surface have modulatory effect on the number and geometry of the adsorption sites (the so-called *ensemble effect*). The emerging A sites are mainly responsible for selective acetylene hydrogenation, whereas the larger Pd ensembles, E sites, favor the ethylene-to-ethane conversion. The second type of selectivity improvements results from modification of the active site geometry and morphology. The third type of improvements is achieved by dilution or blocking of large active sites caused by reducing, deposition technique and ligands addition. 

The catalysts used for selective acetylene hydrogenation can be divided into Pd-based (Pd/Ga [29,30], Pd/Ag [31,32,33], Pd/Cu [205]) and Pd-free, further subdivided into monometallic (Cu [206], Ag [207], Fe [208], Ni [209,210], Au [211]), bimetallic (Au/Ni [212,213], Au/Ag [214], Ni/Zn [8], Cu/Al hydrotalcite [215]), tri-metallic (Cu-Ni-Fe [216]), and metal free (CeO_2_ [217,218]). Today, Pd-based catalysts are often used for selective removal of acetylene [219,220]. However, they are prone to contamination with Pd carbides [221,222,223] and Pd hydrides [31,224], which worsen the performance and lead to over-hydrogenation [225,226]. Addition of carbon monoxide improves the selectivity, because CO competes for adsorption sites with ethylene but not acetylene [32,227]. However, CO co-feeding is inconvenient, as it requires continuous control of CO amounts in order to maintain the full acetylene conversion. In addition, CO promotes the formation of so-called green oil, which is composed of acetylene oligomers [210]. Although the activity of pure Pd is high, its selectivity is limited to 20% at 200 °C [228]. The optimum temperatures for such catalysts are much higher than those accepted in industry; besides, they are really expensive. Addition of the second metal improves the selectivity for two reasons: by changing electronic properties of Pd and by dilution of adsorption sites and thereby reducing the number of E sites. 

An alternative approach is based on addition of selective surface modifiers, that is, ligands with specific affinity to Pd surface. The ligands can be used during both liquid and gas phase hydrogenation. The nitrogen- [229,230], sulfur- [231,232], and phosphorus-containing [233,234] modifiers of this type usually enhance the selectivity by adsorption to the metal surface, which causes the decreasing the number of large Pd ensembles sites. 

The third approach involves reducible oxide support modifications and deposition technique. TiO_2_, La_2_O_3_, Nb_2_O_5_, and CeO_2_ migrate onto the Pd surface during reduction, diluting the multiply coordinated sites and enriching the electron density of Pd [205,235,236,237] Dispersion across the surface of graphene [238] and insertion into zeolite frameworks [239] may also be considered as support modifications.

**Figure 5:**
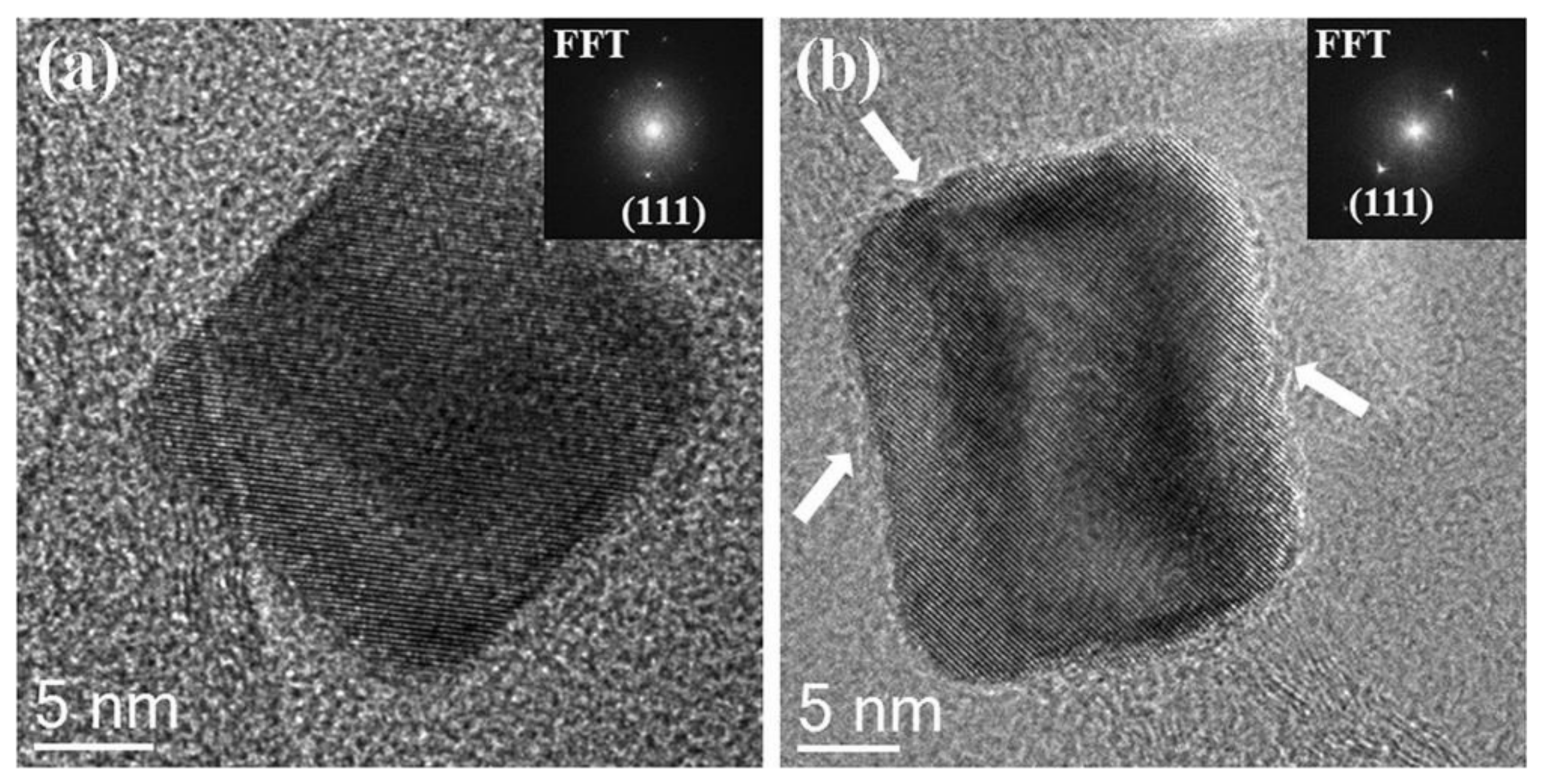
HR TEM images of palladium NPs after 2 h (**a**) and 55 h (**b**) of the reaction. White arrows indicate the sunken regions with steps and terraces. (Reproduced with permission from ref. [248]. Copyright 2017 WILEY-VCH Verlag GmbH & Co. KGaA, Weinheim.).

When pure palladium catalyzes acetylene hydrogenation, conversion and selectivity of the reaction depend on size and morphology of the metal particles. Acetylene hydrogenation is sensitive to the surface structure of Pd NPs. The most selective facet for acetylene hydrogenation is Pd(100). Cubic palladium nanoparticles (NPs) (100) facets can be prepared under controlled conditions [240,241,242,243,244]. Quite unluckily, the low-index facets of Pd [245,246] and Pt [247] are unstable and changed under the reaction conditions. After the reaction, the flat facets turn into sunken facets, and the cubic shape becomes twisted (Figure 5). The selectivity is maintained high for 27 h of the reaction and then sharply decreases [248]. Microstructural changes on the surface of the well-defined (100) facets result in rough (100) facets with steps and corners. These changes are induced by multiple repetitive adsorptions-desorptions. 

**Figure 6:**
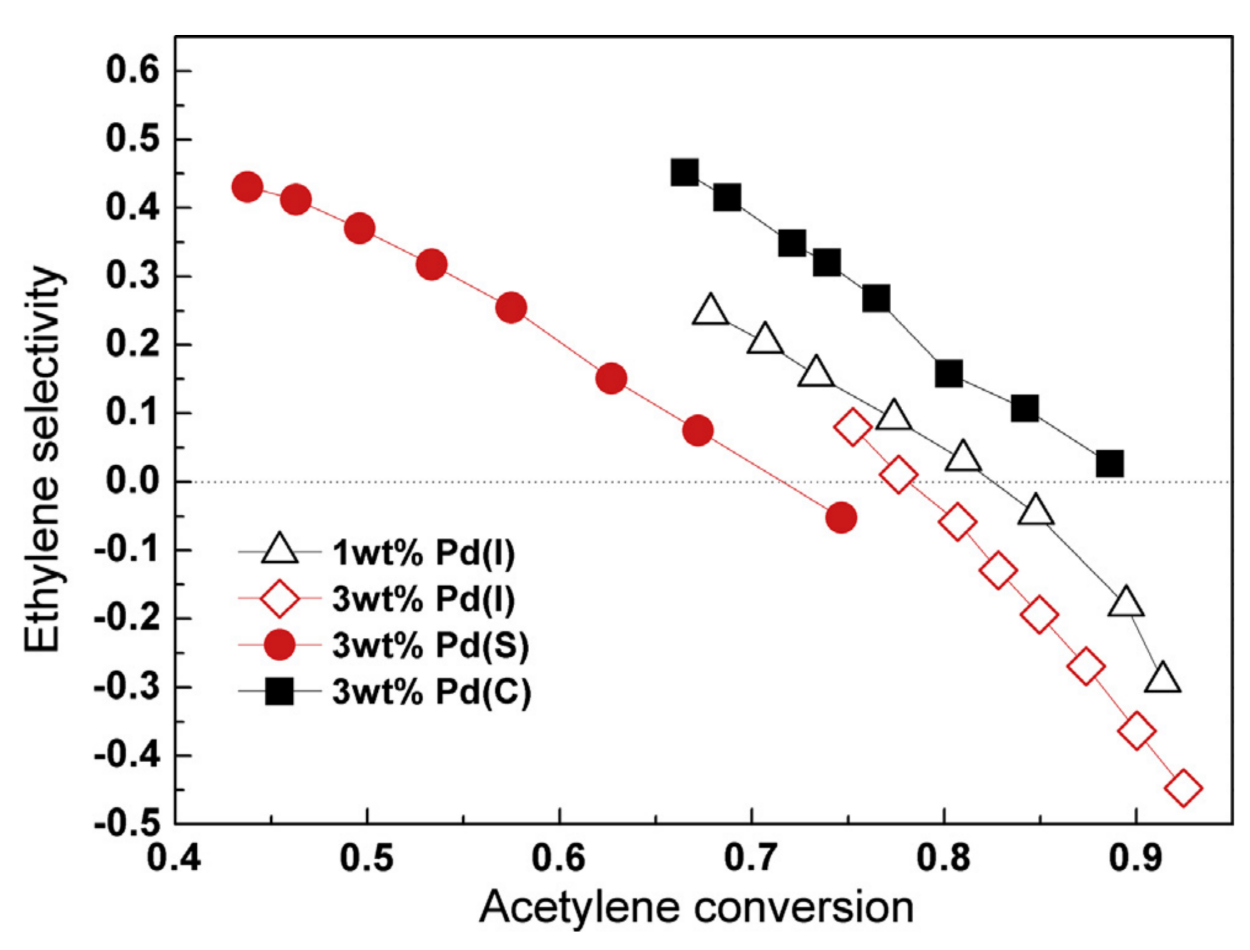
Ethylene selectivity as a function of acetylene conversion. Pd(I)-catalysts without defined morphology prepared by using the incipient wetness impregnation; Pd(S)-catalyst with spherical particles; Pd(C)-catalyst with cubic particles. Negative ethylene selectivity indicates net loss of ethylene due to over-hydrogenation and ethane production (reproduced with permission from [249]. Copyright 2013, Elsevier Inc.).

Cubic NPs with Pd(100) facets show better results than spherical NPs of a similar size, which comprise significant portion of Pd(111) facets [249]. The temperatures of Pd hydride decomposition and hydrocarbon desorption for the cubic NPs are lower than for the spherical NPs. They also show higher acetylene conversion rates and better ethylene selectivity than Pd(222)-faceted spherical particles (Figure 6) or smaller Pd(I) NPs prepared by using the incipient wetness method. 

**Figure 7:**
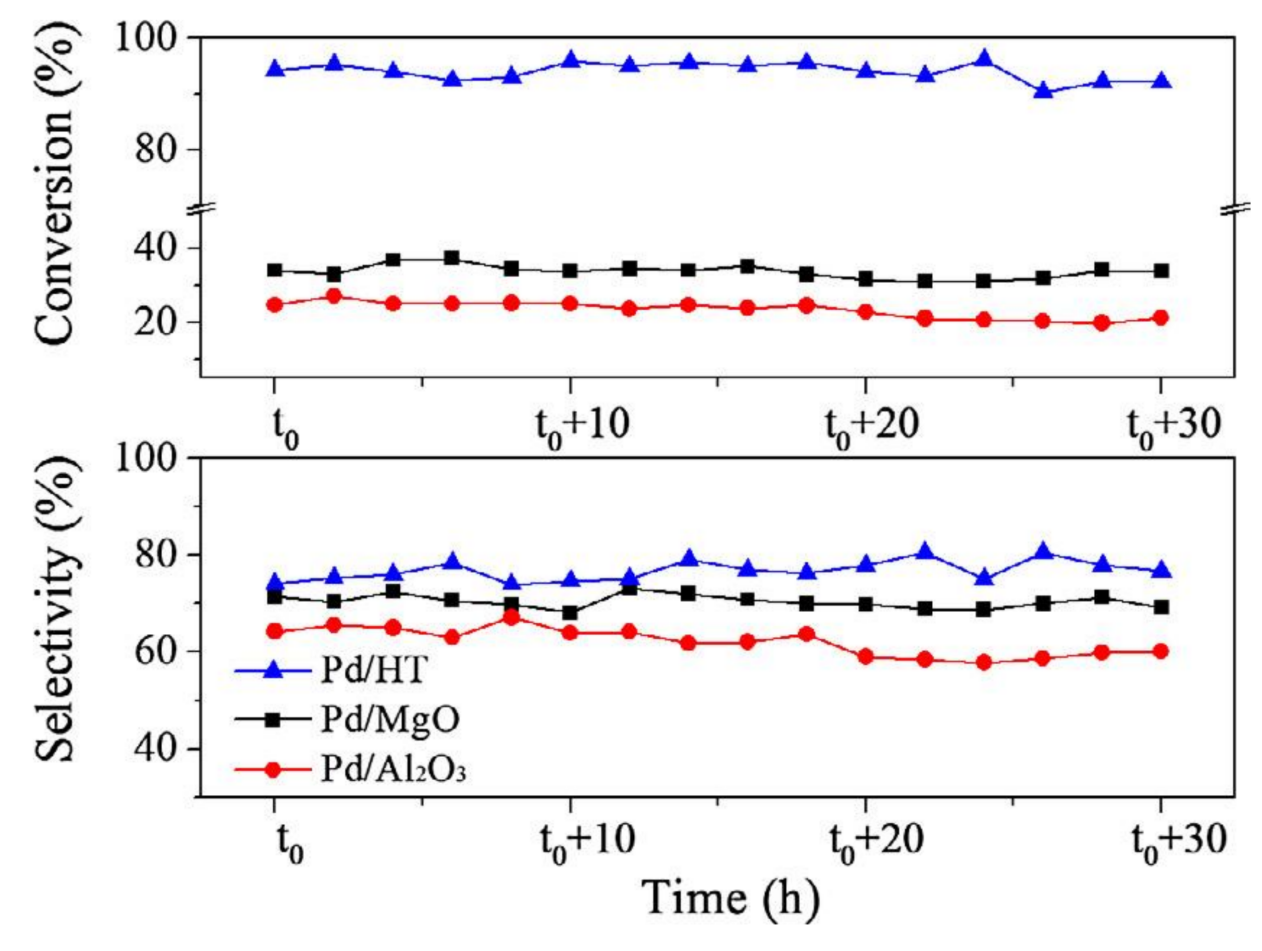
Plots of acetylene conversion and ethylene selectivity for Pd on different supports. (reproduced with permission from [250], Copyright 2015, Elsevier Inc.).

Surface acidity and basicity effect on the status of metals and on catalytic performance. Palladium supported hydrotalcite (Pd/HT) exhibits higher conversion of acetylene compared with aluminum oxide and magnesium oxide supports [250]. HT is a synthetic anionic clay, [M^2+^*_1-x_*M^3+^*_x_*(OH)_2_]*^x^*^+^(A*^n-^*)*_x/n_*·*m*H_2_O, where M stands for metal cations and A represents an interlayer anion. After heat treatment, the interlayer anion and water are removed, leaving a mixture of metal oxides, which determines the acidity/basicity of the surface. Full conversion of acetylene over Pd/HT is reached at 55 °C (Pd/MgO and Pd/Al_2_O_3_ are left behind with 31% and 22%, respectively, Figure 7). Ethylene selectivity of the system is 88% at 80% isoconversion, which is respectively 1.8 and 2.1 times higher than for Pd/MgO and Pd/Al_2_O_3_. It is well known that changing the support affects the parameters of catalytic reduction. Atomic Pd samples deposited on nanocarbons of differential morphology (representing different proportions of nanotubes, nanofibers, and other nanocarbon species) show differential conversions and selectivities [251]. 

**Figure 8:**
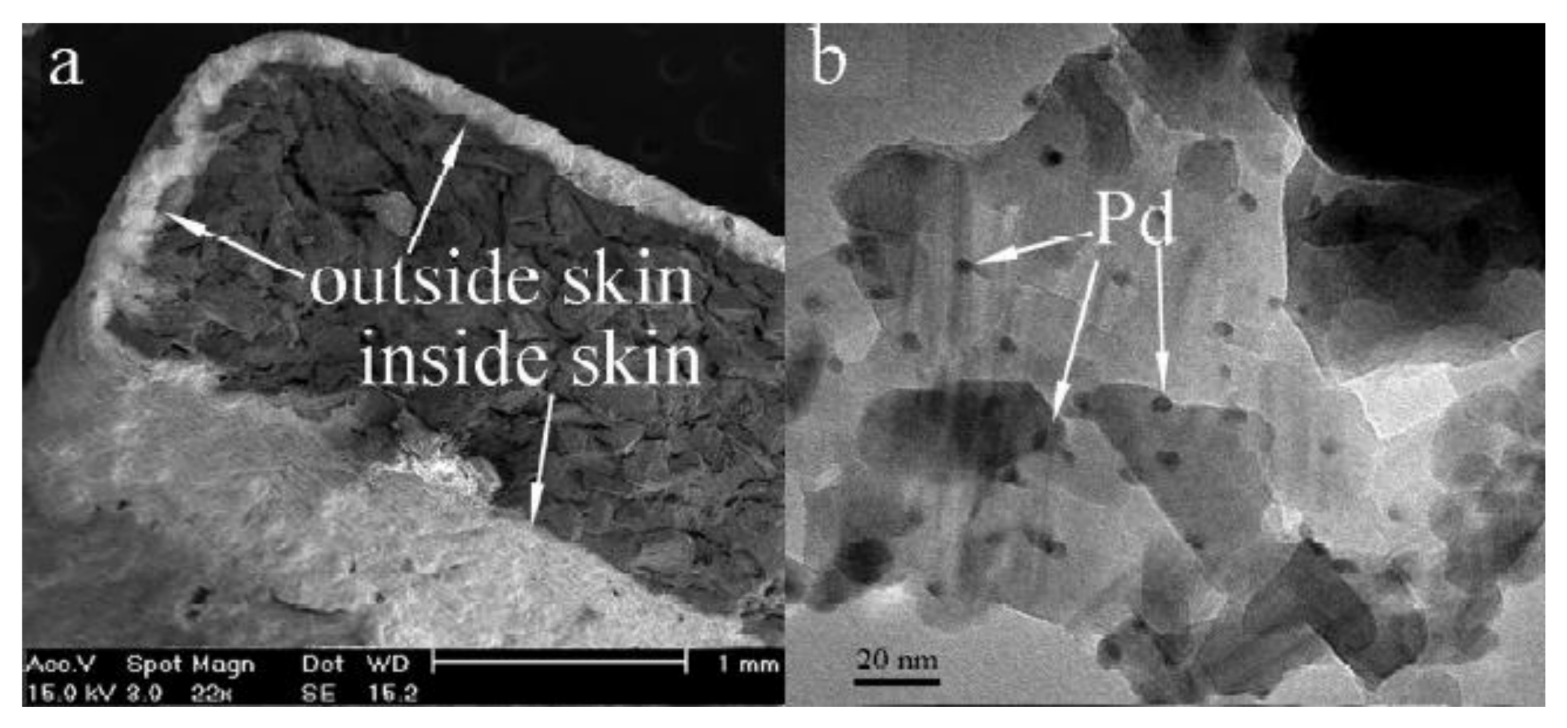
(**a**) SEM image of Pd/α-Al_2_O_3_@SiC catalyst, (**b**) TEM image of Pd particles on Al_2_O_3_ layer of Pd/α-Al_2_O_3_@SiC catalyst (reproduced with permission from [252]. Copyright 2016, the Royal Society of Chemistry).

Pd is usually present as a skin on the surface of a support layer. Dispersion of Pd active sites over the skin favors acetylene hydrogenation and inhibits over-hydrogenation and oligomer formation. Tunable thickness of a support layer allows effective control of Pd penetration range and results in uniform lateral distribution of Pd active sites. Egg-shell Pd/α-Al_2_O_3_@SiC catalysts with the controlled thickness of support layer (50–300 μm, Figure 8) were obtained by a specifically developed procedure and tested in acetylene hydrogenation [252]. The catalysts had the same acetylene conversion but higher ethylene selectivity as compared with the conventional Pd-Ag/α-Al_2_O_3_ catalyst. 

**Figure 9:**
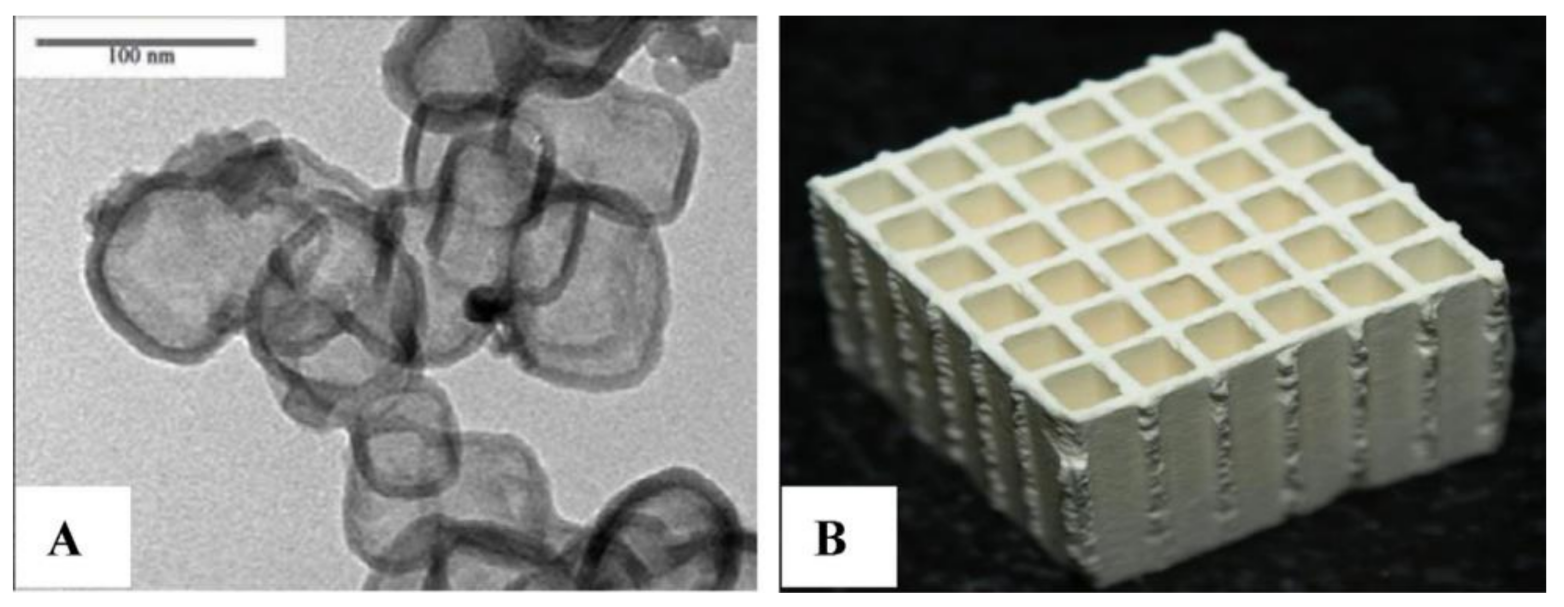
Porous hollow silica NPs (**A**) and monolithic cordierite (**B**) (reproduced with permission from [253]. Copyright 2013, Elsevier Inc.).

A more than 10 times increase in surface area can be achieved by using porous hollow silica NPs as a coating in combination with monolithic cordierite as a ceramic substrate (Figure 9) [253]. Catalytic activity of the samples is enhanced by increasing reaction temperature, although by the cost of ethylene selectivity. The best results among the series are achieved for PdO/PHSNs-Al_2_O_3_/MC with 100% acetylene conversion and 47% ethylene selectivity. This study is an example of successful development of a new monolithic catalyst with increased surface area and maintainable mesoporous structure of the coating layer.

Ionic liquids stabilize nanoscale structures by forming the protective anionic and cationic layers [254,255]. Ionic liquid supports in gas phase hydrogenation, catalyzed by Pd, Ru, Ir, and Rh NPs, resulted in formation of 1,3-butadiene, C6-olefins, and benzene [256,257,258]. In the case of acetylene hydrogenation, ionic liquids with [BF_4_] anion promote higher conversion and lower selectivity, whereas the effects of ionic liquids with [PF_6_] anion are quite the opposite [259]. The Pd/BmimBF_4_/SiO_2_ stability is slightly decreasing after 6 h. The Ar plasma/H_2_-treated Pd/EmimBF_4_/SiO_2_ is more stable than the corresponding product of conventional reduction, due to minimized Pd/IL/SiO_2_ fluctuation phenomena. Noteworthy, the size of Pd NPs and ionic liquids are stable under plasma conditions [260]. The catalytic properties of Pd/TiO_2_ catalysts depend on the way of preparation (impregnation vs electroless deposition) and the type of atmosphere used for calcination-O_2_/N_2_ [261] or H_2_ [262]. Dispersion of Pd increased in the presence of Ti^3+^ significantly. TiO_2_ support prepared in N_2_ atmosphere under electroless conditions possessed similar average crystallite size, surface area and pore diameter resulted in improved catalytic performance. Full acetylene conversion with high ethylene selectivity (92%) is achievable with 1%Pd/TiO_2_-N_2_. At the same time, H_2_ treatment of sol-gel derived TiO_2_ increases the number of Ti^3+^ defective sites on its surface. 

**Figure 10:**
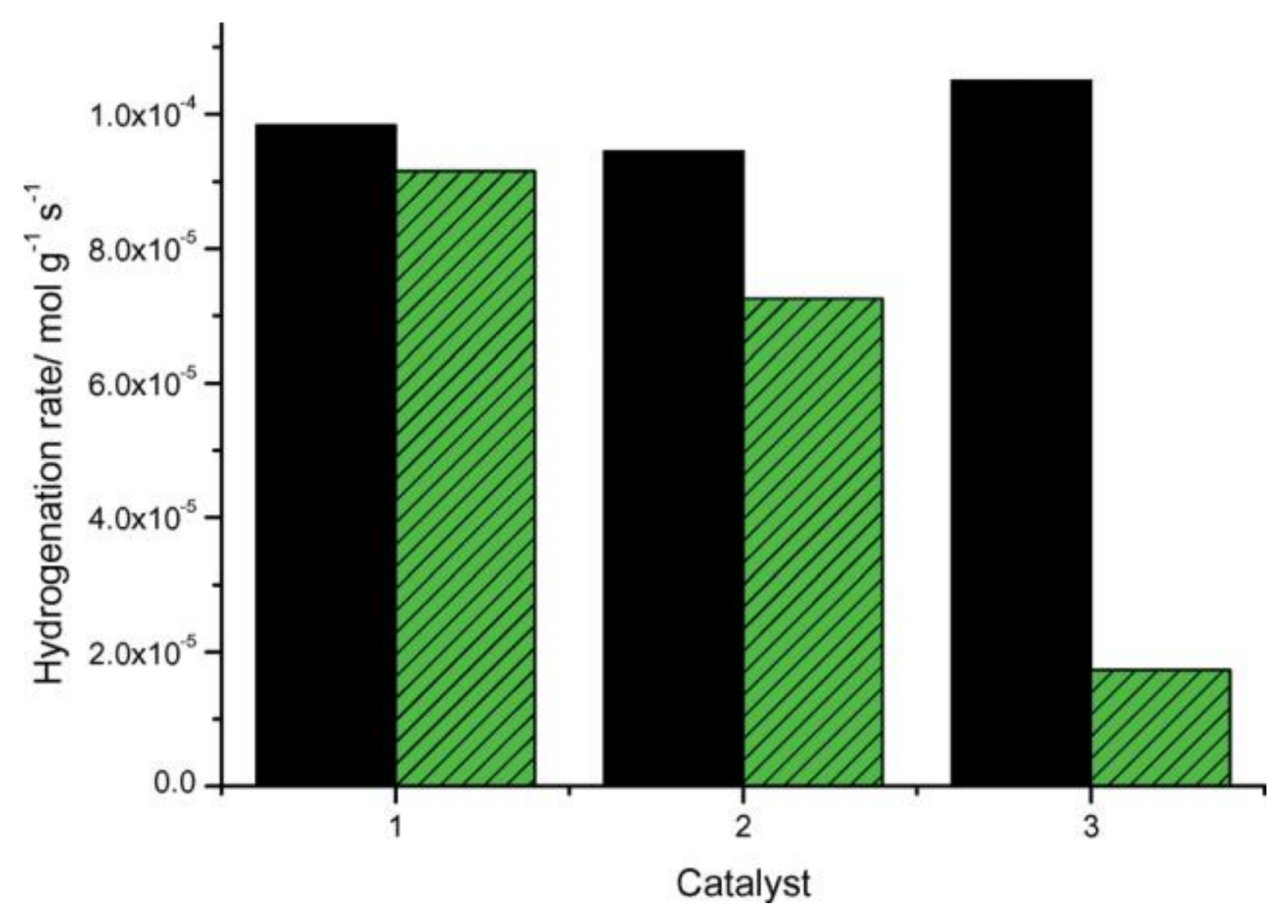
Hydrogenation rates of acetylene (black) and ethylene (shaded green): 1—Pd/TiO_2_, 2—Pd/TiO_2_ with 3‰ CO in feed, 3—PPh_3_ 2.5 Pd/TiO_2_ (reproduced with permission from [263]. Copyright 2015, the Royal Society of Chemistry).

Treatment of a Pd/TiO_2_ catalyst with triphenylphosphine (TPP) improves the selectivity and limits over-hydrogenation under low pressures [263]. Acetylene and ethylene have similar rates of hydrogenation over the unmodified Pd catalyst (Figure 6, label 1). Adding CO (3 ppm) has little impact on the results; although it diminishes the ethylene conversion rate (Figure 6, label 2), continuous addition of CO is required to maintain the gain in selectivity. Adding TPP suppresses ethylene hydrogenation (Figure 10, label 3) and promotes a 10-fold decrease in formation of the gas phase-specific byproducts (Table 1). Probably, acetylene more readily adsorbs to the modified catalyst, whereas ethylene adsorption is hindered. Stability of TPP under both low and high pressures makes its superiority especially relevant. 

**Figure 11:**
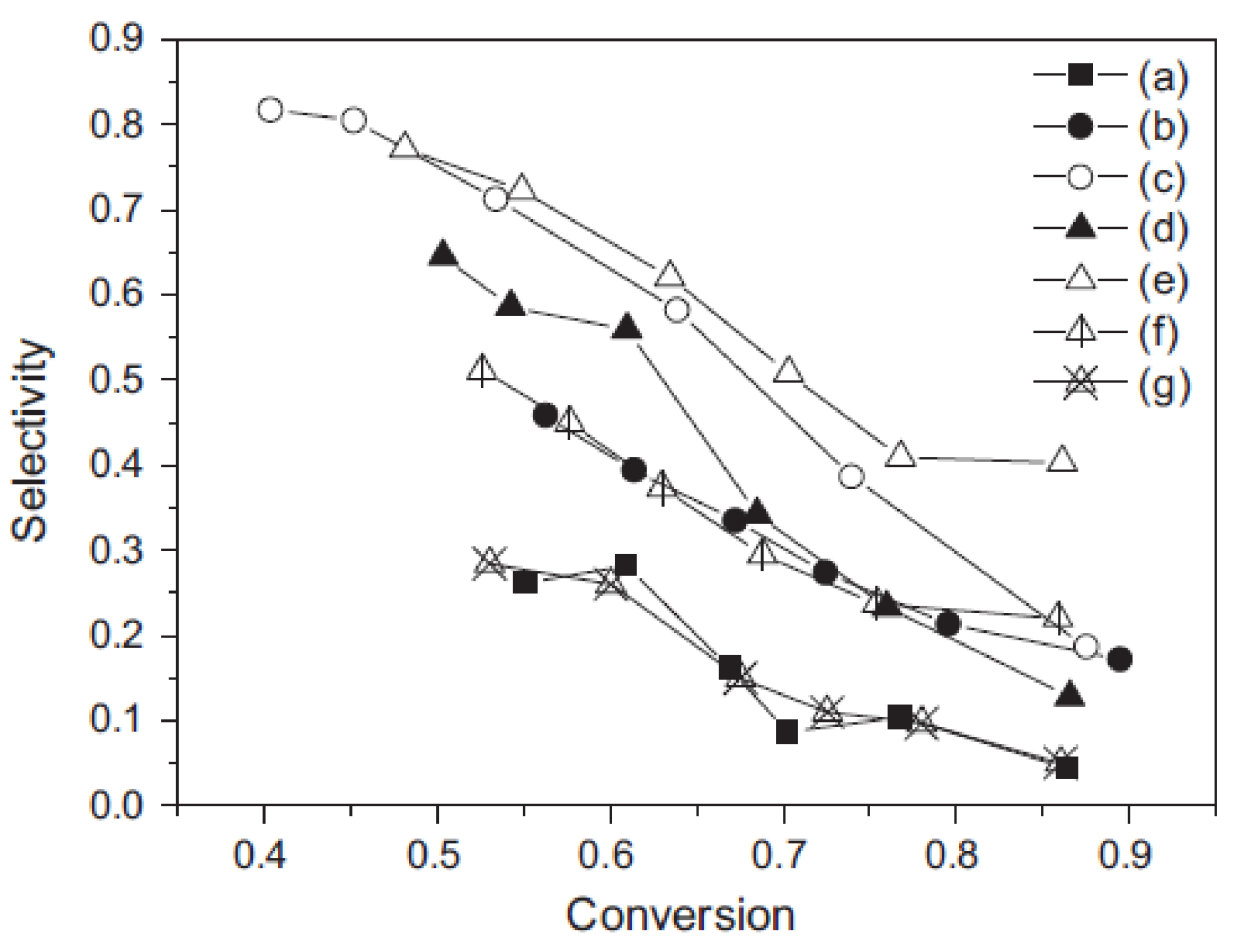
Performance of various Ti-modified Pd–La catalysts in acetylene hydrogenation. (H_2_/Acetylene = 2, reaction temperature = 60 °C). (a) Pd/300, (b) Pd–1La/300, (c) Pd–1La/500, (d) Pd–1La–0.1Ti/300, (e) Pd–1La–0.2Ti/300, (f) Pd–1La–0.4Ti/300, (g) Pd–0.2Ti/300 (reproduced with permission from [264]. Copyright 2014, Elsevier Inc.).

Double additives (Ti and La oxides) are moderate improvers of Pd catalysis [264]. La oxide is beneficial to the performance, but its reduction requires high temperatures. Ti oxide partially covered by La oxide maintained the modified geometric and electronic structures of the catalyst even after low-temperature reduction. It also confers higher ethylene selectivity than the single La oxide. Probably, Ti oxide prevents the La species from re-migrating during high-temperature reduction to their initial positions after air contact. In total, the Pd catalyst modified with Ti and La simultaneously exhibited the best results compared with pure Pd and monomodified catalysts (Figure 11).

#### 2.8.1. Bimetallic Pd-based Catalysts: Pd and Ga/In

**Figure 12:**
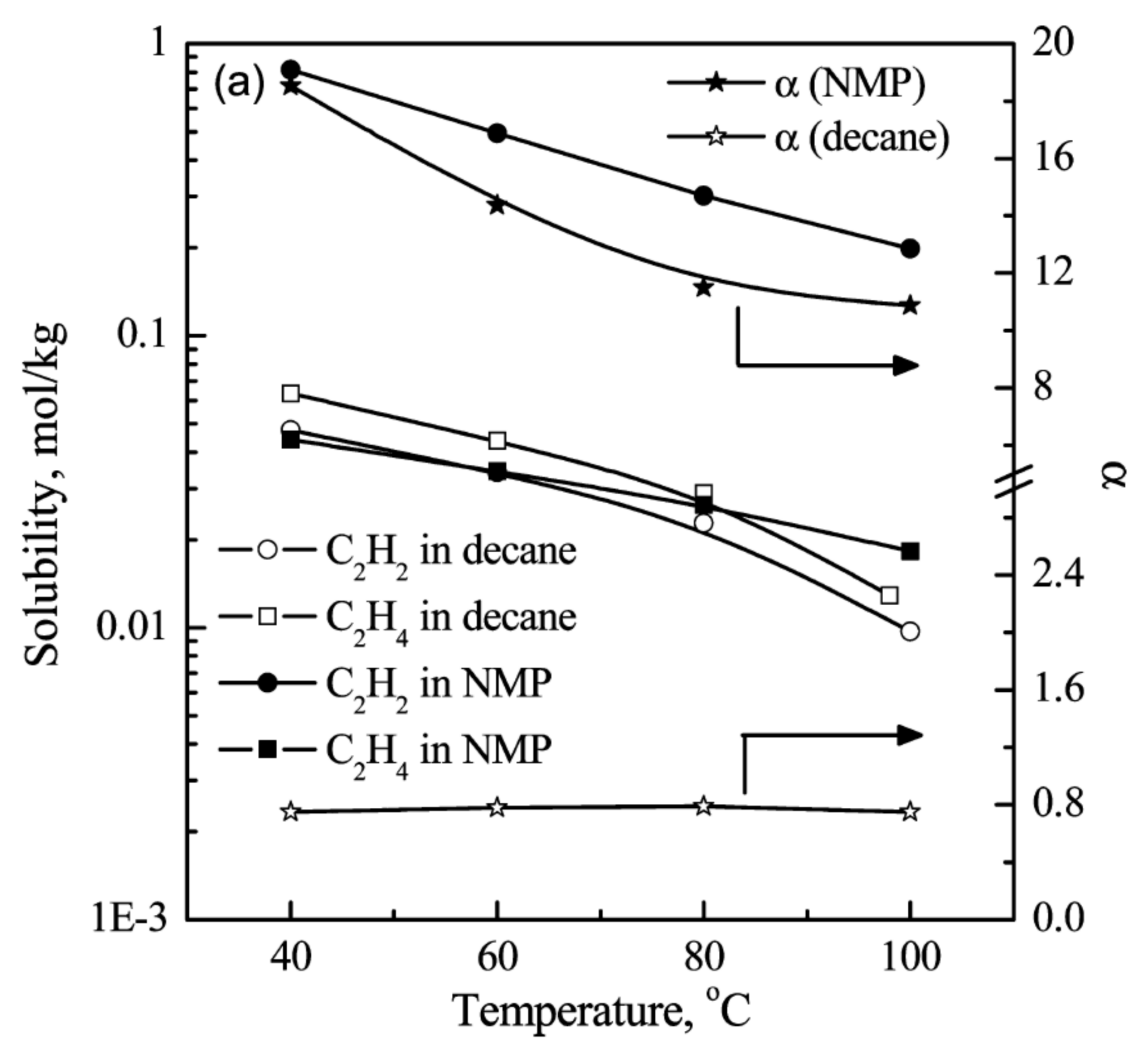
Solubility of acetylene and ethylene in NMP and decane. The catalyst is Pd/low surface area silica gel (reproduced with permission from [265]. Copyright 2013, American Chemical Society.).

As compared with ethylene, acetylene is much more soluble in acetone, dimethylformamide, N-methylpyrrolidone (NMP) and some ionic liquids (Figure 12) [265]. At conversions near 100%, ethylene selectivity becomes unevenly distributed between the liquid and gas phases. For instance, it may constitute 80–96% in the NMP liquid phase and 70% in gas phase. For this reason, NMP augments the hydrogenation, which a nonpolar solvent (e.g., decane) would be incapable of. NMP was also used for acetylene hydrogenation in the presence of 1% Pd/Ga_2_O_3_ catalyst [266]. Another study with a similar catalyst was less successful, providing only 53% acetylene conversion and 36% ethylene selectivity in liquid phase, possibly due to the low solubility of hydrogen in NMP in the presence of alloys with 0.5%Pd–0.08%Ga [267]. Low selectivity at full conversion was also reported for Pd/Ga_2_O_3_-Al_2_O_3_ catalysts in NMP as a liquid phase [268]. 

**Figure 13:**
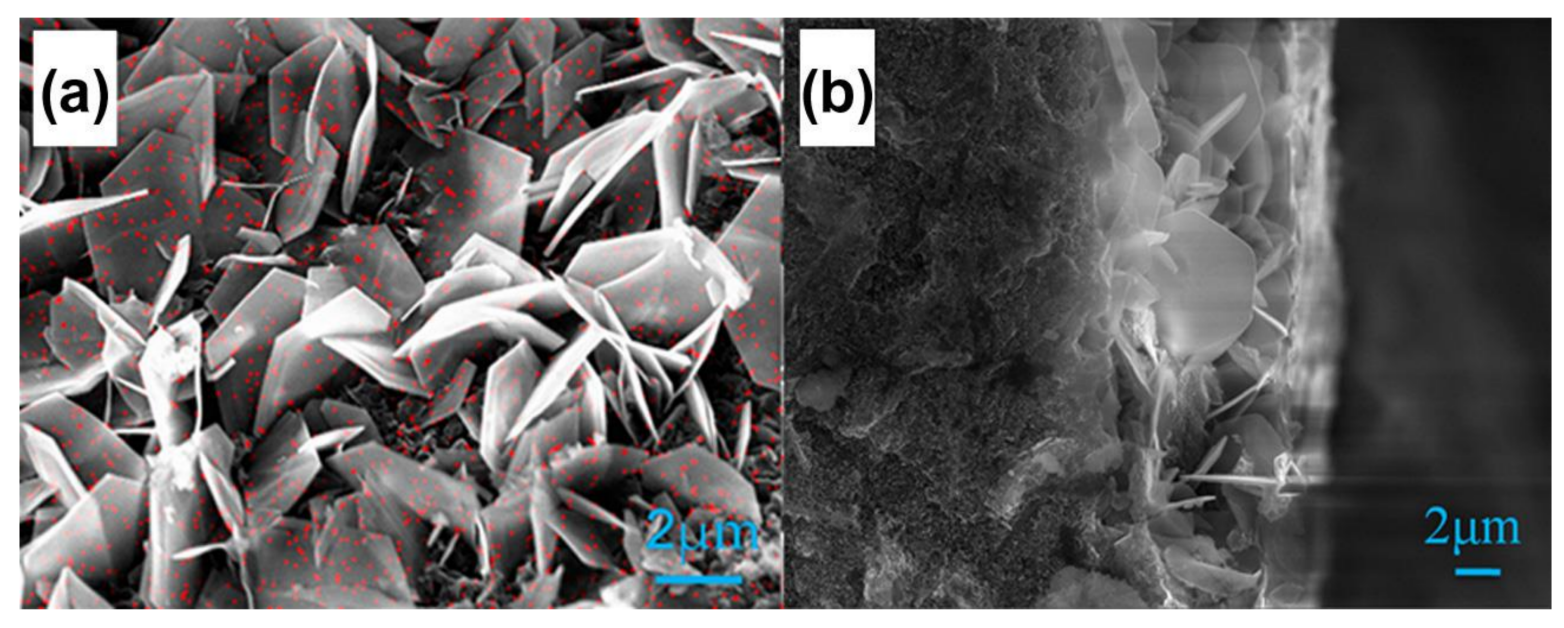
SEM images of the of the bimetallic Pd/Ga catalyst precursor from the surface (**a**) and in the cross section (**b**) (reproduced with permission from [269]. Copyright 2014, Elsevier Inc.).

A bimetallic Mg-Ga-Al-layered double hydroxide (LDH) Pd catalyst is more effective than a monometallic Pd/MgO-Al_2_O_3_ system [269]. Although bimetallic Pd/Ga catalysts are just behind the pure Pd catalyst in activity, the bimetallic system shows much better selectivity in comparison with the monometallic Pd/MgO-Al_2_O_3_ catalyst without Ga. High dispersion of bimetallic Pd/Ga nanoalloy, with the average particle size of 2.6 nm, is due to the uniform distribution of Ga and the electrostatic interaction between Pd and support layer (Figure 13). Strong adhesion of MgGaAl-LDH to Al_2_O_3_ is a consequence of direct growth of the catalyst on the spherical Al_2_O_3_. Pure Pd/Ga alloys (GaPd_2_ and GaPd) are also moderately active, but there was no rate limitation of acetylene concentration and occupation on the catalyst surface [270]. Inkjet printing allows to obtain Pd/Ga catalysts with thin, uniform and homogeneous wall coatings over the entire channel surface [271]. But even in this case the best ethylene selectivity is only 76%. Using Ni instead of Ga in the NiMgAl-LDH catalysts is not particularly beneficial [272], although the strong interaction between Pd and Ni modulates the affinity of hydrogen to the catalyst surface in comparison with similar monometallic systems.

**Figure 14:**
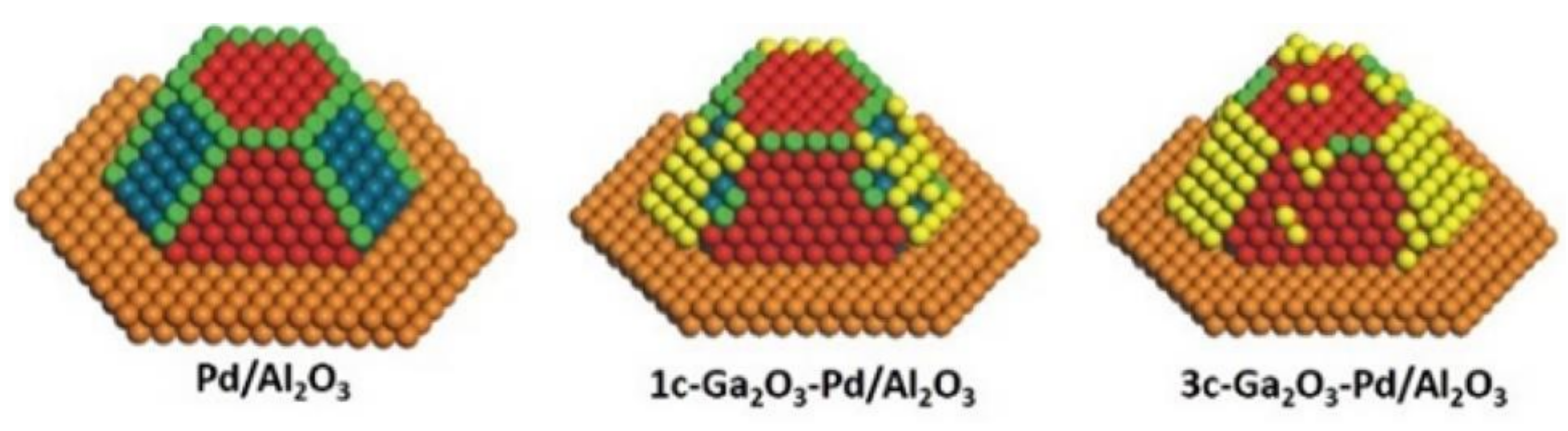
Schematic drawing of supported Pd particles in Pd/Al_2_O_3_- and Ga_2_O_3_-coated Pd particles in 1c-Ga_2_O_3_−Pd/Al_2_O_3_ and 3c-Ga_2_O_3_−Pd/Al_2_O_3_. The red, blue, green, orange, and yellow spheres represent terrace atoms of Pd (111) facets, facets other than Pd(111), corner and edge atoms of Pd(111) facets, Al_2_O_3_, and Ga_2_O_3_, respectively (reproduced with permission from [273]. Copyright 2016, American Chemical Society).

Deposition of Ga_2_O_3_ adlayers on Pd particles supported on alumina enhance the catalyst performance by activating edge sites [273]. The atomic layer deposition results in Ga_2_O_3_ accumulation at the edges and open facets of Pd. These edges are transformed into catalytically active terrace-like sites. The treatment promotes an increase in the number of active sites and suppresses the formation of poisoning carbonaceous deposits on open facets. The catalytically active particles acquire polyhedron shape with (111) and (100) facets (Figure 14). They show 95% ethylene selectivity at 75% acetylene conversion. 

#### 2.8.2. Bimetallic Pd-based Catalysts: Pd and Zn/Co

**Figure 15:**
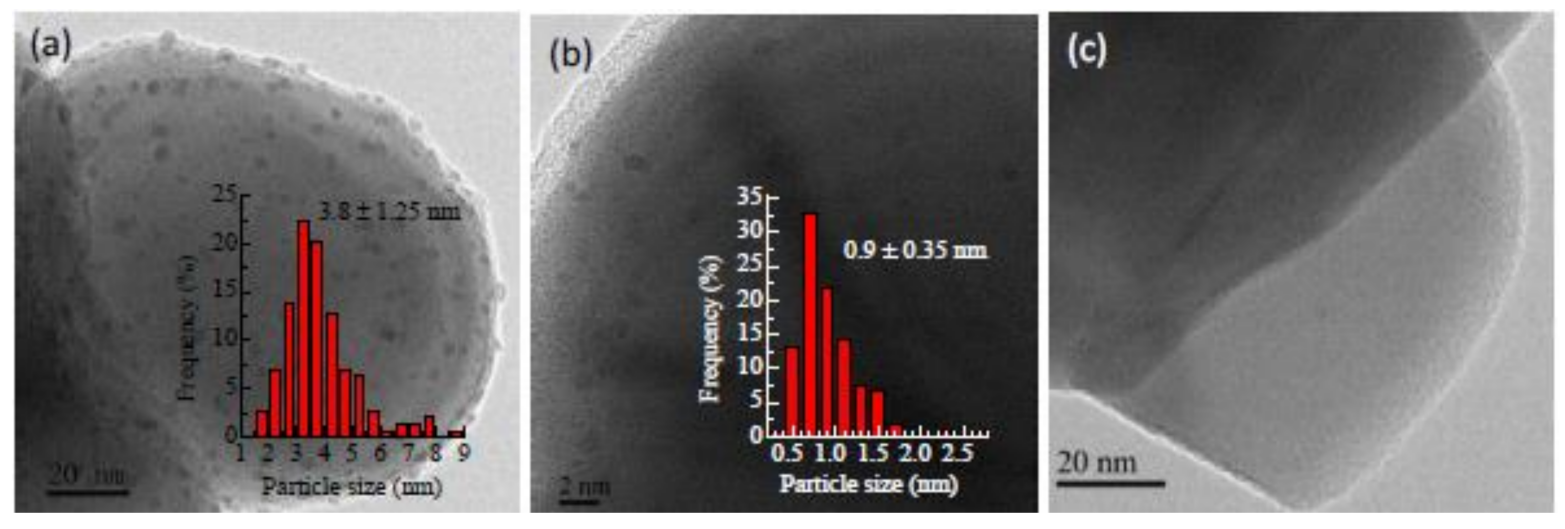
TEM images and particle size distributions of the catalysts: **a**—1%-Pd/ZnO; **b**—0.1%-Pd/ZnO; **c**—0.01%-Pd/ZnO reduced at 100 °C (reproduced with permission from [274]. Copyright 2016, Elsevier Inc.).

A series of Pd/ZnO single-atom catalysts shows 80% ethylene selectivity at nearly full acetylene conversion [274]. Decreasing the Pd loading from 1% to 0.1% and reduction of the samples at relatively low temperatures of ~100 °C leads to smaller Pd NPs with distributed single-atom Pd sites for the 0.01-Pd/ZnO catalyst (Figure 15). These high-valent Pd active sites promote electrostatic interactions with acetylene, but restrain ethylene hydrogenation due to the inability of *σ*-bond formation. Using Zn as an intermetallic catalyst instead of ZnO improves the performance to 90% selectivity at full conversion [275]. Nanostructured Pd-Zn-Pd ensembles have moderate *σ*-bonding mode for acetylene with two neighboring Pd sites and weak *π*-bonding of ethylene adsorption on the single Pd site.

**Figure 16:**
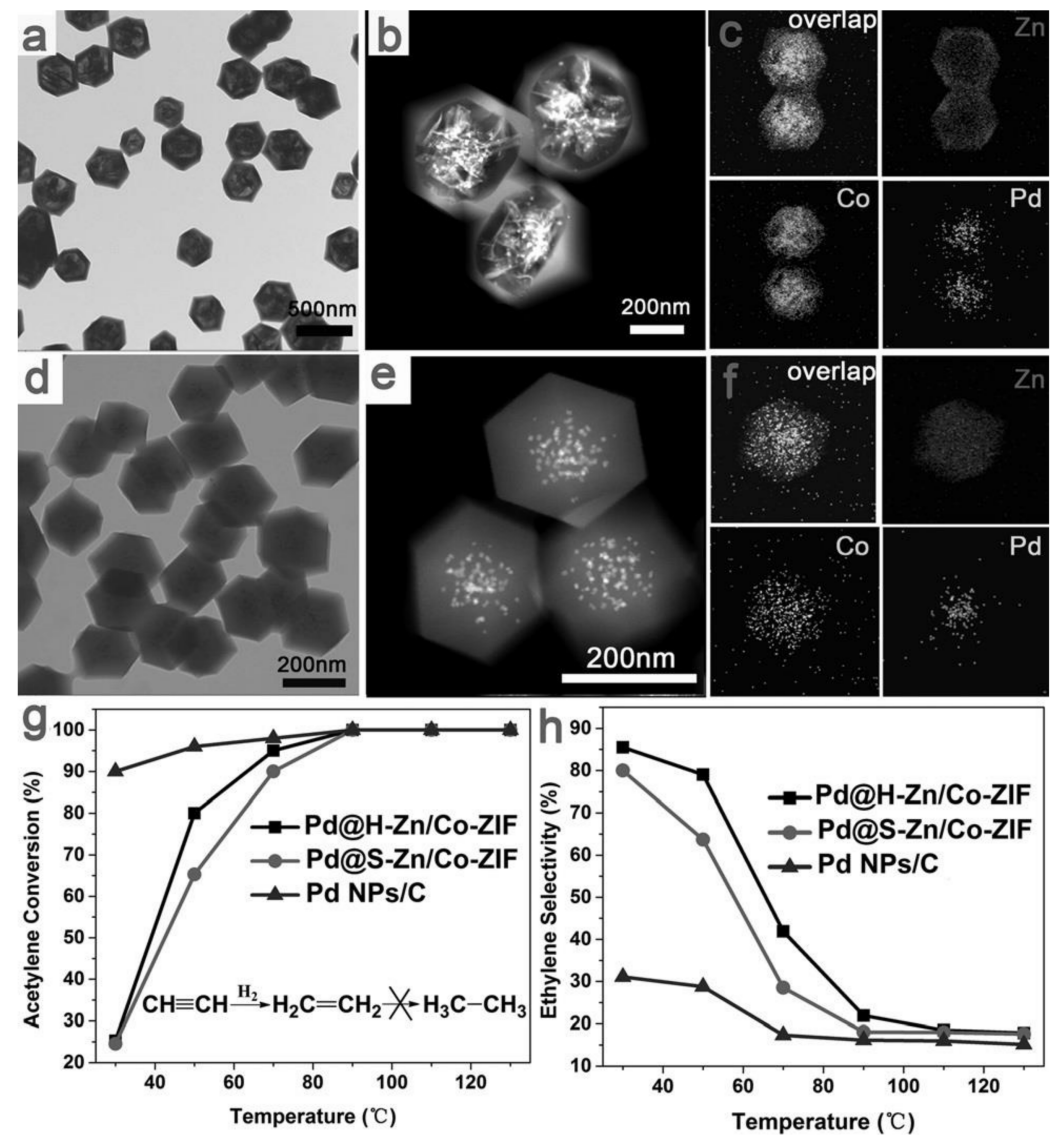
TEM pattern (**a**), HAADF-STEM image (**b**), and EDX mapping (**c**) of Pd@H-Zn/Co-ZIF. TEM pattern (**d**), HAADF-STEM image (**e**), and EDX mapping (**f**) of Pd@S-Zn/Co-ZIF. Acetylene conversion (**g**) and ethylene selectivity (**h**) as functions of temperature (reproduced with permission from [276]. Copyright 2015 WILEY-VCH Verlag GmbH & Co. KGaA).

Well-defined hollow Zn/Co ZIF rhombic dodecahedron was obtained using special modification of ZIF-67@ZIF-8 structures in the presence of Co^2+^ [276]. A noble metal@MOF yolk-shell composite was prepared and tested as a catalyst for acetylene hydrogenation. The high-angle annular dark-field scanning TEM (HAADF-STEM) analysis showed that Pd@H-Zn/Co-ZIF was composed of hollow coordination polymer with embedded Pd NPs (Figure 16). The obtained catalysts were compared with Pd NPs in selective acetylene hydrogenation and demonstrated high activity and ethylene selectivity at mild temperatures.

**Figure 17:**
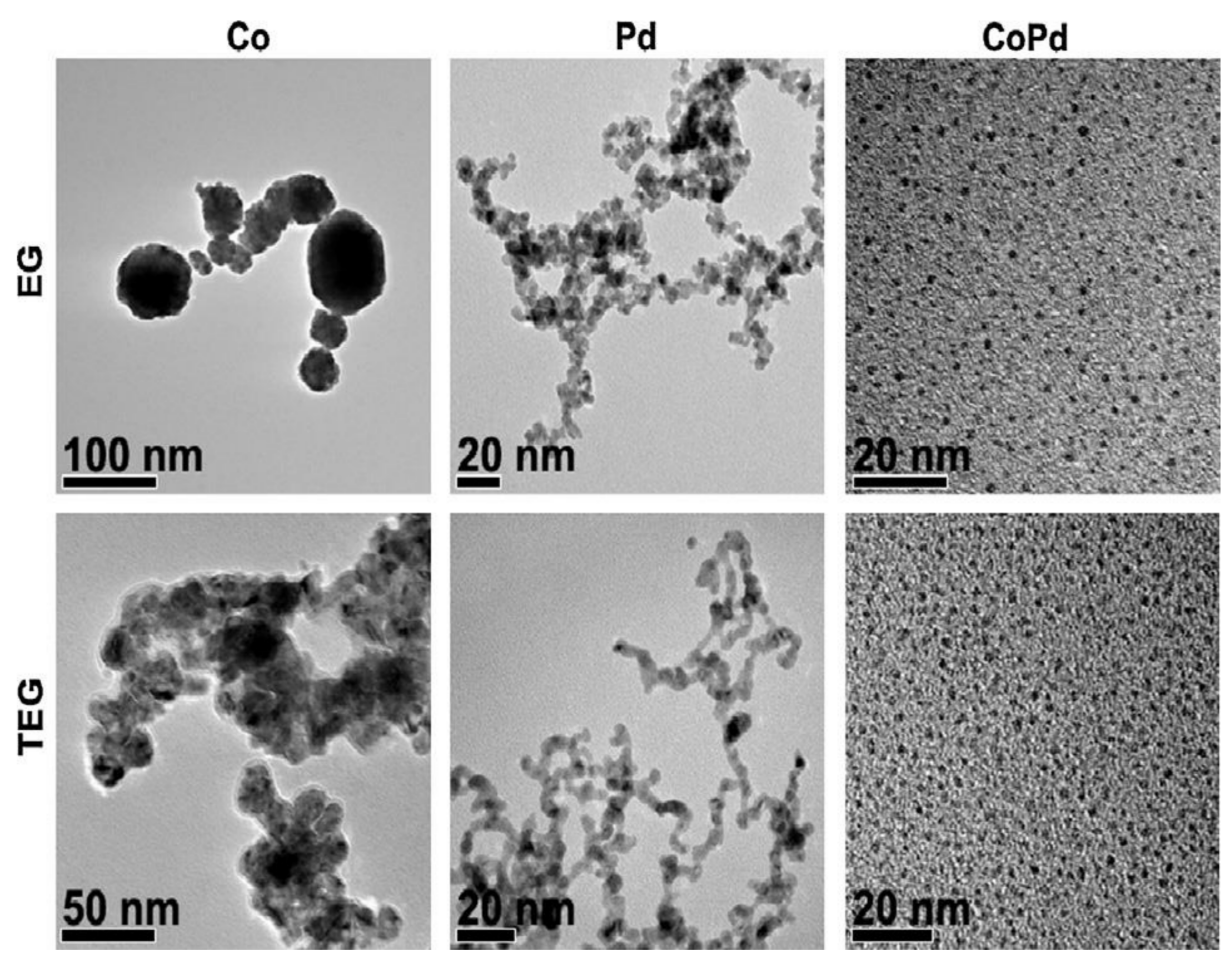
Representative TEM images of the Co, Pd and CoPd nanoparticles and structures formed after refluxing for 1 h at 195 °C in EG or at 280 °C in TEG. EG—ethylene glycol, TEG —tri-ethylene glycol (reproduced with permission from [277]. Copyright 2013, Elsevier Inc.).

**Figure 18:**
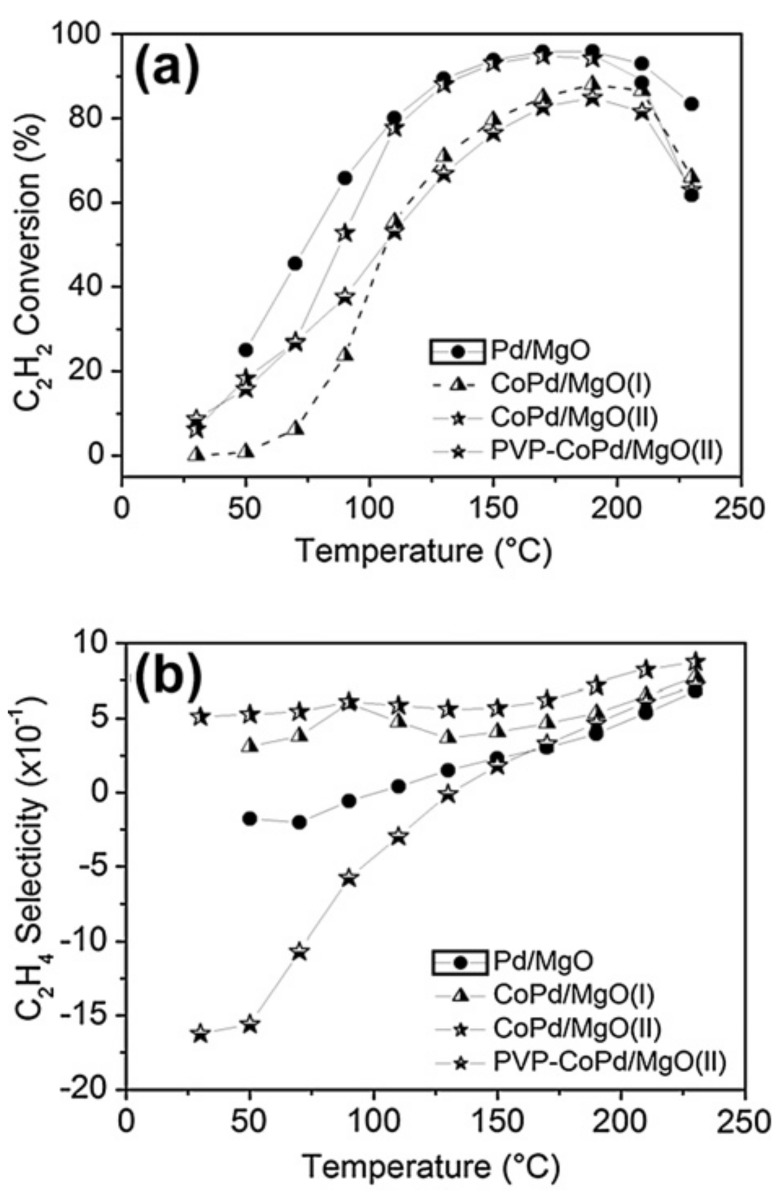

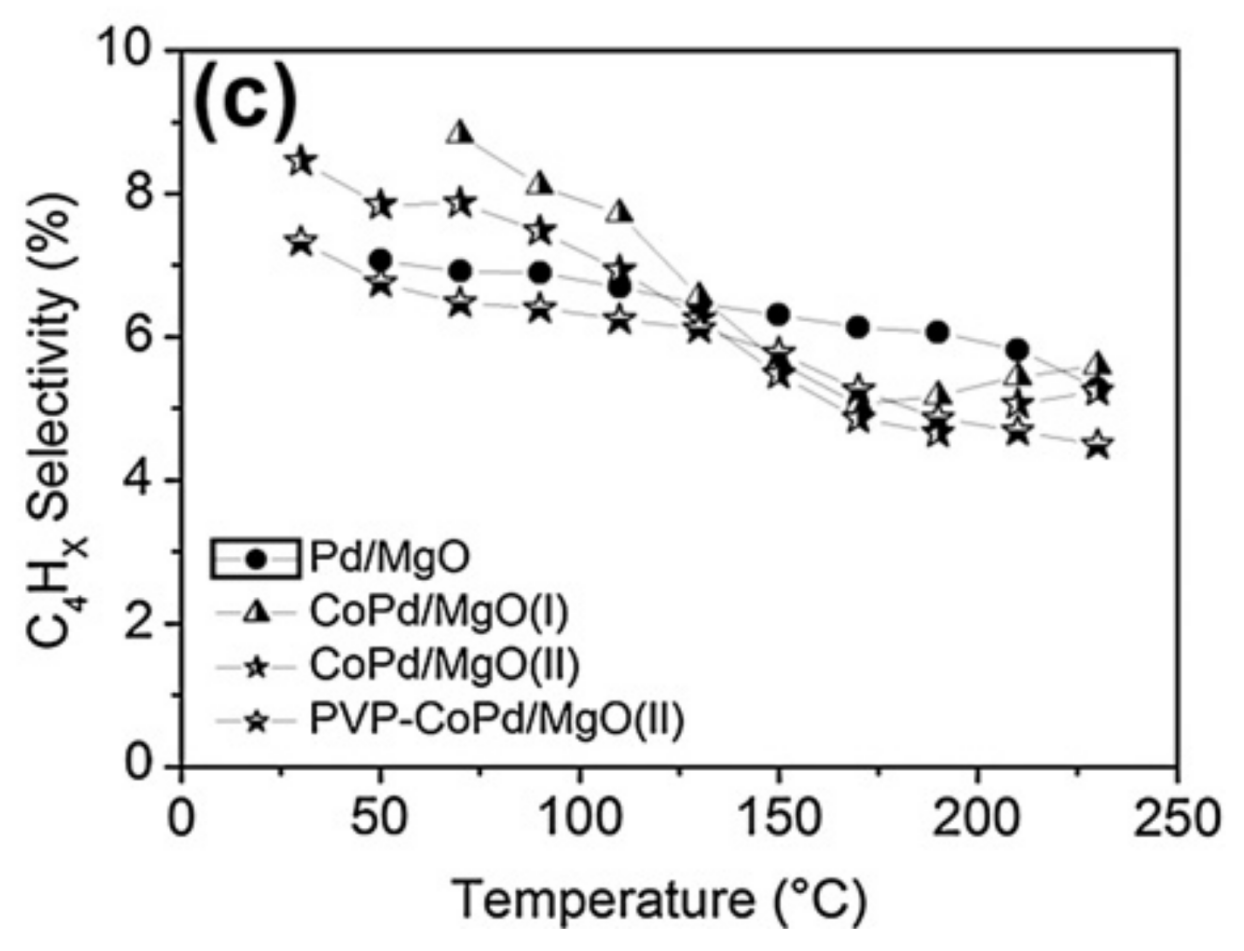
Acetylene conversion (**a**), ethylene selectivity (**b**), and C4 selectivity (**c**) as functions of temperature. Acetylene conversion is given by the difference of the inlet and outlet concentrations in vol %. Ethane formation is given by vol% of ethane in the reactor off-gas. (Conditions: 0.5 vol % C_2_H_2_, 1 vol % H_2_, and 70 vol % C_2_H_4_ in N_2_, flow velocity 50 mL/min.) (reproduced with permission from [277]. Copyright 2013, Elsevier Inc.).

Polyols (tri-ethylene glycol and ethylene glycol) were used as a medium for Pd/Co bimetallic system preparation [277]. CoPd NPs were deposited on active carbon, MgO, and Al_2_O_3_ using poly(N-vinylpyrrolidone) as a stabilizing agent. Addition of Co led to formation of bimetallic NPs with better dispersion, as compared with monometallic Co or Pd NPs (Figure 17). Spherically shaped Co/Pd NPs of 3 nm diameter were catalytically active in hydrogenation and showed significantly higher ethylene selectivity, as compared with Pd NPs (Figure 18). The decreases in acetylene and ethylene adsorption heat were due to effects of Co on the electronic structure of Pd, whereas the increase in ethylene selectivity was caused by separation of individual Pd sites by Co incorporation. 

#### 2.8.3. Pd-Based Bimetallic Catalysts: Pd-Cu/Ag/Au

**Figure 19:**
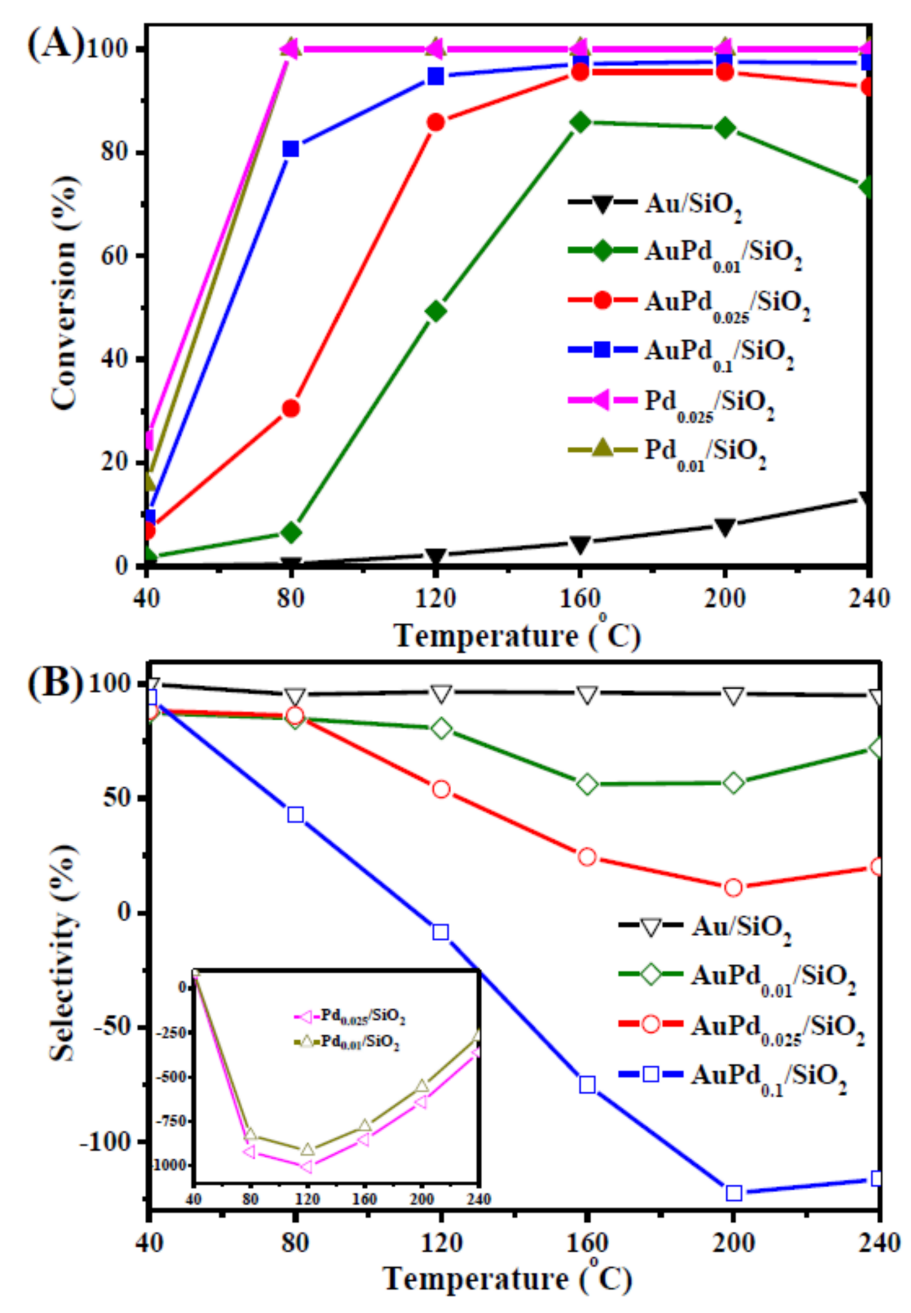
Conversion (A) and (B) selectivity as functions of temperature for acetylene semi-hydrogenation over Au/SiO_2_, AuPd_0.01_/SiO_2_, AuPd_0.025_/SiO_2_ and AuPd_0.1_/SiO_2_ catalysts (reproduced with permission from [278]. Copyright 2014, the Royal Society of Chemistry.).

Pd single-atom alloyed structures supported on silica, with ppm content of Pd, were alloyed with gold NPs and tested in hydrogenation [278]. Interestingly, the bimetallic systems showed a 1000% better ethylene selectivity at 160 °C as compared to monometallic Pd/SiO_2_ with similar Pd loadings (Figure 19). 

The role of Pd in this case is to increase the conversion and to decrease the reaction temperatures, whereas Au provides isolation between Pd atoms and prevents acetylene over-hydrogenation. An Au/Pd alloy with TiO_2_ support, prepared with the 2-nozzle system, exhibited the highest conversion (50%) with high ethylene selectivity (95%) at only 40 °C [279]. Partial coating of the most active bimetallic Au/Pd particles with the Ti precursor afforded less active catalysts due to the formation of Ti-O species. Various configurations of the particles were achieved by using different types of feeding system (single- vs. double-nozzle). Formation of bimetallic NPs was minimized by separated feeding and enhanced by co-feeding. 

The results of acetylene hydrogenation over Ag- and Au-Pd/SiO_2_ bimetallic catalysts suggest the possibility of two alternative pathways [280]. High coverage of Pd with Ag or Au leaves small ensembles of Pd sites, which adsorb acetylene as a π-bonded complex thus promoting its semi-hydrogenation to ethylene. The lower coverage leaves much larger, contiguous and merging Pd surface sites, where acetylene is adsorbed as a multi-σ-bonded complex preferably resolved by ethane formation. 

The alumina phases of Al_2_O_3_ support and the mode of metal deposition on TiO_2_ support dramatically affect the Pd/Ag catalyst performance. Specific combinations of electrostatic properties with acidity determine differential strength of active sites: decreasing acidity of support is caused by a decrease in site strength [281]. A core-shell Pd@Ag bimetallic catalyst of this type, showing 73.5% selectivity at 98.5% conversion, is prepared by photodeposition of Pd and Ag, with the support acting as a photocatalyst during the preparation procedure [33]. The formed species have superficial Pd core and support specific growth of Ag on the Pd particle surface during the photocatalytic deposition process. Blockage of the high coordination Pd sites by the Ag shell, as well as the increased number of isolated Pd sites, results in better performance. 

**Figure 20:**
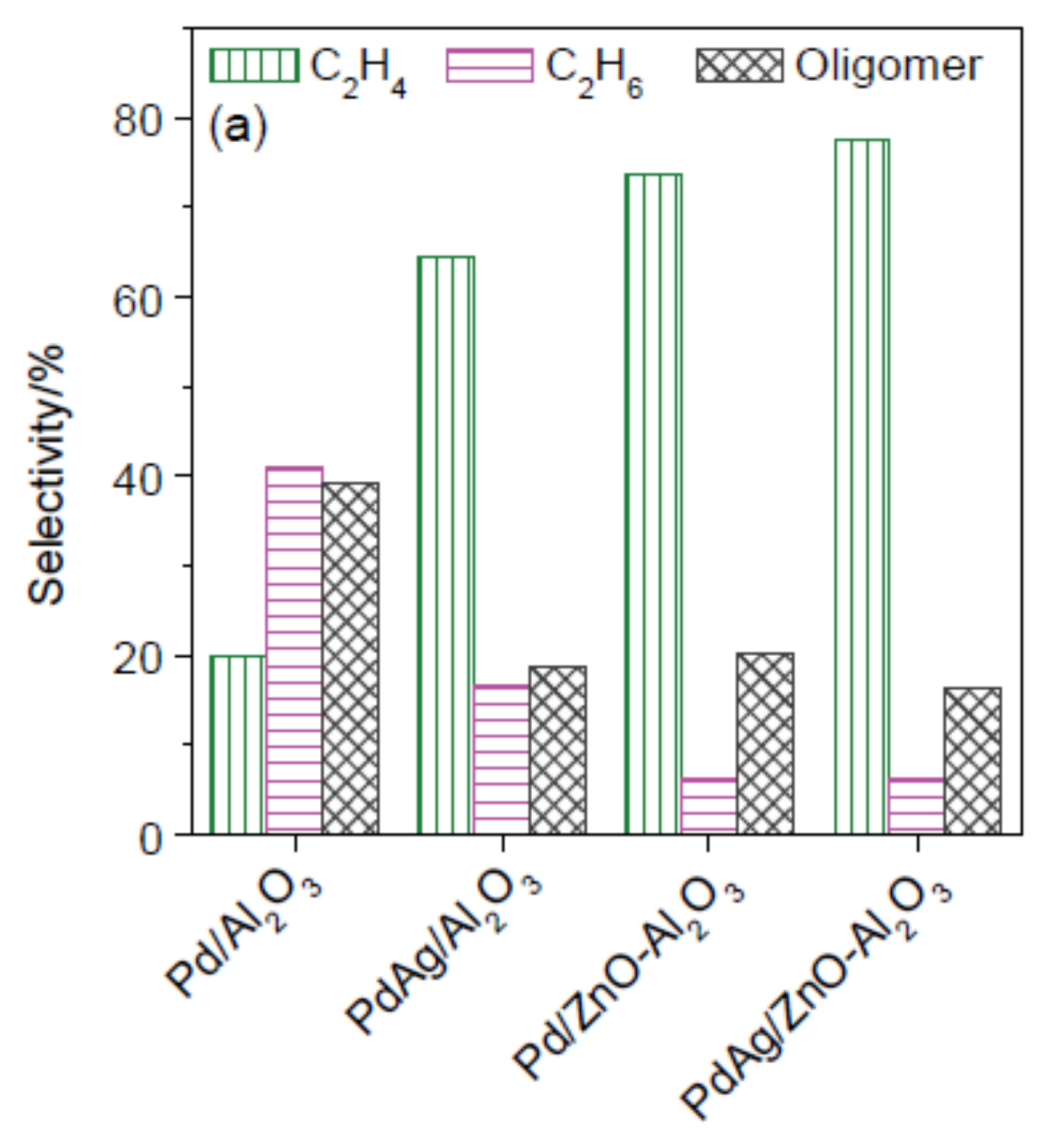

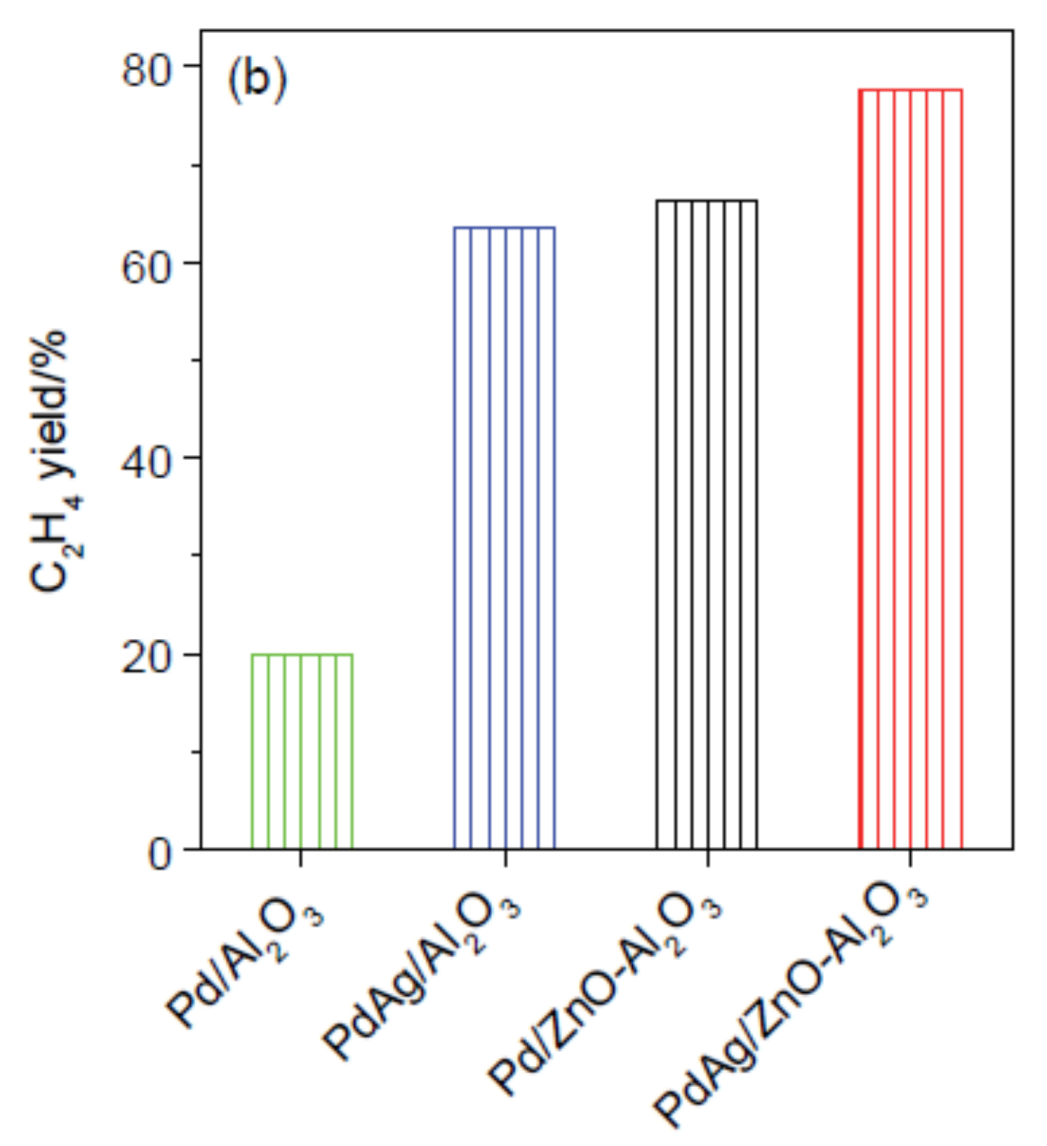
Product selectivities (**a**) and C_2_H_4_ yields (**b**) for PdAg/ZnAl catalysts, 180 min at 90 °C (reproduced with permission from [283]. Copyright 2017 WILEY-VCH Verlag GmbH & Co. KGaA).

A defect-rich Pd/Ag catalyst on NiTi-layered double hydroxide support was tested in acetylene hydrogenation [282]. The compromise values of selectivity (82%) and conversion (90%) were achieved by virtue of the small size and high dispersion of Pd/Ag NPs and the abundance of Ti^3+^ defective sites. Withdrawing of electron density from Ti^3+^ by Pd enhanced the Pd-support interaction. Using ZnAl instead of TiO_2_ significantly improved the selectivity/conversion and also the oligomer selectivity [283]. Decreased amounts of carbonaceous deposits were observed for PdAg/ZnO-Al_2_O_3_ in comparison with Pd/Al_2_O_3_, PdAg/Al_2_O_3_, and Pd/ZnO-Al_2_O_3_ (Figure 20). The results were further improved to full acetylene conversion with 83.8% ethylene selectivity at 70 °C by using mixed Mg-Ti supports [284]. Presumably, the moderate acidic sites of Mg_0.5_Ti_0.5_O_y_ support accelerate hydrogen-spillover effect followed by facilitated hydrogen activation and dissociation. The selectivity is additionally improved by electron transfer from basic sites and Ti^3+^ species of Mg_0.5_Ti_0.5_O_y_ support. A silica gel-supported single-atom Pd/Ag catalyst demonstrated 92.3%/92.6% selectivity/conversion due to extremely low content of Pd (ppm) [285]. The isolated electron-rich Pd atoms are capable of achieving the best results even in an ethylene-rich stream. 

**Figure 21:**
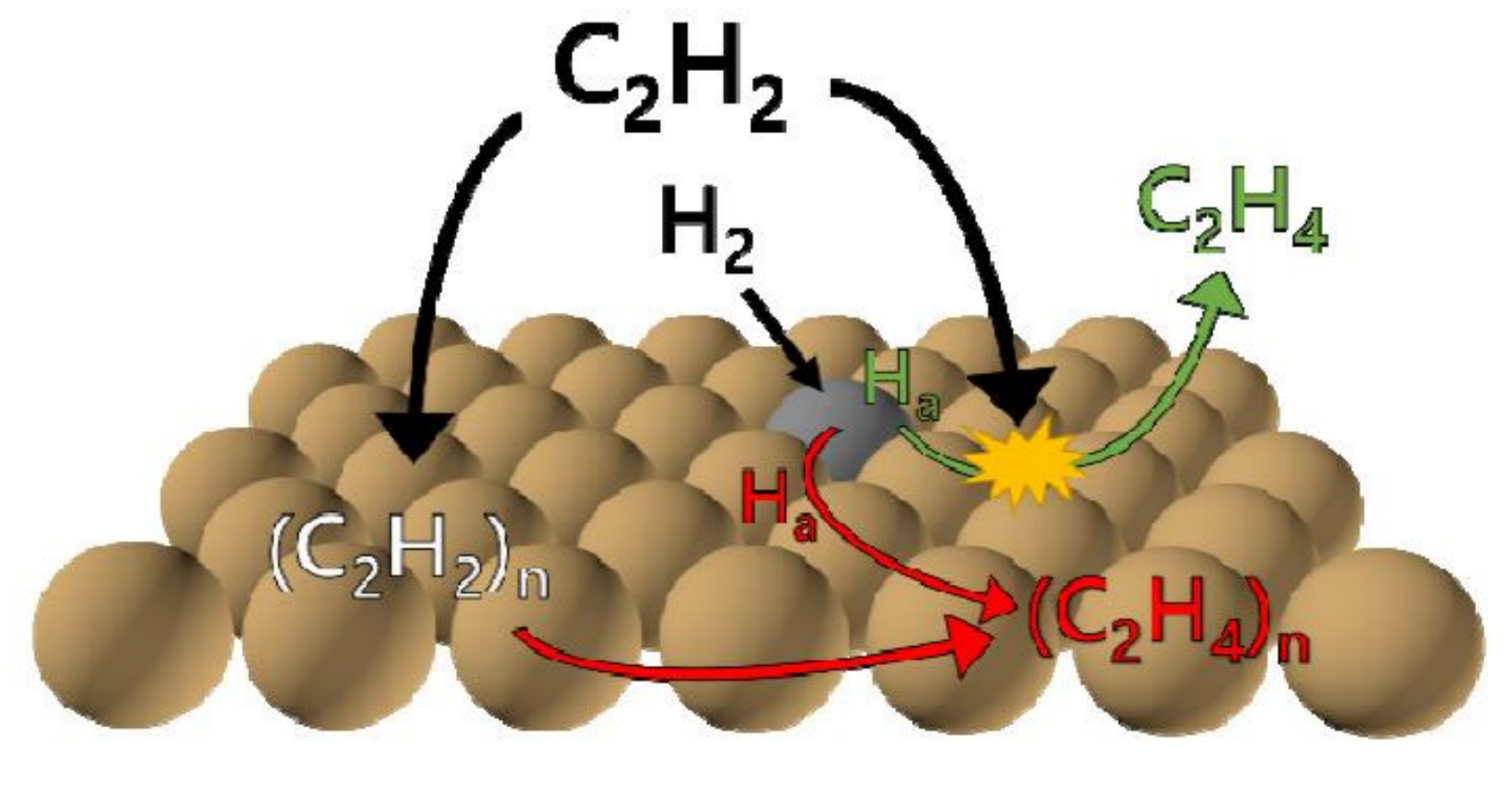
Proposed mechanism of acetylene hydrogenation over a Pd/Cu catalyst with deposited carbonaceous layer (reproduced with permission from r [287]. Copyright 2017, American Chemical Society.).

A bimetallic Pd/Cu (1:50) catalyst on alumina showed a good 70%/99% selectivity/conversion at 423 K [286]. The catalyst combines the properties of Cu and Pd; its high ethylene-selective activity at moderately low temperatures is attributed to facile dissociation of H_2_ on Pd with the immediate spillover onto Cu sites where the reaction proceeds. At lower temperatures, the ability of Cu to convert acetylene is poor (9.59%, Table 2). At higher temperatures, the acetylene conversion increases and the ethane selectivity is kept constant, but the ethylene selectivity is limited to 72% because of oligomerization. A modified Pd/Al_2_O_3_ catalyst shows better acetylene conversion at lower temperatures, but predominantly to ethane. Efficient and selective acetylene hydrogenation over Pd/Cu catalysts proceeds at higher temperatures. Copper generally acts as a surface diluent decreasing the size of the palladium ensembles. After the hydrogenation over Pd/Cu(111) catalyst, the products were simultaneously analyzed in gas phase [287], and reflection absorption infrared spectroscopy and Auger electron spectroscopy confirmed the presence of a carbonaceous layer on the surface (Figure 21). Palladium alters the nature of the carbonaceous species by promoting hydrogenation of polyacetylene, the product of copper-mediated acetylene coupling. 

**Figure 22:**
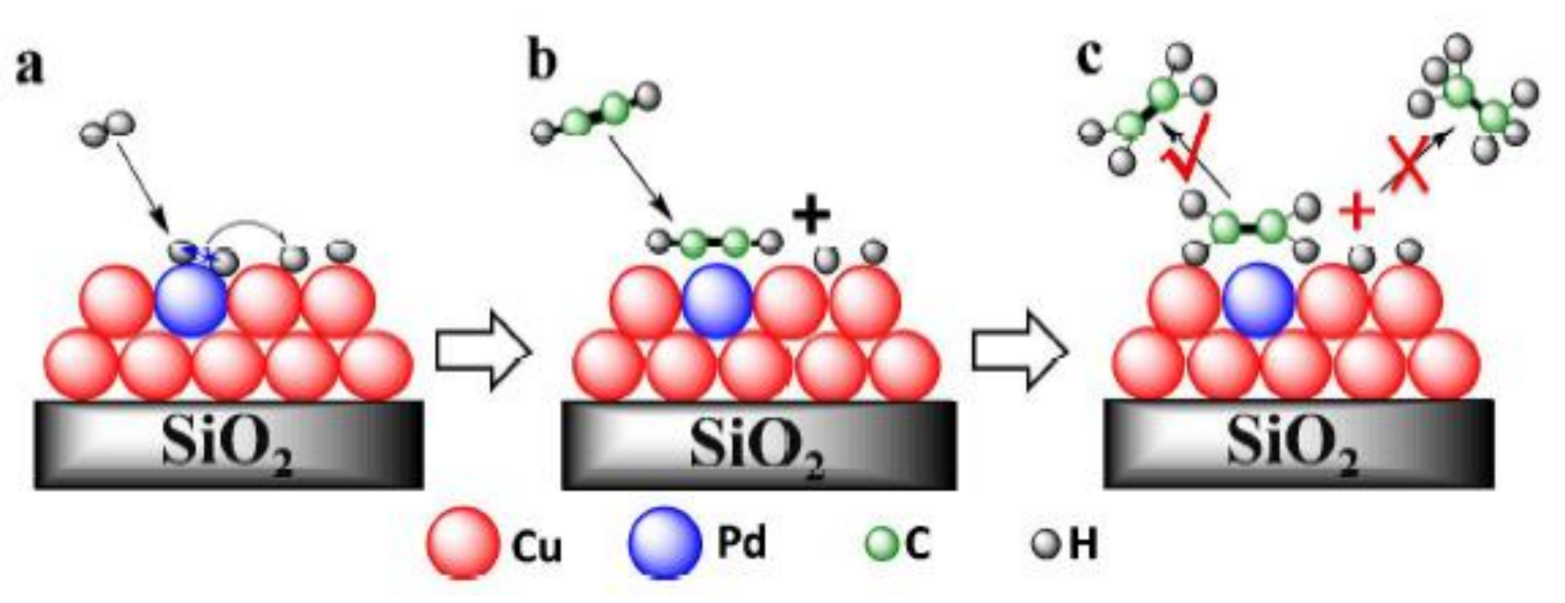
Proposed mechanism of acetylene hydrogenation over a silica-supported Pd/Cu single atom catalyst: **a**—dissociation of H_2_ and spillover of H atoms, **b**—adsorption and hydrogenation of acetylene, **c**—desorption of ethylene (reproduced with permission from [288]. Copyright 2017 American Chemical Society.).

A silica gel-supported alloyed Pd/Cu single atom catalyst showed 85% ethylene selectivity at full acetylene conversion [288]. The mechanistic investigations confirmed the role electron transfer from Cu to Pd in promoting hydrogen (Figure 22). Pd-doped Cu/Al_2_O_3_ catalysts with 50:1 Cu/Pd ratio, prepared by co-impregnation, sequential impregnation, or colloidal approach, show 80%/98% selectivity/conversion at only 353 K [289]. It is important to note that Cu/Pd bimetallic catalysts with fixed Pd content and varied Cu loadings are not as sensitive to the reductive pretreatment as the Pd/Ag catalysts [290]. However, being reduced at 400 °C and 250 °C, these catalysts show similar behaviors.

#### 2.8.4. Pd-Free Monometallic Catalysts: Mo

**Figure 23:**
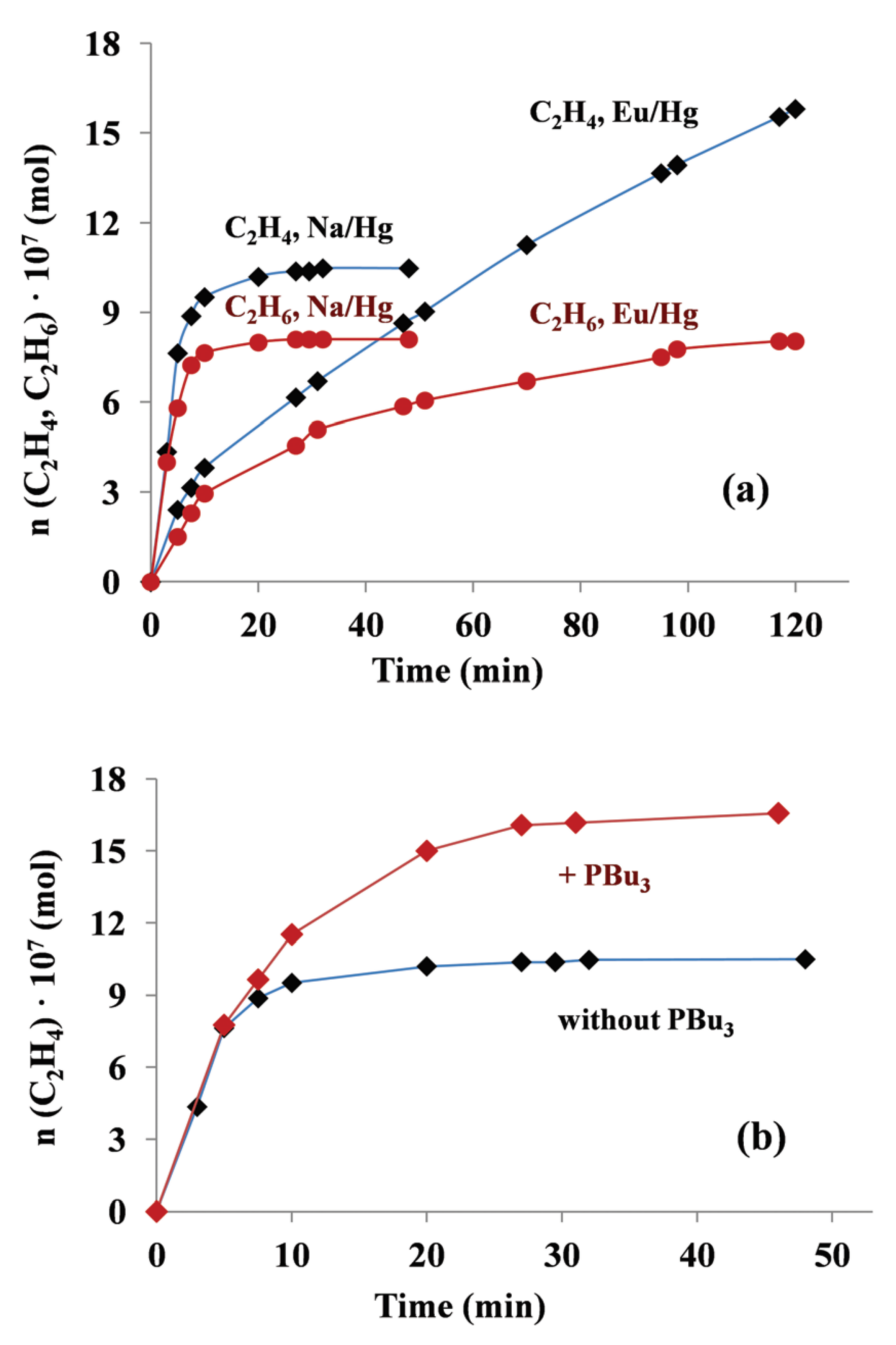
(**a**) Experimental kinetic curves of C_2_H_4_ and C_2_H_6_ accumulation in the reaction of C_2_H_2_ reduction by Na or Eu amalgam in the presence of Mo-complex. (**b**) The effect of PBu_3_ addition (C(PBu_3_) = 5 × 10^−3^ M) on the C_2_H_4_ accumulation rate in C_2_H_2_ reduction by Na amalgam in the presence of Mo-complex. (Conditions: C(Mo) = 2 × 10^−5^ M; 0.5 mL Eu/Hg (0.9 M) or 0.5 mL Na/Hg (3.5 M); 8 ml CH_3_OH; P(C_2_H_2_) = 0.13 atm, C(NaOCH_3_) = 0.09 M, C(H_2_O) = 0.02 M, C(PC) = 3 × 10^−4^ M, 21 °C) (reproduced with permission from [291]. Copyright 2016 the Royal Society of Chemistry.).

A rare reported example of a non-precious metal catalyst for acetylene hydrogenation is based on tri-, tetra-, and hexanuclear mixed-valence molybdenum clusters [291]. The liquid phase reaction was carried out in the presence of Na or Eu amalgams, the [Mo_4_Cl_4_O_2_(OCH_3_)_6_(CH_3_OH)_4_] complex, and methanol as a medium. After the catalyst reduction, ethylene and ethane were detected in the parallel reactions simultaneously. After addition of tributylphosphine to Mo-complex, both ethylene and ethane yields increased more than 50% (Figure 23).

#### 2.8.5. Pd-Free Monometallic Catalysts: Au

Gold catalysts, obtained by photo-dependent synthesis, showed 80% ethylene selectivity at full acetylene conversion after 12 h at 225 °C [292]. The best of them, 1% Au/C-TiO_2_ and 5% Au/C-TiO_2_, were tested for different H_2_/C_2_H_2_ ratios. At low H_2_/C_2_H_2_, both catalysts showed higher ethylene selectivities at limited acetylene conversion. Reciprocally, the excess of C_2_H_2_ favored its high conversion with low ethylene selectivity. After reduction at 250 °C, the 1% Au/C-TiO_2_ stably supported full acetylene conversion with 80% ethylene selectivity.

#### 2.8.6. Pd-Free Monometallic Catalysts: In

Indium oxide catalysts showed extraordinary good results at 550 K, with 85% ethylene selectivity at full conversion even for the ethylene excess conditions [293]. The most stable surface configuration, In_2_O_3-*y*_(111), was chosen to investigate the mechanisms of the main reaction and side reactions. Formation of a unique In_3_O_5_ site with O vacancy was predicted by theoretical calculations. Acetylene and H_2_ molecules co-adsorb to the catalytic site composed of In trimer and adjacent O atoms. This is an example of single-ensemble heterogeneous catalysis, which requires several particular atoms surrounding the empty site for the reaction to occur.

#### 2.8.7. Pd-Free Bimetallic Catalysts: Ni/Zn

**Figure 24:**
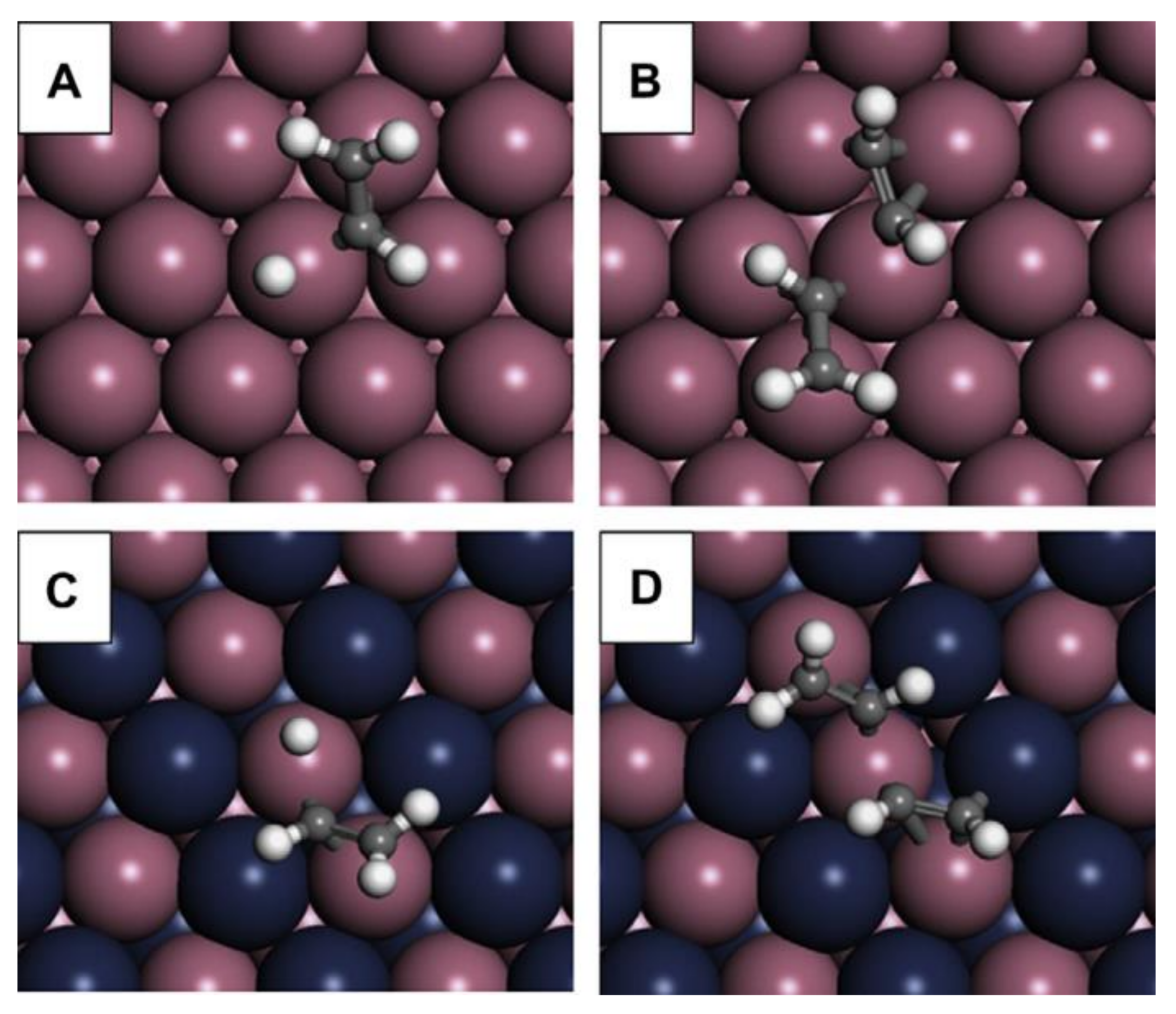
Transition states for vinyl hydrogenation and C–C bond formation on Ni(111) (**A** and **B**) and NiZn(101) (**C** and **D**), respectively. Ni atoms are light pink and Zn atoms are dark blue (reproduced with permission from [294]. Copyright 2014 Elsevier Inc.).

Nickel-zinc catalysts specifically suppress the oligomeric species formation by decreasing acetylene adsorption energy. This effect contributes to the higher ethylene selectivity [294]. Isotope labeling studies in a batch reactor confirmed that ethane was produced from both acetylene and ethylene, while oligomeric species were produced from acetylene only. Partial hydrogenation of acetylene led to adsorbed vinyl, which immediately reacted with adsorbed acetylene yielding C_4_H_5_, whose reaction with adsorbed hydrogen yielded butadiene as a final product (1-butene, *cis*-2-butene, and *trans*-2-butene can also be formed depending on the hydrogen availability). The carbohydrates can desorb from the catalyst surface and further react with acetylene molecules to form higher molecular weight oligomers. Transition states for vinyl hydrogenation and C-C bond formation on Ni and NiZn are shown in Figure 24. 

#### 2.8.8. Pd-Free Bimetallic Catalysts: Fe/Mo

**Figure 25:**
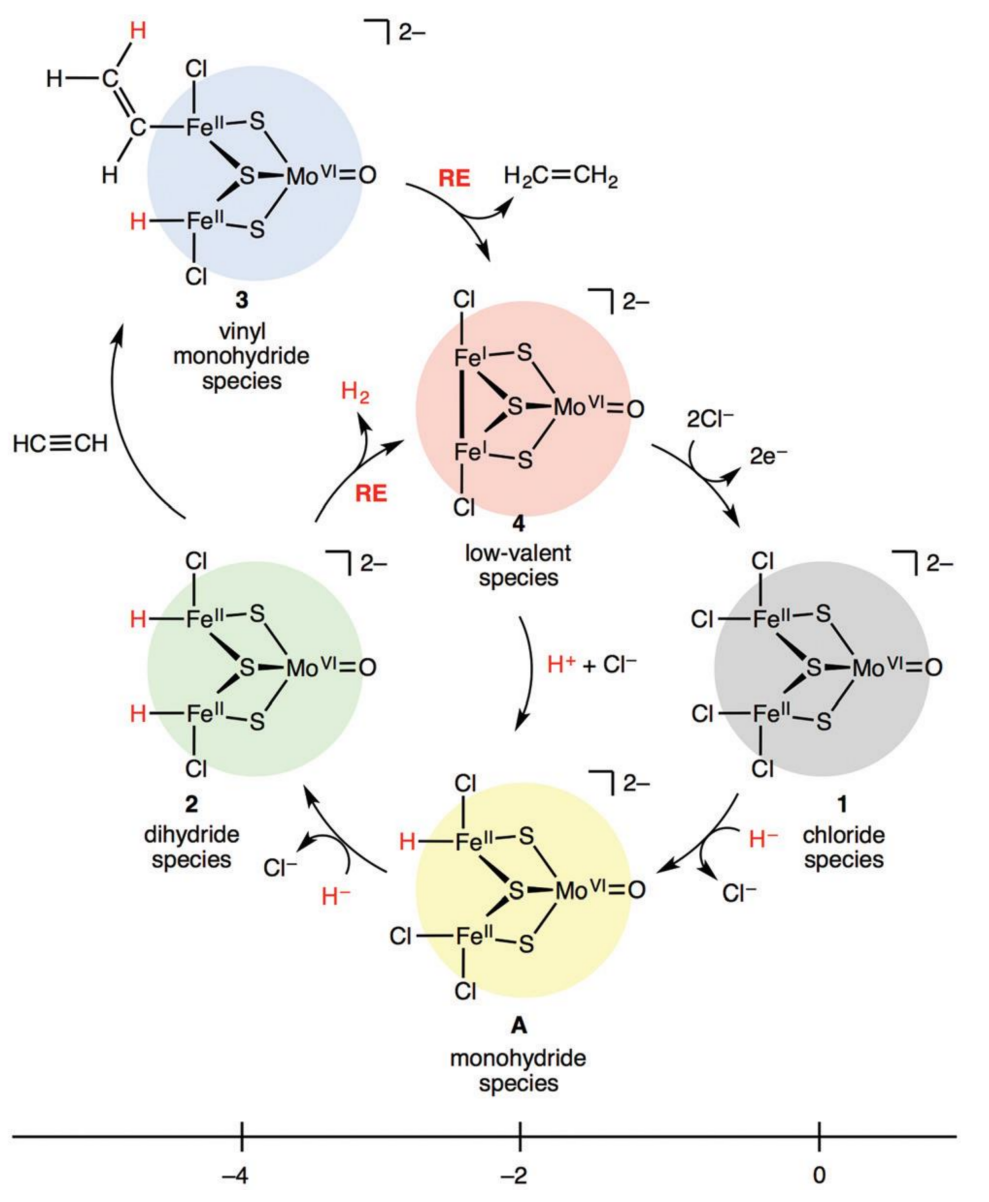
Proposed mechanism of reductive elimination (RE) of H_2_ from the dihydride Fe/Mo species **2** and C_2_H_4_ from the vinyl monohydride Fe/Mo species **3**. The scale of horizontal axis represents oxidation states 0 to –4, with the initial oxidation state of **1** corresponding to 0. Accordingly, the two and four electron-deprived species correspond to –2 and –4 oxidation states, respectively (reproduced with permission from [302]. Copyright 2016 the Royal Society of Chemistry.).

Nitrogenases (Nases) are able to reduce nitrogen to ammonia and acetylene to ethylene [295,296,297,298,299,300,301]. Inorganic Fe/Mo clusters with a [Fe_2_MoOS_3_] core resemble a part of FeMo-cofactor, the active center of nitrogenases [302]. The dihydride complex reduced acetylene to ethylene via the vinyl monohydride complex; the detailed mechanism, verified by using various combinations of reductants, substrates, and heavy water, is shown in Figure 25. Even very small (2.0 ± 0.5 nm) and probably planar Fe^2,3+^ oxide NPs on slightly acidic inorganic oxide supports (TiO_2_, ZrO_2_, or ZnO) are capable of mimicking the natural enzyme activities [303]. Surprisingly, these catalysts selectively and almost totally reduce acetylene and other alkynes not only in batch reactors, but also in flowing streams. Mixed Fe-oxides dissociate and transfer hydrogen onto alkynes in a chemo- and stereoselective manner. Cheap, nontoxic, ligand-free, and ambient condition-resistant, the solid iron catalysts ensure *cis*-selective semi-hydrogenation of different alkynes, including acetylene, in high yields and with good selectivities. 

#### 2.8.9. Pd-Free Bimetallic Catalysts: Pt/Sn

**Figure 26:**
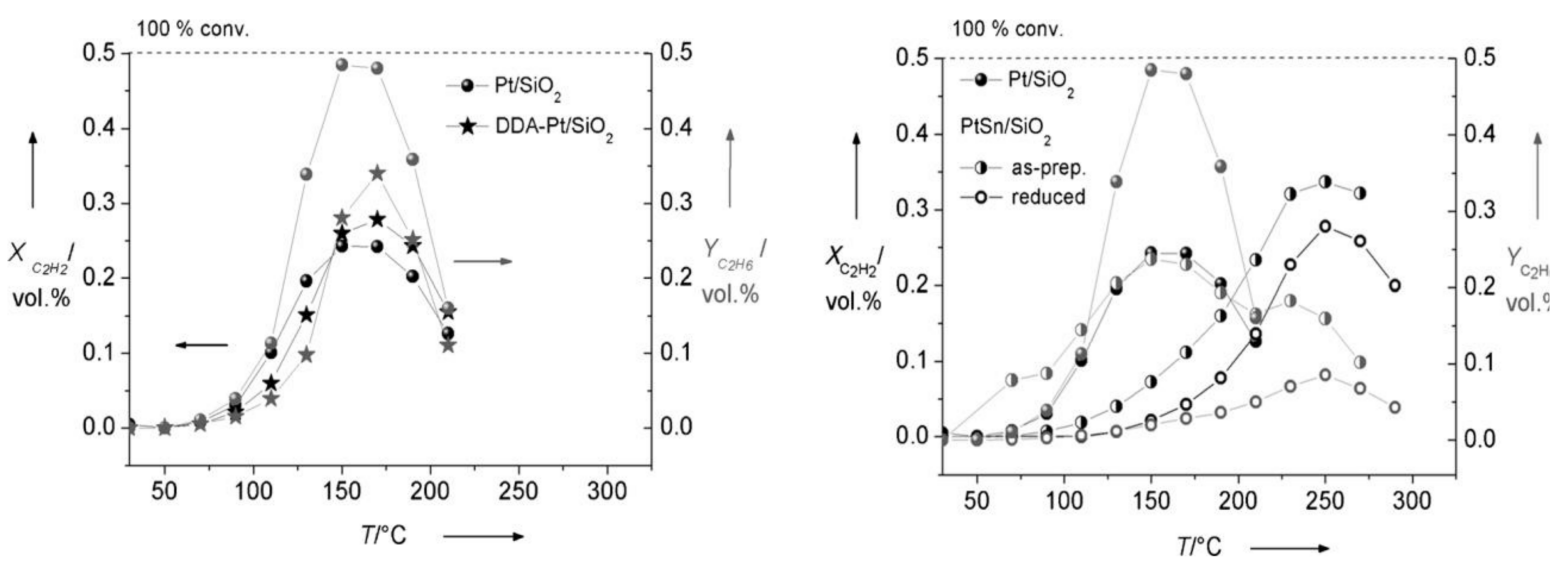
Acetylene conversion X (black symbols, left axis) and ethane time yield Y (gray symbols, right axis) as functions of temperature for: ***Left*** monometallic Pt/SiO_2_ (circles) and DDA-Pt/SiO_2_ (stars) catalysts. ***Right*** Pt/SiO_2_ (filled symbols), Pt/Sn(1:1)/SiO_2_ as prepared (half-filled symbols), and PtSn(1:1)/SiO_2_ prereduced (empty symbols) catalysts. Acetylene conversion is given by the difference in the reactor inlet and outlet concentrations in vol %. Ethane yield is given by vol % of ethane in the reactor off-gas. Reaction conditions: 0.5 vol % C_2_H_2_; 1 vol % H_2_; 70 vol % C_2_H_4_; rest N_2_; flow velocity 50 mLmin^−1^ (reproduced with permission from [304]. Copyright 2013 WILEY-VCH Verlag GmbH & Co. KGaA).

Colloidally prepared platinum and platinum-tin nanocrystals with controllable structural properties were tested in the acetylene semi-hydrogenation [304]. The ligand-free and ligand-capped bimetallic catalysts were separately compared with Pt NPs to sort out possible specific effects of Sn and organic ligands (Figure 26). Using dodecylamine (DDA) promoted a decrease in the unwanted ethylene hydrogenation. Organic ligands are thought to act by diluting or blocking the large Pt ensembles (E sites), while diminishing the surface enrichment of Sn, and thereby suppress ethylene hydrogenation. The bimetallic dodecylamine-capped Pt/Sn catalyst showed the best selectivity in this series, especially relative to the ligand-free and pure Pt catalysts.

#### 2.8.10. Pd-Free Bimetallic Catalysts: Cu/Au

**Figure 27:**
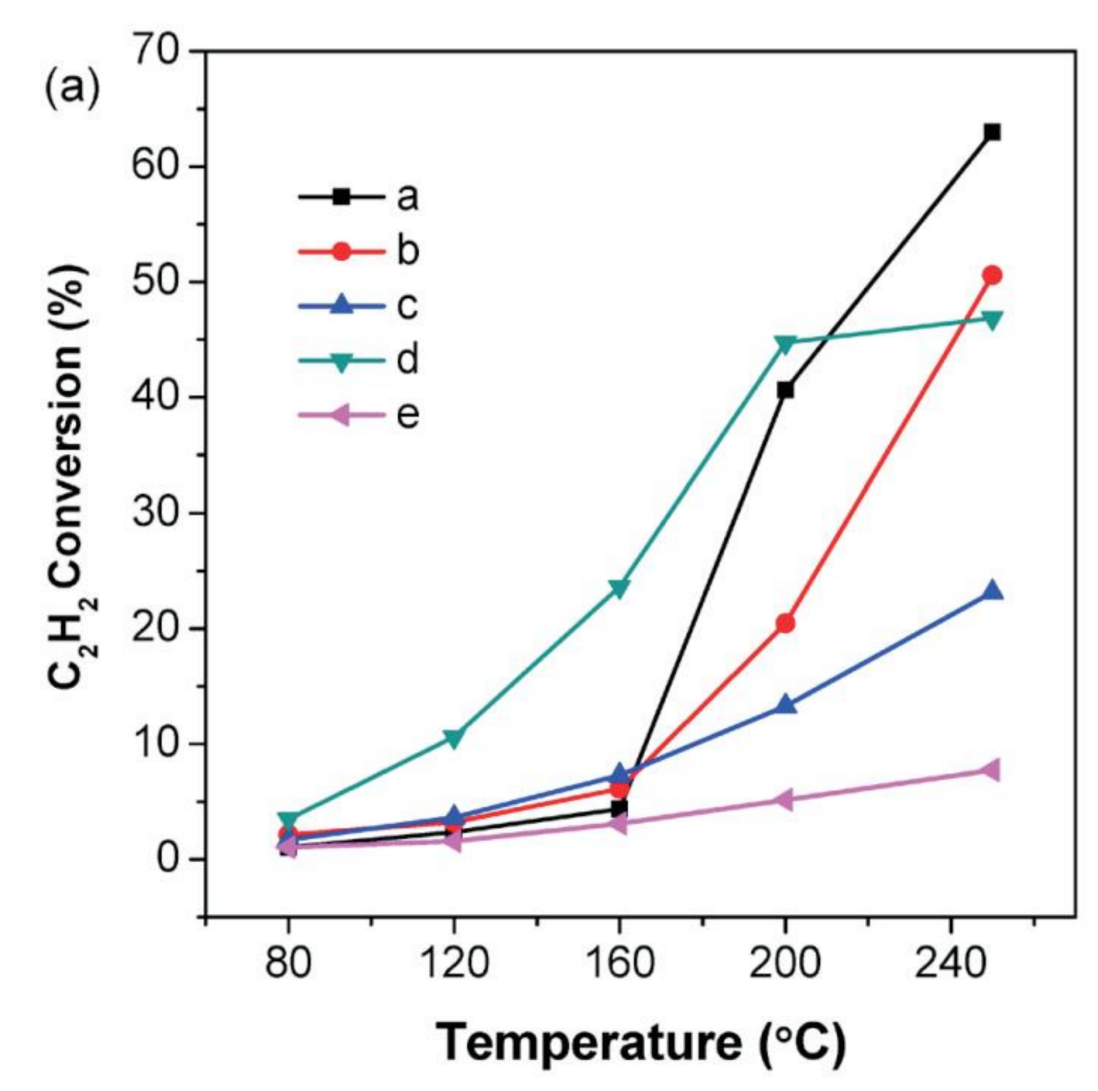

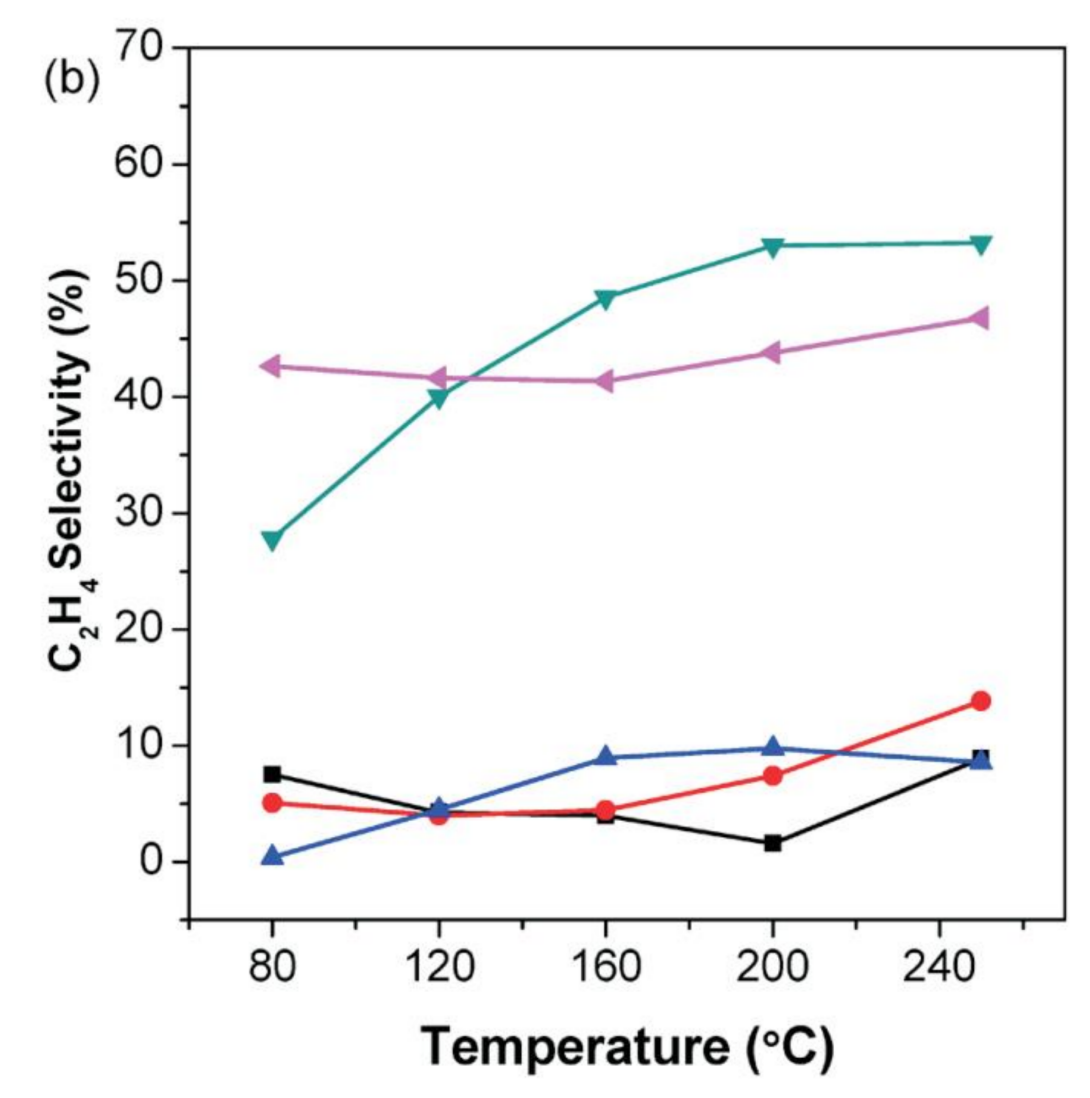
Reaction rate and ethylene selectivity over the SBA-15 supported Cu (**a**), Cu_10_Au_1_ (**b**), Cu_3_Au_1_ (c), Cu_1_Au_3_ (d), and Au (e) catalysts. C_2_H_2_/H_2_/He = 1.5/15/83.5, GHSV = 40,000 mL g^−1^_cat_ h^−1^ (reproduced with permission from [305]. Copyright 2013 WILEY-VCH Verlag GmbH & Co. KGaA).

The synergistic effect of bimetallic Cu/Au catalyst is provided by a combo of the electronic effect and the sintering resistance [305]. As it is, the bimetallic 1:3 Cu/Au catalyst shows an upgraded performance (Figure 27). Probably, the gold renders the copper more electron-deficient. The added amounts of Au reduce the size of Cu ensembles, which suppresses the C-C coupling reactions and results in better ethylene desorption. When Cu dominates in the alloy, the selectivity is very low; the addition of Au leads to an increase in selectivity.

#### 2.8.11. Non-Metallic Catalysts

A one-atom-thick layer of carbon atoms effectively catalyzed the selective acetylene semi-hydrogenation in the absence of a metal [306]. This graphene-based material was catalytically active at room temperature, but achieved its utmost performance at 110–120 °C, with 99% acetylene conversion and 21% ethylene conversion. GO was able to convert 50.6% of acetylene with no measurable ethylene hydrogenation at 150 °C, while rGO converted all acetylene with only 5% ethylene hydrogenation. These results indicate a very high potential of graphene-based catalysts (Table 3). Given that the graphene oxides were contaminated with ppm traces of metals (Fe and Mn) due to the presence of these metals in the parent graphite, the results were validated in additional experiments. Selected GO samples were purposefully contaminated with two known amounts of MnCl_2_ and PdCl_2_ before testing. The results confirmed the intrinsic catalytic potential of graphene materials. 

**Figure 28:**
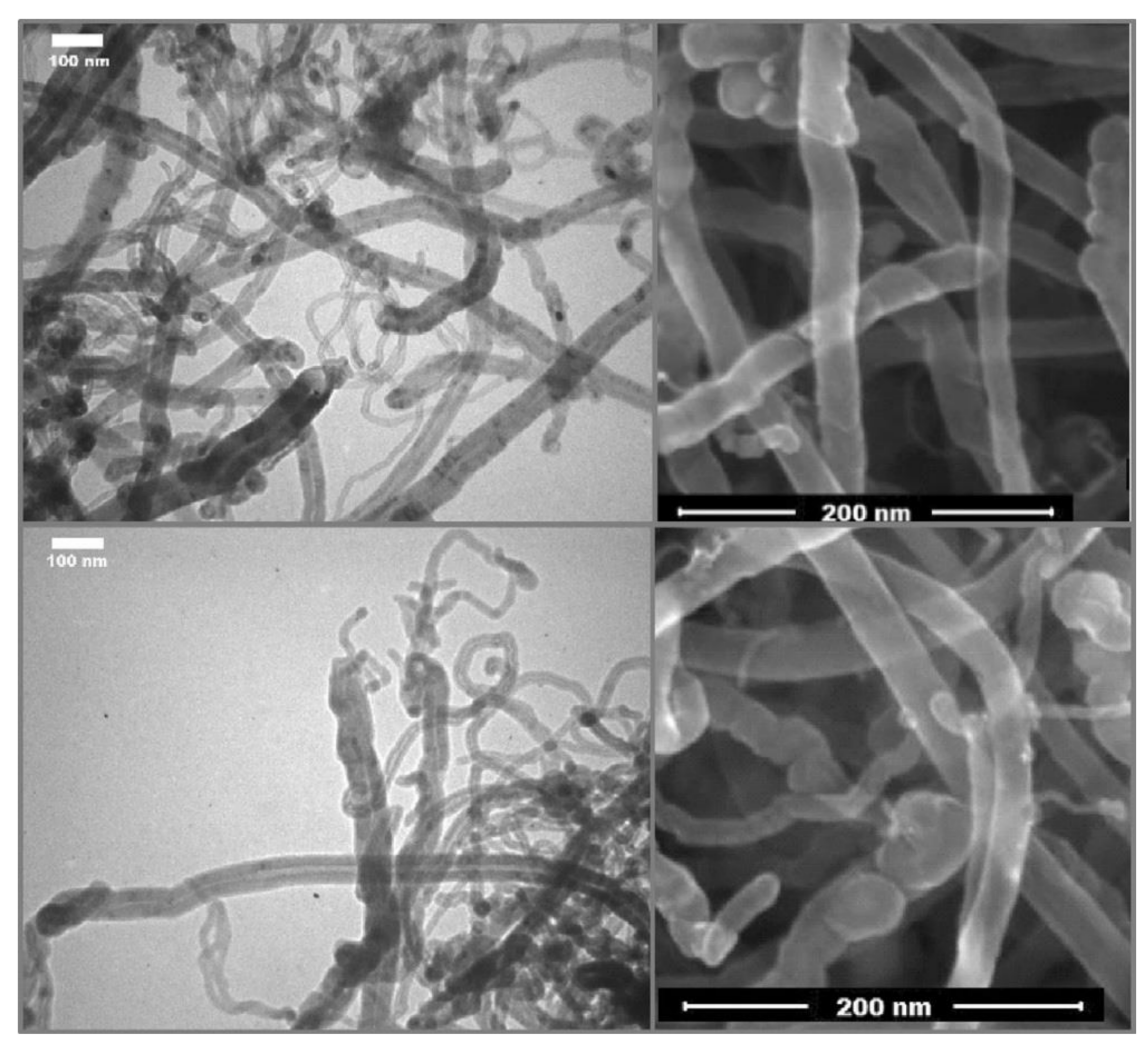
TEM and SEM images (on left and right, respectively) of the MWCNT and MWCNT-400 samples (top and bottom panels, respectively) (reproduced with permission from [307]. Copyright 2017 WILEY-VCH Verlag GmbH & Co. KGaA).

Multiwalled carbon nanotubes (MWCNTs) showed 90% acetylene conversion at 350 °C [307]. The ethylene selectivity constituted ~90% and loosely depended on the acetylene concentration. The higher temperatures led to acetylene decomposition and oligomerization, while the lower temperatures provided no support to acetylene conversion (3% at 80 °C). The nanotubes had cylindrical shape of 10 to 60 nm in diameter (Figure 28). The thicker tubes had thick walls and no sharp bends; the thinner tubes comprised thin walls and a large number of bends. Metal NPs contaminated the inner space of the tubes, and it was impossible to remove them even by heating at reflux in nitric acid. The high number of defects, the low oxygen content, and basicity of the surface compromised the MWCNT performance in selective acetylene hydrogenation.

In summary, the unique properties of Pd-based complexes and nanostructures make them the catalysts of choice for the ethylene-selective acetylene hydrogenation. Huge variety of the available Pd-based materials allows fine tuning of the catalytic process to optimize relative values of selectivity, conversion, and other parameters. Nevertheless, the Pd-based catalysts have certain drawbacks, hence the prospective relevance of their non-Pd and metal-free counterparts. 

### 2.9. Synthesis of Vinyl Chloride

**Scheme 94:**
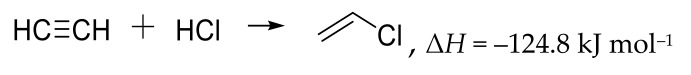
Acetylene hydrochlorination.

Polyvinyl chloride is a commodity chemical, which is employed in manufacturing of electric wires, water pipes and fittings, plastic membranes, and many other items. The monomer, vinyl chloride (VCM), is produced by two alternative routes. The first is based on chlorination and oxychlorination of ethylene, the second is the direct catalytic hydrochlorination of acetylene (Scheme 94).

In industry, the hydrochlorination proceeds at temperatures 130–180 °C with a catalyst, mercuric chloride HgCl_2_, supported on activated carbon (AC). However, HgCl_2_ is highly toxic and causes environmental pollution. An active search for environment-friendly alternative catalytic systems is currently under way.

Noble (Au, Pt, Pd) and some non-noble metal catalysts have been intensively studied as an alternative to catalize the hydrochlorination reaction. Hutchings and co-workers [308] was the first who demonstrated the high activity of AuCl_3_ and dynamic nature of catalyst caused Au^3+^ to Au^0^ reduction during the reaction. Treatment of the catalyst with *aqua regia* was proposed as an effective regeneration method for this system [309]. Specific effects of the *aqua regia* constituents, HCl/HNO_3_, as well as the metal/support modifications in the course of deactivation/regeneration of Au catalysts, were also demonstrated [310,311,312]. Despite HCl and HNO_3_ being able to affect the carbon support prior to deposition, the enhancement effect of *aqua regia* was observed only in the presence of Au during the impregnation step of the catalyst preparation. This was considered to be due to the oxidizing effect of HNO_3_ on both the carbon support and gold, in combination with the nucleation effect of HCl on gold particles over the carbon surface. The activity depends on the presence of Au^3+^ species in the catalyst, and their spatial distribution in particular (not only the concentration matters).

**Figure 29:**
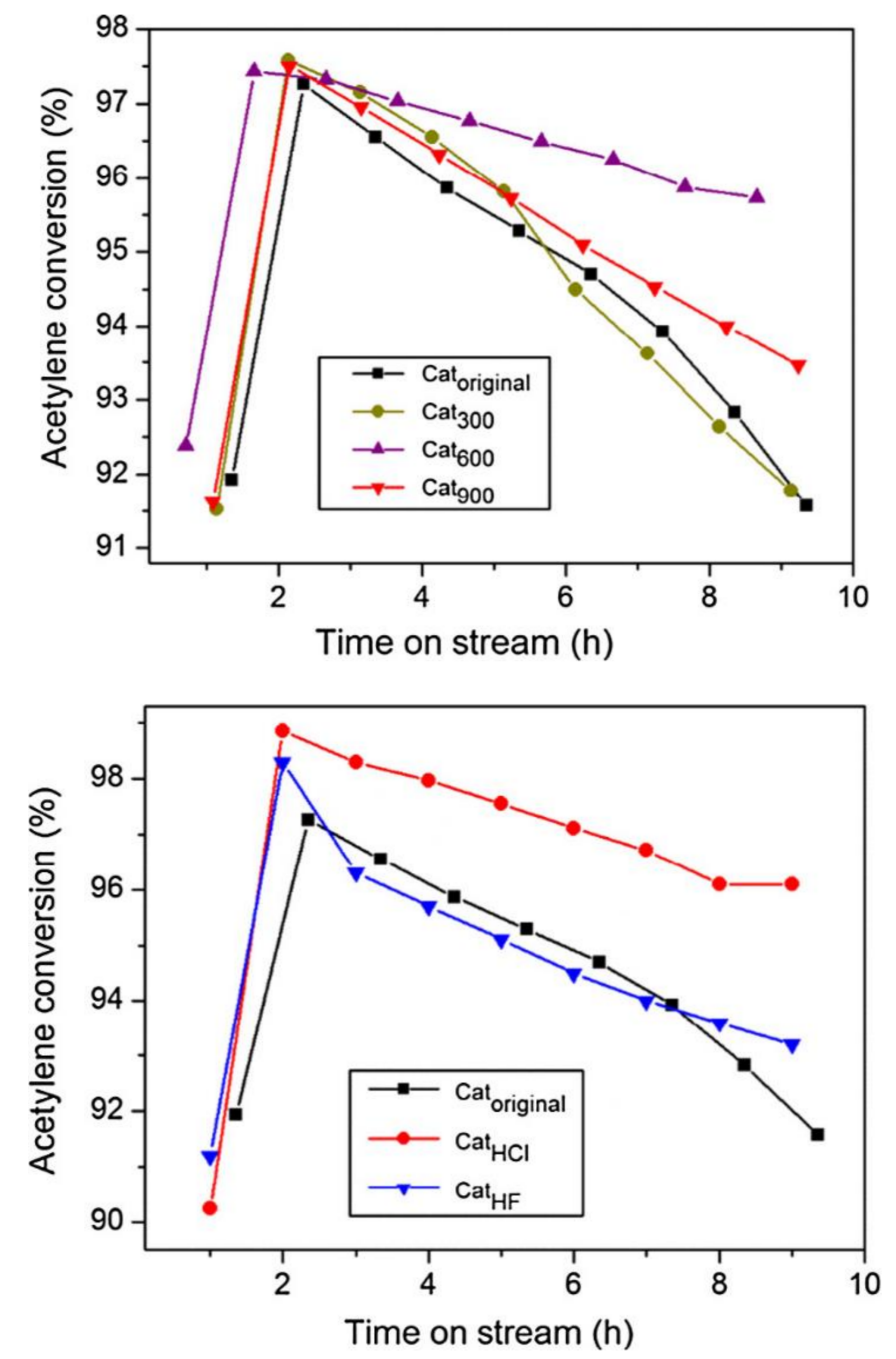
Acetylene conversion by AuCl_3_/C catalysts: temperature activation (**up**) and acid treatment (**down**). Reaction conditions: temperature = 180 °C, C_2_H_2_ GHSV = 360 h^−1^, feed volume ratio between HCl and C_2_H_2_ = 1.1 (reproduced with permission from [313]. Copyright 2015 Elsevier Inc.).

Despite the glorious history of the catalysis by gold, its mechanistic understanding is still incomplete. A series of gold catalysts was prepared by using the aqueous HCl/HF pretreated activated carbon at 300 °C (Cat_300_), 600 °C (Cat_600_), and 900 °C (Cat_900_, Figure 29) [313]. The C_2_H_2_ temperature-programmed desorption of the catalysts was also measured. Compared with Cat_original_ and Cat_HF_, the Cat_HCl_ catalyst showed an increased initial activity with the same decrement over time.

The results indicate that dispersibility of gold positively correlates with the stability of the catalyst. Accordingly, the C_2_H_2_ adsorption/activation capacity negatively correlates with both the catalyst stability and gold dispersibility. Which means, that the active site is not composed entirely of gold dispersed on the carbon surface, and that the carbon itself works as a constituent part of the site responsible for the acetylene activation.

Stability of Au-catalysts can be enhanced by inhibiting Au^3+^ reduction [314], which can be achieved by strengthening the adsorption of hydrogen chloride to the catalyst. The enhanced catalytic activity and stability of AuCl_3_/PPy–MWCNTs in acetylene hydrochlorination, as compared with AuCl_3_/MWCNT, is explained as follows. During the reaction, a lone pair on the nitrogen atom of pyrrole is transferred to the Au^3+^ center via the metal-support interaction. This event increases the electron density of the Au^3+^ center to strengthen the bond between HCl and AuCl_3_. Other N-doped material-based catalysts, such as carbon [315], carbon nanotubes [316], SiC [317], graphene [318] were studied with some interesting results; however, the low conversion efficiencies and high energy demands do not contribute to their commercial applicability. The nitrogen-containing ligands are able to inhibit the reduction of Au^3+^ to Au^0^ [319], although the synthesis of AuCl_3_ complex with 1,10-phenanthroline [AuCl_2_(phen)]Cl and its successful application in acetylene hydrochlorination have been reported [320]. 

Catalytic stability of Au catalysts can be improved by addition of a second metal component, for example, CoCl_3_ [321] or CsCl [322,323]. Currently available Au catalysts for hydrochlorination contain more than 1 wt % of Au. Addition of Cu as a third metallic component to an AC-supported AuCs catalyst allowed obtaining of a trimetallic catalyst with enhanced activity and stability at ~0.25 wt % Au loading [324]. Particularly remarkable activity enhancement in this series was shown by a catalyst with 1:1:4 Au:Cu:Cs weight ratio, at turnover frequency of 73.8 min^−1^ based on Au. The synergy between Au, Cu, and Cs is associated with enhancements in dispersion of Au particles, stabilization of Au in the state of Au^3+^, and adsorption kinetics of the C_2_H_2_ substrate molecules.

Trimetallic Au–Co(III)–Cu(II) catalysts were prepared by using spherical AC (SAC) as a support [325]. The best performance in this series was achieved with Au1Co(III)3-Cu(II)1/SAC, showing 99.9% VCM selectivity at 99.7% acetylene conversion.

ACs have micropores and no interconnected porous network. Mesoporous carbons with regularly interconnected 2–50 nm mesopores could provide an excellent alternative support for the acetylene hydrochlorination catalysts. Mesoporous carbon materials with controllable pore sizes within the range of 5.6–40.2 nm were synthesized using colloidal silica as hard templates and boric acid as a pore expanding agent. The Au catalysts on mesoporous carbon showed excellent performances with increased acetylene conversion. Another material, γ-Al_2_O_3_, was used as support for an AuCl_3_-CuCl_2_ bimetallic catalyst. The AuCl_3_-CuCl_2_/γ-Al_2_O_3_ catalyst promoted 97% acetylene conversion, which is close to AuCl_3_–CuCl_2_/AC. The advantages of using γ-Al_2_O_3_ as a support are provided by its mechanical strength and mesoporous structure. Some zeolite-supported Pd catalysts [326,327,328] are also good for acetylene hydrochlorination.

Another important trend in the acetylene hydrochlorination catalysis involves ruthenium. The calculated activation barriers for acetylene hydrochlorination over HgCl_2_, AuCl_3_ and RuCl_3_ constitute 16.3, 11.9, and 9.1 kcal mol^−1^, respectively [329]. Certain advantages of Ru catalysts make them a proper alternative to other metal catalysts.

**Figure 30:**
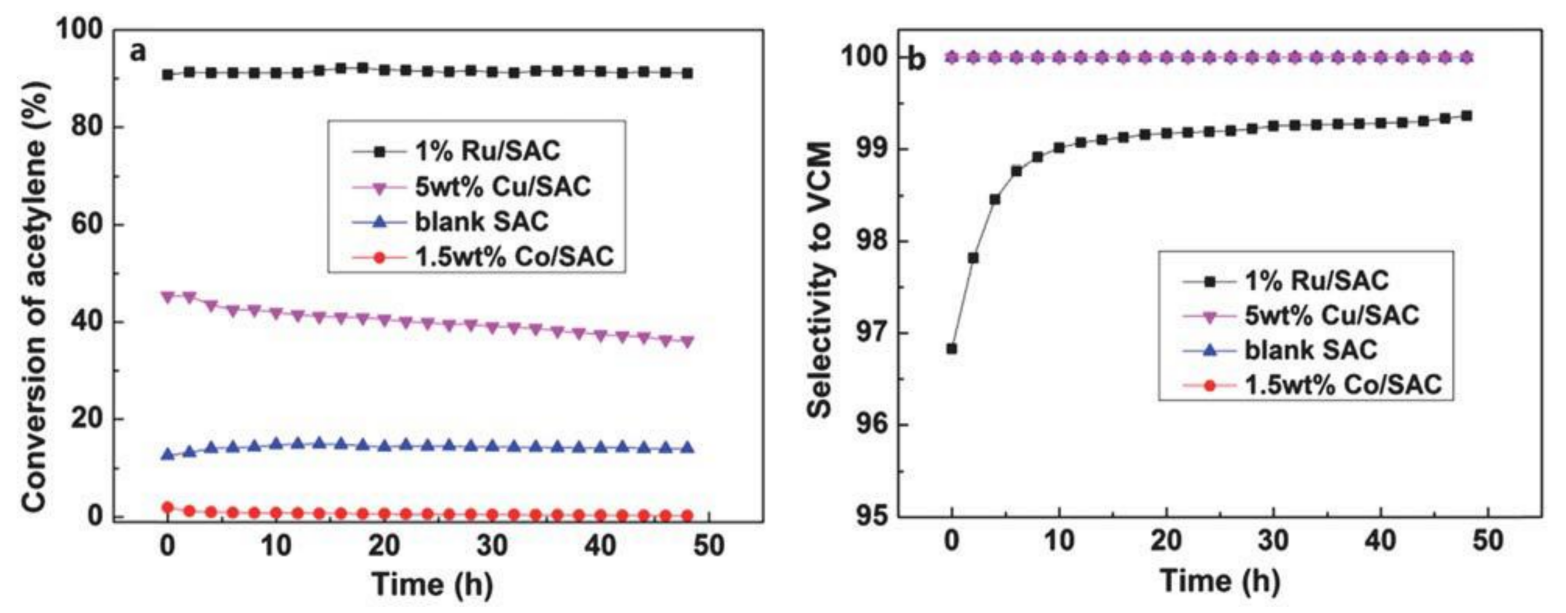
Acetylene conversion (**a**) and VCM selectivity (**b**) over monometallic Ru, Cu, and Co catalysts (reproduced with permission from [330]. Copyright 2013 the Royal Society of Chemistry.).

**Figure 31:**
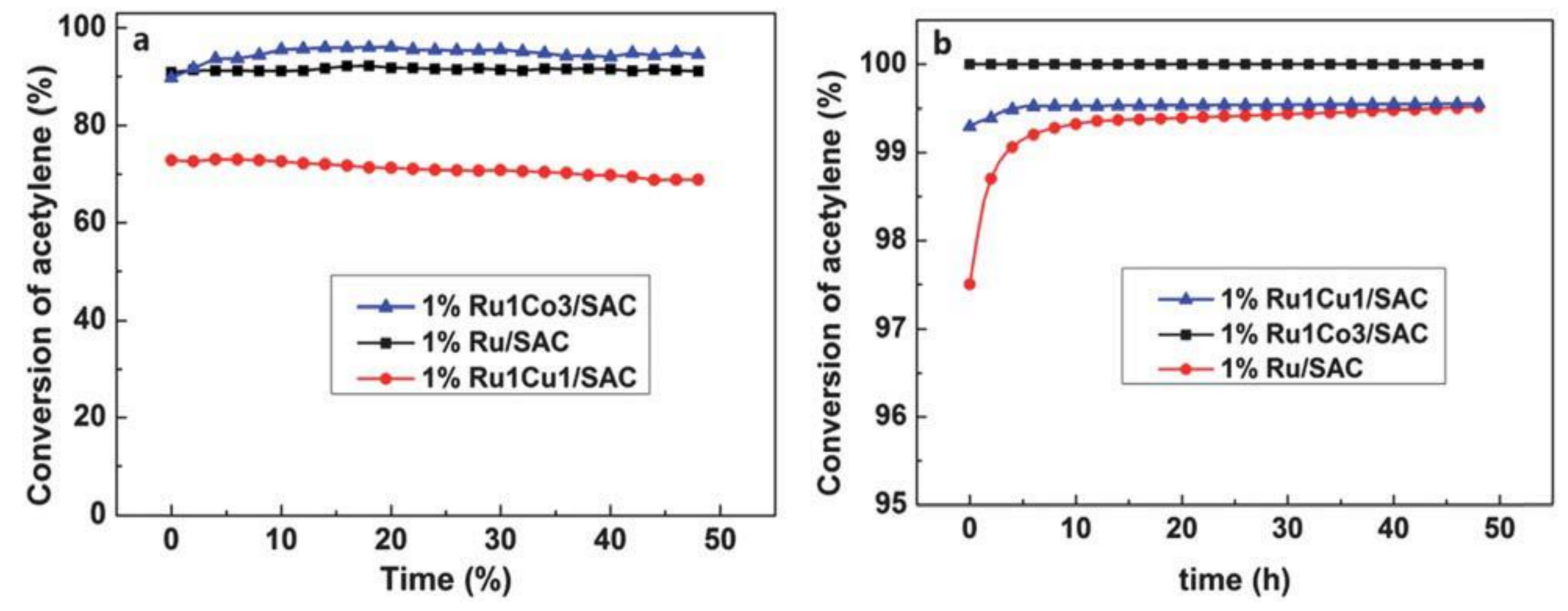
Acetylene conversion (**a**) and VCM selectivity (**b**) over 1%Ru, 1%Ru1Cu1, and 1%Ru1Co3 catalysts (reproduced with permission from [330]. Copyright 2013 the Royal Society of Chemistry.).

Catalytic performance of Ru-catalysts including monometallic Ru, bimetallic Ru–Cu and Ru–Co with spheric active carbon (SAC) as the support was studied [330]. C_2_H_2_ conversion and the selectivity to vinyl chloride (VCM) over monometallic and bimetallic Ru-catalysts are depicted at Figure 30 and Figure 31 (reaction conditions: T = 170 °C, C_2_H_2_ GHSV = 180 h^−1^, V(HCl)/V(C_2_H_2_) = 1.1).

The 1%Ru/SAC catalyst is capable of 91% acetylene conversion at 48 h on stream. Addition of cobalt significantly enhances its catalytic activity leading to 95% acetylene conversion at 48 h on stream, whereas addition of copper results in lower acetylene conversion. A remarkable 99.9% VCM selectivity at 95.0% acetylene conversion has been shown by RuCl_3_/MWCNT catalyst under 170 °C, C_2_H_2_ GHSV= 90 h^−1^, and V(HCl)/V(C_2_H_2_) = 1.1 for 10 h [331]. 

Potassium represents a promising promoter for Ru catalysis [332]. RuCl_3_-KCl/SAC catalysts perform better than Ru/SAC catalysts, with the optimal conversion of acetylene over Ru1K1/SAC reaching 93.4% under 170 °C, C_2_H_2_ hourly space velocity (GHSV) of 180 h^−1^, and V(HCl)/V(C_2_H_2_) = 1.1 feed volume ratio.

**Figure 32:**
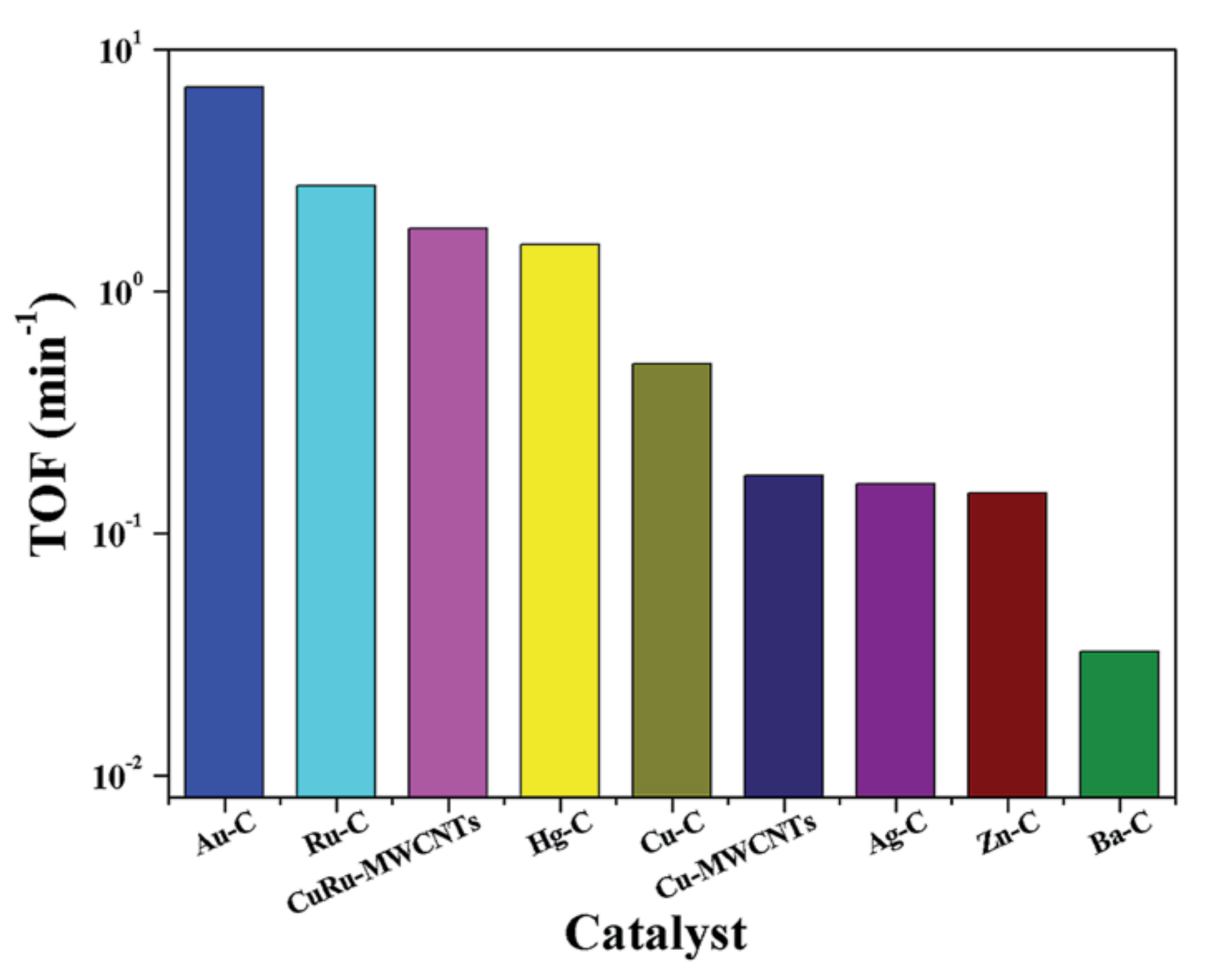
Calculated TOF values for Cu400Ru/MWCNTs in comparison with a variety of reported catalysts. The calculated TOF values for the different catalyst materials reported in literature and the Cu400Ru/MWCNTs catalyst material. (Reproduced with permission from ref. [333]. Copyright 2015 the Royal Society of Chemistry.).

Among a variety of non-noble metals, Cu occupies a worthy position due to its low cost and good thermostability. A catalyst with 400 ppm Ru and 4.24 wt % Cu on carbon nanotubes was highly active (TOF = 1.81 min^−1^) due the synergistic effect between Cu and Ru [333]. Despite the slower acetylene hydrogenation rates in comparison with AuCl_3_ catalysts, this catalyst is superior to other metal complexes known to catalyze this reaction (Figure 32).

Nitrogen-doping of AC [334] and its chemical modification by consecutive nitration, amination and reaction with pyridine [335] were also performed to improve the activity and stability of Ru-based catalysts.

Despite the obvious advantages of Ru catalysts, their usage is fraught with risks of coke deposition. The imidazolium-based ionic liquids 1-butyl-3-methylimidazolium tetrafluoroborate ([BMIM]BF_4_), 1-butyl-3-methylimidazoliumhexafluorophosphate ([BMIM]PF_6_), and 1-butyl-3-methylimidazolium chloride ([BMIM]Cl), were tested as possible inhibitors of the coke deposition [336]. An exemplary Ru10%[BMIM]BF_4_/AC catalyst achieved 99.8% VCM selectivity at 98.9% acetylene conversion under 170 °C and C_2_H_2_ (GHSV) of 180 h^−1^. The results indicate that the addition of imidazolium-based ionic liquids can significantly improve the dispersion of ruthenium species and prevent the coke deposition.

### 2.10. Emerging Fundamental Concepts for Alkynes Transformations

As has been shown in the previous sections (2.1–2.9), the chemistry of acetylene is diverse and occupies a unique position in organic synthesis. Undoubtedly, the synthetic utility of acetylene will be further expanded to a number of new opportunities. Effective translation of new concepts from the emerging areas of alkyne chemistry plays an important role and essentially increases synthetic potential of acetylene. Examples of useful concepts from the area of substituted alkyne chemistry will be briefly discussed in this section. It should be noted, that no detailed coverage of alkyne chemistry will be provided in this section. Instead, it provides a representative selection of recent examples as conceptual highlights. The key emphasis is made on efficient increase in molecular complexity, which is a priority goal in modern organic chemistry [337].

**Figure 33:**
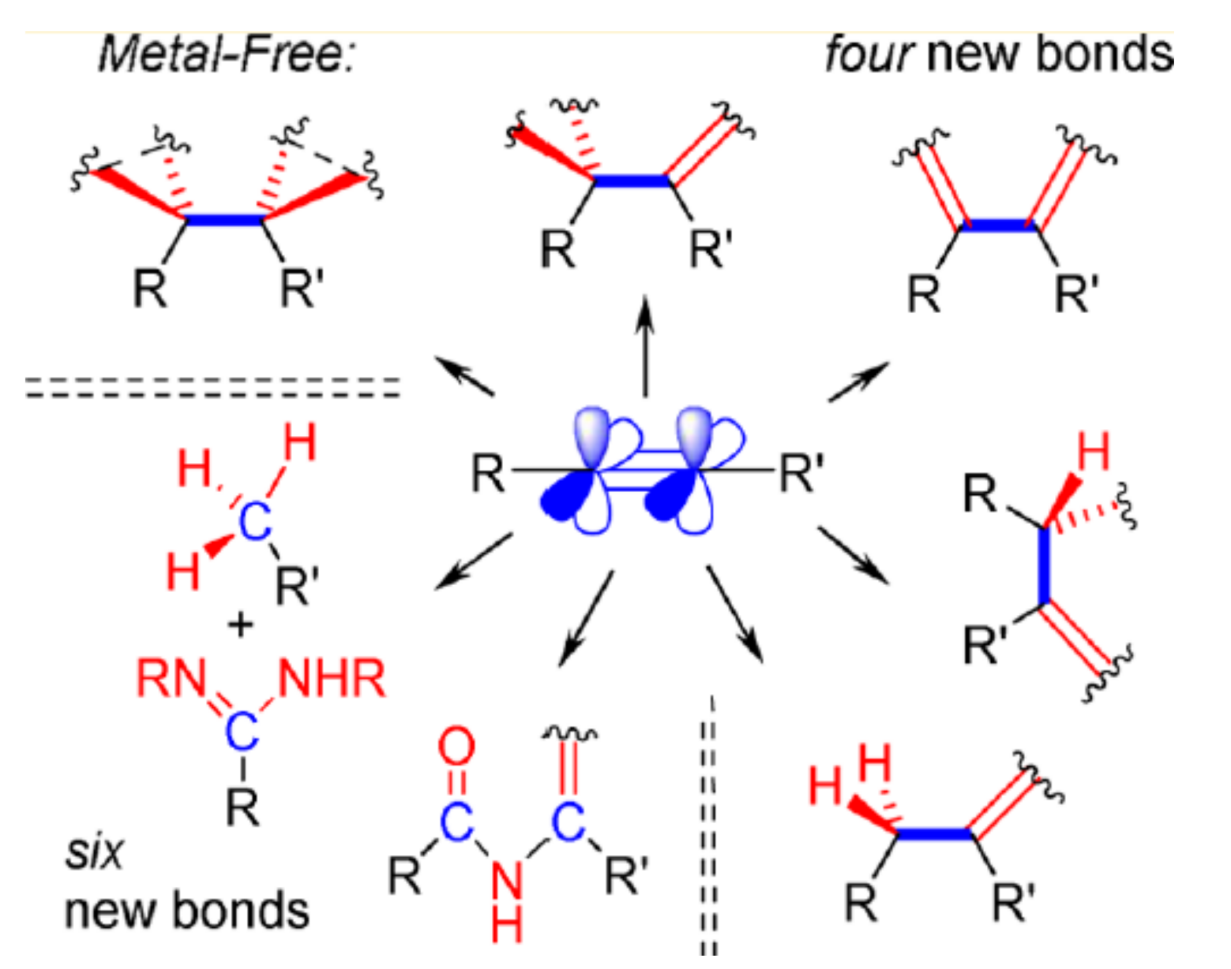
Using both alkyne π-bonds as “two functional groups in one package”. (Reproduced with permission from ref. [338]. Copyright 2013 American Chemical Society.).

The orthogonality of the two π-orbitals in alkynes can be used for the metal-free cascade transformations [338]. The existing examples include ionic chemistry of neutral hydrocarbons, preparation of radicals without radical initiators, generation of excited states without light, “1,2-dicarbene reactivity” of alkynes in “boomerang” radical processes, selective conversion of alkynes into carbonyl compounds, and full disassembly of the alkyne moiety (Figure 33) [338]. This feature provides an access to a wide scope of organic molecules.

**Figure 34:**
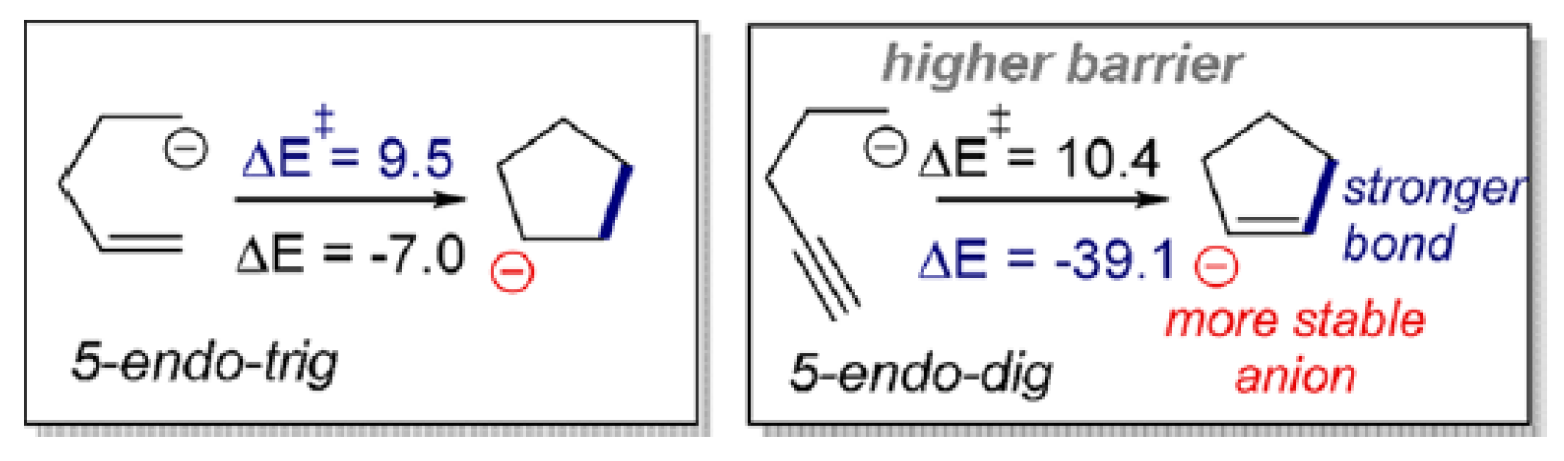
Comparison of alkene and alkyne reactivities in terms of kinetics and thermodynamics (reproduced with permission from [339]. Copyright 2018 American Chemical Society).

The unique property of triple bond is in a high exergonic effect in comparison with the analogous double bond. It explains one of the reasons of the richness of acetylene chemistry in comparison with the unsaturated compounds with double bond (Figure 34) [339]. The high exergonic effect provides a driving force for a number of fascinating transformations. On the other hand, the unique combination of electronic and structural properties balances the activation energy barriers and favors the formation of relatively stable intermediate species. Trapping of relatively stable intermediates provides the mechanistic means for a diversity of organic functionalizations.

**Figure 35:**
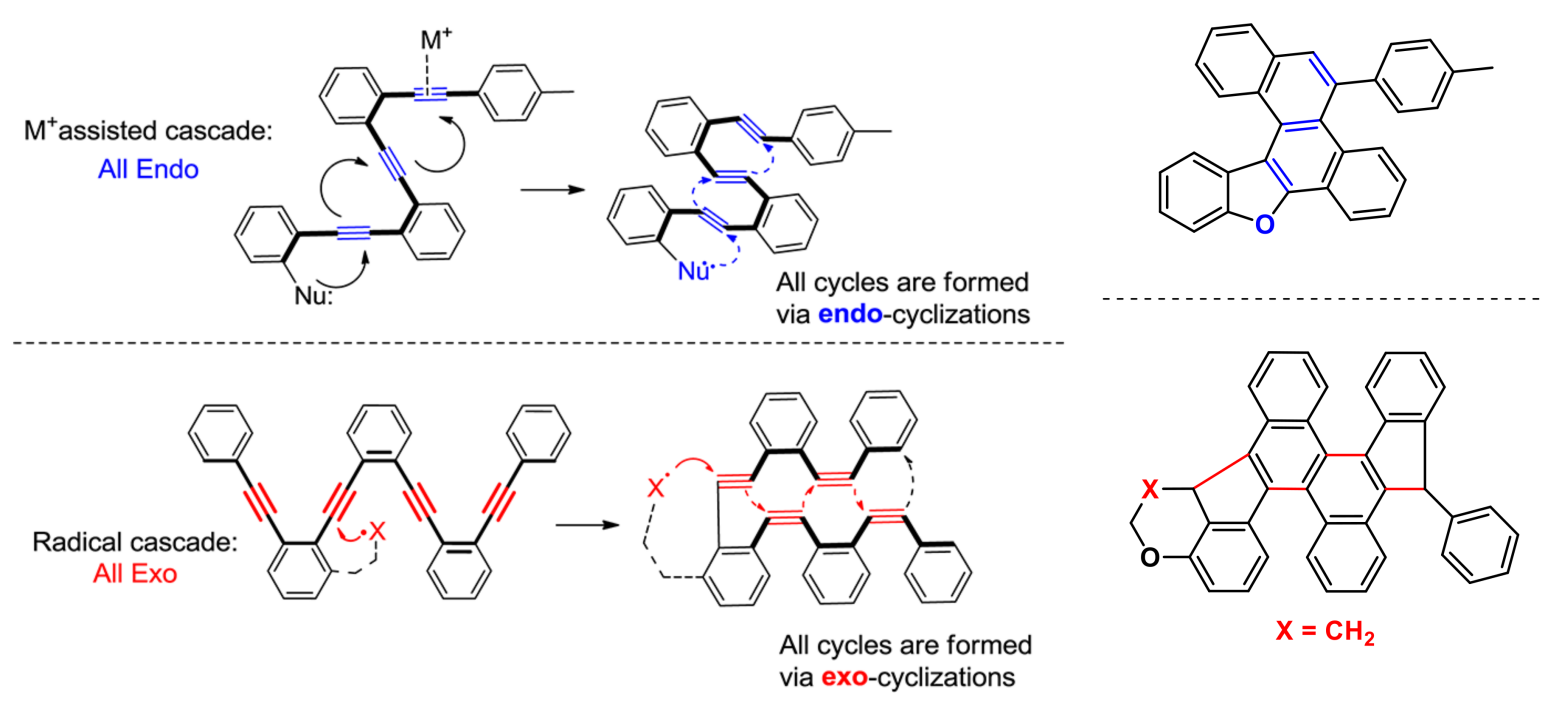
Examples of polyaromatic self-assembling based on the cascade oligocyclization of alkynes (reproduced with permission from [341]. Copyright 2012 American Chemical Society).

Cascade reactions with polyacetylenic derivatives have been recently described as an effective *one-pot* route to increased molecular complexity [338,339]. Interestingly, a minor activating influence on a triple bond of the polyacetylene molecule is sufficient to initiate the avalanche-like process with all the other reactive centers (Figure 35). The use of metal catalysis or a radical initiator triggers the cascade process and directs the pathway toward polyaromatics [340,341].

The methodology is advantageous since it requires the minimal quantities of a catalyst or other initiator. This advantage is explained by the specific properties of triple bond mentioned above (see Figure 34); the mechanism has been discussed in the literature [341]. Compatibility of the cascade reactions with Sonogashira cross-coupling and other transformations [342,343] makes them relevant for fine organic synthesis. Although this concept of increasing molecular complexity was not yet applied to the unsubstituted acetylene, we believe that its potential should be thoroughly evaluated.

**Scheme 95:**
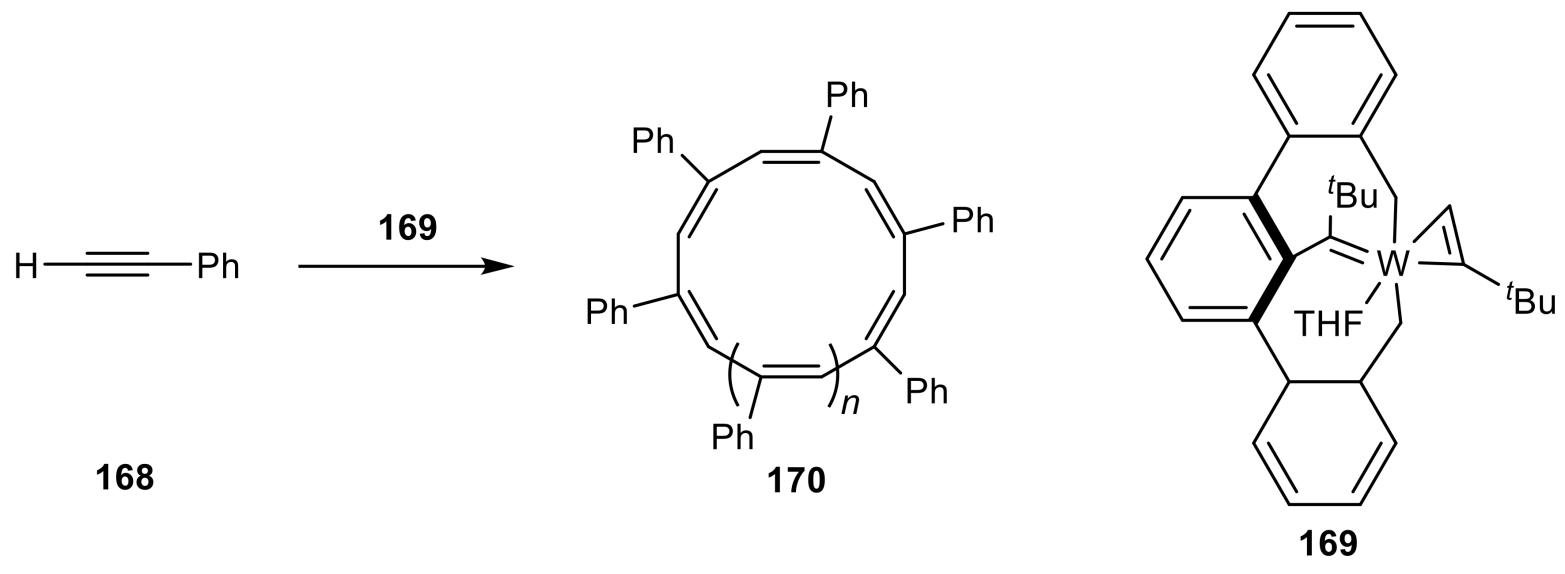
Phenylacetylene polymerization.

Synthesis of macrocyclic polyethynes is another way of rapid assembling of complex molecular structures, especially in combination with the involvement of energy-reach macrocycles in further transformations. In a recent article, an example of successive phenyl acetylene **168** polymerization was described [344]. By subsequent propagation-insertion on a catalyst **169**, a cyclic polyethyne **170** of M*_n_* = 45,600 Da was obtained in a high yield (Scheme 95). Since the cyclic polymers have no terminal groups, they show a number of unique physical properties [344]. For example, the density [345], refractive index [346,347], glass-transition temperature [348], viscoelasticity [349] and surface properties [350] of cyclic polymers differ from those of their linear analogues. Partial replacement of alkynes with acetylene allows obtaining a unique cyclic copolymer material, which is worth studying.

Other potential applications of acetylene are related to additive manufacturing technologies. These technologies have been dramatically upgraded in recent years, due to the progress in research and industrial development. 3D printing provides a rapid link from computer-designed digital models to prototyping/production of materials and objects of desired shape and composition. 3D printing at the molecular level seems promising for direct construction of complex molecules; however the idea is highly challenging. To pump it up to the level of technology, it would be necessary to select a scope of simple molecules (versatile, accessible and modifiable) to be used as building blocks. The simplest low-molecular weight hydrocarbons—methane, ethylene and acetylene—can be certainly considered. The key restrictions for molecular 3D printing with hydrocarbons are related to the limited possibilities of the C-H-activation and atom-economical modification. By contrast with more saturated candidates, acetylene is capable of being engaged in two atom-economical addition reactions; it has a relatively reactive hydrogen atom at each of the carbons and no unreactive C-H bonds (Table 4) [351]. Hence, it is highly probable that alkyne molecules will serve as a main building material for molecular 3D printing [351]. 

In this section, only few examples are shown to illustrate some important directions of acetylene chemistry. Given the rapidity of contemporary progress, many other possibilities of acetylene utilization may arise in the nearest future.

## 3. Conclusions

Chemistry of alkynes is a well-developed area, which represents a fundamental tool for organic synthesis. In contrast with other alkynes, the use of acetylene in research practice is rather limited, mostly due to the technical difficulties of handling gaseous acetylene and for the concomitant safety reasons. Circumvention of these obstacles would undoubtedly boost the use of acetylene in organic synthesis. A corresponding rapid trend observed in recent years favors a renaissance of acetylene chemistry, with full actualization of its rich potential.

Literature analysis carried out in the present review shows a wide scope of chemical transformations, which relate acetylene with such processes as vinylation, cross-coupling reactions, access to substituted alkynes and synthesis of heterocycles. These transformations provide an access to new drugs, pharmaceutical substances, biologically active molecules and valuable building blocks (synthones). Safe and reliable procedures for preparation of vinyl derivatives are in high demand, as they allow production of monomers for the synthesis of polymers and copolymers with unique properties. Particularly tempting are applications of acetylene-derived functionalized molecules in materials science.

Besides the noticeable progress in acetylene chemistry, several problems still remain a challenge. Of particular importance is the development of convenient practical procedures with high safety standards to utilize acetylene-based syntheses in everyday laboratory practice. Another challenging topic concerns the development of Green reactions involving acetylene, directed towards minimization of wastes and increased cost-efficiency.

Exceptional reaction capacities of acetylene are associated with several different reaction channels and complex mechanisms. Comprehensive understanding of the mechanisms of organic transformations of acetylene is one of the primary challenges. Even the well-known acetylene transformations (for example, selective hydrogenation or hydrochlorination) have rather complex mechanisms, which must be taken into account when developing chemical procedures and industrial technologies.

Considered in the present review, the current research trends clearly show that the great potential of acetylene molecule is far from being completely utilized, and a number of fascinating studies can be anticipated in the nearest future. Translation of new basic findings from alkyne chemistry to applied fields is of special importance. Cutting-edge research projects reinforce a dedicated search for effective routes of transforming acetylene into the products of increasing molecular complexity. This direction will remain a top priority in the nearest future.

## Figures and Tables

**Table 1:** Byproducts formed at 120 min of on-stream hydrogenation (323 K, 1 bar) for acetylene only, ethylene only, and acetylene/ethylene mixture.

	Oligomers/FID Signal	Methane/FID Signal
Acetylene only		
Pd/TiO_2_	248,173	61,404
Pd/TiO_2_ + 3‰ CO	296,540	103,000
PPh_3_ 2.5 Pd/TiO_2_	315,656	-
Ethylene only		
Pd/TiO_2_	170	3445
Pd/TiO_2_ + 3‰ CO	799	3392
PPh_3_ 2.5 Pd/TiO_2_	290	3100
Mixed acetylene/ethylene reaction		
Pd/TiO_2_	52,415	14,763
Pd/TiO_2_ + 3‰ CO	174,911	-
PPh_3_ 2.5 Pd/TiO_2_	4969	9393
PPh_3_ 10 Pd/TiO_2_	4332	6262

**Table 2:** Acetylene conversion and ethylene/ethane/oligomer selectivities over 10% Cu/Al_2_O_3_ and 1.67% Pd/Al_2_O_3_.

Catalyst	Temperature (K)	Acetylene Conversion (%)	Ethylene Selectivity (%)	Ethane Selectivity (%)	Oligomer Selectivity (%)
10% Cu/Al_2_O_3_	323	9.59	53.2	9.9	37.0
	373	60.71	60.5	8.6	30.8
	423	99.20	71.5	8.0	20.5
1.67% Pd/Al_2_O_3_	323	85.17	0.0	93.1	6.9

**Table 3:** Catalytic performances of graphene-based catalysts.

Catalyst	Conversion (%)		Product Distribution (%)				
	C_2_H_2_	C_2_H_4_	C_2_H_4_	C_2_H_2_	C_2_H_6_	C_4_	C_4_+
Gr	81.0	14.0	92.2	3.2	1.1	3.5	—
Gr *	99.0	21.0	91.6	0.9	1.2	6.3	—
GO	22.5	0	48.1	5.0	0.3	46.6	—
rGO	87.5	4.7	55.1	1.2	1.3	42.4	—
(N)Gr	26.7	6.7	62.5	7.2	0.7	11.5	25.2
(P)Gr	14.6	2.7	72.7	8.8	0.7	17.7	0.1
(S)Gr	24.7	6.4	54.1	6.3	0.4	0.0	39.1

GO, graphene oxide; Gr, graphene; rGO, reduced GO. Results of selective catalytic acetylene hydrogenation in the presence of ninefold excess of ethylene. Reaction conditions: 10% C_2_H_2_ in 90% C_2_H_4_; (C_2_H_2_+C_2_H_4_):H_2_ = 1:3, 110 °C, 7 mg of catalyst, gas hourly space velocity (GHSV) of 14.600 mLh^−1^ g^−1^. * 10% C_2_H_2_ in 90% C_2_H_4_; (C_2_H_2_ + C_2_H_4_):H_2_ = 1:3, 120 °C, 7 mg of catalyst, GHSV of 7.800 mLh^−1^ g^−1^.

**Table 4:** Potential chemical building blocks for a molecular 3D printer [351].

Building Block Molecule	Number of Low-Reactive Substituents at the C Atom	Number of Activated Substituents at the C Atom	Number of Atom-Economical Steps
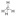	4	0	0
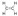	2	0	1
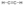	0	1	2
